# Redisposition of apiosporous genera *Induratia* and *Muscodor* in the Xylariales, following the discovery of an authentic strain of *Induratia apiospora*

**DOI:** 10.1186/s40529-023-00372-1

**Published:** 2023-04-13

**Authors:** Marjorie Cedeño-Sanchez, Rahel Schiefelbein, Marc Stadler, Hermann Voglmayr, Konstanze Bensch, Christopher Lambert

**Affiliations:** 1 Department Microbial Drugs, Helmholtz-Centre for Infection Research GmbH, Inhoffenstraße 7, 38124 Braunschweig, Germany; 2 Institute of Microbiology, Technische Universität Braunschweig, Spielmannstraße 7, 38106 Braunschweig, Germany; 3 Department of Botany and Biodiversity Research, University of Vienna, Rennweg 14, 1030 Vienna, Austria; 4 Department of Forest and Soil Sciences, Institute of Forest Entomology, Forest Pathology and Forest Protection, BOKU-University of Natural Resources and Life Sciences, Franz- Schwackhöfer-Haus, Peter-Jordan-Straße 82/I, 1190 Vienna, Austria; 5 Westerdijk Fungal Biodiversity Institute, Uppsalalaan 8, 3584 CT Utrecht, The Netherlands; 6 Department of Cell Biology, Helmholtz-Centre for Infection Research GmbH, Inhoffenstraße 7, 38124 Braunschweig, Germany

**Keywords:** Fungi, Lectotypification, Sordariomycetes, Phylogeny, New combination

## Abstract

**Background:**

The genus *Induratia* is based on *Induratia apiospora*, a xylarialean pyrenomycete from New Zealand with clypeate uniperitheciate stromata, hyaline apiospores and a nodulisporium-like anamorph. However, because of the lack of DNA data from the generic type, its phylogenetic affinities have remained unresolved. Recently, two fungal species with teleomorphs strikingly similar to *Induratia* were discovered in Thailand*.* However, they did not produce an anamorph and were found to be phylogenetically close to the species classified within the hyphomycete genus *Muscodor*, which was described after *Induratia*. Therefore, in 2020 the species of *Muscodor* were transferred to *Induratia*, and a new family Induratiaceae was proposed.

**Results:**

We have encountered an unpublished ex-holotype strain of *Induratia apiospora* among the holdings of the ATCC collection, enabling detailed morphological and molecular phylogenetic investigations. We observed the characteristic nodulisporium-like anamorph described in the original publication. Phylogenetic analyses of multigene sequence data revealed a close relationship of *Induratia apiospora* to the Barrmaeliaceae, while a close relationship to the *Induratia* species formerly classified within *Muscodor* was rejected.

**Conclusions:**

We here classify *Induratia apiospora* within the Barrmaeliaceae and consider Induratiaceae to be synonymous with the former. As the holotype specimen of *Induratia apiospora* is apparently lost, an isotype specimen from WSP is selected as lectotype. We also propose that the genus *Muscodor* is resurrected within the Xylariaceae, and formally transfer several *Induratia* species to *Muscodor*.

**Supplementary Information:**

The online version contains supplementary material available at 10.1186/s40529-023-00372-1.

## Background

The taxonomy of the Sordariomycetes and other Ascomycota has changed drastically in the past decade, owing to the advent of multi-locus phylogenies, which were often combined in polyphasic studies, using morphological and chemotaxonomic data as additional evidence. The currently proposed classification of genera and higher taxa (cf. Hyde et al. 2020; Wijayawardene et al. 2022) is steadily changing as new evidence becomes available, in particular when the species that have been first described in the pre-molecular era are cultured and sequenced for the first time. A fair example are genera of the Xylariales, and especially so the Xylariaceae s. lat. where it has become evident due to the availability of molecular data that the classical discrimination of higher taxa based on ascus structure (uni-, bitunicate), fruiting bodies, anamorph-teleomorph connections and ascospore morphology alone is not feasible. This has been reflected by the recent re-organization of the families (Wendt et al. 2018), where the results of a four-locus genealogy were better in agreement with chemotaxonomy and anamorphic morphology than with the classical concept based on ascospore shape. Interestingly, the aforementioned phylogeny was even backed up at genus level by a concurrent phylogenomic study using the amino acid sequences of 4912 orthologue genes for the core genera of the Hypoxylaceae (Wibberg et al. 2021). Other families and genera of Xylariales, for which by far not that many data are available, are in bad need of further studies. It is to be expected that it will take several years more to generate sufficient amounts of data to attain a stable phylogeny. Numerous taxa are still only known from old morphological descriptions (often restricted to the teleomorphs), or have only recently been recollected and cultured to generate molecular data and study their anamorphs for the first time. These studies sometimes revealed rather unexpected phylogenetic affinities but also showed the limits of a morphocentric approach for phylogenetic assessments (Jaklitsch et al. 2014; 2016; Voglmayr et al. 2022).

Aside from attempts to re-discover fresh specimens corresponding to the old fungal taxa, it may at times also be feasible to screen the inventories of the large culture collections, since those may contain valuable reference or ex-type strains that have not been reported in the original literature. The current paper describes such a case.

The genus *Induratia* was originally described by Samuels et al. (1987) based on a single specimen from New Zealand that featured uniperitheciate stromata, asci with an amyloid ascal apparatus and apiosporous ascospores. The authors also observed a nodulisporium-like conidial stage in the mycelial culture they obtained, and this new combination had at that time given rise to the erection of a new monotypic genus. In the following decades the genus almost remained forgotten, except that Miller and Huhndorf (2005) included a specimen they referred to as “*Induratia* sp. SMH 1255” originating from Puerto Rico in their phylogenetic study of Sordariales and other Sordariomycetes. However, Miller and Huhndorf (2005) neither included any morphological data of the specimen (which is housed in the fungarium of the University of Illinois as ILLS 82598), nor cultured it nor studied the anamorph. Samarakoon et al. (2020) later included the DNA sequences derived from this collection and provided microscopic details of the stromata and ascogenic structures. These morphological features resembled the drawings of *Induratia apiospora* by Samuels et al. (1987)*,* even though the holotype specimen of this species has apparently been lost and could neither be located at the ZT nor the PDD herbarium. The molecular data of *Induratia* sp. SMH 1255 resembled those derived from two specimens that were freshly collected from Thailand. Moreover, they also clustered with the sequences of all hitherto described species of the genus *Muscodor*. This gave rise to the synonymisation of *Induratia* and *Muscodor*, with the former, older name taking priority over the younger *Muscodor*, and the erection of the new family Induratiaceae, which also included the similar genus *Emarcea* (Samarakoon et al. 2020).

However, we have recently encountered a culture labeled *Induratia apiospora* in the catalogue of the ATCC (Manassas, USA) via a random Google search on information on the genus on the Internet and decided to order and study it for comparison. The current paper is dedicated to the description of its characteristics and the necessary changes in the taxonomy of the Xylariales.

## Methods

### Morphological studies on strain ATCC 60639

A Google search for *Induratia apiospora* revealed a culture deposited as ATCC 60639 by G.J. Samuels not mentioned by Samuels et al. (1987). From discussions with two of the authors of the original paper, it was confirmed that this strain was indeed derived from the holotype specimen (O. Petrini and G.J. Samuels, personal communications). The strain *Induratia apiospora* (Samuels et al. 1987) was thus purchased from the American Type Culture Collection (ATCC, Mannassas, Virginia) under the accession ATCC 60639 and cultured on Yeast-Malt agar (10 g/L malt extract, 4 g/L D-glucose, 4 g/L yeast extract, supplemented with 20 g/L agar and adjusted to pH 6.3 prior to sterilization). The mycelia were transferred to a new plate once the strain had covered the medium by excision of a 5 mm^2^ square overgrown with mycelium on a regular basis (2–4 weeks). Plates were frequently monitored for sporulation.

For the morphological analysis of strain ATCC 60639, a small piece of sporulating mycelium was extracted and the dimensions of conidiogenous structures measured in distilled water and lactic acid. To observe the macro-morphology of the cultures, the strains were grown on Yeast-Malt agar (YM6.3; malt extract 10 g/L, yeast extract 4 g/L, D-glucose 4 g/L, agar 20 g/L, pH 6.3 before autoclaving), 2% Malt Extract Agar (MEA), Oatmeal Agar (OA, Sigma-Aldrich, Steinheim, Germany), and potato dextrose agar (PDA, Himedia, Mumbai, India) and the cultures checked at four weeks after inoculation. Photomicrographs were obtained using a DS-Fi3 camera connected to a Nikon eclipse Ni-U microscope (Nikon Europe BV, Amsterdam, Netherlands).

### DNA extraction, PCR amplification and sequencing

The DNA extraction protocol and the solutions used for PCR amplification followed the description of Wendt et al. (2018). PCR programs followed Samarakoon et al. (2020), with the exemption of using the primer pair ITS1f and ITS4 (White et al. 1990) instead of ITS5 and ITS4. Briefly, the following settings were used: ITS: 94 °C for 30 s, 56 °C for 50 s, 72 °C for 60 s; LSU: LR0R/LR5: 94 °C for 30 s, 55 °C for 50 s, 72 °C for 60 s (Vilgalys and Hester 1990); SSU: NS1/NS4: 94 °C for 30 s, 54 °C for 50 s, 72 °C for 60 s (White et al. 1990); *rpb2*: fRPB2-5F/fRPB2-7cR: 95 °C for 45 s, 57 °C for 50 s, 72 °C for 90 s (Liu et al. 1999); *tub2*: T1/T22: 95 °C for 60 s, 54 °C for 110 s, 72 °C for 120 s (O’Donnell and Cigelnik 1997). PCR amplicons were purified as described in Wendt et al. (2018) and sequences generated with the Sanger sequencing method at the Microsynth sequencing company, using respective forward and reverse primers (Microsynth SeqLab GmbH, Göttingen, Germany). Sequences were assembled as a consensus sequence from both reads by using the de-novo assembly program contained in Geneious® R7.1.9 (Kearse et al. 2012). The generated and further used sequences in this study are listed with their respective GenBank Acc. No. in Table 1.

**Table 1:** Feature table listing all sequences used for molecular phylogenetic inference. GenBank sequence accession numbers, type status of taxa, country of origin and specimen/strain numbers are given in the respective columns

Species	Specimen or strain number	Origin	Status	GenBank accession numbers				References
				ITS	LSU	*rpb2*	*tub2*	
*Albicollum longisporum*	CBS 147283	Spain	HT	ON869286	ON869286	ON808465	ON808509	Voglmayr et al. (2022)
*Albicollum vincensii*	CBS 147286	Austria	ET	ON869297	ON869297	ON808475	ON808519	Voglmayr et al. (2022)
*Amphirosellinia nigrospora*	HAST 91092308	Taiwan	HT	GU322457	N/A	GQ848340	GQ495951	Hsieh et al. (2010)
*Annulohypoxylon truncatum*	CBS 140778	Texas	ET	KY610419	KY610419	KY624277	KX376352	Kuhnert et al. (2017), Wendt et al. (2018)
*Anthostomelloides krabiensis*	MFLUCC 15–0678	Thailand	HT	KX305927	KX305928	KX305929	N/A	Tibpromma et al. (2017)
*Astrocystis concavispora*	MFLUCC 14–0174	Italy		KP297404	KP340545	KP340532	KP406615	Daranagama et al. (2015)
*Barrmaelia macrospora*	CBS 142768	Austria	ET	KC774566	KC774566	MF488995	MF489014	Voglmayr et al. (2018)
*Barrmaelia moravica*	CBS 142769	Austria	ET	MF488987	MF488987	MF488996	MF489015	Voglmayr et al. (2018)
*Barrmaelia oxyacanthae*	CBS 142770	Austria		MF488988	MF488988	MF488997	MF489016	Voglmayr et al. (2018)
*Barrmaelia rappazii*	CBS 142771	Norway	HT	MF488989	MF488989	MF488998	MF489017	Voglmayr et al. (2018)
*Barrmaelia rhamnicola*	CBS 142772	France	ET	MF488990	MF488990	MF488999	MF489018	Voglmayr et al. (2018)
*Biscogniauxia marginata*	MFLUCC 12–0740	France		KJ958407	KJ958408	KJ958409	KJ958406	Daranagama et al. (2015)
*Camillea obularia*	ATCC 28093	Puerto Rico		KY610384	KY610429	KY624238	KX271243	Wendt et al. (2018)
*Clypeosphaeria mamillana*	CBS 140735	France	ET	KT949897	KT949897	MF489001	MH704637	Jaklitsch et al. (2016), Voglmayr et al. (2018), Liu et al. (2019)
*Collodiscula japonica*	CBS 124266	China		JF440974	JF440974	KY624273	KY624316	Jaklitsch and Voglmayr (2011), Wendt et al. (2018)
*Creosphaeria sassafras*	STMA 14087	Argentina		KY610411	KY610468	KY624265	KX271258	Wendt et al. (2018)
*Daldinia concentrica*	CBS 113277	Germany		AY616683	KY610434	KY624243	KC977274	Triebel et al. (2005), Kuhnert et al. (2014), Wendt et al. (2018)
*Dematophora necatrix*	CBS 349.36	Argentina		AY909001	KF719204	KY624275	KY624310	Pelaez et al. (2008), Wendt et al. (2018)
*Diatrype disciformis*	CBS 197.49	Netherlands		N/A	DQ470964	DQ470915	N/A	Zhang et al. (2006)
*Digitodochium amoenum*	CBS 147285	Austria	ET	ON869303	ON869303	ON808481	ON808525	Voglmayr et al. (2022)
*Emarcea castanopsidicola*	CBS 117105	Thailand	HT	AY603496	MK762717	MK791285	MK776962	Duong et al. (2004), Samarakoon et al. (2020)
*Emarcea eucalyptigena*	CBS 139908	Malaysia	HT	KR476733	MK762718	MK791286	MK776963	Crous et al. (2015), Samarakoon et al. (2020)
*Entalbostroma erumpens*	ICMP 21152	New Zealand	HT	KX258206	N/A	KX258204	KX258205	Johnston et al. (2016)
*Entoleuca mammata*	100 J.D.R	France		GU300072	N/A	GQ844782	GQ470230	Hsieh et al. (2010)
*Entonaema liquescens*	ATCC 46302	USA		KY610389	KY610443	KY624253	KX271248	Wendt et al. (2018)
*Entosordaria perfidiosa*	CBS 142773	Austria	ET	MF488993	MF488993	MF489003	MF489021	Voglmayr et al. (2018)
*Entosordaria quercina*	CBS 142774	Greece	HT	MF488994	MF488994	MF489004	MF489022	Voglmayr et al. (2018)
*Eutypa lata*	UCR-EL1	USA		JGI	JGI	JGI	JGI	
*Graphostroma platystomum*	CBS 270.87	France		JX658535	DQ836906	KY624296	HG934108	Zhang et al. (2006), Stadler et al. (2014), Koukol et al. (2015), Wendt et al. (2018)
*Hypocreodendron sanguineum*	J.D.R. 169	Mexico		GU322433	N/A	GQ844819	GQ487710	Hsieh et al. (2010)
*Hypomontagnella monticulosa*	MUCL 54604	French Guiana	ET	KY610404	KY610487	KY624305	KX271273	Wendt et al. (2018)
*Hypoxylon fragiforme*	MUCL 51264	Germany	ET	KC477229	KM186295	KM186296	KX271282	Stadler et al. (2013), Daranagama et al. (2015), Wendt et al. (2018)
*Induratia apiospora*	ATCC 60639	New Zealand	HT	OP862879	OP862881	OP879469	OP879468	This study
*Jackrogersella multiformis*	CBS 119016	Germany	ET	KC477234	KY610473	KY624290	KX271262	Kuhnert et al. (2014), Kuhnert et al. (2017), Wendt et al. (2018)
*Kretzschmaria deusta*	CBS 163.93	Germany		KC477237	KY610458	KY624227	KX271251	Stadler et al. (2013), Wendt et al. (2018)
*Leptomassaria simplex*	CBS 147282	Austria	ET	ON869305	ON869305	ON808483	ON808527	Voglmayr et al. (2022)
*Linosporopsis ischnotheca*	CBS 145761	Switzerland	ET	MN818952	MN818952	MN820708	MN820715	Voglmayr and Beenken (2020)
*Linosporopsis ochracea*	CBS 145999	Germany	ET	MN818958	MN818958	MN820714	MN820721	Voglmayr et al. (2022)
*Lopadostoma turgidum*	CBS 133207	Austria	ET	KC774618	KC774618	KC774563	MF489024	Jaklitsch et al. (2014), Voglmayr et al. (2018)
*Muscodor albus*	9-6	N/A		HM034857	HM034865	N/A	HM034844	Zhang et al. (2010)
*Muscodor albus*	MONT 620		HT	AF324336	N/A	N/A	N/A	Worapong et al. (2001)
*Muscodor brasiliensis*	LGMF 1256		HT	KY924494	N/A	MF510171	N/A	Pena et al. (2019)
*Muscodor camphorae*	NFCCI 3236		HT	KC481681	N/A	N/A	N/A	Meshram et al. (2017)
*Muscodor cinnanomi*	BCC 38842		HT	GQ848369	N/A	N/A	N/A	Suwannarach et al. (2010)
*Muscodor coffeanum*	COAD 1842	Brazil	HT	KM514680	N/A	KP862881	N/A	Hongsanan et al. (2015)
*Muscodor crispans*	MONT 2347		HT	EU195297	N/A	N/A	N/A	Mitchell et al. (2008)
*Muscodor darjeelingensis*	NFCCI 3095		HT	JQ409997	N/A	N/A	N/A	Saxena et al. (2014)
*Muscodor equiseti*	JCM 18233		HT	JX089322	N/A	N/A	N/A	Suwannarach et al. (2013)
*Muscodor fengyangensis*	CGMCC 2862	China	HT	HM034856	HM034859	HM034849	HM034843	Zhang et al. (2010)
*Muscodor ghoomensis*	NFCCI 3234		HT	KF537625	N/A	N/A	N/A	Meshram et al. (2015)
*Muscodor indicus*	NFCCI 3235		HT	KF537626	N/A	N/A	N/A	Meshram et al. (2015)
*Muscodor kashayum*	NFCCI 2947		HT	KC481680	N/A	N/A	N/A	Meshram et al. (2013)
*Muscodor musae*	JCM 18230		HT	JX089323	N/A	N/A	N/A	Suwannarach et al. (2013)
*Muscodor oryzae*	JCM 18231		HT	JX089321	N/A	N/A	N/A	Suwannarach et al. (2013)
*Muscodor roseus*	MONT 2098		HT	AH010859	N/A	N/A	N/A	Worapong et al. (2002)
*Muscodor* sp.	SMH 1255			MN250031	AY780069	N/A	AY780119	Miller and Huhndorf (2005), Samarakoon et al. (2020)
*Muscodor strobelii*	NFCCI 2907		HT	JQ409999	N/A	N/A	N/A	Meshram et al. (2014)
*Muscodor suthepensis*	JCM 18232		HT	JN558830	N/A	N/A	N/A	Suwannarach et al. (2013)
*Muscodor suturae*	MSUB 2380		HT	JF938595	N/A	N/A	N/A	Kudalkar et al. (2012)
*Muscodor thailandica*	MFLUCC 17-2669	Thailand	HT	MK762707	MK762714	MK791283	MK776960	Samarakoon et al. (2020)
*Muscodor tigerensis*	NFCCI 3172		HT	JQ409998	N/A	N/A	N/A	Saxena et al. (2015)
*Muscodor vitigenus*	MONT P-15		HT	AY100022	N/A	N/A	N/A	Daisy et al. (2002)
*Muscodor yucatanensis*	MEXU 25511		HT	FJ917287	N/A	N/A	N/A	González et al. (2009)
*Muscodor yunnanensis*	CGMCC 3.18908	China	HT	MG866046	MG866038	MG866059	MG866066	Chen et al. (2019)
*Muscodor ziziphi*	MFLUCC 17-2662	Thailand	HT	MK762705	MK762712	MK791281	MK776958	Samarakoon et al. (2020)
*Magnostiolata mucida*	MFLU 19-2133	Thailand	HT	MW240673	MW240603	MW658652	MW775618	Samarakoon et al. (2020)
*Nemania ethancrensonii*	CBS 148337	USA	HT	ON869311	ON869311	ON808489	ON808533	Voglmayr et al. (2022)
*Nemania primolutea*	HAST 91102001	Taiwan	HT	EF026121	N/A	GQ844767	EF025607	Hsieh et al. (2010)
*Nemania uda*	CBS 148422	Austria		ON869312	ON869312	ON808488	ON808532	Voglmayr et al. (2022)
*Obolarina dryophila*	MUCL 49882	France		GQ428316	GQ428316	KY624284	GQ428322	Pažoutová et al. (2010), Wendt et al. (2018)
*Occultitheca rosae*	HKAS 102393	China	HT	MW240672	MW240602	MW658651	MW775617	Samarakoon et al. (2020)
*Oligostoma insidiosum*	CBS 147288	Switzerland	ET	ON869314	ON869314	ON808491	ON808535	Voglmayr et al. (2022)
*Podosordaria mexicana*	WSP 176	Mexico		GU324762	N/A	GQ853039	GQ844840	Hsieh et al. (2010)
*Poronia punctata*	CBS 656.78	Australia	HT	KT281904	KY610496	KY624278	KX271281	Senanayake et al. (2015), Wendt et al. (2018)
*Pyrenopolyporus hunteri*	MUCL 52673	Ivory Coast	ET	KY610421	KY610472	KY624309	KU159530	Kuhnert et al. (2017), Wendt et al. (2018)
*Rhopalostroma angolense*	CBS 126414	Ivory Coast		KY610420	KY610459	KY624228	KX271277	Wendt et al. (2018)
*Rosellinia corticium*	MUCL 51693	France		KY610393	KY610461	KY624229	KX271254	Wendt et al. (2018)
*Rostrohypoxylon terebratum*	CBS 119137	Thailand	HT	DQ631943	DQ840069	DQ631954	DQ840097	Tang et al. (2007), Fournier et al. (2011)
*Ruwenzoria pseudoannulata*	MUCL 51394	D. R. Congo	HT	KY610406	KY610494	KY624286	KX271278	Wendt et al. (2018)
*Sarcoxylon compunctum*	CBS 359.61	South Africa		KT281903	KY610462	KY624230	KX271255	Senanayake et al. (2015), Wendt et al. (2018)
*Spiririma gaudefroyi*	CBS 147284	Spain	ET	ON869320	ON869320	ON808497	ON808541	Voglmayr et al. (2022)
*Stilbohypoxylon elaeicola*	Y.M.J. 173	French Guiana		EF026148	N/A	GQ844826	EF025616	Hsieh et al. (2010)
*Stromatoneurospora phoenix*	BCC 82040	Thailand		MT735133	MT735133	MT742605	MT700438	Becker et al. (2020)
*Thamnomyces dendroideeus*	CBS 123578	French Guiana		FN428831	KY610467	KY624232	KY624313	Stadler et al. (2010), Wendt et al. (2018)
*Xylaria apoda*	HAST 90080804	Taiwan		GU322437	N/A	GQ844823	GQ495930	Hsieh et al. (2010)
*Xylaria arbuscula*	CBS 126415	Germany		KY610394	KY610463	KY624287	KX271257	Fournier et al. (2011), Wendt et al. (2018)
*Xylaria atrosphaerica*	HAST 91111214	Taiwan		GU322459	N/A	GQ848342	GQ495953	Hsieh et al. (2010)
*Xylaria digitata*	HAST 919	Ukraine		GU322456	N/A	GQ848338	GQ495949	Hsieh et al. (2010)
*Xylaria hypoxylon*	CBS 122620	Sweden	ET	KY610407	KY610495	KY624231	KX271279	Sir et al. (2016b), Wendt et al. (2018)
*Xylaria ianthinovelutina*	HAST 553	French West Indies		GU322441	N/A	GQ844828	GQ495934	Hsieh et al. (2010)
*Xylaria laevis*	HAST 419	French West Indies		GU324746	N/A	GQ848359	GQ502695	Hsieh et al. (2010)
*Xylaria longipes*	CBS 148.73	Germany		MH860649	MH872351	KU684280	KU684204	Vu et al. (2019), U'Ren et al. (2016)
*Xylaria oxyacanthae*	J.D.R. 859	USA		GU322434	N/A	GQ844820	GQ495927	Hsieh et al. (2010)
*Xylaria polymorpha*	MUCL 49884	France		KY610408	KY610464	KY624288	KX271280	Wendt et al. (2018)

### Taxon selection and molecular phylogenetic inference

To assess the affinities of the newly generated sequences within the order Xylariales, the manually curated alignment and taxon set recently presented by Voglmayr et al. 2022 was used, further restricting sequences derived from Xylariaceae from 154 to 94 taxa. The newly generated sequences of the ITS, LSU, *rpb2* and *tub2* loci were inserted into the data matrix and manually checked for consistency. The different loci were subjected to IQTree2 (Minh et al. 2020) for molecular phylogenetic inference using Maximum-Likelihood criterion with options for a partitioned analysis (Chernomor et al. 2016) of the supermatrix with prior testing for the optimal nucleotide substitution model by using ModelFinder (Kalyaanamoorthy et al. 2017) following BIC criterion and 1000 non-parametric bootstrap (Felsenstein 1985) replicates. Concurrently, the optimal nucleotide substitution models for each locus were calculated with PartitionFinder2 (Lanfear et al. 2016) as implemented in the PhyloSuite V.1.2.2 (Zhang et al. 2020) program package and to-be-tested models restricted to the ones available in MrBayes 3.2.7a (Ronquist et al. 2012). Partitions were regarded as unlinked and testing options set to BIC optimality criterion and test strategy set to test all. A phylogenetic inference using MrBayes 3.2.7a followed, with settings used and described by Kemkuignou et al. (2022). Briefly, a random starting tree was used to calculate 200.000.000 generations with convergence controlled to arrive at an average split frequency of 0.01. Tree sampling was done every 1000 generations, of which the first 25% were discarded as “burn-in”. Four incrementally heated chains were used for the Markov Chain Monte Carlo (MCMC), with temperature set to 0.15. The BEAGLE library (Ayres et al. 2012) and a parallel Metropolis coupling for the MCMC (Altekar et al. 2004) were used to calculate in total four chains in parallel. The resulting bootstrap (bs) ≥ 70% and posterior probabilities ≥ 0.95 were mapped on the respective bipartition on the found maximum-likelihood tree.

## Results

### Morphological studies on strain ATCC 60639

**Fig. 1:**
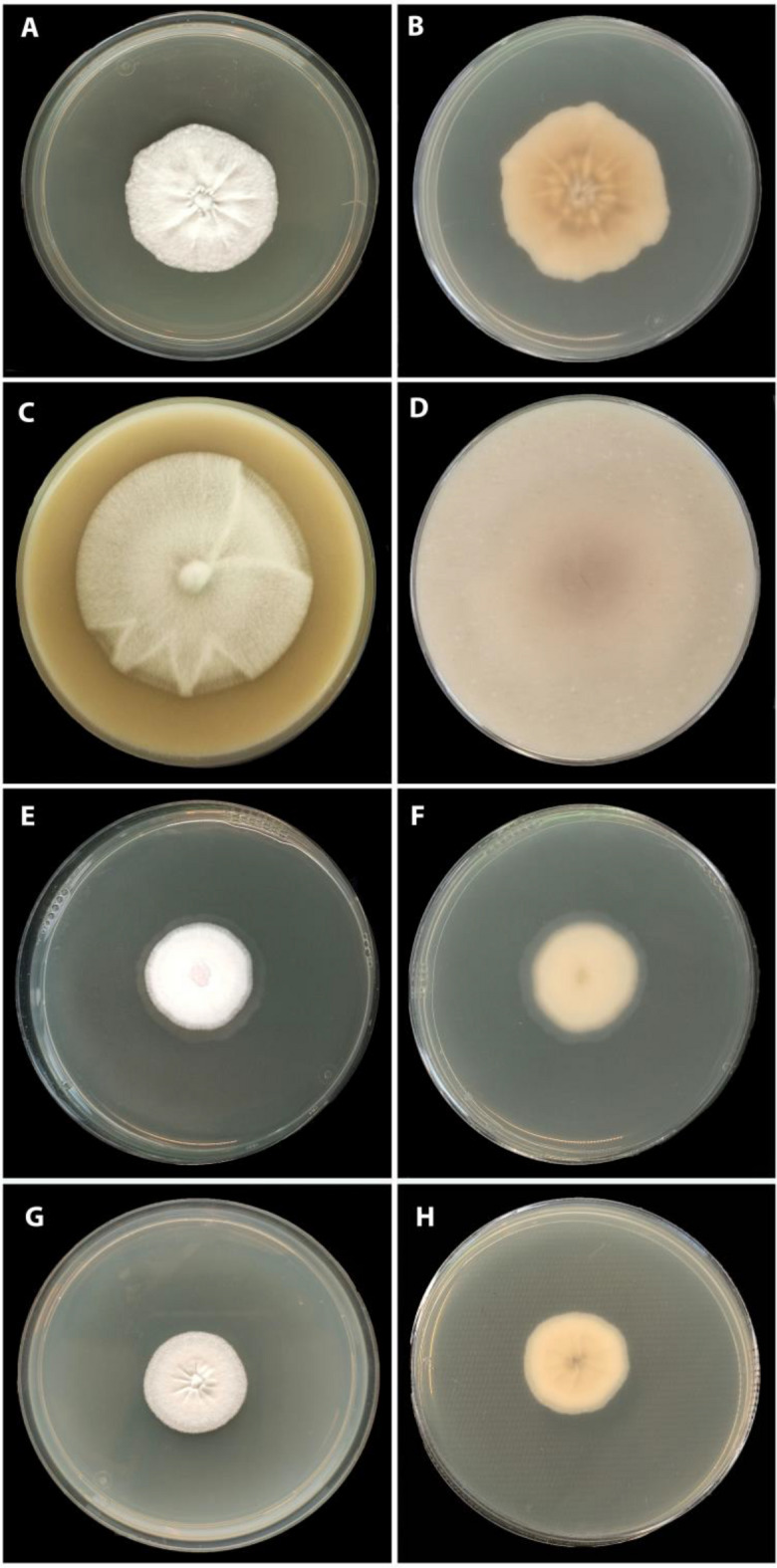
Colonies of *Induratia apiospora* (ATCC 60639) after four weeks **A**, **B** on MEA; **C**, **D** on OA; **E**, **F** on PDA; **G**, **H** on YM6.3

The culture we obtained from ATCC showed the following characteristics (Fig. 1):

**Culture characteristics**: On YM6.3 and PDA: 2.6 cm in four weeks. Mycelium white, wavy or cottony hyphal growth, margin filiform to slightly undulate, flat to slightly elevate. Reverse white to pale. On MEA: 4.2 cm in four weeks, differs by having a mycelium with a margin slightly undulate, flat. On OA: 5.8 cm in four weeks, differing to have a margin entire. The information is summarized in Table 2. **Sporulating regions:** in patches, citrine (13) olivaceous (48). Conidiogenous structure nodulisporium-like, abundant, smooth to slightly roughened. Conidiogenous cells: melanized, smooth to slightly roughened, 34.5–66.5 × 2–3 µm (n = 23). Conidia: hyaline with cytoplasm content melanized, smooth, ellipsoidal to obovoid, 4–6 × 2–4 µm (n = 41).

**Table 2:** Rates of growth and culture characteristics of *Induratia apiospora* ATCC60639 in four different media at four weeks of incubation

Media	area (cm^2^)	diameter (cm)	Temperature	Margin	Elevation	Mycelium color
YM6.3	5.5	2.6	25 °C	filiform to slightly undulate	slightly elevate	White
MEA	14.0	4.2		slightly undulate	flat	White
OA	26.5	5.8		Entire	slightly elevate	White
PDA	5.4	2.6		filiform to slightly undulate	slightly elevate	White

**Fig. 2:**
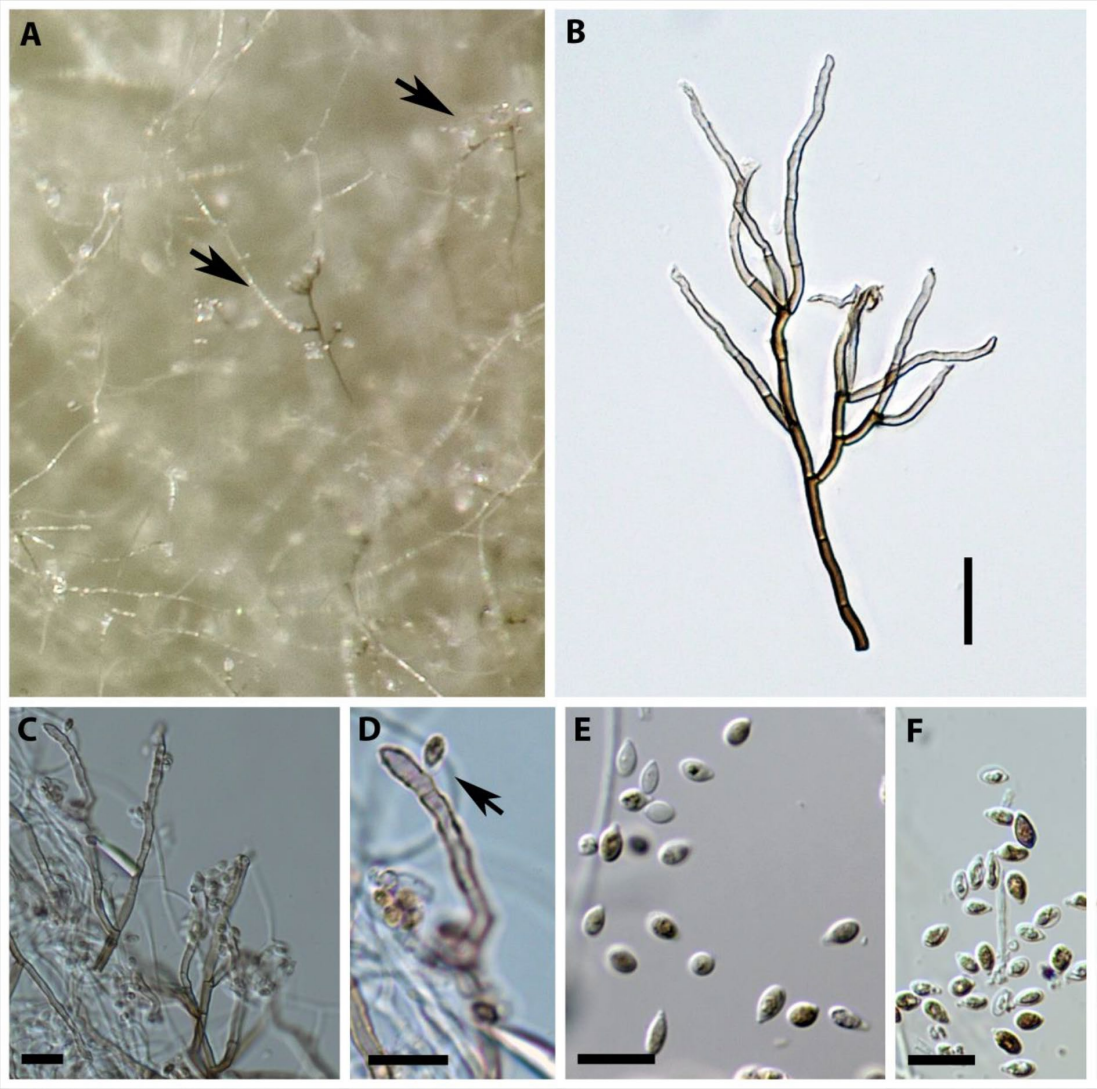
Morphology of the anamorph of the ex-type strain of *Induratia apiospora* (ATCC 60639) on YM6.3. **A** Conidiophore on surface mycelium. **B** Single nodulisporium-like conidiophore. **C**, **D** Conidiogenous cells. **E**, **F** Conidia. Scale bars: **B** 20 µm, **E**, **F** 10 µm

These characteristics (see also Fig. 2) are indeed well in accordance with what Samuels et al. (1987) had reported for *Induratia apiospora*. According to the description by Samuels et al. (1987), the anamorph is nodulisporium-like, conidiogenous cells are light brown, slightly roughened, conidia are hyaline, ellipsoidal to obovoid, 4–6 × 2–4 µm.

### Molecular phylogenetic inference

**Fig. 3:**
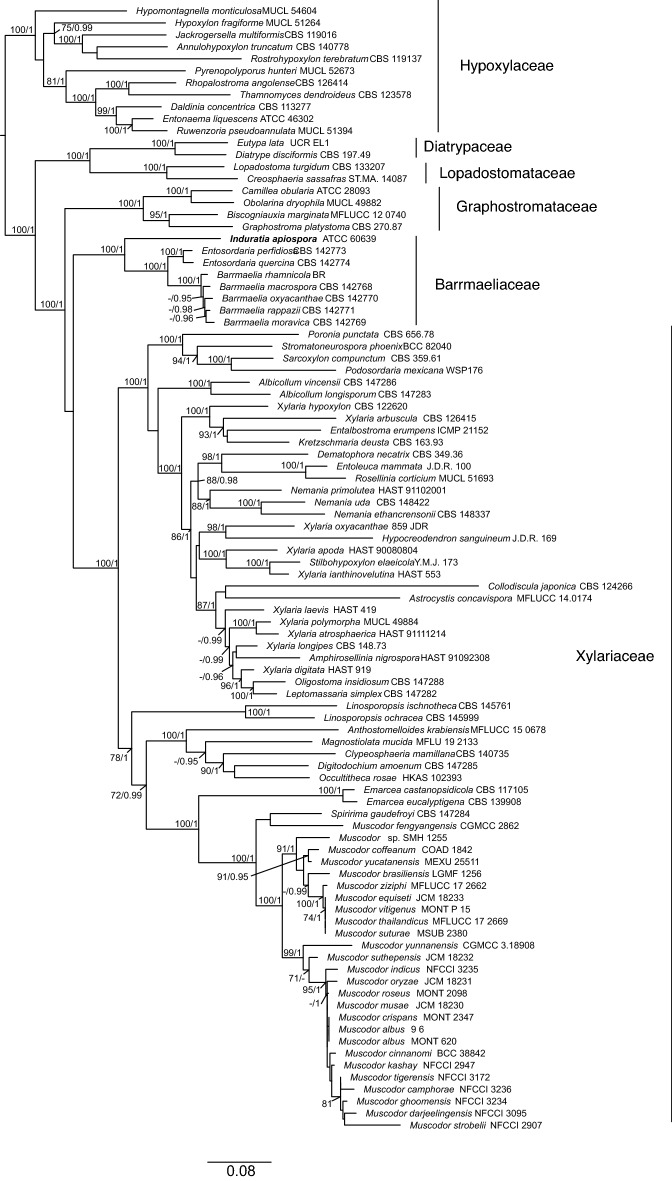
Maximum Likelihood Tree (lLn = − 89939.9947) inferred from a manually edited alignment of ITS, LSU, *rpb2* and *tub2* sequences featuring sequences derived from Hypoxylaceae, Diatrypaceae, Lopadostomataceae, Graphostromataceae, Barrmaeliaceae and Xylariaceae. The position of the newly generated and concatenated sequences of *Induratia apiospora* are marked in bold. Bootstrap and Bayesian posterior probabilities ≥ 70% and ≥ 0.95, respectively, are given at bipartitions

The alignment subjected to IQTree and MrBayes consisted of 4679 sites in total, distributed among loci corresponding to the ITS (584 sites), LSU (1329 sites), *rpb2* (1235 sites) and *tub2* (1531 sites). The generated sequence of the SSU for *Induratia* *apiospora* ATCC 60639 has not been used for phylogenetic reconstruction, but has been deposited under the GenBank Acc. No. OQ748100 for the scientific community to use. A detailed list of alignment features and the tested models per partition, as well as the alignments can be found in the (Additional file 1: Tables S1–S3). The final inferred tree following maximum-likelihood analysis had an lLn of − 89939.9947 (Fig. 3). The topology of trees generated from the ML and Bayesian approach were identical, showing a similar arrangement of the taxa as previously presented by Voglmayr et al. (2022). Briefly, the Hypoxylaceae, Diatrypaceae, Lopadostomataceae, Graphostromataceae, Xylariaceae and Barrmaeliaceae received maximum support. The position of the latter was not resolved. Sequences derived from *Induratia* sensu Samarakoon et al. (2020; given as *Muscodor* in Fig. 3), *Spiririma gaudefroyi* and *Emarcea* received maximum support in a clade nested inside the Xylariaceae. Surprisingly, the sequences of *Induratia apiospora* ATCC 60639 clustered in a basal position to a clade formed by *Entosordaria* and *Barrmaelia* representing the Barrmaeliaceae, each clade receiving maximum support.

### Taxonomy

Our phylogenetic study showed that the ex-type strain of *Induratia* resolves within the Barrmaeliaceae. The Induratiaceae are thus regarded as a synonym of Barrmaeliaceae and *Muscodor* is resurrected. The holotype specimen of *Induratia* could still not be recovered, hence the isotype (WSP73242; MyCoPortal 2022) located at Shaw Mycology Herbarium (WSP; Washington State University) is chosen as lectotype. The genera *Emarcea* and *Muscodor*, which were previously classified within Induratiaceae, are now formally accommodated in the Xylariaceae.

**Barrmaeliaceae** Voglmayr & Jaklitsch, Mycol. Progr. 17(1–2): 162 (2017) [2018], emend. Cedeño-Sanchez, M. Stadler, Voglmayr & C. Lambert.

MycoBank MB 822042

Type genus: *Barrmaelia* Rappaz

≡ Induratiaceae Samarak., Thongbai, K.D. Hyde & M. Stadler, Fungal Diversity 101: 188 (2020) [MB833443], syn. nov.

Other genera in the family: *Entosordaria* Höhn. (see Voglmayr et al. 2018), *Induratia* Samuels, E. Müll. & Petrini (see below)*.*

Saprobic on wood or bark. Stroma if present mostly in wood and blackening the surface in wide areas or in elongate bands, sometimes darker or carbonized around the ostioles; entostroma prosenchymatous, poorly developed, sometimes delimited by a black carbonized line (*Induratia*), without KOH-extractable pigments; ectostroma variable, from virtually absent, poorly developed to strongly carbonized and clypeus-like. Ascomata (perithecia) globose, sometimes raising the substrate, singly, in small groups or gregarious. Peridium melanized, pseudoparenchymatous to prosenchymatous. Hamathecium of numerous persistent, hyaline, septate paraphyses. Asci eight-spored, cylindrical, persistent, with inamyloid or amyloid apical ascus apparatus. Ascospores hyaline, yellow to dark brown; unicellular with or without germ slit (*Barrmaelia*), or two-celled with septum near one end, the small cell hyaline, the large cell dark brown and with an apical germ apparatus consisting of radial slits (*Entosordaria*), or hyaline, two-celled, apiosporous without germ slit (*Induratia*); allantoid, ellipsoid or fusoid, inequilateral, slightly inequilateral or nearly equilateral, with narrowly or broadly rounded ends. Anamorph, where known, libertella-like (*Barrmaelia*; Rappaz 1995), or nodulisporium-like (*Induratia*).

### Key to the genera of Barrmaeliaceae

1. Ascospores one-celled, asymmetrically ellipsoid to allantoid, uniformly light to dark brown, with or without a longitudinal germ slit, without appendages; anamorph libertella-like……………….……………*Barrmaelia.*

1. Ascospores two-celled, apiosporous, germ locus absent or consisting of apical radial slits, with or without appendages…………………………………2

2. Ascospores with submedian septum, entirely hyaline, without germ locus, with hyaline cellular appendages at each end while still in the ascus; anamorph nodulisporium-like………………………………………*Induratia*.

2. Ascospores with septum near one end, small ascospore cell hyaline, large ascospore cell dark brown and with an apical germ apparatus consisting of radial slits, without appendages; anamorph unknown………………………………………………*Entosordaria.*

***Induratia*** Samuels, E. Müll. & Petrini, Mycotaxon 28(2): 484 (1987).

**Type species**: ***Induratia apiospora*** Samuels, E. Müll. & Petrini, Mycotaxon 28(2): 484 and Fig. 5 (1987).

MycoBank MB 130900

**Holotype**: New Zealand, North Island, Hokianga Co., Waipoua State Forest, near Yakas Track, on decorticated wood, 30 May 1982, G.J. Samuels and P. Johnston (PDD 44399, lost). **Lectotype:** (designated here, MBT 10010382) New Zealand North Island, Hokianga Co., Waipoua State Forest, near Yakas Track, on decorticated wood, 30 May 1982, G.J. Samuels and P. Johnston (WSP73242).

**Ex-type culture:** ATCC 60639, deposited by G.J. Samuels; duplicates sent to CBS under accession CBS 149733 and ICMP 24754.

### Resurrection of Muscodor

The genus *Muscodor* is now placed in the Xylariaceae, where it had originally been accommodated based on phylogenetic relationship, however anamorph morphology, a traditionally important character for the characterization of Xylariales, is still lacking. Below we list all species that were formerly accepted in *Muscodor*, including those that have been invalidly described in the original publications. The typifications have already been corrected and changed by Samarakoon et al. (2020) according to the rules of the International Code of Nomenclature for Algae, Fungi, and Plants (ICN), when those designations were validated in *Induratia*. We here introduce new combinations for these *Induratia* names in *Muscodor*.

***Muscodor*** Worapong, Strobel & W.M. Hess, Mycotaxon 79: 71 (2001).

**Type species: *Muscodor albus*** Worapong, Strobel & W.M. Hess, Mycotaxon 79: 71 (2001).

≡ *Induratia alba* (Worapong, Strobel & W.M. Hess) Samarak., Thongbai, K.D. Hyde & M. Stadler, Fungal Divers. 101(1): 193 (2020).

### Other accepted species

***Muscodor brasiliensis*** (L.C. Pena, Servienski & V. Kava ex Samarak., Thongbai, K.D. Hyde & M. Stadler) Cedeño-Sanchez, M. Stadler, Voglmayr & C. Lambert, ***comb. nov*.**

MycoBank MB 846432

Basionym: *Induratia brasiliensis* L.C. Pena, Servienski & V. Kava ex Samarak., Thongbai, K.D. Hyde & M. Stadler, Fungal Divers. 101(1): 196 (2020).

[originally described as: *Muscodor brasiliensis* L.C. Pena, Servienski & V. Kava, Microbiol. Res. 221: 32 (2019), nom. inval., Art. 40.7 (Shenzhen)]

***Muscodor camphorae*** Meshram, N. Kapoor, G. Chopra & S. Saxena [as ‘*camphora*’], Mycosphere 8(4): 571 (2017).

≡ *Induratia camphorae* (Meshram, N. Kapoor, G. Chopra & S. Saxena) Samarak., Thongbai, K.D. Hyde & M. Stadler, Fungal Divers. 101(1): 193 (2020).

***Muscodor cinnamomi*** Suwannar., Bussaban, K.D. Hyde & Lumyong, Mycotaxon 114: 19 (2011) [2010].

≡ *Induratia cinnamomi* (Suwannar., Bussaban, K.D. Hyde & Lumyong) Samarak., Thongbai, K.D. Hyde & M. Stadler, Fungal Divers. 101(1): 193 (2020).

***Muscodor coffeanus*** A.A.M. Gomes, Pinho & O.L. Pereira [as ‘*coffeanum*’], in Hongsanan et al., Cryptog. Mycol. 36(3): 368 (2015).

≡ *Induratia coffeana* (A.A.M. Gomes, Pinho & O.L. Pereira) Samarak., Thongbai, K.D. Hyde & M. Stadler, Fungal Divers. 101(1): 193 (2020).

***Muscodor crispans*** A.M. Mitch., Strobel, W.M. Hess, Pérez-Vargas & Ezra, Fungal Divers. 31: 41 (2008).

≡ *Induratia crispans* (A.M. Mitch., Strobel, W.M. Hess, Pérez-Vargas & Ezra) Samarak., Thongbai, K.D. Hyde & M. Stadler, Fungal Divers. 101(1): 193 (2020).

***Muscodor darjeelingensis*** (Meshram, N. Kapoor & S. Saxena ex Samarak., Thongbai, K.D. Hyde & M. Stadler) Cedeño-Sanchez, M. Stadler, Voglmayr & C. Lambert, ***comb. nov.***

MycoBank MB 846443

Basionym: *Induratia darjeelingensis* Meshram, N. Kapoor & S. Saxena ex Samarak., Thongbai, K.D. Hyde & M. Stadler, Fungal Divers. 101(1): 198 (2020).

[originally described as: *Muscodor darjeelingensis* Meshram, N. Kapoor & S. Saxena, Sydowia 66(1): 61 (2014), nom. inval., Art. 40.7 (Shenzhen)]

***Muscodor equiseti*** Suwannar. & Lumyong, in Suwannarach, Kumla, Bussaban, Hyde, Matsui & Lumyong, Ann. Microbiol. 63(4): 1350 (2013).

≡ *Induratia equiseti* (Suwannar. & Lumyong) Samarak., Thongbai, K.D. Hyde & M. Stadler, Fungal Divers. 101(1): 193 (2020).

***Muscodor fengyangensis*** (Chu L. Zhang ex Samarak., Thongbai, K.D. Hyde & M. Stadler) Cedeño-Sanchez, M. Stadler, Voglmayr & C. Lambert, ***comb. nov.***

MycoBank MB 846442

Basionym: *Induratia fengyangensis* Chu L. Zhang ex Samarak., Thongbai, K.D. Hyde & M. Stadler, Fungal Divers. 101(1): 198 (2020).

[originally described as: *Muscodor fengyangensis* Chu L. Zhang, Fungal Biol. 114(10): 801 (2010), nom. inval., Art. 40.1 (Shenzhen)]

***Muscodor ghoomensis*** S. Saxena, M. Gupta & Meshram, Sydowia 67: 136 (2015).

≡ *Induratia ghoomensis* (S. Saxena, M. Gupta & Meshram) Samarak., Thongbai, K.D. Hyde & M. Stadler, Fungal Divers. 101(1): 193 (2020).

***Muscodor heveae*** (Siri-Udom & Lumyong ex Samarak., Thongbai, K.D. Hyde & M. Stadler) Cedeño-Sanchez, M. Stadler, Voglmayr & C. Lambert, ***comb. nov.***

MycoBank MB 846436

Basionym: *Induratia heveae* Siri-Udom & Lumyong ex Samarak., Thongbai, K.D. Hyde & M. Stadler, Fungal Divers. 101(1): 199 (2020).

[originally described as: *Muscodor heveae* Siri-Udom & Lumyong, Ann. Microbiol. 66(1): 442 (2015), nom. inval., Art. 40.7 (Shenzhen)]

***Muscodor indicus*** S. Saxena, M. Gupta & Meshram [as ‘*indica*’], Sydowia 67: 136 (2015).

≡ *Induratia indica* (S. Saxena, M. Gupta & Meshram) Samarak., Thongbai, K.D. Hyde & M. Stadler, Fungal Divers. 101(1): 193 (2020).

***Muscodor kashay*** (Meshram, N. Kapoor & S. Saxena ex Samarak., Thongbai, K.D. Hyde & M. Stadler) Cedeño-Sanchez, M. Stadler, Voglmayr & C. Lambert, ***comb. nov.***

MycoBank MB 846437

Basionym: *Induratia kashay* Meshram, N. Kapoor & S. Saxena ex Samarak., Thongbai, K.D. Hyde & M. Stadler, Fungal Divers. 101(1): 199 (2020).

[originally described as: *Muscodor kashayum* Meshram, N. Kapoor & S. Saxena, Mycology 4(4): 198 (2013), nom. inval., Art. 40.7 (Shenzhen)]

***Muscodor musae*** Suwannar. & Lumyong, in Suwannarach, Kumla, Bussaban, Hyde, Matsui & Lumyong, Ann. Microbiol. 63(4): 1347 (2013).

≡ *Induratia musae* (Suwannar. & Lumyong) Samarak., Thongbai, K.D. Hyde & M. Stadler, Fungal Divers. 101(1): 193 (2020).

***Muscodor oryzae*** Suwannar. & Lumyong, in Suwannarach, Kumla, Bussaban, Hyde, Matsui & Lumyong, Ann. Microbiol. 63(4): 1349 (2013).

≡ *Induratia oryzae* (Suwannar. & Lumyong) Samarak., Thongbai, K.D. Hyde & M. Stadler, Fungal Divers. 101(1): 194 (2020).

***Muscodor roseus*** Worapong, Strobel & W.M. Hess, Mycotaxon 81: 467 (2002).

≡ *Induratia rosea* (Worapong, Strobel & W.M. Hess) Samarak., Thongbai, K.D. Hyde & M. Stadler, Fungal Divers. 101(1): 194 (2020).

***Muscodor strobelii*** Meshram, S. Saxena & N. Kapoor, Mycotaxon 128: 96 (2014).

≡ *Induratia strobelii ***(**Meshram, S. Saxena & N. Kapoor) Samarak., Thongbai, K.D. Hyde & M. Stadler, Fungal Divers. 101(1): 194 (2020).

***Muscodor suthepensis*** Suwannar. & Lumyong, in Suwannarach, Kumla, Bussaban, Hyde, Matsui & Lumyong, Ann. Microbiol. 63(4): 1349 (2013).

≡ *Induratia suthepensis* (Suwannar. & Lumyong) Samarak., Thongbai, K.D. Hyde & M. Stadler, Fungal Divers. 101(1): 194 (2020).

***Muscodor suturae*** Kudalkar, Strobel & Riy.-Ul-Hass. [as ‘*sutura*’], Mycoscience 53(4): 322 (2012).

≡ *Induratia suturae* (Kudalkar, Strobel & Riy.-Ul-Hass.) Samarak., Thongbai, K.D. Hyde & M. Stadler, Fungal Divers. 101(1): 194 (2020).

***Muscodor thailandicus*** (Samarak., Thongbai, K.D. Hyde & M. Stadler) Cedeño-Sanchez, M. Stadler, Voglmayr & C. Lambert, ***comb. nov.***

MycoBank MB 846434

Basionym: *Induratia thailandica* Samarak., Thongbai, K.D. Hyde & M. Stadler, Fungal Divers. 101(1): 194 (2020).

***Muscodor tigerensis*** (S. Saxena, Meshram & N. Kapoor ex Samarak., Thongbai, K.D. Hyde & M. Stadler) Cedeño-Sanchez, M. Stadler, Voglmayr & C. Lambert, ***comb. nov.***

MycoBank MB 846438

Basionym: *Induratia tigerensis* S. Saxena, Meshram & N. Kapoor ex Samarak., Thongbai, K.D. Hyde & M. Stadler Fungal Divers. 101(1): 199 (2020).

[originally described as: *Muscodor tigerensis* S. Saxena, Meshram & N. Kapoor, Ann. Microbiol. 65(1): 53 (2014) [2015], [as ‘*tigerii*’], nom. inval., Art. 40.7 (Shenzhen)].

***Muscodor vitigenus*** Daisy, Strobel, Ezra & W.M. Hess, in Daisy, Strobel, Ezra, Castillo, Baird & Hess, Mycotaxon 84: 45 (2002).

≡ *Induratia vitigena* (Daisy, Strobel, Ezra & W.M. Hess,) Samarak., Thongbai, K.D. Hyde & M. Stadler, Fungal Divers. 101(1): 196 (2020).

***Muscodor yucatanensis*** M.C. González, A.L. Anaya, Glenn & Hanlin, Mycotaxon 110: 365 (2009).

≡ *Induratia yucatanensis* (M.C. González, A.L. Anaya, Glenn & Hanlin) Samarak., Thongbai, K.D. Hyde & M. Stadler, Fungal Divers. 101(1): 196 (2020).

***Muscodor yunnanensis*** (C.L. Zhang ex Samarak., Thongbai, K.D. Hyde & M. Stadler) Cedeño-Sanchez, M. Stadler, Voglmayr & C. Lambert, ***comb. nov.***

MycoBank MB 846435

Basionym: *Induratia yunnanensis* C.L. Zhang ex Samarak., Thongbai, K.D. Hyde & M. Stadler, Fungal Divers. 101(1): 199 (2020).

[originally described as: *Muscodor yunnanensis* C.L. Zhang, in Chen et al., Mycosphere 10(1): 193 (2019), nom. inval., Art. 40.8 (Shenzhen)]

***Muscodor ziziphi*** (Samarak., Thongbai, K.D. Hyde & M. Stadler) Cedeño-Sanchez, M. Stadler, Voglmayr & C. Lambert, ***comb. nov.***

MycoBank MB 846433

Basionym: *Induratia ziziphi* Samarak., Thongbai, K.D. Hyde & M. Stadler, Fungal Divers. 101(1): 196 (2020).

### Notes

The specimen SMH 1255 from Puerto Rico, originally studied by Miller and Huhndorf (2005) and later by Samarakoon et al. (2020), has so far been treated as a member of the genus *Induratia.* It should henceforth be treated as a species of *Muscodor*, as its phylogenetic affinities are with the latter genus. Even though the species has not been formally described, the taxonomic name associated with the GenBank Acc. Nos for its DNA sequences ought to be changed.

***Emarcea*** Duong, Jeewon & K.D. Hyde, Stud. Mycol. 50(1): 255 (2004).

MycoBank MB 500070.

**Type species: *Emarcea castanopsidicola*** Duong, Jeewon & K.D. Hyde, Stud. Mycol. 50(1): 255 (2004).

### Notes

Since the genus *Emarcea* is phylogenetically closely related to the species in the *Muscodor* clade, it is again classified within the Xylariaceae, together with the *Muscodor* species discussed above.

## Discussion

Multi-locus molecular phylogenetic analysis proved to serve as a powerful tool to emend placements of taxonomic groups in the Xylariales over the last years, exemplified by the erection of the Barrmaeliaceae to accommodate a phylogenetically distinct and well-supported clade of *Barrmaelia* and *Entosordaria* (Voglmayr et al. 2018). Other examples include the emendation of the Hypoxylaceae in concordance with chemotaxonomic information (Wendt et al. 2018) or the resurrection of *Dematophora*, distinguished from *Rosellinia* species by its synnematal geniculosporium-like anamorph and often a phytopathogenic lifestyle (Wittstein et al. 2020). Regarding the recent synonymisation of *Muscodor* with *Induratia* (Samarakoon et al. 2020), some doubts remained on the validity of that approach, e.g., because the anamorph described by Samuels et al. (1987) was hitherto not observed in the *Muscodor* cultures, which seem to be unable to sporulate. The proposed taxonomy of Samarakoon et al. (2020) was based on the rather similar teleomorphs of *M. thailandicus* (≡ *I. thailandica*) and *M. ziziphi* (≡ *I. ziziphi*) and the *Muscodor* sp. (≡ *Induratia* sp.) specimen reported by Miller and Huhndorf (2005) and characterized by Samarakoon et al. (2020), featuring typical xylariaceous asci and apiospores. The results of the corresponding phylogenies unfortunately did not include any sequences of *Induratia* before erection of its eponymous family, due to a lack of access to molecular data to compare (Samarakoon et al. 2020). Only two years later, Voglmayr et al. (2022) found that DNA sequences of *Spiririma* (≡ *Helicogermslita*) *gaudefroyi*, clustered within the Induratiaceae clade. Since *S. gaudefroyi* features dark ascospores, this gave reason to challenge the concept of restricting apiosporous Xylariales to the Induratiaceae. In our study, the ex-holotype culture of *Induratia apiospora* isolated by Samuels et al. (1987) clustered in a basal position with *Barrmaelia* and *Entosordaria*, while the position of the Barrmaeliaceae remained unchanged. As the *Induratia* spp. transferred from *Muscodor* showed no relationship with the ex-holotype culture of *I. apiospora,* the genus *Muscodor* is resurrected. Furthermore, this implied that the Induratiaceae cannot be retained as an own family, from a phylogenetic perspective, but should be merged with Barrmaeliaceae (Voglmayr et al. 2022). Morphological characters formerly used to segregate the Induratiaceae from other members of the Xylariaceae, i.e. the presence of apiosporous ascospores, can apparently not serve beyond species discrimination. The resurrected *Muscodor* still showed a paraphyletic structure, indicated by a clade formed by *Spiririma* and *Muscodor fengyangensis*, which may result in further re-arrangements in the future, e.g., when a larger number of anamorph/teleomorph relationships has been established.

Serious degrees of synapomorphies are well documented within the Xylariaceae, illustrated by the paraphyly of the genus *Xylaria*, and so far it has not been possible to resolve this problem even by extensive taxon sampling (Hsieh et al. 2010; Voglmayr et al. 2018, 2022; Voglmayr and Beenken 2020). A similar situation was previously encountered for another family in the Xylariales, the Hypoxylaceae. An important feature of the Hypoxylaceae is the prolific secondary metabolism of its members, which has long been exploited for chemotaxonomic purposes. Recently, it was possible to match chemotaxonomic data by performing genome mining for secondary metabolite-encoding biosynthesis gene clusters (Wibberg et al. 2021; Kuhnert et al. 2021) after sequencing of the full genomes of some representative strains using 3^rd^ generation techniques with phylogenomic approaches. This example indicates the feasibility to sample for additional phenotypic data, i.e. data about the secondary metabolism, and attempting to link it with phylogenetic data on a larger scale to explore taxonomic affinities. Almost all Xylariales are well-known to be very prolific secondary metabolite producers, and in particular *Muscodor* species are known to produce volatile antibiotics. Therefore, following the previously presented example of the Hypoxylaceae, we suggest the inclusion of chemotaxonomic data and in particular studies on the non-volatile metabolites, combined with robust synthetic chemistry enabling the identification of the produced metabolites for the future.

## Supplementary Information

**Additional file 1.** Images of the isotype located in the Shaw Mycological Herbarium (WSP), provided by Monique H. Slipher, are shown in Fig. S1. Auxilliary information and characteristics covering the molecular phylogenetic analysis as well as the alignment are given in the Tables S1–S3.

## Data Availability

All data except for the DNA sequences, which are deposited in GenBank (https://www.ncbi.nlm.nih.gov/genbank/) are available in the manuscript or the Additional file 1.
