# Supplementary material for: Redisposition of apiosporous genera Induratia and Muscodor in the Xylariales, following the discovery of an authentic strain of Induratia apiospora
Source: Bot Stud. 2023 Apr 13;64:8. doi: 10.1186/s40529-023-00372-1 (PMC10102272; doi:10.1186/s40529-023-00372-1)
Supplement: Supplementary file 1 — Additional file 1. Images of the isotype located in the Shaw Mycological Herbarium (WSP), provided by Monique H. Slipher, are shown in Fig. S1. Auxilliary information and characteristics covering the molecular phylogenetic analysis as well as the alignment are given in the Tables S1–S3. [file 40529_2023_372_MOESM1_ESM.doc]

**Redisposition of apiosporous genera in the Xylariales, following the discovery of an authentic strain of *Induratia apiospora***

Marjorie Cedeño-Sanchez^1,2^, Rahel Schiefelbein^1,2^, Marc Stadler^1,2^, Hermann Voglmayr^3,4^, Konstanze Bensch^5^, Christopher Lambert^1,2,6^,*

^1^Department for Microbial Drugs, Helmholtz-Centre for Infection Research GmbH, Inhoffenstraße 7, 38124 Braunschweig, Germany

^2^Institute of Microbiology, Technische Universität Braunschweig, Spielmannstraße 7, 38106 Braunschweig

^3^Department of Botany and Biodiversity Research, University of Vienna, Rennweg 14, 1030, Vienna, Austria

^4^Institute of Forest Entomology, Forest Pathology and Forest Protection, Department of Forest and Soil Sciences, BOKU-University of Natural Resources and Life Sciences, Franz- Schwackhöfer-Haus, Peter-Jordan-Straße 82/I, 1190 Vienna, Austria

^5^Westerdijk Fungal Biodiversity Institute, Uppsalalaan 8, 3584 CT Utrecht, Netherlands.

^6^Department for Cell Biology, Helmholtz-Centre for Infection Research GmbH, Inhoffenstraße 7, 38124 Braunschweig, Germany.

*Corresponding author

E–mail: christopher.lambert@helmholtz-hzi.de; Tel.: +49-531-6181-2913

**Additional file 1**

Figure S1: Images of the specimen WSP73242 (**A**, **B**) and voucher (**C**, **D**) of the extant isotype of *I. apiospora* located at Shaw Mycology Herbarium (WSP) provided by Monique H. Slipher.


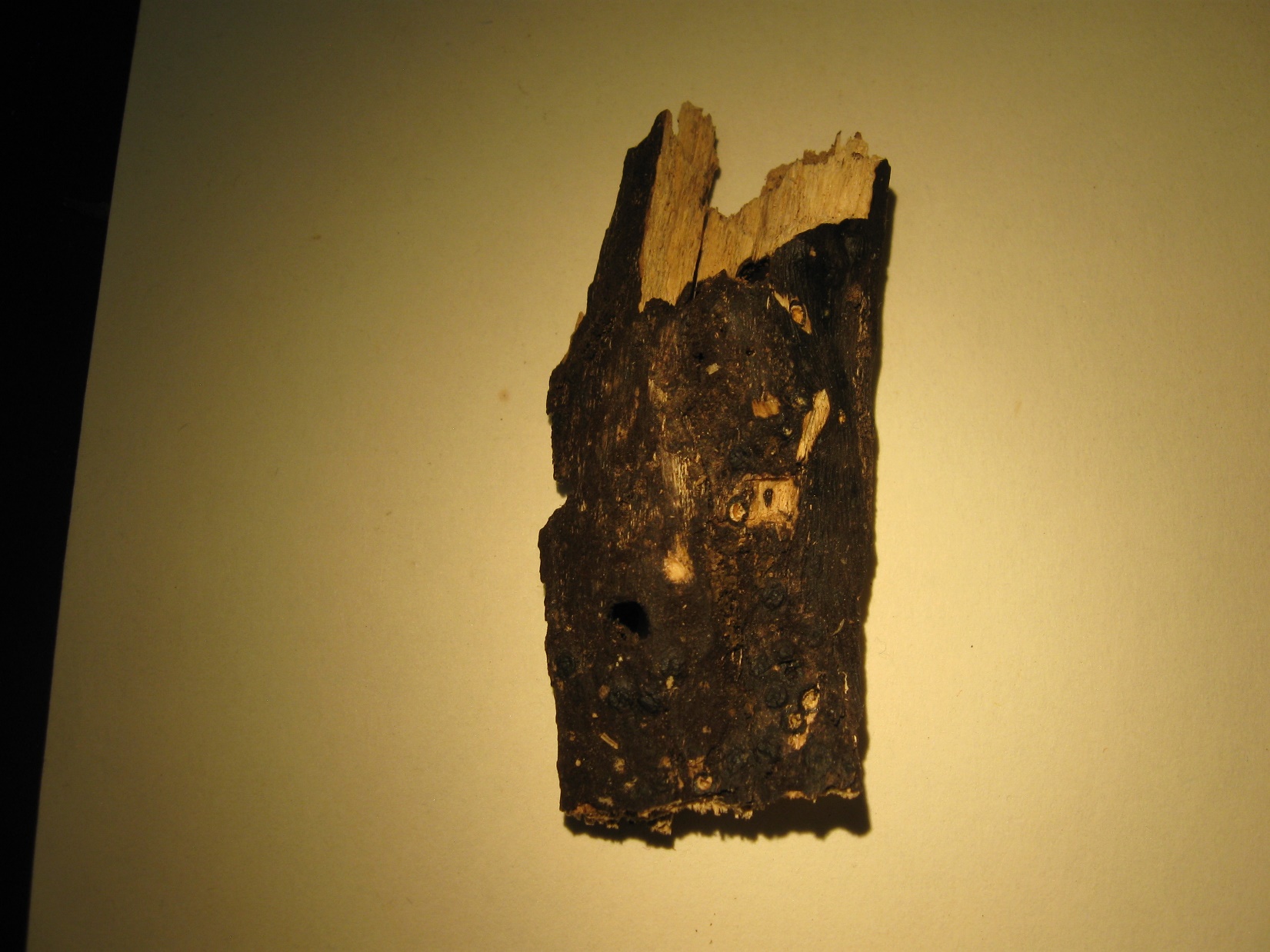

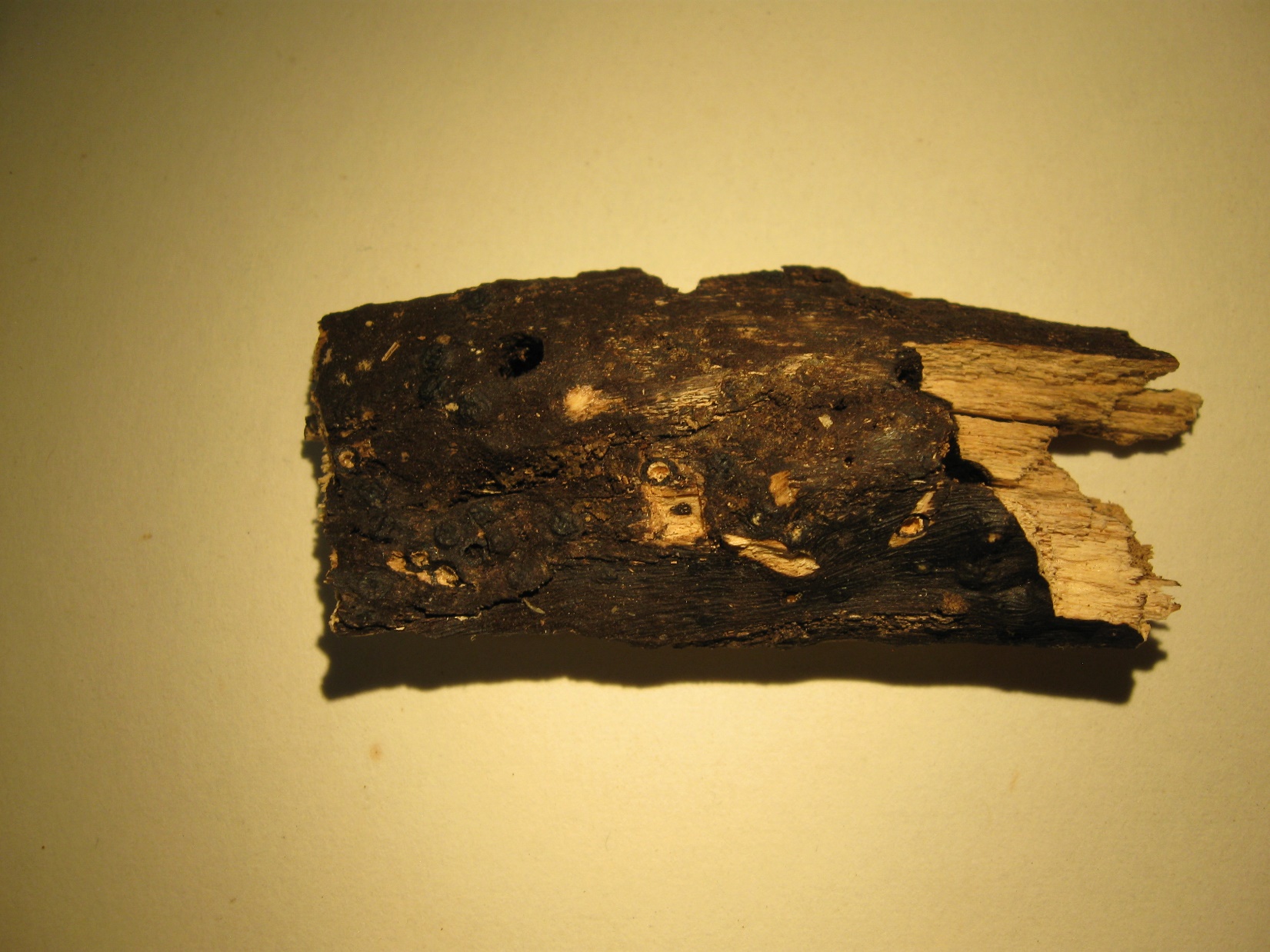

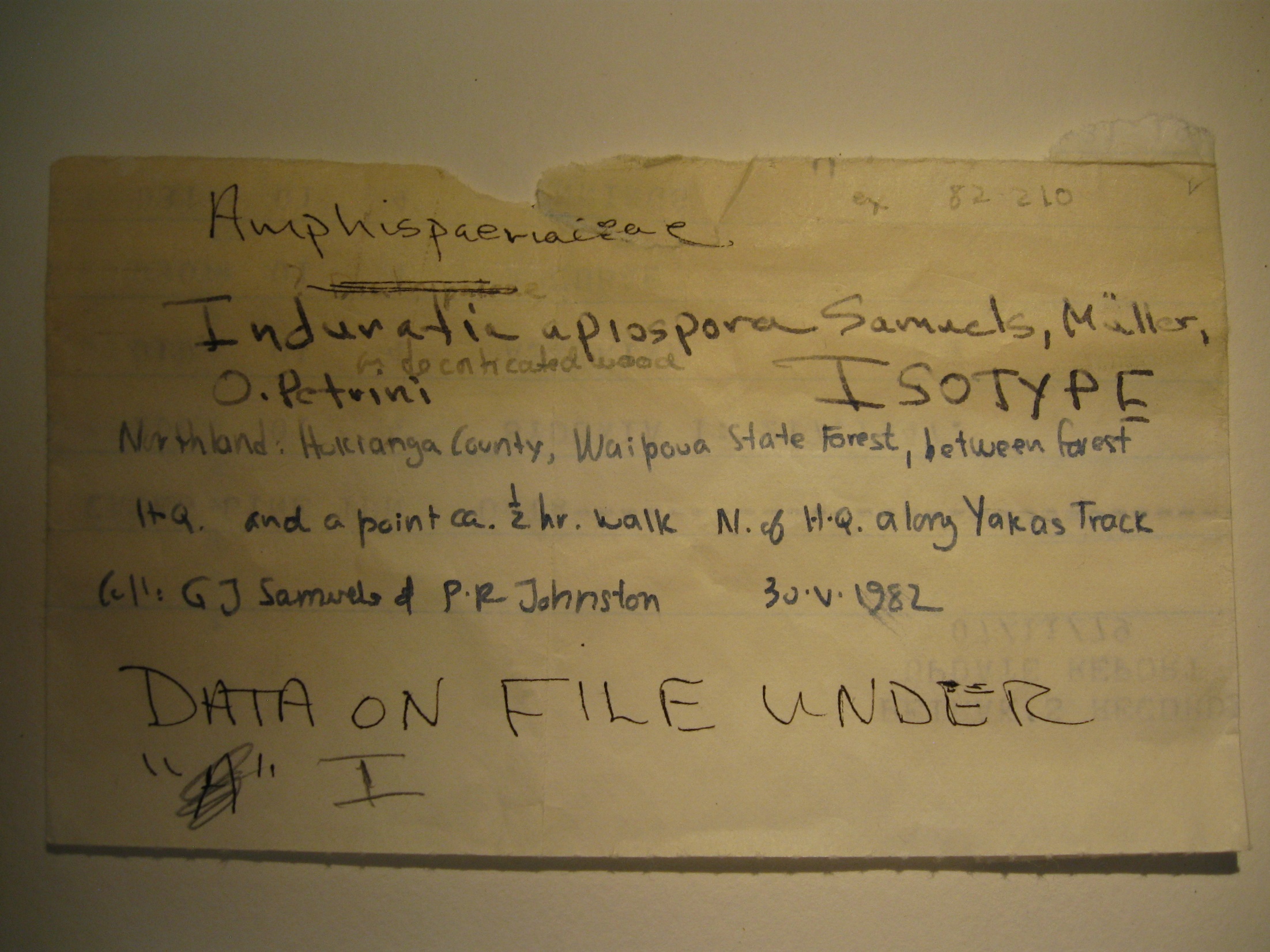

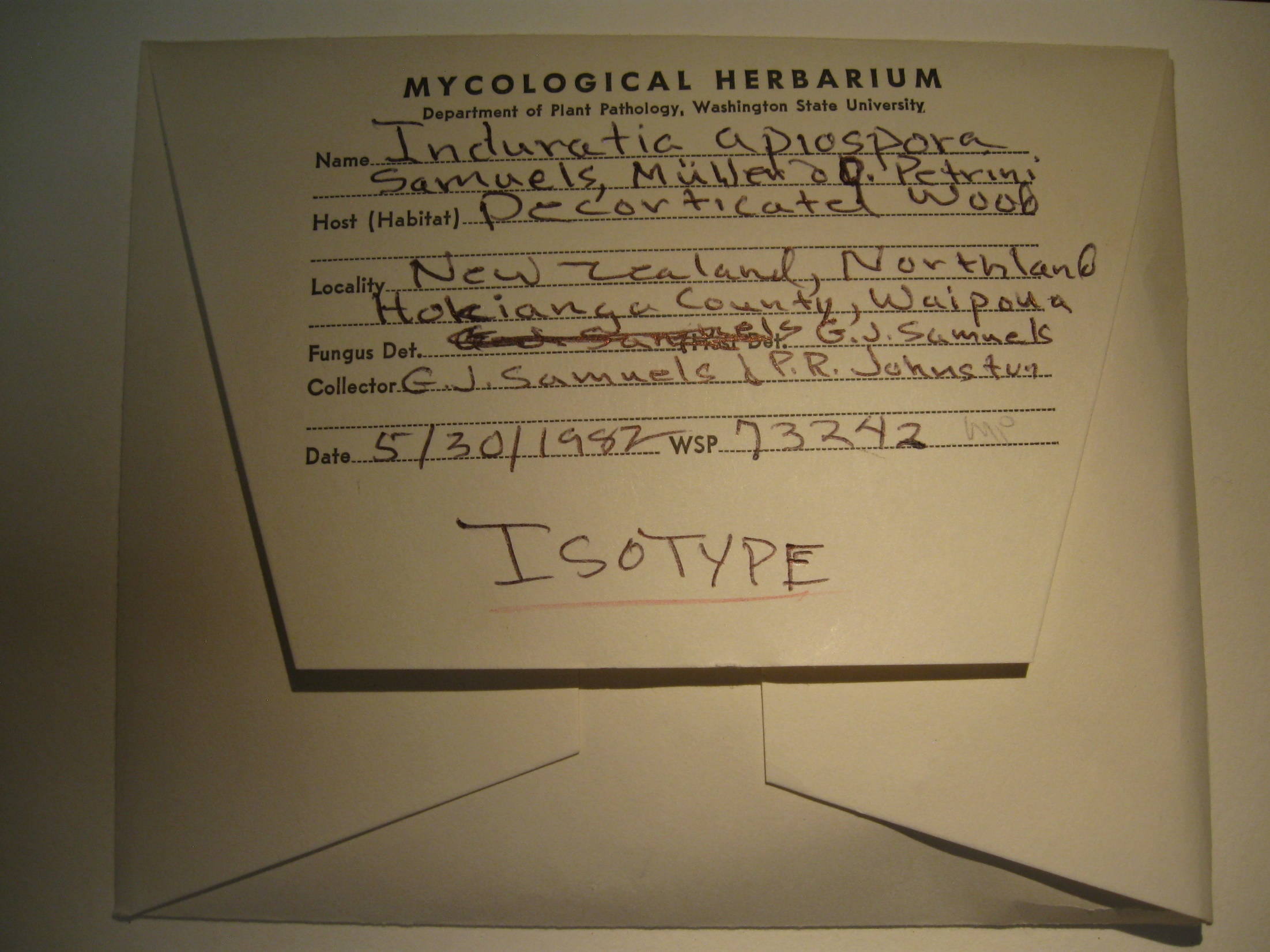


A

B

C

D

Table S1: Characteristics of the MAFFT and manually curated alignment used as input for IQTree 2.2.0.

| Locus | Sequence No. | Sites | Unique | Informative | Invariant | Constant |
| --- | --- | --- | --- | --- | --- | --- |
| ITS | 93 | 584 | 420 | 303 | 202 | 202 |
| LSU | 61 | 1329 | 418 | 177 | 1036 | 1036 |
| *rpb2* | 74 | 1235 | 854 | 669 | 485 | 485 |
| *tub2* | 72 | 1531 | 996 | 826 | 605 | 605 |

Table S2: Best fit nucleotide substitution models as tested by IQTree 2.2.0 as well as separate rates across sites as tested by ModelFinder.

| Locus | Substitution Model | Speed | Parameters |
| --- | --- | --- | --- |
| ITS | TIM2e+R5 | 1.0093 | TIM2e{1.66715,2.72385,5.15346}+FQ+R5  {0.446834,0.0415917,0.179125,0.421506,0.189711,  1.44012,0.136913,2.76526,0.047417,5.35898} |
| LSU | TNe+I+I+R3 | 0.1348 | TNe{3.71906,7.09652}+FQ+I{0.549012}+  R3{0.348862,0.648098,0.081921,5.30948,0.0202053,16.7751} |
| *rpb2* | TIM3+F+I+I+R4 | 1.4445 | TIM3{1.44502,4.84063,8.31598}  +F{0.260511,0.264506,0.255262,0.219721}  +I{0.329824} +R4{0.182136,0.128291,0.12863,  0.668883,0.206545,1.67847,0.152866,3.55813} |
| *tub2* | GTR+F+R4 | 1.3889 | GTR{1.0151,4.10865,1.39884,0.795228,4.70485}  +F{0.203967,0.312263,0.239172,0.244598}  +R4{0.454053,0.0144795,0.124032,0.415068,0.220573  ,1.41992,0.201341,3.1228} |

Table S3: Alignments used for the molecular phylogenetic inference:

# ITS

>Lopadostoma_turgidum_CBS_133207

TCATTACAGAGTTTCCAAACTCCCAAACCCATGTGAACTTACC-TATGTTGCCTCGGCAG

GACTTAGCTACCCGGTAG----TACCCGGTATTACGTACCCTGTAA--------------

---------------------------------TTGCTGGTAAGCCTGCCGAAGGACCAT

TAAACTCTGTTT-ATTGTGGCACTCTGAATCCAAAACTAAATAAGTTAAAACTTTCAACA

ACGGATCTCTTGGTTCTGGCATCGATGAAGAACGCAGCGAAATGCGATAAGTAATGTGAA

TTGCAGAATTCAGTGAATCATCGAATCTTTGAACGCACATTGCGCCCACTAGTATTCTGG

TGGGCATGCCTGTTCGAGCGTCATTTCGACCCCTAAGCCTTAGTTGTTTAGCGTTGGGGG

CTTGCGTCC-CGGCGCAACCCCTTAAATATCGTGGCAGGGCTCGCGTCGCCTTGAAGTGT

AGTAATTATGTTCTCACTTTTAGAGGGTGATCGCGCACAGCCAAGTAAAACCTTTATATC

TAGTGGTTGACCTCGGATCAGGTAGGAATACCCGCTGAACTTAA

>Creosphaeria_sassafras_ST.MA._14087

TCATTACAGAGTTTCTAA-CTCCCAAACCCATGTGAACCTACC-TATGTTGCCTCGGCGG

GAGAAGCCTACCCGGTAC----TACCCTGTAGGAACTACCCTGTAG--------------

---------------------------------TTGATGGCGGACCCGCCGGTGGACATT

TAAACTCTGTTC-ACTGTGATACTCTGAGTAATAAACTAAATAAGTTAAAACTTTCAACA

ACGGATCTCTTGGTTCTGGCATCGATGAAGAACGCAGCGAAATGCGATAAGTAATGTGAA

TTGCAGAATTCAGTGAATCATCGAATCTTTGAACGCACATTGCGCCCATTAGTATTCTAG

TGGGCATGCCTGTTCGAGCGTCATTTCAACCCTTAAGCCTTAGTTGCTTAGCGTTGGGAG

GTTGCCTC----TCGCAACTCCCTAAAATCAGTGGCAAGGACCACGACACCTTGAAGTGT

AGTAGTTTAATTCTCACTTTGG---AGGACCGTAGCCCAGCCATAAACCAACTATTTAAT

TATTGGTTGACCTCGGATCAGGTAGGAATACCCGCTGAACTTAA

>Diatrype_disciformis_CBS_197.49

------------------------------------------------------------

------------------------------------------------------------

------------------------------------------------------------

------------------------------------------------------------

------------------------------------------------------------

------------------------------------------------------------

------------------------------------------------------------

------------------------------------------------------------

------------------------------------------------------------

--------------------------------------------

>Eutypa_lata_UCR_EL1

TCATTACAGAGTTCCTAA-CTCC-AAACCCATGTGAACTTACC-TATGTTGCCTCGGCGG

GGAAG-CCTACCCGGTAC----TACCCTGTAGGAGCTACCCTGTAGCCCG----------

---------------------------------CTGCAGGCCTACCCGCCGGTGGACACT

TAAACTCTGTTTTTTAGTGATTATCTGAGTTTTATACTTAATAAGTTAAAACTTTCAACA

ACGGATCTCTTGGTTCTGGCATCGATGAAGAACGCAGCGAAATGCGATAAGTAATGTGAA

TTGCAGAATTCAGTGAATCATCGAATCTTTGAACGCACATTGCGCCCATTAGTATTCTAG

TGGGCATGCCTGTTCGAGCGTCATTTCGACCTTCAAGCCCTAGCTGCTTGGTGTTGGGAG

CCTATCT---CCGGATAGCTCCTCAAAAGCATTGGCGGAGTCGCGGTG-ACCCCAAGCGT

AGTAATTCT--TCTCGCTTTAGTGTACGGCTGACGTCTTGCCGTTAAACCCCCATTTTTT

AAATGGTTGACCTCGGATCAGGTAGGAATACCCGCTGAACTTAA

>Entoleuca_mammata_J.D.R._100

TCATTAGAGAGTTCCTAACTCCCCCAACCCCTGTGAACATACCATACGTTGCCTCGGCGG

GGGGC-------------------------------------------------------

----------------------------------------GCCACCCGCCGGCGGCCCAC

GAAACTCTGTTTTGCCCTGAATCTCTGAAGGATGAACTAAATCAGTTAAAACTTTCAACA

ACGGATCTCTTGGCTCTGGCATCGATGAAGAACGCAGCGAAATGCGATACGTAGTGTGAA

TTGCAGAATTCAGTGAATCATCGAATCTTTGAACGCACATTGCGCCCGCTAGTATTCTAG

CGGGCATGCCTGTTCGAGCGTCATTTCAACCCTTAAGCCCCTGTTGCTTAGTGTTGGGAG

CCGACGGCGTTGCCGTCGCTCCTCAAATCCAGTGGCGGAGCCGGTTCGCGCTCTAGGCGT

AGTAGATTTCATCTCGCCTACGGCCGGGCCGGCCCTCCTGCCGTTAAACCCCTCCTCCAC

AGAAGGTTGACCTCGAATCAGGTAGGAATACCCGCTGAACTT--

>Rosellinia_corticium_MUCL_51693

TCATTAGAGAGTG-CCTA-CTCCCAAACCCATGTGAACTTACCTTACGTTGCCTCGGCGG

GGGAGGGCTGC-----------CACCCC--------------------------------

----------------------------------------CCCTCCGCCAGGCGGCCCAC

CAAACCCTGTTTAGCCCTGAATCTCTGAGACGATAAAACAATGAGTTAAAACTTTCAACA

ACGGATCTCTTGGCTCTGGCATCGATGAAGAACGCAGCGAAATGCGATACGTAGTGTGAA

TTGCAGAATTTAGTGAATCATCGAATCTTTGAACGCACATTGCGCCCGCCAGTATTCTGG

CGGGCATGCCTGTTCGAGCGTCATTTCAACCCTTAAGCCCTTGTCGCTTAGTGTTGGGAG

CCGACGGCGTTGCCGTCGCTCCTCAAATCCAGTGGCGGAGCCGGTTCGCGCTCTGGGCGT

AGTAGATTTCATCTCGCCTGCAGCCGGGGCCGGCCCCCTGCCGTAAAACCACCATGTACC

CAAAGGTTGACCTCGAATCAGGTAGGAATACCCGCTGAACTTAA

>Xylaria_hypoxylon_CBS_122620

TCATTAAAGAGTTTTACAACTCCCAAACCCATGTGAACTTACCTTCTGTTGCCTCGGCAG

GTCGTGTTTACCCTGTGAGTCCTACCCTGTAG-CCCTACCTGGTAG--------------

---------------------------------ACACGGGTACGCCTGCCGGTGGCCCAT

GAAACTCTGTTAATTCTAGTTATTCTGAATCTATAACTAAATAAGTTAAAACTTTCAACA

ACGGATCTCTTGGTTCTGGCATCGATGAAGAACGCAGCGAAATGCGATAAGTAATGTGAA

TTGCAGAATTCAGTGAATCATCGAATCTTTGAACGCACATTGCGCCCATTAGTATTCTAG

TGGGCATGCCTGTTCGAGCGTCATTTCAACCCTTAAGCCCCTGTTGCTTAGCGTTGGGAG

CCTACAG---TTCTGTAGCTCCCCAAAGTTAGTGGCGGAGTCGGTTCACACTCTAGACGT

AGTAGATTCTATCTCGTCTGT-AGTGAGGCCGGTCCCCTGCCGTAAAACCCCCCAATTTT

TAAAGGTTGACCTCGGATCAGGTAGGAATACCCGCTGAACTTAA

>Xylaria_arbuscula_CBS_126415

TCATTAAAGAGTTAAACAACTCCTAAACCCATGTGAACCTACC-TTTGTTGCCTCGGCAG

GTCGCACTTACCCGGAGGGACCTACCCTGTAG-CCTTACCCGGTAG--------------

---------------------------------TTGCCGGATAACCTGCCGGTGGTCTAC

TAAACTCTGTTTACTAT-GTTATTCTGAATATATAACTAAATAAGTTAAAACTTTCAACA

ACGGATCTCTTGGTTCTGGCATCGATGAAGAACGCAGCGAAATGCGATAAGTAATGTGAA

TTGCAGAATTCAGTGAATCATCGAATCTTTGAACGCACATTGCGCCCATTAGTATTCTAG

TGGGCATGCCTGTTCGAGCGTCATTTCAACCCTTAAGCC-CTGTTGCTTAGCGTTGGGAG

CCTACAGATACTCTGTAGTTCCTTAAAGTTAGTGGCGGAGTCGGTTCACACTCTAGACGT

AGTAAATTTTATCTCGCCTAT-AGATGAGCCGGTCCCTTGCCGTAAAACCCCCTAATTTC

TAAAGGTTGACCTCGGATCAGGTAGGAATACCCGCTGAACTTAA

>Oligostoma_insidiosum_CBS_147288

TCATTAAAGAGTG-TATAACTCCCAAACCCATGTGAACATACCCTACGTTGCCTCGGCAG

GTCGCGGCTACCCTGTAGGACCTACCCCGTAG--CCTACCCGGTAG--------------

---------------------------------ACGCGGGTAAGCCTGCCGGCGGCCCAT

CAAACTCTGTTTCCTACTG-AATTCTGAACCTATAACTAAATAAGTTAAAACTTTCAACA

ACGGATCTCTTGGTTCTGGCATCGATGAAGAACGCAGCGAAATGCGATAAATAATGTGAA

TTGCAGAATTCAGTGAATCATCGAATCTTTGAACGCACATTGCGCCCATTAGTATTCTAG

TGGGCATGCCTGTTCGAGCGTCATTTCAACCCTTAAGCCCCTGCTGCTTAGTGTTGGGAG

CCTACCG---TACGGTAGCTCCCCAAAGTTAGTGGCGGAGTCGGTTCACACTCTAGACGT

AGTAAAT-CTATCTCGCCTATCAGTTGGACCGGTCCCTTGCCATAAAACACCC--AATTT

TAAAGGTTGACCTCGGATCAGGTAGGAATACCCGCTGAACTTAA

>Xylaria_laevis_HAST_419

TCATTAAAGAGTTCTATAACTCCCAAACCCATGTGAACATACCATACGTTGCCTCGGCAG

GTCGCGCCTACCCCGTAGGTCCTACCCTATAG--CCTACCCGGTAG--------------

---------------------------------ACGCGGGTAAGCCTGCCGGCGGCCCAA

GAAACTCTGTTTAGTATTG-AATTCTGAACCTATAACTAAATAAGTTAAAACTTTCAACA

ACGGATCTCTTGGTTCTGGCATCGATGAAGAACGCAGCGAAATGCGATAAGTAATGTGAA

TTGCAGAATTCAGTGAATCATCGAATCTTTGAACGCACATTGCGCCCATTAGTATTCTAG

TGGGCATGCCTGTTCGAGCGTCATTTCAACCCTTAAGCCCTCGTTGCTTAGTGTTGGGAG

CCTACGC---TACCGTAGCTCCTCAAAGTTAGTGGCGGAGTCGGTTCACACTCTAGACGT

AGTA-ATTTTATCTCGCCTATCAGTTGGACCGGTCCCCTGCCGTAAAACACCCCAATTTC

TAAAGGTTGACCTCGGATCAGGTAGGAATACCCGCTGAACTT--

>Xylaria_longipes_CBS_148.73

-CATTAAAGAGTTCTATAACTCCCAAACCCATGTGAACATACCTTACGTTGCCTCGGCAG

GTCGTGCCTACCCCGTAGGTCCTACCCTGTAG--CCTACCCGGTAG--------------

---------------------------------ACGCGGGTAAGCCTGCCGGCGGCCCAC

GAAACTCTGTTTAGTATTG-AATTCTGAACCTATAACTAAATAAGTTAAAACTTTCAACA

ACGGATCTCTTGGTTCTGGCATCGATGAAGAACGCAGCGAAATGCGATAAGTAATGTGAA

TTGCAGAATTCAGTGAATCATCGAATCTTTGAACGCACATTGCGCCCATTAGTATTCTAG

TGGGCATGCCTGTTCGAGCGTCATTTCAACCCTTAAGCCCCTGTTGCTTAGTGTTGGGAG

CCTACGG---TATAGTAGCTCCTGAAAGTTAGTGGCGGAGTCGGTTCACACTCTAGACGT

AGTAGATTTTATCTCGCCTATCAGTTGGACCGGTCCCTTGCCGTAAAACCACCTAATTTC

TAAAGGTTGACCTC------------------------------

>Xylaria_digitata_HAST_919

TCATTAAAGAGTTTTATAACTCCCAAACCCATGTGAACATACCTTACGTTGCCTCGGCAG

GTCGCGCCTACCCCGTAAGTCCTACCCTGTAG--CCTACCCGGTAG--------------

---------------------------------ACACGGGTAAGCCTGCCGGCGGCCCAC

GAAACTCTGTTTAGTATTG-AATTCTGAACCTATAACTAAATAAGTTAAAACTTTCAACA

ACGGATCTCTTGGTTCTGGCATCGATGAAGAACGCAGCGAAATGCGATAAGTAATGTGAA

TTGCAGAATTCAGTGAATCATCGAATCTTTGAACGCACATTGCGCCCATTAGTATTCTAG

TGGGCATGCCTGTTCGAGCGTCATTTCAACCCTTAAGCCTCTGTTGCTTAGTGTTGGGAG

CCTACGG---TATAGTAGCTCCTCAAAGTTAGTGGCAGAGTCGGTTCACACTCTAGACGT

AGTAGATTTTATCTCGCCTATTAGTTGGACCGGTCCCTTGCCGTAAAACCCCCTAATTTC

TAAAGGTTGACCTCGGATCAGGTAGGAATACCCGCTGAACTT--

>Leptomassaria_simplex_CBS_147282

TCATTAAAGAGTGTTACAACTCCTAAACCCATGTGAACATACCTTACGTTGCCTCGGCAG

GTCGCGCCTACCCTGTGG----------G----CCCTACCCGGTAG--------------

---------------------------------ACGCCGGTAAGCCTGCCGGTGGCCCAC

GAAACTCTGTTTGATATTG-AATTCTGAACCTATAACTAAATAAGTTAAAACTTTCAACA

ACGGATCTCTTGGTTCTGGCATCGATGAAGAACGCAGCGAAATGCGATAAGTAATGTGAA

TTGCAGAATTCAGTGAATCATCGAATCTTTGAACGCACATTGCGCCCATTAGTATTCTAG

TGGGCATGCCTGTTCGAGCGTCATTTCAACCCTTAAGCCTCTGCTGCTTAGTGTTGGGAG

CCTACAG---CATAGTAGCTCCTCAAAGTTAGTGGCGGAGTCGGTTCACACTCTAGACGT

AGTAGATTTTATCTCGCCTATCAGTTGGACCGGTCCCCTGCCGTAAAACACCC-AATTTT

TAAAGGTTGACCTCGGATCAGGTAGGAATACCCGCTGAACTTAA

>Stilbohypoxylon_elaeicola_Y.M.J._173

TCATTAAAGAGTGTAACAACTCCCAAACCCATGTGAACATACCTTACGTTGCCTCGGCAG

GTCGCGCCCACCCCGTCGACCCTACCCTGTAG-CCCTACCCGGTGG--------------

---------------------------------GAGCGGGTAAGCCTGCCGGCGGCCCAC

AAAACTCTGTTTAGCATTGCACTTCTGAACGTATAACTAAATAAGTTAAAACTTTCAACA

ACGGATCTCTTGGTTCTGGCATCGATGAAGAACGCAGCGAAATGCGATAAGTAATGTGAA

TTGCAGAATTCAGTGAATCATCGAATCTTTGAACGCACATTGCGCCCATTAGTATTCTAG

TGGGCATGCCTGTTCGAGCGTCATTTCAACCCTTAAGCCCCTGTTGCTTAGCGTTGGGAG

CCTACTC-----CGGTAGCTCCTCAAAGTTAGTGGCGGAGTCGGTACACACTCTAGACGT

AGTAATTCTCACCTCGCCTACGAGATGTGCCGGTCCCCTGCCGTTAAACACACCAATTTC

CAAAGGTTGACCTCGGATCAGGTAGGAATACCCGCTGAACTT--

>Xylaria_ianthinovelutina_HAST_553

TCATTAAAGAGTGTAATAACTCCCAAACCCATGTGAACATACCTTACGTTGCCTCGGCAG

GTCGCGCCTACCCCGTAGGCCCTACCCTGTGG-CGCTACCCGGTAG--------------

---------------------------------ACGCGGGTAAGCCTGCCGGCGGCCCAC

GAAACTCTGTTTAGCATTGTACTTCTGAACATATAACTAAATAAGTTAAAACTTTCAACA

ACGGATCTCTTGGTTCTGGCATCGATGAAGAACGCAGCGAAATGCGATAAGTAATGTGAA

TTGCAGAATTCAGTGAATCATCGAATCTTTGAACGCACATTGCGCCCATTAGTATTCTAG

TGGGCATGCCTGTTCGAGCGTCATTTCAACCCTTAAG-CCCTGTTGCTTAGCGTTGGGAG

CCTGCTA-----TCGCAGCTCCTCAAAGTTAGTGGCGGAGTCGGTACACACTCTAGACGT

AGTAATTCTTATCTCGCCTATAGGTTGTGCCGGTCCCCTGCCGTTAAACCCCCCAATTTC

TAAAGGTTGACCTCGGATCAGGTAGGAATACCCGCTGAACTT--

>Xylaria_polymorpha_MUCL_49884

TCATTAAAGAGTTTTATAACTCCCAAACCCATGTGAACATACCGTACGTTGCCTCGGCAG

GCTGCATCT--CCTGTGAGTCCTACCCTGTAG--CTTAGCGGGAAG--------------

---------------------------------ATGCATTAAAGCCTGCCGGCGGCCCAT

TAAACTCTGTTTATTTTTG-AATTCTGAGGCTATAA-TAAATAAGTTAAAACTTTCAACA

ACGGATCTCTTGGTTCTGGCATCGATGAAGAACGCAGCGAAATGCGATAAGTAATGTGAA

TTGCAGAATTTAGTGAATCATCGAATCTTTGAACGCACATTGCGCCCACTAGTATTCTAG

TGGGCATGCCTGTTCGAGCGTCATTTCAACCCTTAAGCCCCTGTTGCTTAGTGTTGGGAG

CCTACGG---CAGCGTAGCTCCTCAAATGTAGTGGCGGAGTTGGTTCACACTCTAGACGT

AGTAATTTTTATCTCGCCTGTGAGTTGGACCGGTCCCTCGCCGTAAAACCCCCAAAATTT

TAAAGGTTGACCTCGGATCAGGTAGGAATACCCGCTGAACTTAA

>Xylaria_atrosphaerica_HAST_91111214

TCATTAAAGAGTGTTATAACTCCTAAACCCATGTGAATATACCGTATGTTGCTTCGGTAG

GCTGGATCTATCCCGTGG----------------TTCCCTTGGGAG--------------

---------------------------------ATGTGGAAAAGCCTGCCGGCGGCCTAT

TTAATCCTGTTTATTTTTG-AATTCTGAGGTTATAA-TTAATCAGTTAAAACTTTCAACA

ACGGATCTCTTGGTTCTGGCATCGATGAAGAACGCAGCGAAATGCGATAAGTAATGTGAA

TTGCAGAATTTAGTGAATCATCGAATCTTTGAACGCACATTGCGCCCATTAGTATTCTAG

TGGGCATGCCTGTTCGAGCGTCATTTCAACCCTTAAGCCTCTGTTGCTTAGTGTTGGGAG

CCTACGA---TGGCGTAGCTCCTCAAAAGTAGTGGCGGAGTCGGTTCACACTCTAGACGT

AGTAAATTTTATCTCGCCTGTGAGTTGGACCGGTCCCTTGCCGTAAAACCCCCCTATTTT

ACAAGGTTGACCTCGGATCAGGTAGGAATACCCGCTGAACTT--

>Amphirosellinia_nigrospora_HAST_91092308

TCATTAAAGAGTTCTATAACTCCCAAACCCATGTGAACATACCTTACGTTGCCTCGGCGA

GCCGCGCCCACCACGCAGACCCCGCCTTGCGG--CCTACTTGGCGA--------------

---------------------------------ACGCGGGGTAACCCGCCGGCGGCCCAC

GAAACTCTGTTTAATACTATATCTCTGAACCTATAACTAAATAAGTTAAAACTTTCAACA

ACGGATCTCTTGGTTCTGGCATCGATGAAGAACGCAGCGAAATGCGATAAGTAATGTGAA

TTGCAGAATTCAGTGAATCATCGAATCTTTGAACGCACATTGCGCCCATTAGTATTCTAG

TGGGCATGCCTGTTCGAGCGTCATTTCAACCCTTAAGCCTCTGTCGCTTAGTGTTGGGAG

CCTACAG-----CGGTAGCTCCCCAAAGTTAGTGGCGGAGCTGGTTCACACTTCAGGCGT

AGTAGTA-TTATCTCGCCCGCGAGTCGGACCGGCCTCCTGCCGTAAAACCCCCTAATTTA

TCAAGGTTGACCTCGGATCAGGTAGGAATACCCGCTGAACTT--

>Dematophora_necatrix_CBS_349.36

-CATTAAAGAGTTCTATAACTCCCAAACCCATGTGAACATACCACGCGTTGCCTCGGCAG

GTCGCGCCTACCCCGAAGGCCCTACCCTGTAG--CCTACCCGGTGG--------------

---------------------------------GCGCGGGCCAACCTGCCGGCGGCCCAC

GAAACTCTGTTTAGCATTG-AATTCTGAACACATAACTAAATAAGTTAAAACTTTCAACA

ACGGATCTCTTGGTTCTGGCATCGATGAAGAACGCAGCGAAATGCGATAAGTAATGTGAA

TTGCAGAATTCAGTGAATCATCGAATCTTTGAACGCACATTGCGCCCATTAGTATTCTAG

TGGGCATGCCTGTTCGAGCGTCATTTCAACCCTTAAGCCCCTGTTGCTTAGTGTTGGGGG

CCTGCAGCG-TGCTGCAGCCCCTCGAAGTCAGTGGCGGAGTCGGTCCACACTCTAGACGT

AGTAGATTTCATCTCGCCTAT-GGTTGTGCCGGTCCCCTGCCGTAAAACACCCCCTATAC

CAAAGGTTGACCTCN-----------------------------

>Xylaria_oxyacanthae_859_JDR

TCATTACAGAGTTTCGTAACTCCCAAACCCATGTGAACCTACTCTGCGTTGCCTCGGCAG

GTCGCGCTCACCCGGTGGACCCTACCCTGTAG-GCCTACCCGGTGG--------------

---------------------------------ACGCGGTAAAGCCTGCCGGCGGCCCAC

AAACTCTTGTTTATTATTGATCTTCTGAGCTTATAACTAAATAAGTTAAAACTTTCAACA

ACGGATCTCTTGGTTCTGGCATCGATGAAGAACGCAGCGAAATGCGATAAGTAATGTGAA

TTGCAGAATTCAGTGAATCATCGAATCTTTGAACGCACATTGCGCCCATTAGTATTCTAG

TGGGCATGCCTGTTCGAGCGTCATTTCAACCCTTAAGCCCCTGCTGCTTAGTGTTGGGAG

CCTACAG---CGATGTAGCTCCTCGAAGTTAGTGGCGGAGTCGGTTCACACTCTAGACGT

AGTAAATATTGTCTCGCCTAC-GGTTGTGCCGGTCCCCTGCCGTAAAACACCCCGTTTTC

TCAAGGTTGACCTCGGATCAGGTAGGAATACCCGCTGAACTT--

>Albicollum_vincensii_CBS_147286

TCATTAAAGAGTATAAAAACTCCCAAACCCATGTGAATATACCACTTGTTGCCTCGGCAG

GTCGCGCTTACCCGGTAACTCCTACCCTGGAG-CCCTACCCTGTAG--------------

---------------------------------ACGCGAGTAAGCCTGCCGACGGCCCCT

AAAACACTGTTTACCACTGAAACTCTGAATACATAACTAAATAAGTTAAAACTTTCAACA

ACGGATCTCTTGGTTCTGGCATCGATGAAGAACGCAGCGAAATGCGATAAGTAATGTGAA

TTGCAGAATTCAGTGAATCATCGAATCTTTGAACGCACATTGCGCCCATTAGTATTCTAG

TGGGCATGCCTGTTCGAGCGTCATTTCAACCCTTAAGCC-CTGTTGCTTAGCGTTGGGAG

CCTACCG---TGTGGTAGTTCCCTAAAATCAGTGGCGGAGTCGGTTCATACTCTAAGCGT

AGTAAATTTTCTCTCGCTTCTGTAGTTGGGTCGTCGCCTGCCGTAAAACCCCCCAAT-TT

ATAAGGTTGACCTCGGATCAGGTAGGAATACCCGCTGAACTTAA

>Albicollum_longisporum_CBS_147283

TCATTACAGAGTATAAAAACTCCTAAACCCATGTGAATCTACCTATTGTTGCTTCGGCAG

GTCGCATCTATATGGTAACCCCAACTCTGGGG-CCCTACCCTATAG--------------

---------------------------------TTGCGGGTAAGCCTGCCGAGGGCCCTT

AAAACACTGTATGTAATTGAAATTCTGAATTGATAACTAAAT-AGTTAAAACTTTCAACA

ACGGATCTCTTGGTTCTGGCATCGATGAAGAACGCAGCGAAATGCGATAAGTAATGTGAA

TTGCAGAATTCAGTGAATCATCGAATCTTTGAACGCACATTGCGCCCATTAGTATTCTAT

TGGGCATGTCTGTTCGAGCGTCATTTCAACCCTTAAGCC-TCGTTGCTTAGCGTTGGGAG

CTTACCGTCCTGCGGTAACTCTCTAAAAGTAGTGGCGGAGTCGGTTCTCACTCTAAGCGT

AGTACTATATATCTCGCTTCTGTAGTGGTATCGTCCCTCGCCGTAAAACACCCCAA-TTA

TCAAGGTTGACCTCGGATCAGGTAGGAATACCCGCTGAACTTAA

>Stromatoneurospora_phoenix_BCC_82040

TCATTATAGAGTT-TTCAAACTCCCAACCCATGTGAACATACCTATTGTTGCCTCGGCAG

GTCGCAGACTGCCGGAAAGTCCCCTACCCTGG-GCCTACCCGGGAG--------------

---------------------------------CTGCGGTCAAGCCTGCCGGCGGCCCGC

AAAACTCTGTTTAGCATTGAAATTCTGAAT-CATAACTAAATAAGTTAAAACTTTCAACA

ACGGATCTCTTGGTTCTGGCATCGATGAAGAACGCAGCGAAATGCGATAAGTAATGTGAA

TTGCAGAATTCAGTGAATCATCGAATCTTTGAACGCACATTGCGCCCATTAGTATTCTAG

TGGGCATGCCTGTCCGAGCGTCATTTCAACCCTTAAGCCCCTGTTGCTTAGTGTTGGGAG

ACTAC-G---CAGCGTAGCTCCTCAAAGTTAGTGGCAGAGTCAGTTCAAACTCTAGGTGT

AGTAAATCTCTTCTCGCCTCTGTAGTTGGGCTGCCCCTA---------------------

--------------------------------------------

>Sarcoxylon_compunctum_CBS_359.61

TCATTATAGAGTT-TACAAACTCCAAACCCCTGTGAACATACCTATCGTTGCCTCGGCAG

GTCGTGGCCTACCTGTAAACCTCCCACCGGGC-TGCTACCCGGGAGT-------------

---------------------------------CCGCGGTGAAGCCTGCCGGCGGCCCAC

GAAACTCTGTTA-TTATTGAATCTCTGAAT-CTTAACTAAATAAGTTAAAACTTTCAACA

ACGGATCTCTTGGTTCTGGCATCGATGAAGAACGCAGCGAAATGCGATAAGTAATGTGAA

TTGCAGAATTCAGTGAATCATCGAATCTTTGAACGCACATTGCGCCCATTAGTATTCTAG

TGGGCATGCCTGTTCGAGCGTCATTTCAACCCTTAAGCCCCTGTTGCTTAGTGTTGGGAG

CCTACGT---TGGCGTAGCTCCTCAAAGTTAGTGGCAGAGTCAGTTCATACTCTAGGCGT

AGTAATTTTTATCTCGCTTTTGTAGTTGGGCTGGCCCTCGCCGTAAAACCCCCTATTTTT

AAAAGGTTGACCTCGGATCAGGTAGGAATACCCGCTGAACTTAA

>Podosordaria_mexicana_WSP176

TCATTATAGAGTT-TACAAACTCCCAACCCCTGTGAACATACCTGACGTTGCTTCGGCAG

GCCTC-------------------------------------------------------

----------------------------------------ACAGCCTGCCAGCAGCCCAT

CAAACTCTGTTTAATATTGAATCTCTGAAT-CTTAACTAAATAAGTTAAAACTTTCAACA

ACGGATCTCTTGGTTCTGGCATCGATGAAGAACGCAGCGAAATGCGATAAGTAATGTGAA

TTGCAGAATTCAGTGAATCATCGAATCTTTGAACGCACATTGCGCCCATTAGTATTCTAG

TGGGCATGCCTGTTCGAGCGTCATTTCAACCCTTAAGCCCCCGTTGCTTAGTGTTGGGAG

CCTACTC---CTCTGTAGCTCCTCAAAGTCAGTGGCAGAGTCGGTTCAAACTCTAGGCGT

AGTAATTCTTATCTCGTCTCTGTAGTTGGGCTGGCCCTCGCCATAAAACCCCCTATTTTT

TAAAGGTTGACCTCGGATCAGGTAGGAATACCCGCTGAACTT--

>Poronia_punctata_CBS_656.78

TCATTATAGAGTTTAAAAACTCCCAAACCCATGTGAACTTACCTATCGTTGCCTCGGCAG

GCTGTGCCGGCCCCGCGGGCCCTACCCCGTAACACCCCCCCGGGCG--------------

---------------------------------GCGCCTAAAGGCCTGCCGGCGGCCCAC

GAAACTCTTTGTA-TACTGTAATTCTGAATCAATAACTAAATAAGTTAAAACTTTCAACA

ACGGATCTCTTGGTTCTGGCATCGATGAAGAACGCAGCGAAATGCGATAAGTAATGTGAA

TTGCAGAATTCAGTGAATCATCGAATCTTTGAACGCACATTGCGCCCATTAGTATTCTAG

TGGGCATGCCTGTTCGAGCGTCATTTCAACCCTTAAGCCCCCGTTGCTTAGCGTTGGGAG

CCTGCCGGGGCCCCGCAGCTCCCCAAAGACAGTGGCAGAGTCGGTACGTACTCTAGGCGT

AGTAAATCTTTTCTCGCCTCTGCAGTCGGTCTGGCACTCGCCGTAAAACACCCCATTTAT

CAAAGGTTGACCTCGGATCAGGTAGGAATACCCGCTGAACTTAA

>Entalbostroma_erumpens_ICMP_21152

TCATTAAAGAGTTCCCTAACTCCCAAACCCATGTGAACTTACC-TCTGTTGCCTCGGCGG

GCCGCACTCACCGCGTAG---------------CCCTACCCGGTAG--------------

---------------------------------GTGCGGA-CGGCCCGCCGGTGGCCCAC

GAAACTCTGTTTAACATTG-CATTCTGAACCTACAACTAAATAAGTTAAAACTTTCAACA

ACGGATCTCTTGGTTCTGGCATCGATGAAGAACGCAGCGAAATGCGATAAGTAATGTGAA

TTGCAGAATTCAGTGAATCATCGAATCTTTGAACGCACATTGCGCCCATTAGTATTCTAG

TGGGCATGCCTGTTCGAGCGTCATTTCAACCCTTAAGCCCCTGTTGCTTAGTGTTGGGAG

CCTACAG---CCCTGTAGCTCCCCAAAGTTAGTGGCGGGGCCGGTTCCATCCCTAGACGT

AGTAAATCTTATCTCGCCTAC-GGACGCGCCGGTCCCTCGCCGTAAAACACCCC-ATTTT

CTCAGGTTGACCTCGGATCAGGTAGGAATACCCGCTGAACTT--

>Induratia_apiospora_ATCC_60639

TCATTAAAGAGTTTTTAAACTCCTAAACCCATGTGAACATACCTATTGTTGCCTCGGTAG

GTCGTGCCTACCCTGTAG----TAC---GTAT----------------------------

---------------------------------ATGCTACATGGCCTACCGAAGGCCTTT

-AAACTCTGTTA-TCAGTGGAATTCTGAACTCATAACTAAATAAGTTAAAACTTTCAACA

ACGGATCTCTTGGTTCTGGCATCGATGAAGAACGCAGCGAAATGCGATAAGTAATGTGAA

TTGCAGAATTCAGTGAATCATCGAATCTTTGAACGCACATTGCGCCCATTAGTATTCTAG

TGGGCATGCCTGTTCGAGCGTCATTTCAACCCCTAAGCC-TTGTTGCTTACTGTTGGGAG

TCTACATACC--CTGTAGTTCCTTAAAGTTAGTGGCGGAGTTAGGTCATACTCTAAGCGT

AGTAAT--TCATCTCGCTTTTGAGGTGATTTA---TACAGCCGTAAAACCTTT-AATTTT

AAGTGGTTGACCTCGGATCAGGTAGGAATACCCGCTGAACTTAA

>Barrmaelia_rhamnicola_BR

TCATTATAGAGTTATATAAACTCC-AACCCATGTGAACATACCAATTGTTGCCTCGGCAG

GCTGTGTCTACCCTGTAGAACCTACCCTGTAG-ACCTACCCGGTGGC-------------

---------------------------------ACGCTACAAGGCCTGCCGAAGG-CCCC

TGAACTCTATTATCTATTGGAATTATGAATACGCAACTTAATAAGTTAAAACTTTCAACA

ACGGATCTCTTGGTTCTGGCATCGATGAAGAACGCAGCGAAATGCGATAAGTAATGTGAA

TTGCAGAATTCAGTGAATCATCGAATCTTTGAACGCACATTGCGCCCATTAGTATTCTAG

TGGGCATGCCTGTTCGAGCGTCATTTCGACCCTTAAGCC-ATGTTGCTTAGTGTTGGGAG

CCTACGT---CCCTGTAGCTTCTCAAAGTCAGTGGCGGAGTCGGGTCACACTCTAAGCGT

AGTAATCTTT-TCTCGCTTCTGTAGTTGTCCCGGTTCCTGCCGTAAAACCCCTAATTTTA

TCTAGGTTGACCTCGGATCAGGTAGGAATACCCGCTGAACTTAA

>Barrmaelia_macrospora_CBS_142768

TCATTATAGAGTTACACAAACTCC-AACCCATGTGAACATACCAACTGTTGCCTCGGCTG

GCTGTGCCTACCCTGTAGAACCTACCCTGTAG-ACCTACCCGGTGGT-------------

---------------------------------ACGCTACAAGGCCTGCCGAAGG-CCCC

CGAACTCTATTATCTATTGGAATTCTGAATACGCAACTTAATAAGTTAAAACTTTCAACA

ACGGATCTCTTGGTTCTGGCATCGATGAAGAACGCAGCGAAATGCGATAAGTAATGTGAA

TTGCAGAATTCAGTGAATCATCGAATCTTTGAACGCACATTGCGCCCATTAGTATTCTAG

TGGGCATGCCTGTTCGAGCGTCATTTCGACCCTTAAGCC-CTGTTGCTTAGTGTTGGGAG

CCTACGT---CCCTGTAGCTCCTCAAAGTCAGTGGCGGAGTCGGGTCACACTCTAAGCGT

AGTCATCTTT-TCTCGCTTCTGTAGTTGTCCCGGTTCCTGCCGTAAAACCCCTAA-TTTA

TCTAGGTTGACCTCGGATCAGGTAGGAATACCCGCTGAACTTAA

>Barrmaelia_rappazii_CBS_142771

TCATTATAGAGTTATACAAACTCC-AACCCATGTGAACATACCAACTGTTGCCTCGGCAG

GCTGTGTCTACCCTGTAGAACCTACCCTGTAG-ACCTACCTGGTGGC-------------

---------------------------------ACGCCACAA-GCCTGTCGAAGG-CCCC

TGAACTCTATTATCTATTGGAATTCTGAATACGCAACTTAATAAGTTAAAACTTTCAACA

ACGGATCTCTTGGTTCTGGCATCGATGAAGAACGCAGCGAAATGCGATAAGTAATGTGAA

TTGCAGAATTCAGTGAATCATCGAATCTTTGAACGCACATTGCGCCCATTAGTATTCTAG

TGGGCATGCCTGTTCGAGCGTCATTTCGACCCTTAAGCC-CTGTTGCTTAGTGTTGGGAG

CCTACGT---CCCTGTAGCTCCTCAAAGTCAGTGGCGGAGTCGGGTCACACTCTAAGCGT

AGTAATCTTT-TCTCGCTTCTGTAGTTGTCCCGGTTCCTGCCGTAAAACCCCTAATTTTA

TCTAGGTTGACCTCGGATCAGGTAGGAATACCCGCTGAACTTAA

>Barrmaelia_oxyacanthae_CBS_142770

TCATTATAGAGTTATACAAACTCC-AACCCATGTGAACATACCAACTGTTGCCTCGGCAG

GCTGTGCCTACCCTGTAGAAC-TACCCTGTAG-ACCTACCTGGTGGC-------------

---------------------------------ACGCTACAAGGCCTGTCGAAGG-CCCC

CGAACTCTATTATCTATTGGAACTCTGAATACGCAACTTAATAAGTTAAAACTTTCAACA

ACGGATCTCTTGGTTCTGGCATCGATGAAGAACGCAGCGAAATGCGATAAGTAATGTGAA

TTGCAGAATTCAGTGAATCATCGAATCTTTGAACGCACATTGCGCCCATTAGTATTCTAG

TGGGCATGCCTGTTCGAGCGTCATTTCGACCCTTAAGCC-CTGTTGCTTAGTGTTGGGAG

CCTACGT---CCCTGTAGCTCCTCAAAGTTAGTGGCGGAGTCGGGTCACACTCTAAGCGT

AGTAATCTTT-TCTCGCTTCTGTAGTTGCCCTGGTTCCTGCCGTAAAACCCCTAATTTTA

TCTAGGTTGACCTCGGATCAGGTAGGAATACCCGCTGAACTTAA

>Barrmaelia_moravica_CBS_142769

TCATTATAGAGTTATACGAACTCC-AACCCATGTGAACATACCAACTGTTGCCTCGGCAG

GCCGTGTCTACCCTGTAGACCTTACCCTGTAG-ACCTACCCGGTGGC-------------

---------------------------------ACGCCACAAGGCCTGCCGAAGG-CCCC

TGAACTCTATTATCTATTGGAATTCTGAATACGCAACTTAATAAGTTAAAACTTTCAACA

ACGGATCTCTTGGTTCTGGCATCGATGAAGAACGCAGCGAAATGCGATAAGTAATGTGAA

TTGCAGAATTCAGTGAATCATCGAATCTTTGAACGCACATTGCGCCCATTAGTATTCTAG

TGGGCATGCCTGTTCGAGCGTCATTTCGACCCTTAAGCC-CTGTTGCTTAGTGTTGGGAG

CCTACGT---CCCTGTAGCTCCTCAAAGTCAGTGGCGGAGTCGGGTCACACTCTAAGCGT

AGTAATCTTT-TCTCGCTTCTGCAGTTGTCCCGGTTCCTGCCGTAAAACCCCTAA-TTTA

TCTAGGTTGACCTCGGATCAGGTAGGAATACCCGCTGAACTTAA

>Entosordaria_perfidiosa_CBS_142773

TCATTATAGAGTTACAAAAACTCCAAACCCATGTGAACATACCAATTGTTGCCTCGGCAG

GTTGTGCCTACCCTGTAGAACCTACCCTGTAG-ACCTACCTGGTGGC-------------

---------------------------------GCGCCAGAAGGCCTGCCGAAGGACCCC

CAAACTCTATTTTATAATGGAATTCTGAATACGCAACTTAATAAGTTAAAACTTTCAACA

ACGGATCTCTTGGTTCTGGCATCGATGAAGAACGCAGCGAAATGCGATAAGTAATGTGAA

TTGCAGAATTCAGTGAATCATCGAATCTTTGAACGCACATTGCGCCCATTAGTACTCTAG

TGGGCATGCCTGTTCGAGCGTCATTTCGACCCTTAAGCC-CTGTTGCTTAGTGTTGGGAG

CCTACCT---CCCTGTAGCTCCTCAAAGTTAGTGGCGGAGTCGGGTCGTACTCTAAGCGT

AGTAGTTTCT-TCTCGCTTCTGCAGTCGTCCTGGTTCCTGCCGTAAAACCCCTAA-TTTA

TCTAGGTTGACCTCGGATCAGGTAGGAATACCCGCTGAACTTAA

>Entosordaria_quercina_CBS_142774

TCATTATAGAGTTACAAAAACTCCAAACCCATGTGAACATACCAATTGTTGCCTCGGCAG

GCTGTGTCTACCCTGTAGGACCTACCCTGTAG-CCCTACCTGGTGGC-------------

---------------------------------ACGCCAAAAGGCCTGCCGAAGGACCCC

CAAACTCTATTATATAGTGGAACTCTGAATACGCAACTTAATAAGTTAAAACTTTCAACA

ACGGATCTCTTGGTTCTGGCATCGATGAAGAACGCAGCGAAATGCGATAAGTAATGTGAA

TTGCAGAATTCAGTGAATCATCGAATCTTTGAACGCACATTGCGCCCATTAGTATTCTAG

TGGGCATGCCTGTTCGAGCGTCATTTCGACCCTTAAGCC-CTGTTGCTTAGTGTTGGGAG

CCTACCT---CCCTGTAGCTCCTCAAAGTTAGTGGCGGAGTCGGGTCGTACTCTAAGCGT

AGTAATCTTT-TCTCGCTTCTGCAGTCGTCCTGGTTCCCGCCGTAAAACCCCTAA-TTTA

TCTAGGTTGACCTCGGATCAGGTAGGAATACCCGCTGAACTTAA

>Xylaria_apoda_HAST_90080804

TCATTAAAGAGTTTTACA-ACTCCAAACCC-TGTGAACATACCCTACGTTGCCTCGGCAG

GT-GCACCTACCCTGTAGGCCTTACCCTGTAA-ACCTACCCGGTAG--------------

---------------------------------ATGC-GGTCAGCCTGCCGGTGGCCCCT

ATAACTCTGTTTGGCATTGTATTTCTGAATATAGAATTAAATAAGTTAAAACTTTCAACA

ACGGATCTCTTGGTTCTGGCATCGATGAAGAACGCAGCGAAATGCGATAAGTAATGTGAA

TTGCAGAATTCAGTGAATCATCGAATCTTTGAACGCACATTGCGCCCATTAGTATTCTAG

TGGGCATGCCTGTTCGAGCGTCATTTCAACCCTTAAGCCTCTGTTGCTTAGTGTTGGGGG

CCTACTT---CGCTGTAGCCCCTGAAAGTTAGTGGCGGAGCCGGCTCATGCTCCAGACGT

AGTAATGTTTATCTCGCCTGT-AGCTGTGCCGGTCCCCTGCCGTAAAACACCCCATTTTT

TAAAGGTTGACCTCGGATCAGGTAGGAATACCCGCTGAACTT--

>Nemania_primolutea_HAST_91102001

TCATTAAAGAGTTTTACAACTCCCAAACCCCTGTGAACATACCTTCTGTTGCCTCGGCAG

GCCTCGCCTACCCTCTAGCCCCTACACCGTAG--CCTAGCCGGGTG--------------

---------------------------------GTGCG-CGGACCCTGCCGGCGGCCCGC

GAAACTCTGTTTAGCACTGAATCTCTGAACATATAACTAAATAAGTTAAAACTTTCAACA

ACGGATCTCTTGGTTCTGGCATCGATGAAGAACGCAGCGAAATGCGATAAGTAATGTGAA

TTGCAGAATTCAGTGAATCATCGAATCTTTGAACGCACATTGCGCCCATTAGTATTCTAG

TGGGCATGCCTGTTCGAGCGTCATTTCAACCCTTAAGCCCCTGTTGCTTAGCGTTGGGAG

CCTACGG---CAGCGTAGCTCCCCAAAGTTAGTGGCGTGGTCGGTTCACACTCCAGACGT

AGTAGATTTCGTCTCGCCTGT-AGTTGGACCGGTCCCCTGCCGTAAAACACCCCAATTCT

AAAAGGTTGACCTCGGATCAGGTAGGAATACCCGCTGAACTT--

>Nemania_uda_CBS_148422

TCATTACAGAGTTCCAAAACTCCCAAACCCATGTGAACATACCTCGCGTTGCCTCGGCAG

GGGGCGCCTACCCCGGAGCACCTACCCTGTAG-CCTTACCCGGTG---------------

--------------------------------------GGCGACCCTGCCGACGGCCCCC

GAAACTCTGTTTAGCATTGGATTTCTGAAAACATAACTAAATAAGTTAAAACTTTCAACA

ACGGATCTCTTGGTTCTGGCATCGATGAAGAACGCAGCGAAATGCGATAAGTAATGTGAA

TTGCAGAATTCAGTGAATCATCGAATCTTTGAACGCACATTGCGCCCACTAGTATTCTGG

TGGGCATGCCTGTTCGAGCGTCATTTCAACCCTTAAGCCCTCGTTGCTTAGCGTTGGGAG

ACTACTGGAGTCCTGTAGCTCCCTAAAGTCAGTGGCGGAGCCGGTTCGCACTCCAGACGT

AGTAGCTTACACATCGCCTGTAGCCCGGCCAGGTCCCCTGCCATAAAACACCCCATTTCT

AAAAGGTTGACCTCGGATCAGGTAGGAATACCCGCTGAACTTAA

>Nemania_ethancrensonii_CBS_148337

TCATTAACGAGTTTCAAAACTCCCAAACCCATGTGAACATACCCTGCGTTGCCTCGGCAG

G-CGCGCCCCTCTCGGGCCCCT--------------------------------------

------------------------------------------ACCCTGCCGGAGGCCATC

ACAACTCTGTATAGTATC--ATTTCTGAAATCATAACTAAATAAGTTAAAACTTTCAACA

ACGGATCTCTTGGTTCTGGCATCGATGAAGAACGCAGCGAAATGCGATAAGTAATGTGAA

TTGCAGAATTCAGTGAATCATCGAATCTTTGAACGCACATTGCGCCCACTAGTATTCTGG

TGGGCATGCCTGTTCGAGCGTCATTTCAACCCTTAAGCCCCCGTTGCTTAGCGTTGGGAG

CCTGCCTAGGCCCGGCAGCCCCCTAAAGTCATCGGCGGAGCCGGTTTGCACTCCAGACGT

AGTAGCTCACACATCGCCTGTAGAGCGGCCCGGTCCCCTGCCGTAAAACACCCCATATCC

AAAAGGTTGACCTCGGATCAGGTAGGAATACCCGCTGAACTTAA

>Clypeosphaeria_mamillana_CBS_140735

TCATTATAGAGTATAAAAACTCCTAAACCCCTGTGAACATACCTATTGTTGCCTTGGCGG

CGCCC--------------------CTC--------------------------------

--------------------------------------GCGGGGACCGCCGAAGGCCCCC

TAAACTCTGTTTTACTTTGCAACTCTGAATCTAAAAACAAATAAGTTAAAACTTTCAACA

ACGGATCTCTTGGTTCTGGCATCGATGAAGAACGCAGCGAAATGCGATAAGTAATGTGAA

TTGCAGAATTCAGTGAATCATCGAATCTTTGAACGCACATTGCGCCCATTAGTATTCTAG

TGGGCATGCCTGTTCGAGCGTCATTAATACCATTAAGCC-CCGTAGCTTAGCGTTGGGAG

CCTGCTGGCGGCCTGCAGCTCCTCAAAAATAGTGGCGGAGTTAGCGTATACTCTAGGCGT

AGTAATTTTTCACTCGCTTCTGTG-TTGCGCTGGCGCCTGCCGTGAAACAGCCCAATTTT

AAAGGTTTGACCTCGGATCAGGTAGGAATACCCGCTGAACTTAA

>Digitodochium_amoenum_CBS_147285

TCATTAGAGAGTATAATAACTCCCAAACCCATGTGAACATACCTCTCGTTGCCTCGGCGG

AGAGGGACTACCCCAAAGTGACTACCCGGAGGCGCCTACCCGTTAG--------------

---------------------------------GCGTCGCTCCCGCCGCCGCTGGCCCAC

GAAACTCTGTTTTATATTGCAACTCTGAATCTTAAAACAAATAAGTTAAAACTTTCAACA

ACGGATCTCTTGGTTCTGGCATCGATGAAGAACGCAGCGAAATGCGATAAGTAATGTGAA

TTGCAGAATTCAGTGAATCATCGAATCTTTGAACGCACATTGCGCCCATTAGTATTCTAG

TGGGCATGCCTGTTCGAGCGTCATTTCGACCATTAAGCC-CTGTTGCTTAGCGTTGGGAG

CCTGCTGGC-TCCTGCAGCTCCTCAAAGTTAGTGGCGGAGTTAGTACATACTCTAGGCGT

AGTAATTTTCTTCTCGCCTCTGTGGTTGTGCTGGCGCCTGCCGTAAAACACCCAAAATTT

AACTGGTTGACCTCGGATCAGGTAGGAATACCCGCTGAACTTAA

>Occultitheca_rosae_HKAS_102393

TCATTAGAGAGTATAACAGCTCCCAAACCCATGTGAACATACCTCTCGTTGCCTCGGCGG

AGAGGGTCCATCCTGGAGACACTACCTCGAGG-GGCTACCCGGGAG--------------

---------------------------------CCACTGACCTCGCCGCCGCCGGCCCGC

GAAACTCTGTTT-ATTTTGCAACTCTGAATCTTCAAACAAATAAGTTAAAACTTTCAACA

ACGGATCTCTTGGTTCTGGCATCGATGAAGAACGCAGCGAAATGCGATAAGTAATGTGAA

TTGCAGAATTCAGTGAATCATCGAATCTTTGAACGCACATTGCGCCCATTAGTATTCTAG

TGGGCATGCCTGTTCGAGCGTCATTTCAACCCTTAAGCC-CTGTTGCTTAGCGTTGGGAG

CCAGCTGGCCGCCTGCAGCTCCTCAAAGTTAGTGGCGGAGTTAGTGTATACTCTAGGCGT

AGTAATTATTATCTCGCTTTTGTGGCTACGCTGGCGCCTGCCGTAAAACACCCAACTTCT

AAATGGTTGACCTCGGATCAGGTAGGAATACCCGCTGAACTTAA

>Magnostiolata_mucida_MFLU_19_2133

TCATTACAGAGTGTAAAAACTCCCAAACCCCTGTGAACCTACCTACTGTTGCCTCGGCAG

GCAGAGGCTACCCGG-------TACTTC-CGG----------------------------

---------------------------------GTGTGCCCGAGCCTGCCGCGGGCCCCT

GAAACTCTGTTT-ATTGTGGAATTCTGAATTGCAAAACAAACAAGTTAAAACTTTCAACA

ACGGATCTCTTGGTTCTGGCATCGATGAAGAACGCAGCGAAATGCGATAAGTAATGTGAA

TTGCAGAATTCAGTGAATCATCGAATCTTTGAACGCACATTGCGCCCATTAGTATTCTAG

TGGGCATGCCTGTTCGAGCGTCATTTCAACCCTTAAGCC-CTGTTGCTTAGTGTTGGGAG

CCTACTGGCCGCCTGTAGCTCCTCAAAGTTAGTGGCGGAGTTAGCACTCACTCTAGGCGT

AGTAATCACTATCTCGCCTCTGTGGTGGTGCTGGCCCCTGCCGTAAAACCCCCCAATTTT

CAATGGTTGACCTCGGATCAGGTAGGAATACCCGCTGAACTTAA

>Anthostomelloides_krabiensis_MFLUCC_15_0678

TCATTATAGAGTTTAAAAAACTCCCAACCCATGTGAACCTACC-TCTGTTGCCTCGGCGG

ACC-------CCCCG---------------------------------------------

----------------------------------------GGGGCCGCCAGAGGACCCGT

CAAACTCTGTAT-TTTGTGGAACTCTGAATCTCAAACTTAAT-AGTTAAAACTTTCAACA

ACGGATCTCTTGGTTCTGGCATCGATGAAGAACGCAGCGAAATGCGATAAGTAATGTGAA

TTGCAGAATTCAGTGAATCATCGAATCTTTGAACGCACATTGCGCCCATTAGTATTCTAT

TGGGCATGCCTGTTCGAGCGTCATTTCAACCCTCAAGCC-CTGTCGCTTGGCGTTGGGAG

CCCGCGG---AGACGCGGCTCCCCAAAGTTAGTGGCGGAGTCGGCACGGACTCTAGGCGT

AGTAATTACCACCTCGCCTCTGAAGCTGGCCGGCGCCCTGCCGTAAAACACCCCAATTCT

AAATGGTTGACCTCGGATCAGGTAGGAATACCCGCTGAACTTAA

>Linosporopsis_ischnotheca_CBS_145761

TCATTACAGAGCGTAAAAATCTCCCAACCCATGTGAACATACCTCTCGTTGCCTCGGCAG

GCCAC------CCAG---CGCCCACCCT----------CTTGGTGG--------------

---------------------------------CGGTGGTGCTCCCTGCCGCCGGCCCCC

CGAACTC-GTTTTATCTTGGAACTCTGAAT-AACAGTATAATACGTTAAAACTTTCAACA

ACGGATCTCTTGGCTCTGGCATCGATGAAGAACGCAGCGAAATGCGATAAGTAATGTGAA

TTGCAGAATCCAGTGAATCATCGAATCTTTGAACGCACATTGCGCCCACTAGTATTCTAG

TGGGCATGCCTGTTCGAGCGTCATTTCAACCCCTAAGCCCCAGCTGCTTCGTGTTGGGAG

CCTGCCGCGCCGCGGCAGCTCCTTAAAGTCAGTGGCGGAGTTGGCTCACACTCTAGGCGC

AGTAAATCTCTTCTCGCCTTTGCAGTTGGGCCATCGCCTGCCGTAAAACCCCCC--CATC

TAAAGGTTGACCTCGGATCAGGTAGGAATACCCGCTGAACTTAA

>Linosporopsis_ochracea_CBS_145999

TCATTACAGAGTGTAAAAA-CTCCCAACCCATGTGAATATACCTATCGTTGCCTCGGCAG

GTCCC------CCG-------------------ACCTACCCTGTAG--------------

---------------------------------TAAAGGTGCCACCTGCCGCTGGCCCGC

GAAACTCGTTTTA-TTTTGGAACTCTGAAT-AACTATACAATAAGTTAAAACTTTCAACA

ACGGATCTCTTGGTTCTGGCATCGATGAAGAACGCAGCGAAATGCGATAAGTAATGTGAA

TTGCAGAATTCAGTGAATCATCGAATCTTTGAACGCACATTGCGCCCATTAGTATTCTAG

TGGGCATGCCTGTTCGAGCGTCATTTCGACCCTGAAGCCCTGGTTGCTTCGCGTTGGGAG

CCTGCCGCGCCGCGGCAGCTCCTCAAAGTTAGTGGCGGAGTCGGTTCTCACTCTAGGCGT

AGTAA--TTTTTCTCGCCTCTGTAGTTTGACCGTCGCCTGCCGTAAAATCCCCC-ATTTC

TAAAGGTTGACCTCGGATCAGGTAGGAATACCCGCTGAACTTAA

>Emarcea_castanopsidicola_CBS_117105

TCATTATAGAGTTTTACAACTCCCAACCCTATGTGAACATACCTATCGTTGCCTCGGCGG

CGCGG-CCGGCCCTGAAGTGCTTACCCTGTAGGCGCTACCCGGTGGTCA-----------

---------------------------------GGGTAGGCCCGCCCGCCGGCGGCCCAC

GAAACTCTGTTTAGCA--GTTCTTCTGAATCGTAAACTTAATAAGTTAAAACTTTCAACA

ACGGATCTCTTGGTTCTGGCATCGATGAAGAACGCAGCGAAATGCGATAAGTAATGTGAA

TTGCAGAATTCAGTGAATCATCGAATCTTTGAACGCACATTGCGCCCATTAGTATTCTAG

TGGGCATGCCTGTTCGAGCGTCATTTCAACCCCTCAAGCCCTGTTGCTTGGTGCTGGGAG

CCTACAGCCCCGCTGTAGCTCCTCAAAGTTAGTGGCGGAGTCGGTACATACTCTAGGCGT

AGTAATT-CTATCTCGTCTCTGTGGTTGTGCTGTCCCCTGCCGTAAAACCCC--AACTTT

ATAGGTTTGACCTCGGATCAGGTAGGAATACCCGCTGAACTTAA

>Emarcea_eucalyptigena_CBS_139908

TCATTATAGAGTTTTACAACTCCCAACCCTATGTGAACATACCTATCGTTGCCTCGGCGG

CGCGG-CCGGCCCTGAAGCGCTTACCCTGTAGGCGCTACCCGGTGGTTA-----------

---------------------------------GGGTAGGCCCGCCCGCCGGCGGCCCAC

GAAACTCTGTTTAGCA--GTTCTTCTGAATCGTAAACTTAATAAGTTAAAACTTTCAACA

ACGGATCTCTTGGTTCTGGCATCGATGAAGAACGCAGCGAAATGCGATAAGTAATGTGAA

TTGCAGAATTCAGTGAATCATCGAATCTTTGAACGCACATTGCGCCCATTAGTATTCTAG

TGGGCATGCCTGTTCGAGCGTCATTTCAACCCCTCAAGCCCTGTTGCTTGGTGCTGGGAG

CCTACAGCCCCGCTGTAGCTCCTCAAAGTTAGTGGCGGAGTCAGTACACACTCTAGGCGT

AGTAATT-CTATCTCGCCTCTGCGGTTGTGCTGTCCCCTGCCGTAAAACCCC--AACTTT

ATAGGTTTGACCTCGGATCAGGTAGGAATACCCGCTGAACTTAA

>Kretzschmaria_deusta_CBS_163.93

TCATTAAAGAGTTTTACAACTCCTAAACCCATGTGAACTTACCTTCTGTTGCCTCGGCAG

GCAGGTGCTACCCTGTAGACCCTATCCTATAG-ACCTACCCGGTAGAG------------

---------------------------------ATGCGGTGCCACCTGCCGGTGGACTGC

-AAATTCTGTTTAGTAATGGTATTCTGAATCATTATATGGTTAAGTTAAAACTTTCAACA

ACGGATCTCTTGGTTCTGGCATCGATGAAGAACGCAGCGAAATGCGATAAGTAATGTGAA

TTGCAGAATTTAGTGAATCATCGAATCTTTGAACGCACATTGCGCCCATTAGTATTCTAG

TGGGCATGCCTGTTCGAGCGTCATTTCAACCCTTAAGCCTCTGTTGCTTAGTGTTGGGAG

CCTACCGCATTGCTGTAGCTCCTTAAAGTTAGTGGCGGAGTCGGTTCACACTCTAGGCGT

AGTAAAGATATTCTCGCCTGTGAGATGAGCCGGTCCCCTGCCGTAAAACACCCCTTTTTT

ATAAGGTTGACCTCGGATCAGGTAGGAATACCCGCTGAACTTAA

>Collodiscula_japonica_CBS_124266

TCATTAGAGAGTC-TACAACTCCGTAACCCATGTGAACATACCTCCCGTTGCCTCCGCAG

GCCTCGCCGACTCTGCGG----CGTCCT----------CCAGTCGG--------------

---------------------------------AGGCGGCCCCGGACAGGGGCGTCCACC

CTAACTCTGAATCGTACTG-AGCTCTGA-AACATGACAAAATAAGTTAAAACTTTCAACA

ACGGATCTCTTGGTTCTGGCATCGATGAAGAACGCAGCGAAATGCGATAAGTAATGTGAA

TTGCAGAATTCAGTGAATCATCGAATCTTTGAACGCACATTGCGCCCATTAGTATTCTAT

TGGGCATGCCTGTTCGAGCGTCATTTCAACCCTCAAGCC-CTGTTGCTTGGTGTTGGGAG

CCTACGG---CGACGTAGCTCCTCAAAGTCAGTGGCGGAGTCGGTTCACACTCTAGACGT

AGTAATCTCTATCTCGCCTGTGAGTTGGACTGGTGCCCTGCCGTGAAACCCCCCAATTTC

TAAAGGTTGACCTCGGATCAGGTAGGAATACCCGCTGAACTTAA

>Daldinia_concentrica_CBS_113277

------CCGAGTTTCTAA-ACTCCAACCCTTTGTGAACTTACC-GTCGTTGCCTCGGCGG

GCTGCGCTTACCCTGTAG----TACCCTGTAG---CTACCCGGTAG--------------

---------------------------------GCGCGTCCAAGCCCGCCGGTGGACCAC

TAAACTCTGTTTAATACCGAATCTCTGAATCTTCAACTTAGTAAGTTAAAACTTTCAACA

ACGGATCTCTTGGTTCTGGCATCGATGAAGAACGCAGCGAAATGCGATAAGTAATGTGAA

TTGCAGAATTCAGTGAATCATCGAATCTTTGAACGCACATTGCGCCCATTAGTATTCTAG

TGGGCATGCCTATTCGAGCGTCATTTCAACCCTTAAGCCTTAGTTGCTTAGCGTTGGGAG

TCTGCGCTGTCGGCGCAGTTCCTCAAAGTGATTGGCGGAGTTAGGGCATACTCTAAGCGT

AGTAATATTCTTCTCGCTTCTGTAGTTGCCTGGCGGCTTGCCGTTAAACCCCTN------

--------------------------------------------

>Entonaema_liquescens_ATCC_46302

TCATTACTGAGTTTCTAA-ACTCCAACCCTATGTGAACCTACC-GTCGTTGCCTCGGCGG

GCTGCGCTTACCCGGTAG----TACCCTGTAG---CTACCCGGTAGC-------------

---------------------------------GCGTTACCAAGCCCGCCGGTGGACCAC

TAAACTCTGTTAAATACTGTATCTCTGAATCTTCAAACTTAATAGTTAAAACTTTCAACA

ACGGATCTCTTGGTTCTGGCATCGATGAAGAACGCAGCGAAATGCGATAAGTAATGTGAA

TTGCAGAATTCAGTGAATCATCGAATCTTTGAACGCACATTGCGCCCATTAGTATTCTAG

TGGGCATGCCTATTCGAGCGTCATTTCAACCCTTAAGCCCCTGTTGCTTAGTGTTGGGAA

TCTGCGTC--GGGCGCAGTTCCTTAAAGTGATTGGCGGAGTTAGGGCATACTCTGAGCGT

AGTAATATTCTTCTCGCTTCTGAAGTTGCCTGGCGGCCGGCCGTAAAACCCCTATATCTT

TAGTGGTTGACCTCGAATTAGGTAGGAATACCCGCTGAACTTAA

>Ruwenzoria_pseudoannulata_MUCL_51394

TCATTACCGAGTTTCTAAAACTCCAACCCTATGTGAACTTACC-GTCGTTGCCTCGGCGG

GCTGCGCTTGCCCGGTAG----TGCCCTGCGGCGGCTACCCGGTGTAGC-----------

---------------------------------GCGCGACAAGGCCCGCCGGTGGACCAC

TAAACTCTGTTAAATACTGTATCTCTGAATTTCAAACTTAATAAGTTAAAACTTTCAACA

ACGGATCTCTTGGTTCTGGCATCGATGAAGAACGCAGCGAAATGCGATAAGTAATGTGAA

TTGCAGAATTCAGTGAATCATCGAATCTTTGAACGCACATTGCGCCCATTAGTATTCTAA

TGGGCATGCCTATTCGAGCGTCATTTCAACCCTTAAGCCCCTGTTGCTTAGTGTTGGGAA

TCTGCGTC--GGGCGCAGTTCCTCAAAGTAATCGGCGGAGTTGGGGCTTACTCTGAGCGT

AGTAGTATCTTTCTCGCTTCTGTGGTAGCCTGGCGGCCGGCCGTAAAACCCCTATATCTT

TAGTGGTTGACCTCGAATTAGGTAGGAATACCCGCTGAACTTAA

>Hypomontagnella_monticulosa_MUCL_54604

TCATTACTGAGTTTCAAAACTCCCAACCCTTTGTGAACTTACC-ACTGTTGCCTCGGCGA

GTTGT-GCTACCCTATAG----TACCCTGTAG---CTACCCGGGAAC-------------

---------------------------------ACATT--CAAGCTCGCCAGAGGACCTA

CCAACTCTGTTT-ATACTGTATCTCTGAACTTATAACTAAATAAGTTAAAACTTTCAACA

ACGGATCTCTTGGTTCTGGCATCGATGAAGAACGCAGCGAAATGCGATAAGTAATGTGAA

TTGCAGAATTCAGTGAATCATCGAATCTTTGAACGCACATTGCGCCCATTAGTATTCTAG

TGGGCATGCCTATTCGAGCGTCATTTCAACCCTTAAGCCTCAGTTGCTTAGTATTGGGAC

TCTACGACCTTAGCGTAGTTCCTTAAAGGTAGTGGCGGAGTTATAGCACACTCTAAGCGT

AGTAATTCT--TCTCGCTTCTGTAGTGGTATAGTTGCTAGCCATAAAACACCCCCTATTT

TAATGGTTGACCTCGGATTAGGTAGGAATACCCGCTGAACTTAA

>Jackrogersella_multiformis_CBS_119016

-------------------CTCCAAACCCTTTGTGAACCTACCTCCAGTTGCCTCGGCGC

TCTGCGGCTACCCCGTAG----CGCCCTGTAGCGGCTACCCTGTAG--------------

---------------------------------CCGGCTCACGGCCCGCCGAAGGACCCC

TAAACTCTGTTTAATAGTGTATTTCTGAATCCAAACAAAAATAAGTTAAAACTTTCAACA

ACGGATCTCTTGGTTCTGGCATCGATGAAGAACGCAGCGAAATGCGATAAGTAATGTGAA

TTGCAGAATTCAGTGAATCATCGAATCTTTGAACGCACATTGCGCCCATTAGTATTCTAG

TGGGCATGCCTATTCGAGCGTCATTTCGACCATTAAGCC-CTGTAGCTTAGCGTTGGGAG

CCTACGG---TAGCGTAGCTCCTCAAAGTCAGTGGCGGAGTTATGGCGTACTCTGAGCGT

AGTAAATCTTTTCTCGCTTCTGTAGTCGCCTAGCTGCCTGCN------------------

--------------------------------------------

>Spiririma_gaudefroyi_CBS_147284

TCATTACAGAGTTTCTAAACTCCCAACCCTTTGTGAACTTACC-TTTGTTGCTTCGGCGG

CGGAGGCT-ACCCTGTAGGGGCCACCAG-TATTGGTTACCCGGTAGT----GGAACTGAT

TCCATCCCAGCGTTATTTCGTAATGGGTCAGCTCTGGGTGTCTTCCCGCCGGCGGCCAAC

TAAACTCTGTCT-TTTTTAGAATTCTGAATCGTAAACTTAATAAGTTAAAACTTTCAACA

ACGGATCTCTTGGTTCTGGCATCGATGAAGAACGCAGCGAAATGCGATAAGTAATGTGAA

TTGCAGAATTCAGTGAATCATCGAATCTTTGAACGCACATTGCGCCCATTAGCATTCTAG

TGGGCATGCCTGTTCGAGCGTCATTTCACCACTTAAGCC-CTGTTGCTTAGCGTTGGGGG

CCTACGGCGCGCCTATAGCCCCTTGAAGCGATTGGCGGAGTTGGTCCTCACTCTAAGCGT

AGTAATT-CTTTCTCGCTTCTGTAGTGGTGCCGGCCCCTGCCGTAAAACCTTACAGTATA

CAATGGTTGACCTCGGATCAGGTAGGAATACCCGCTGAACTTAA

>Muscodor_thailandica_MFLUCC_17_2669

-CATTACAGAGTTTCTAAACTCCCAACCCTATGTGAACTTACC-TTTGTTGCTTCGGCGG

CGGAGGCT-ACCCTGCGGAGAATACCACTTAGTGGTTACCCTGTAGTTTCAGGTAC----

--------------------------ATCAGCTCGGTAGTCATCCCCGCCGGCGGCCAAC

TAAACTCTGTTT-TCTTTGGAATTCTGAATAACAAACTTAATAAGTTAAAACTTTCAACA

ACGGATCTCTTGGTTCTGGCATCGATGAAGAACGCAGCGAAATGCGATAAGTAATGTGAA

TTGCAGAATTCAGTGAATCATCGAATCTTTGAACGCACATTGCGCCCATTAGCATTCTAG

TGGGCATGCCTGTTCGAGCGTCATTTCACCACTTAAGCC-TTGTTGCTTAGCGTTGGGAG

CCTACGGCACGCCCGTAGCTCCTTAAAGTGATTGGCGGAGTTGGTTCTCACTCTAAGCGT

AGTAATT-ATATCTCGCTTCTGTAGTGGTCCCGGCCCCTGCCGTAAAACCCCTTA---TA

CAAAGGTTGACCTCGGATCAGGTAGGAATACCCGCTGAACTTAA

>Muscodor_ziziphi_MFLUCC_17_2662

-CATTACAGAGTTTCTAAACTCCCAACCCTATGTGAACTTACC-TTTGTTGCTTCGGCGG

CGGAGGCT-ACCCTGCGGAGAATACCACTTAGTGGTTACCCTGTAGTTTCAGGTAC----

--------------------------ATCAGCTCGGTAGTCATCCCCGCCGGCGGCCAAC

TAAACTCTGTTT-TCTTTGGAATTCTGAATAACAAACTTAATAAGTTAAAACTTTCAACA

ACGGATCTCTTGGTTCTGGCATCGATGAAGAACGCAGCGAAATGCGATAAGTAATGTGAA

TTGCAGAATTCAGTGAATCATCGAATCTTTGAACGCACATTGCGCCCATTAGCATTCTAG

TGGGCATGCCTGTTCGAGCGTCATTTCACCACTTAAGCC-TTGTTGCTTAGCGTTGGGAG

CCTACGGCACGCCCGTAGCTCCTTAAAGTGATTGGCGGAGTTGGTTCTCACTCTAAGCGT

AGTAATT-ATATCTCGCTTCTGTAGTGGTCCCGGCCCCTGCCGTAAAACCCCTTA---TA

CAAAGGTTGACCTCGGATCAGGTAGGAATACCCGCTGAACTTAA

>Muscodor_equiseti_JCM_18233

TCATTACAGAGTTTCTAAACTCCCAACCCTATGTGAACTTACC-TTTGTTGCTTCGGCGG

CGGAGGCT-ACCCTGCGGAGAATACCACTTAGTGGTTACCCTGTAGTTTCAGGTAC----

--------------------------ATCAGCTCGGTAGTCATCCCCGCCGGCGGCCAAC

TAAACTCTGTTT-TCTTTGGAATTCTGAATAACAAACTTAATAAGTTAAAACTTTCAACA

ACGGATCTCTTGGTTCTGGCATCGATGAAGAACGCAGCGAAATGCGATAAGTAATGTGAA

TTGCAGAATTCAGTGAATCATCGAATCTTTGAACGCACATTGCGCCCATTAGCATTCTAG

TGGGCATGCCTGTTCGAGCGTCATTTCACCACTTAAGCC-TTGTTGCTTAGCGTTGGGAG

CCTACGGCACGCCCGTAGCTCCTTAAAGTGATTGGCGGAGTTGGTTCTCACTCTAAGCGT

AGTAATT-ATATCTCGCTTCTGTAGTGGTCCCGGCCCCTGCCGTAAAACCCCTTA---TA

CAAAGGTTGACCTCGGATCAGGTAGGAATACCCGCTGAACTTAA

>Muscodor_vitigena_MONT_P_15

TCATTACAGAGTTTCTAAACTCCCAACCCTATGTGAACTTACC-TTTGTTGCTTCGGCGG

CGGAGGCT-ACCCTGCGGAGAATACCACTTAGTGGTTACCCTGTAGTTTCAGGTAC----

--------------------------ATCAGCTCGGTAGTCATCCCCGCCGGCGGCCAAC

TAAACTCTGTTT-TCTTTGGAATTCTGAATAACAAACTTAATAAGTTAAAACTTTCAACA

ACGGATCTCTTGGTTCTGGCATCGATGAAGAACGCAGCGAAATGCGATAAGTAATGTGAA

TTGCAGAATTCAGTGAATCATCGAATCTTTGAACGCACATTGCGCCCATTAGCATTCTAG

TGGGCATGCCTGTTCGAGCGTCATTTCACCACTTAAGCC-TTGTTGCTTAGCGTTGGGAG

CCTACGGCACGCCCGTAGCTCCTTAAAGTGATTGGCGGAGTTGGTTCTCACTCTAAGCGT

AGTAATT-ATATCTCGCTTCTGTAGTGGTCCCGGCCCCTGCCGTAAAACCCCTTA---TA

CAAAGGTTGACCTCGGATCAGGTAGGAATACCCGCTGAACTTAA

>Muscodor_suturae_MSUB_2380

-------AGAGTTTCTAA-CTCCCAACCCTATGTGAACTTACC-TTTGTTGCTTCGGCGG

CGGAGGCT-ACCCTGCGGAGAATACCACTTAGTGGTTACCCTGTAGTTTCAGGTAC----

--------------------------ATCAGCTCGGTAGTCATCCCCGCCGGCGGCCAAC

TAAACTCTGTTT-TCTTTGGAATTCTGAATAACAAACTTAATAAGTTAAAACTTTCAACA

ACGGATCTCTTGGTTCTGGCATCGATGAAGAACGCAGCGAAATGCGATAAGTAATGTGAA

TTGCAGAATTCAGTGAATCATCGAATCTTTGAACGCACATTGCGCCCATTAGCATTCTAG

TGGGCATGCCTGTTCGAGCGTCATTTCACCACTTAAGCC-TTGTTGCTTAGCGTTGGGAG

CCTACGGCACGCCCGTAGCTCCTTAAAGTGATTGGCGGAGTTGGTTCTCACTCTAAGCGT

AGTAATT-ATATCTCGCTTCTGTAGTGGTCCCGGCCCCTGCCGTAAAACCCCTTA---TA

CAAAGGTTGACCTCGGATCAGGTAGGAATACCCGCTGAACTTAA

>Muscodor_coffeana_COAD_1842

TCATTACAGAGTTTCTAAACTCCCAACCCTATGTGAACTTACC-TTTGTTGCTTCGGCGG

CGGAGGCT-ACCCTGCGGGGATTACCACTTAGTGATTACCCTGCAGTCCCAGGTAC----

--------------------------ATTTGTTCGGTAGTCATCCCCGCCGGCGGCCAAC

TAAACTCTGTTT-TCTTTGGAATTCTGAATCATAAACTTAATAAGTTAAAACTTTCAACA

ACGGATCTCTTGGTTCTGGCATCGATGAAGAACGCAGCGAAATGCGATAAGTAATGTGAA

TTGCAGAATTCAGTGAATCATCGAATCTTTGAACGCACATTGCGCCCATTAGCATTCTAG

TGGGCATGCCTGTTCGAGCGTCATTTCACCACTTAAGCC-CTGTTGCTTAGCGTTGGGAG

CCTACGGCATGCCCGTAGCTCCTTAAAGTGATTGGCGGAGTTGGTTCTCACTCTAAGCGT

AGTAACT-ATATCTCGCTTTTGTAGTGGTTCCGGCCCCTGCCGTAAAACCCCTTA---TA

TAAAGGTTGACCTCGGATCAGGTAGGAATACCCGCTGAACTTAA

>Muscodor_yucatanensis_MEXU_25511

TCATTACAGAGTTTCTAAACTCCCAACCCTATGTGAACTTACC-TTTGTTGCTTCGGCGG

CGGAGGCT-ACCCTGCGGGGATTACCACCTAGTGGTTACCCTGCAGTCTCAGGTAC----

--------------------------ATCTGTTCGGTAGTCATCCCCGCCGGCGGCCAAC

TAAACTCTGTTT-TCTTTGGAATTCTGAATCATAAACTTAATAAGTTAAAACTTTCAACA

ACGGATCTCTTGGTTCTGGCATCGATGAAGAACGCAGCGAAATGCGATAAGTAATGTGAA

TTGCAGAATTCAGTGAATCATCGAATCTTTGAACGCACATTGCGCCCATTAGCATTCTAG

TGGGCATGCCTGTTCGAGCGTCATTTCACCACTTAAGCC-CTGTTGCTTAGCGTTGGGAG

CCTACGGCATGCCCGTAGCTCCTTAAAGTGATTGGCGGAGTTGGTTCTCACTCTAAGCGT

AGTAACT-ATATCTCGCTTCTGTAGTAGTTCCGGCCCCTGCCGTAAAACCCCTTA---TA

CAAAGGTTGACCTCGGATCAGGTAGGAATACCCGCTGAACTTAA

>Muscodor_sp._SMH_1255

TCATTACAGAGTTTCTAAACTCCTAACCCTATGTGAACTTACC-TTTGTTGCTTCGGCGG

CGGAGGCT-ACCCTGCGGGGACTACCACCTAGTGGTTACCCTGCAGTCCTAGGTAC----

--------------------------AGTAG-TCGGTAGCCATCCCCGCCGGCGGCCAAC

TAAACTCTGTTT-TCTTTGGAATTCTGAATCGTAAACTTAATAAGTTAAAACTTTCAACA

ACGGATCTCTTGGTTCTGGCATCGATGAAGAACGCAGCGAAATGCGATAAGTAATGTGAA

TTGCAGAATTCAGTGAATCATCGAATCTTTGAACGCACATTGCGCCCATTAGCATTCTAG

TGGGCATGCCTGTTCGAGCGTCATTTCACCACTTAAGCC-CTGTTGCTTAGCGTTGGGGG

CCTACGGCATGCCTGTAGCCCCTTAAAGTGATCGGCGGAGTTGGTTCTCACTCTAAGCGT

AGTAACT-ATATCTCGCTTCTGCAGTGGTCCCGGTCCCTGCCGTAAAACTTACTA---AA

CAAAGGTTGACCTCGGATCAGGTAGGAATACCCGCTGAACTTAA

>Muscodor_brasiliensis_LGMF_1256

------------------------AACCCTATGTGAACTTACC-TTTGTTGCTTCGGCGG

CGGAGGCT-ACCCTATGGGG--TACCACCTAGTGGTTACCCTGTAGTCCCAGGTACTGGA

TCATGCCCAACGTCTTATCGTCTATGAACAGTTCGGTAGTCATCCCCGCCGGCGGCCAAC

TAAACTCTGTTT-TCTTTGGAATTCTGAATTATAAACTTAATAAGTTAAAACTTTCAACA

ACGGATCTCTTGGTTCTGGCATCGATGAAGAACGCAGCGAAATGCGATAAGTAATGTGAA

TTGCAGAATTCAGTGAATCATCGAATCTTTGAACGCACATTGCGCCCATTAGCATTCTAG

TGGGCATGCCTGTTCGAGCGTCATTTCACCACTTAAGCC-CTGTTGCTTAGCGTTGGGAG

CCTACGGCCTGCCCGTAGCTCCTTAAAGTGATTGGCGGAGTTGGTTCTCACTCTAAGCGT

AGTAACT-ATATCTCGCTTCTGTAGTGGTTCCGGCCCCTGCCGTAAAACCCCTTG---TA

CAAAGGTTGACCTCGGATCAGGTAGGAATACCCGCTGAACTTAA

>Muscodor_alba_9_6

------CAGAGTTTCCAAACTCCCAACCCTATGTGAACTTACC-TTTGTTGCTTCGGCGG

CGGAGGCT-ACCCTATAGGGGATACCACATAGTGGTTACCCTGTAGTCCCAGGTGCTAGA

TCGTGCTCAACGTCTTATCGTCTACGACTAGCTCGGTGGCCCTCCCCGCCGGCGGCCAAC

TAAACTCTGTTT--TTATGGCATTCTGAATTATAAACTTAATAAGTTAAAACTTTCAACA

ACGGATCTCTTGGTTCTGGCATCGATGAAGAACGCAGCGAAATGCGATAAGTAATGTGAA

TTGCAGAATTCAGTGAATCATCGAATCTTTGAACGCACATTGCGCCCATTAGCATTCTAG

TGGGCATGCCTGTTCGAGCGTCATTTCACCACTTAAGCC-CTGTTGCTTAGCGTTGGGAG

CCTACGGCACGCCCGTAGCTCCCTAAAGTGATTGGCGGAGTTGGTTCTCACTCTAGGCGT

AGTAAAT-CTATCTCGCCTCTGTAGTGGTTCCGGCCCCTGCCGTAAAACCCCCT--ATAT

CAAAGG--------------------------------------

>Muscodor_alba_MONT_620

TCATTACAGAGTTTCCAAACTCCCAACCCTATGTGAACTTACC-TTTGTTGCTTCGGCGG

CGGAGGCT-ACCCTATAGGGGATACCACATAGTGGTTACCCTGTAGTCCCAGGTGCTAGA

TCGTGCTCAACGTCTTATCGTCTACGACTAGCTCGGTGGCCCTCCCCGCCGGCGGCCAAC

TAAACTCTGTTT--TTATGGCATTCTGAATTATAAACTTAATAAGTTAAAACTTTCAACA

ACGGATCTCTTGGTTCTGGCATCGATGAAGAACGCAGCGAAATGCGATAAGTAATGTGAA

TTGCAGAATTCAGTGAATCATCGAATCTTTGAACGCACATTGCGCCCATTAGCATTCTAG

TGGGCATGCCTGTTCGAGCGTCATTTCACCACTTAAGCC-CTGTTGCTTAGCGTTGGGAG

CCTACGGCACGCCCGTAGCTCCCTAAAGTGATTGGCGGAGTTGGTTCTCACTCTAGGCGT

AGTAAAT-CTATCTCGCCTCTGTAGTGGTTCCGGCCCCTGCCGTAAAACCCCCT--ATAT

CAAAGGTTGACCTCGGATCAGGTAGGAATACCCGCTGAACTTAA

>Muscodor_crispans_MONT_2347

---------------------CCCAACCCTATGTGAACTTACC-TTTGTTGCTTCGGCGG

CGGAGGCT-ACCCTATAGGGGATACCACATAGTGGTTACCCTGTAGTCCCAGGTGCTAGA

TCGTGCTCAACGTCTTATCGTCTACGACTAGCTCGGTGGCCCTCCCCGCCGGCGGCCAAC

TAAACTCTGTTT--TTATGGCATTCTGAATTATAAACTTAATAAGTTAAAACTTTCAACA

ACGGATCTCTTGGTTCTGGCATCGATGAAGAACGCAGCGAAATGCGATAAGTAATGTGAA

TTGCAGAATTCAGTGAATCATCGAATCTTTGAACGCACATTGCGCCCATTAGCATTCTAG

TGGGCATGCCTGTTCGAGCGTCATTTCACCACTTAAGCC-CTGTTGCTTAGCGTTGGGAG

CCTACGGCACGCCCGTAGCTCCCTAAAGTGATTGGCGGAGTTGGTTCTCACTCTAGGCGT

AGTAAAT-CTATCTCGCCTCTGTAGTGGTTCCGGCCCCTGCCGTAAAACCCCCT--ATAT

CAAAGGTTGA----------------------------------

>Muscodor_musae_JCM_18230

-NATTACAGAGTTTCTAAACTCCCAACCCTATGTGAACTTACC-TTTGTTGCTTCGGCGG

CGGAGGCT-ACCCTATAGGGGATACCACATAGTGGTTACCCTGTAGTCCCAGGTGCTAGA

TCGTGCTCAACGTCTTATCGTCTACGACTAGCTCGGTGGCCCTCCCCGCCGGCGGCCAAC

TAAACTCTGTTT--TTATGGCATTCTGAATTATAAACTTAATAAGTTAAAACTTTCAACA

ACGGATCTCTTGGTTCTGGCATCGATGAAGAACGCAGCGAAATGCGATAAGTAATGTGAA

TTGCAGAATTCAGTGAATCATCGAATCTTTGAACGCACATTGCGCCCATTAGCATTCTAG

TGGGCATGCCTGTTCGAGCGTCATTTCACCACTTAAGCC-CTGTTGCTTAGCGTTGGGAG

CCTACGGCACGCCCGTAGCTCCCTAAAGTGATTGGCGGAGTTGGTTCTCACTCTAGGCGT

AGTAAAT-CTATCTCGCCTCTGTAGTGGTTCCGGCCCCTGCCGTAAAACCCCCT--ATAT

CAAAGGTTGACCTCGGATCAGGTAGGAATACCCGCTGAACTTAA

>Muscodor_oryzae_JCM_18231

TCATTACAGAGTTTCTAAACTCCCAACCCTATGTGAACTTACC-TTTGTTGCTTCGGCGG

CGGAGGCT-ACCCTATAGGGGATACCACATAGTGGTTACCCTGTAGTCCCAGGTGCTAGA

TCGTGCTCAACGTCTTATCGTCTACGACTAGCTCGGTGGCCCTCCCCGCCGGCGGCCAAC

TAAACTCTGTTT--TTATGGCATTCTGAATTATAAACTTAATAAGTTAAAACTTTCAACA

ACGGATCTCTTGGTTCTGGCATCGATGAAGAACGCAGCGAAATGCGATAAGTAATGTGAA

TTGCAGAATTCAGTGAATCATCGAATCTTTGAACGCACATTGCGCCCATTAGCATTCTAG

TGGGCATGCCTGTTCGAGCGTCATTTCACCACTTAAGCC-CTGTTGCTTAGCGTTGGGAG

CCTACGGCACGCCCGTAGCTCCCTAAAGTGATTGGCGGAGTTGGTTCTCACTCTAGGCGT

AGTAAAT-CTATCTCGCCTCTGTAGTGGTTCCGGCCCCTGCCGTAAAACCCCCT--ATAT

CAAAGGTTGACCTCGGATCAGGTAGAAACCCCCGGAAAATT---

>Muscodor_rosea_MONT_2098

TCATTACAGAGTTTCTAAACTCCCAACCCTATGTGAACTTACC-TTTGTTGCTTCGGCGG

CGGAGGCT-ACCCTATAGGGGATACCACATAGTGGTTACCCTGTAGTCCCAGATGCTAGA

TCGTGCTCAACGTCTTATCGTCTACGACTAGCTCGGTGGCCCTCCCCGCCGGCGGCCAAC

TAAACTCTGTTT--TTATGGCATTCTGAATTATAAACTTAATAAGTTAAAACTTTCAACA

ACGGATCTCTTGGTTCTGGCATCGATGAAGAACGCAGCGAAATGCGATAAGTAATGTGAA

TTGCAGAATTCAGTGAATCATCGAATCTTTGAACGCACATTGCGCCCATTAGCATTCTAG

TGGGCATGCCTGTTCGAGCGTCATTT-ACCACTTAAGCC-CTGTTGCTTAGCGTTGGGAG

CCTACGGCACGCCCGTAGCTCCCTAAAGTGATTGGCGGAGTTGGTTCTCACTCTAGGCGT

AGTAAAT-CTATCTCGCCTCTGTAGTGGTTCCGGCCCCTGCCGTAAAACCCCCT--ATAT

CAAAGGTTGACCTCGGATCAGGTAGGAATACCCGCTGAACTTAA

>Muscodor_kashayum_NFCCI_2947

----------------------------------------------------CTCGG---

-GGAGGCT-ACCCTATAGGGGATACCACATAGTGGTTACCCTGTAGTCCCAGGTGCTAGA

TCGTGCTCAACGTCTTATCGTCTACGACTAGCTCGGTGGCCCTCCACGCCGGCGGCCAAC

TAAACTCTGTTT--TTATGGCATTCTGAATTATAAACTTAATAAGTTAAAACTTTCAACA

ACGGATCTCTTGGTTCTGGCATCGATGAAGAACGCAGCGAAATGCGATAAGTAATGTGAA

TTGCAGAATTCAGTGAATCATCGAATCTTTGAACGCACATTGCGCCCATTAGCATTCTAG

TGGGCATGCCTGTTCGAGCGTCATTTCACCACTTAAGCC-CTGTTGCTTAGCGTTGGGAG

CCTACGGCACGCCCGTAGCTCCCTAAAGTGATTGGCGGAGTTGGTTCTCACTCTAGGCGT

AGTAAAT-CTATCTCGCCTCTGTAGTGGTTCCGGCCCCTGG-------------------

--------------------------------------------

>Muscodor_tigerensis_NFCCI_3172

-----------------------------------------------------------T

GGGAGGCT-ACCCTATAGGGGATACCACATAGTGGTTACCCTGTAGTCCCAGGTGCTAGA

TCGTGCTCAACGTCTTATCGTCTACGACTAGCTCGGTGGCCCTCCCCGCCGGCGGCCAAC

TAAACTCTGTTT--TTATGGCATTCTGAATTATAAACTTAATAAGTTAAAACTTTCAACA

ACGGATCTCTTGGTTCTGGCATCGATGAAGAACGCAGCGAAATGCGATAAGTAATGTGAA

TTGCAGAATTCAGTGAATCATCGAATCTTTGAACGCACATTGCGCCCATTAGCATTCTAG

TGGGCATGCCTGTTCGAG-----------CACTTAAGCC-CTGTTGCTTAGCGTTGGGAG

CCTACGGCACGCCCGTAGCTCCCTAAAGTGATTGGCGGAGTTGGTTCTCACTCTAGGCGT

AGTAAAT-CTATCTCGCCTCTGTAGTGGTTCCGGCCCCTA--------------------

--------------------------------------------

>Muscodor_cinnanomi_BCC_38842

TCATTACAGAGTTTCTAAACTCCCAACCCTATGTGAACTTACC-TTTGTTGCTTCGGCGG

CGGAGGCT-ACCCTATAGGGGATACCACATAGTGGTTACCCTGTAGTCCCAGGTGCTAGA

TCGTGCTCAACGTCTTATCGTCTACGACTAGCTCGGTGGCCCTCCCCGCCGGCGGCCAAC

TAAACTCTGTTT--TTATGGCATTCTGAATTATAAACTTAATAAGTTAAAACTTTCAACA

ACGGATCTCTTGGTTCTGGCATCGATGAAGAACGCAGCGAAATGCGATAAGTAATGTGAA

TTGCAGAATTCAGTGAATCATCGAATCTTTGAACGCACATTGCGCCCATTAGCATTCTAG

TGGGCATGCCTGTTCGAGCGTCATTTCACCACTTAAGCC-CTGTTGCTTAGCGTTGGGAG

CCTACGGCACGCCCGTAGCTCCCTAAAGTGATTGGCGGAGTTGGTTCTCACTCTAGGCGT

AGTAAAT-CTATCTCGCCTCTGTAGTGGTTCCGGCCCCCTGCCCAAACCCCCCT--ATAT

CAAAGGTTGACCTCGGATCAGGTAGGAATACCCGCTGAACTTAA

>Muscodor_camphorae_NFCCI_3236

-----------------------------------------------------------T

GGGAGGCT-ACCCTATAGGGGATACCACATAGTGGTTACCCTGTAGTCCCAGGTGCTAGA

TCGTGCTCAACGTCTTATCGTCTACGACTAGCTCGGTGGCCCTCCCCGCCGGCGGCCAAC

TAAACTCTGTTT--TTATGGCATTGGGAGTGATAATCTAAATAATTTAAAACTTTCAACA

ACGGATCTCTGGGTTCTGGCATCGATGAAGAACGCAGCGAAATGCGATAAGTAATGTGAA

TTGCAGAATTCAGTGAATCATCGAATCTTTGAACGCACATTGCGCCCATTAGCATTCTAG

TGGGCATGCCTGTTCGAGCGTCATTTCACCACTTAAGCC-CTGTTGCTTAGCGTTGGGAG

CCTACGGCACGCCCGTAGCTCCCTAAAGTGATTGGCGGAGTTGGTTCTCACTCTAGGCGT

AGTAAAT-CTATCTCGCCTCTGTAGTGGTTCCGGCCCCTAA-------------------

--------------------------------------------

>Muscodor_ghoomensis_NFCCI_3234

---------------------------------------------------------TTC

GGGAGGCT-TCCCTATAGGGGATACCACATAGTGGTTACCCTGTAGTCCCAGGTGCTAGA

TCGTGCTCAACGTCTTATCGTCTACGACTAGCTCGGTGGCCCTCCCCGCCGGCGCCCAAC

TAAACTCTGTTT--TTATGGCATTCTGAATTATAAACTTAATAAGTTAAAACTTTCAACA

ACGGATCTCTTGGTTCTGGCATCGATGAAGAACGCAGCGAAATGCGATAAGTAATGTGAA

TTGCAGAATTCAGTGAATCATCGAATCTTTGAACGCACATTGCGCCCATTAGCATTCTAG

TGGGCATGCCTGTTCGAGCGTCATTTCACCACTTAAGCC-CTGTTGCTTAGCGTTGGGAG

CCTACGGCACGCCCGTAGCTCCCTAAAGTGATTGGCGGAGTTGGTTCTCACTCTAGGCGT

AGTAAAT-CTATCTCGCCTCTGTAGTGGTTCCGGCCCCTAAA------------------

--------------------------------------------

>Muscodor_indica_NFCCI_3235

------------------------------------------------------------

-----------CCTATAGGGGATACCACATAGTGGTTACCCTGTAGTCCCACGTCCTAGA

TCCGGCTCAACGTCTTATCGTCTACGACTAGCTCGGTGGCCCTCCCCGCCGGCGGCCAAC

TAAACTCTGTTT--TTATGGCATTCTGAATTATAAACTTAATAAGTTAAAACTTTCAACA

ACGGATCTCTTGGTTCTGGCATCGATGAAGAACGCAGCGAAATGCGATAAGTAATGTGAA

TTGCAGAATTCAGTGAATCATCGAATCTTTGAACGCACATTGCGCCCATTAGCATTCTAG

TGGGCATGCCTGTTCGAGCGTCATTTCACCACTTAAGCC-CTGTTGCTTAGCGTTGGGAG

CCTACGGCACGCCCGTAGCTCCCTAAAAGGATTGGCGGAGTTGGTTCTCACTCTAGGCGT

AGTAAAT-CTATCTCGCCTCTGTAGTGGTC------------------------------

--------------------------------------------

>Muscodor_suthepensis_JCM_18232

--ATTACAGAGTTTCTAAACTCCCAACCCTATGTGAACTTACC-TTTGTTGCTTCGGCGG

CGGAGGCT-ACCCTATAGGGGATACCACCTAGTGGTTACCCTGTAGTCCCGGGTGCTGAT

TCTTCCTCAACGTCTTATCGTCAAAGGTCAGTTTGGTGGCCCTCCCCGCCGGCGGCCAAC

TAAACTCTGTTT--TTATGGCATTCTGAATTATAAACTTAATAAGTTAAAACTTTCAACA

ACGGATCTCTTGGTTCTGGCATCGATGAAGAACGCAGCGAAATGCGATAAGTAATGTGAA

TTGCAGAATTCAGTGAATCATCGAATCTTTGAACGCACATTGCGCCCATTAGCATTCTAG

TGGGCATGCCTGTTCGAGCGTCATTTCACCACTTAAGCC-CTGTTGCTTAGCGTTGGGAG

CCTACGGCACGCCCGTAGCTCCCTAAAGTGATTGGCGGAGTTGGTTCTCACTCTAGGCGT

AGTAAAT-CTATCTCGCCTCTGTAGTGGTTCCGGCCCCTGCCGTAAAACCCCCT--ATAT

CAAAGGTTGACCTCG-----------------------------

>Muscodor_darjeelingensis_NFCCI_3095

----------------------------------------------------------TC

GGGAGGCT-ACCCTATAGGGGATACCACATAGTGGTTACCCTGTAGTCCCAGGTGCTAGA

TCGTGCTCAACGTCTTATCGTCTACGACTAGCTCGGTGGCCCTCCCCGCCGGCGGCCAAC

TAAACTCTGTTT--TTATGGCATTCTGAATTATAAACTTAATAAGTTAAAACTTTCAACA

ACGGATCTCTTGGTTCTGGCATCGANGAAGAACGCAGCGAAACGCGATAAGTAACGTGAA

TTGCCGAATTCAGTGAATCCTCGAATCTTTGAACGCCCATTGCGCCCGTTAGCCTTNTAG

TGGGCATGCATGTTCGAGCGTCATTTCACCACTTAAGCC-CTGTTGCTTAGCGTTGGGAG

CCTACGGCACGCCCGTAGCTCCCTAAAGTGATTGGCGGAGTTGGTTCTCACTCTAGGCGT

AGTAAAT-CTATCTCGCCTCTGTAGTGGTTCCGGCCCCTAA-------------------

--------------------------------------------

>Muscodor_strobelii_NFCCI_2907

----------------------------------------------------------TC

GGGAGGCT-ACCCCATAGGGGATACCACATAGTGGTTACCCGGTAGTCCCAGGTGCTAGA

TCGTGCTCAACGTCTTATGGTCTACGGGCAGCTCGGNGGCCCTCCCCGCCGGCGGCCAAC

TAAACTCNGTTT--TTATGGCATTNTGAATCATAAACTTAATAAGTTAAAACTTTCAACA

ACGGATCTNTTGGTTCTGGCATCGATGAAGAACGCAGCGAAATGCGATAAGTAACGTGAA

TTGCAGAATTCAGTGAATCATCGAATCTTTGAACGCACATTGCGCCCATTAGCATTCTAG

TGGGCATGCCTGTTCGAGCGTCATTTCACCACTTAAGCC-CTGTTCCTNAGCGTTGGGAG

CCTACGGCACGCCCGTAGCTCCTTAAAGTGATG-GCGGAGTTGTTCTCACNNTTAGGCCG

GAGTAAA-TCATCTNGCCTCTCTAATGGTTCCGGCCCCCGA-------------------

--------------------------------------------

>Muscodor_yunnanensis_CGMCC_3.18908

-CATTACAGAGTTTCTAAACTCCCAACCCTATGTGGACTTACTT-TTGTTGCTTCGGCGG

CGGAGGTCA--ACTACGGGGGACACCACGCAGTGGCTACCCTGTAGTCCCAGGTGCTGAT

TCCTGCACAGCGTTTTCTCGCACAGGGTCAGCTCGGTGGCCCTCCCCGCCGGCGGCCAAC

TAAACTCTGTCT-TTTTTGGCATTCTGAATCATAAACTTAAT-AGTTAAAACTTTCAACA

ACGGATCTCTTGGTTCTGGCATCGATGAAGAACGCAGCGAAATGCGATAAGTAATGTGAA

TTGCAGAATTCAGTGAATCATCGAATCTTTGAACGCACATTGCGCCCATTAGCATTCTAG

TGGGCATGCCTGTTCGAGCGTCATTTCACCACTTAAGCC-ATGTTGCTTAGCGTTGGGAG

CCTACGGCGCGCCCGTAGCCCCCTAAAGTGATTGGCGGAGTCGGTTCTCACTCTAGACGT

AGTAAAT-CA-TCTCGCCTCTGTAGTGGTCCCGGCCCCTGCCGTAAAACCCCC--TATAT

CAAAGGTTGACCTCGGATCAGGTAGGAATACCCGCTAAACTTAA

>Muscodor_fengyangensis_CGMCC_2862

------CAGAGTTTCTAAACTCCCAACCCTTTGTGAACCTACC-ATCGTTGCTTCGGCGG

CGGAGGGCTACGCTGCAAGCGCTACCCTGTAG---TTACCCTGTAGTCCCAGGG------

-----------------------------AGCTGGACTGCCCTCCCCGCCGGCGGCCAAC

TAAACTCTGTTT-TCTCTGAAACTCTGAATTATAAACTTAATAAGTTAAAACTTTCAACA

ACGGATCTCTTGGTTCTGGCATCGATGAAGAACGCAGCGAAATGCGATAAGTAATGTGAA

TTGCAGAATTCAGTGAATCATCGAATCTTTGAACGCACATTGCGCCCATTAGCATTCTAG

TGGGCATGCCTGTTCGAGCGTCATTTCACCACTTAAGCC-CTGTTGCTTAGCGTTGGGGG

CCTACGGCACGCCTGTAGCCCTTTAAAGTGATTGGCGGAGTTAGTTCTATCTCTAAGCGT

AGTAATT-TCTTCTCGCTTCTGCAGTAGTGCTGGCCCCCGCCGTAAAACCCTCC--TATA

CAATGG--------------------------------------

>Camillea_obularia_ATCC_28093

TCATTAGCGAGTTTTACAAACTCCAAACCCCTGTGAACATACCTATTGTTGCCTCGGCAG

GTCGTGGTCTCTCTACAGTAGTTACCCTGTAGTGCTTACCCCGCAGC-------------

---------------------------------ACCCAACAAGACCTGTCAGAGGACCTT

TAAACTCTATTTTATAACGTATCTCTGAATAACTATACAAATAAGTTAAAACTTTCAACA

ACGGATCTCTTGGTTCTGGCATCGATGAAGAACGCAGCGAAATGCGATAAGTAATGTGAA

TTGCAGAATTCAGTGAATCATCGAATCTTTGAACGCACATTGCGCCTAATAGTATTCTGT

TAGGCATGCCTGTTCGAGCGTCATTTCAACCCCCAAGCCCTATTTGCTTGACGTTGGGAG

TTTACGG---AAACGTAATTCCTCAAATATAGTGGCGGAGCTAGGTCGTGCTCTAAGCGT

AGTAACTTAATTCTCGCTTCTGTAGCCGGCTAAGGTCCTGCCGTAAAACCCCTAATTTTT

CTCTGGTTGACCTCGGATCAGGTAGGAATACCCGCTGAACTTAA

>Obolarina_dryophila_MUCL_49882

------GCGAGTTCTACAAACTCCAAACCCATGTGAACATACCTACTGTTGCCTCGGCAG

GTCGTGACCGCCCCCCGGCAGCTACCCTGCAGCGCCTACCCCGCAGC-------------

---------------------------------ACGCTG-AAGGCCTGCCGAAGGTCCCC

TAAACTCTGTTTTACACCGTATCTCTGAGTTATTATAAAAATAAGTTAAAACTTTCAACA

ACGGATCTCTTGGTTCTGGCATCGATGAAGAACGCAGCGAAATGCGATAAGTAATGTGAA

TTGCAGAATTCAGTGAATCATCGAATCTTTGAACGCACATTGCGCCTAATAGTATTCTGT

TAGGCATGCCTGTTCGAGCGTCATTTCAACCCCCAAGCCTTATTTGCTTGACGTTGGGAG

TTTACGG---AGACGTAATTCCTCAAATATAGTGGCGGAGCTAGGTCGTGCTCTGAGCGT

AGTAGCTAAAACCTCGCTTCTGTAGCCGGCCTGGGTCCTGCCGTAAAACCCCCTATTTTC

TTATGGTTGACCTCGGATCAGGTAGGAATACCCGCTGAACTTAA

>Biscogniauxia_marginata_MFLUCC_12_0740

TCATTAGCGAGTTTGGAAAACTCCAAACCCCTGTGAATATACCTATTGTTGCCTCGGCAG

GCTGTG---GTCTGGTAGCTCCTACCCTGTAGCTGCTACCCCGCAGC-------------

---------------------------------ACGTTGTAAGGCCTGCCGGAGGACCTC

TAAACTCTGAATATTACTGTATGTCTGAGTATTTGAGAAATTGAGTTAAAACTTTCAACA

ACGGATCTCTTGGTTCTGGCATCGATGAAGAACGCAGCGAAATGCGATAAGTAATGTGAA

TTGCAGAATTCAGTGAATCATCGAATCTTTGAACGCACATTGCGCCCTGCAGTACTCTGC

TGGGCATGCCTGTTCGAGCGTCATTTCGACCATCAAGCCCTGTATGCTTGACGTTGGGAA

TTTACAG---TGCTGTAATTCCTTAAATCCAGTGGCGGAGCCGGGTCATGCTCTAGGCGT

AGTAATTATATCCTCGCCTCTGTAGCTGTCCCTTATCCTGCCGTAAAGCCCACTAATTAT

AACTGGTTGACCTCN-----------------------------

>Graphostroma_platystoma_CBS_270.87

TCATTAGCGAGTT-AACAACTCCAAAACCCATGTGAACATACCTATCGTTGCCTCGGCAG

GCTGCGGTTACCCTGTAGGAGCTACCCTGTAG-AGCTACCTCGTAAGCT-----------

---------------------------------GCTGCCTAAAGCCTGCCGGCGGACCCC

TAAACTCTGAAT-TTACTGTATCTCTGAGTTAAAACAAAAATAAGTTAAAACTTTCAACA

ACGGATCTCTTGGTTCTGGCATCGATGAAGAACGCAGCGAAATGCGATAAGTAATGTGAA

TTGCAGAATTCAGTGAATCATCGAATCTTTGAACGCACATTGCGCCTAATAGTATTCTGT

TAGGCATGCCTGTTCGAGCGTCATTTCGACCATTAAGCCCTGTTTGCTTAGCGTTGGGAA

CTTACGCC--TGCCGTAATTCCTTAAATTCAGTGGCGGAGCTAGGTCATGCTCTAAGCGT

AGTAATTATTTCCTCGCTTCTGTAGCTGGCCTATATCCTGN-------------------

--------------------------------------------

>Astrocystis_concavispora_MFLUCC_14.0174

TCATTAACGAGTTTCCCAACTCCCAAACCC-TGTGAACATACCTATCGTTGATCCGTACG

AT-GC-CTCGC----TGA----CGC-----------------------------------

---------------------------------GGGGCGCGCGGCCTGCCGGCGGCCCAA

CCAACCATGTTT-ACTTTTGAGCTCTGA--ACACAACTAAACAAGTTAAAACTTTCAACA

ACGGATCTCTTGGTTCTGGCATCTATGAAGAACGCAGCGAAATGCGATAAGTAATGTGAA

TTGCAGAATTCAGTGAATCATCGAATCTTTGAACGCACATTGCGCCCATTAGTATTCTAG

TGGGCATGCCTGTTCGAGCGTCATTTCAACCCTCAAGCCCCTGTCGCTTGGCGTTGGGAG

ACTACAG---CGACGTATCTCCCCAAATACAGTGGCGGGGTCGGTTCGCACCCTAGGCGT

AGTAGCATCTATCTCGCCTGCGGGCCGGGCCGGTCCCCTGCCGTGAAACCCCCC-ATTTC

TAAAGGTTGACCTCGGATCAGGTAGGAAAACCCCTTGAACTTAA

>Rhopalostroma_angolense_CBS_126414

-------------------ACTCCCACCCTTTGCGAACCTACC-ACTGTTGCCTCGGCGG

GACGC----GC----GAG----AAC-----------------------------------

---------------------------------GCGCT--CAGGCCCGCCGGCGGACTGC

TATATTCTGTCAGGTAACGTAACTCTGAATCTTCAAACAAATAAGTTAAAACTTTCAACA

ACGGATCTCTTGGTTCTGGCATCGATGAAGAACGCAGCGAAATGCGATAAGTAATGTGAA

TTGCAGAATTCAGTGAATCATCGAATCTTTGAACGCACATTGCGCCCGTTAGTATTCTAG

CGGGCATGCCTGTTCGAGCGTCATTACGACCCTTAAGCC-CTGTAGCTTAGCGTTGGGAA

CCTAGGTTTCGTGCCCAGCTCCCTAAAGGTAGTGGCGGAGTCGGGTCCTGCTCTGAGCGT

AGTAGTATTCTTCTCGCTTCGGTAGTAGCCCGGCGGCCTGCCGTAAAACAACCC------

---------------------------------------CCTAN

>Annulohypoxylon_truncatum_CBS_140778

-------------------------ACCCTTTGTGAACCTACC-GTCGTTTCCTCGGCGC

ACTGCGACCGCCCCGCAGTACTTACCCTGTAC-ATCTACCCTGCAG--------------

---------------------------------CAGCT--AAGGCGCAGCGGCGCACCGC

CAAACTCT-TTTACCACT--ACGTCTGAACACTATGCAAAATAGTTTAAAACTTTCAACA

ACGGATCTCTTGGTTCTGGCATCGATGAAGAACGCAGCGAAATGCGATAAGTAATGTGAA

TTGCAGAATTCAGTGAATCATCGAATCTTTGAACGCACATTGCGCCCATTAGTATTCTAG

TGGGCATGCCTATTCGAGCGTCATTTCGACCCTTAAGCC-CTGTTGCTTAGCGTTGGGAG

TCTGCGGC--GGCCGCAGTTCCTTAAAGTCAGTGGCGGAGCTGTGGCACACTCTAGGCGT

AGTAGTTTCCGCCTCGCCTCCAGAGTGGCCCGGCTGCCTGCCGTAAAACCCCTAATTTTC

TAGTGGTTGACCTCGGATTAGGTAGGAATACCCGCTGAACTTAA

>Hypoxylon_fragiforme_MUCL_51264

TCATTAGAGGAATCCAAAACTCCCAACCCCTAGTGAACTTACC-ACTGTTTCCTCGGCGT

GCCGTGCCTACCCTGTAG----TACCCTGTAG---CTACCCTGTAGACCCGGGTCC----

---------------------------------CCGCTCAAGGTCCCGCCGAAGTACCC-

TGAACTCTGTTTAA--GTGGAATTCTGAATCTTCAACTAAATAAGTTAAAACTTTCAACA

ACGGATCTCTTGGTTCTGGCATCGATGAAGAACGCAGCGAAATGCGATAAGTAATGTGAA

TTGCAGAATTCAGTGAATCATCGAATCTTTGAACGCACATTGCGCCCATTAGTATTCTAG

TGGGCATGCCTATTCGAGCGTCATTTCAACCCTTAAGCCTCTGTTGCTTAGCGTTGGGAG

TCTACGG---CCCTGTAGTTCCTGAAAACCATTGGCGGAGTCAGGGAGCACTCTAAGCGT

ATTACACATTGTCTCGCTTTGGATATTCCCCGCCTCCACGCCGTAAAACCCCCC--ATAT

CAAATGTTGACCTCG----------------------------N

>Hypocreodendron_sanguineum_J.D.R._169

TCATTACAGAGTTAAACAAACTCACACCCATTGTGAACCTACCTACCGTTGCCTCGGCGG

CCTCTGCCAAACCCGGCACTCCTGCTCTGTGGTCCGTGTCTGGTTGG-------------

-------------------------------------------GGCCGCCGGCGCACCAC

AAAATTCTGTTTTCTTTTGCAACTCTGAACAGACAAAAAAATTAGTCAAAACTTTCAACA

ACGGATCTCTTGGTTCTGGCATCGATGAAGAACGCAGCGAAATGCGATAAGTAATGTGAA

TTGCAGAATTTAGTGAATCATCGAATCTTTGAACGCACATTGCGCCCACTAGTATTCTGG

TGGGCATGCCTGTTCGAGCGTCATTTCAACCCTTAAGCCTTTGCTGCTTAGTGTTGGGAG

CCGGGCGGCCTGCGGCCGCTCCTCAAAGTCAGTGGCGGAGTCGGTACACGCTCTAGACGC

AGTAACTTTCATCTCGTCTAC-GGTTGTGCCGGCCCCCCGCCGTAAAACACCCCCAATAA

CTAGAGTTGACCTCGGATCAGGTAGGGATACCCGCTGAACTT--

>Thamnomyces_dendroidea_CBS_123578

TCATTAGCGAGTGTAATAACTCGTAGCCTCGTGCGAACCTACC--GCGTAGCCTCGGCGG

GTCGTG---GCCCGG-----------------TGTTTAGCCGGGTG--------------

---------------------------------GCGCGCTATGGCCCGTCGGTGGACGTT

TTAACCTTGCCCA-CG----TATTCTGAATGGACTAGTAAACTA-TTACAACTTTCAACG

ACGGATCTCTTGGTTCTGGCATCGATGAAGAACGCAGCGAAATGCGATACGTAATGCGAA

TTGCAGAATTCAGTGAGTCATCGAATCTTTGAACGCACATTGCGCCCGCTAGCATTCTAG

CGGGCATGCCTGCTCGAGCGTCATTACA-CCCCTAAGCC-----TAGCTTGCGTTGGGAA

TCTAGTCAGCCCGGCTAGTTCCTCAAAATCAGTGGCGGAGTCAGGATAGACCGTACGCGT

AGTAA---TCATCTCGCCTGCGTAG---CCTGGCGGCTTGCCGTAAAACATCCT------

CCACAGTN------------------------------------

>Pyrenopolyporus_hunteri_MUCL_52673

------------------------AACCCTTTGTGAACCTACC-GTCGTTGCCTCGGCGT

GACGCACCTACCCCGCAAATCCTGCCCTGTCGATTCTGCTCCAAAGCTTG----------

---------------------------------ATGCTCCAAAGCTCGAAATGAAGCTCC

AAAACTT-ATTTT---TT--TTTTCTGAATTTCAATTAAAATCAGTTAAAACTTTCAACA

ACGGATCTCTTGGTTCTGGCATCGATGAAGAACGCAGCGAAATGCGATAAGTAATGTGAA

TTGCAGAATTCAGTGAATCATCGAATCTTTGAACGCACATTGCGCCCATTAGTATTCTAG

TGGGCATGCCTATTCGAGCGTCATTTCGACCCTTAAGCCCTCGTTGCTTAGCGTTGGGAG

CCTGCGTCCCGGGCGCAGTTCCTCAAAGTTAGTGGCGGAGCTAGGGCACACTCTAAGCGT

AGTAAGC-TATTCTCGCTTCTGTGGTGTCCTGGCTTCCTGCCGTAAAACCCCN-------

--------------------------------------------

>Rostrohypoxylon_terebratum_CBS_119137

TCATTACTGAGTTTAAAAACTCCCAACCCACTGTGAACCTACCTCTGTTTCCTCCGGCGC

TCCGCG---ACCCTTTAG----CAC----TAGCGTCCACCCCGCGTCCTA----------

---------------------------------GCGGGGTATGATTTCACAATGGCCCCT

GAAACTTCGTTTTGTGATGGAACCGCAGGTTAAAACCAAAATCAGTTAAAACTTTCAACA

ACGGATCTCTTGGTTCTGGCATCGATGAAGAACGCAGCGAAATGCGATAAGTAATGTGAA

TTGCAGAATTCAGTGAATCATCGAATCTTTGAACGCACATTGCGCCCGTTAGCATTCTAG

CGGGCATGCCTATTCGAGCGTCATTACAACCCTTAAGCC-CTGTTGCTTAGCGTTGGGAG

TCTGCGC---GGGCGCAGTTCCTTAAATGTAGTGGCGGAGTTACAGCACACCCTGAGCGT

AGTAGTGTTCAGCTCGCTCCCGGGGAATTGTGGCTGCTTGCCGTAAAA------------

# LSU

>Lopadostoma_turgidum_CBS_133207

AGAAACCAACAGGGATTGCCCTAGTAACGGCGAGTGAAGCGGCAACAGCTCAAATTTGAA

ATCTGGCCCTTGGGTCCGAGTTGTAATTTGTAGAGGATGCTTTTGGTACGGCGCCTTCCG

AGTTCCCTGGAACGGGACGCCATAGAGGGTGAGAGCCCCGTACGGTTGGATGCCTAGCCT

CTGTAAAGCTCTTTCGACGAGTCGAGTAGTTTGGGAATGCTGCTCTAAATGGGAGGTAAA

TTTCTTCTAAAGCTAAATACCGGCCAGAGACCGATAGCGCACAAGTAGAGTGATCGAAAG

ATGAAAAGCACTTTGAAAAGAGGGTTAAATAGCACGTGAAATTGTTGAAAGGGAAGCGCT

TATGACCAGACTTTTACCTGGCGGATCATCCGGCGTTCTCGCCGGTGCACTTCGCTAGGT

TGAGGCCAGCATCGATTTTTGTAGGGGGATAAAGACTTCGGGAAAGTAGCTCTTCGGGGA

GTGTTATAGCCCGCTGTGTAATACCCTTACGGGGATCGAGGTTCGCGCTCTGCAAGGATG

CTGGCATAATGGTCATCAGTGACCCGTCTTGAAACACGGACCAAGGAGTCGAACATTTAT

GCGAGTGTTTGGGTGTTAAACCCTCACGCGTAATGAAAGTGAACGGAGGTGAGAGCCCTT

AAGGGTGCATCATCGACCGATCCTGAAGTCTTCGGATGGATTTGAGTAAGAGCATAACTG

TTCGGACCCGAAAGATGGTGAACTATGCGTGGATAGGGTGAAGCCAGAGGAAACTCTGGT

GGAGGCTCGCAGCGGTTCTGACGTGCAAATCGATCGTCAAATCTGCGCATGGGGGCGAAA

GACTTATCGAACCAT---------------------------------------------

------------------------------------------------------------

------------------------------------------------------------

------------------------------------------------------------

------------------------------------------------------------

------------------------------------------------------------

------------------------------------------------------------

------------------------------------------------------------

---------

>Creosphaeria_sassafras_ST.MA._14087

NNNNNNNNNNNNNNNNTGCCCTAGTAACGGCGAGTGAAGCGGCAACAGCTCAAATTTGAA

ATCTGGCCTTCGGGTCCGAGTTGTAATTTGTAGAGGATGCTTTTGGTGCGGTACCTTCCG

AGTTCCCTGGAACGGGACGCCATAGAGGGTGAGAGCCCCGTACGGTTGGATACCTAGCCT

CTGTAAAGCTCCTTCGACGAGTCGAGTAGTTTGGGAATGCTGCTCTAAATGGGAGGTAAA

TTTCTTCTAAAGCTAAATACCGGCCAGAGACCGATAGCGCACAAGTAGAGTGATCGAAAG

ATGAAAAGCACTTTGAAAAGAGGGTTAAATAGCACGTGAAATTGTTGAAAGGGAAGCGCT

TATGACCAGACTTTTGTCCGGCGGATCATCCGGTGTTCTCACCGGTGCACTTCGCCGGGC

TGAGGCCAGCATCGATTTCTGTAGGGGGATAAAGACTTCAGGAAAGTAGCTCCTCGGGGA

GTGTTATAGCCAGTTGTGTAATACCCCTATAGGGATCGAGGTTCGCGCATCGCAAGGATG

CTGGCATAATGGTCATCAGCGACCCGTCTTGAAACACGGACCAAGGAGTCGAACATTTAT

GCGAGTGTTTGGGTGTTAAACCCTCACGCGTAATGAAAGTGAACGGAGGTGAGAGCCCTT

ACGGGTGCATCATCGACCGATCCTGATGTCTTCGGATGGATTTGAGTAAGAGCATAACTG

TTCGGACCCGAAAGATGGTGAACTATGCGTGGATAGGGTGAAGCCAGAGGAAACTCTGGT

GGAGGCTCGCAGCGGTTCTGACGTGCAAATCGATCGTCAAATCTGCGCATGGGGGCGAAA

GACTAATCGAACCATCTAGTAGCTGGTTACCGCCGAAGTTTCCCTCAGGATAGCAGTGTT

G-TTTTCAGTTTTATGAGGTAAAGCGAATGATTAGGGACTCGGGGGCGCCATATAGCCTT

CATCCATTCTCAAACTTTAAATATGTAAGAAGCCCTTGTTACTTAATTGAACGTGGGCAT

TCGAATGTATCAACACTAGTGGGCCATTTTTGGTAAGCAGAACTGGCGATGCGGGATGAA

CCGAACGCGAGGTTAAGGTGCCAGAGTAGACGCTCATCAGACACCACAAAAGGTGTTAGT

ACATCTTGACAGCAGGACGGTGGCCATGGAAGTCGGAATCCGCTAAGGACTGTGTAACAA

CTCACCTGCCGAATGTACTAGCCCTGAAAATGGATGGCGCTCAAGCGTCTCACCCATACC

TCGCCCTTAGGGTAGAAACGATGCCCTAAGGAGTAGGCGGCCGTGGGGGTCAGNNNNNNN

NNNNNNNNN

>Diatrype_disciformis_CBS_197.49

AGAAACCAACAGGGATTGCCCTAGTAACGGCGAGTGAAGCGGCAACAGCTCAAATTTGAA

ATCTGGCCTTCGGGTCCGAGTTGTAATTTGTAGAGGATGCTTTTGGTGAGGTGCCTTCCG

AGTTCCTTGGAACAGGACGCCTTAGAGGGTGAGAGCCCCGTACGGTTGGACACCAAGCCT

TTGTAAAGCTCCTTCGACGAGTCGAGTAGTTTGGGAATGCTGCTCTAAATGGGAGGTAAA

TTTCTTCTAAAGCTAAATACCGGCCAGAGACCGATAGCGCACAAGTAGAGTGATCGAAAG

ATGAAAAGTACTTTGAAAAGAGGGTTAAATAGCACGTGAAATTGTTGAAAGGGAAGCGTT

TATGACCAGACCTTTGTCAGGCGGATCATCCGGTGTTCTCACCGGTGCACTTCGCCTGGC

TCAGGCCAGCATCGATTTCTGTAGAGGGATAAAGACCATGGGAACGTAGCTCTTCGGGGA

GTGTTATAGCCCTAGGTGTAATACCTTTACGGGGATCGAGGTTCGCGCTTCGCAAGGATG

CTGGCGTAATGGTCATCAATGACCCGTCTTGAAACACGGACCAAGGAGTCGAACATTTAT

GCAAGTGTTTGGGTGTTAAACCCTCACGCGTAATGAAAGTGAACGGAGGTGAGAGCCTTG

TTAGGTGCATCATCGACCGATCCTGATGTATTCGGAAGGATTTGAGTAAGAGCATAACTG

TTCGGACCCGAAAGATGGTGAACTATGCGTGGATAGGGTGAAGCCAGAGGAAACTCTGGT

GGAGGCTCGCAGCGGTTCTGACGTGCAAATCGATCGTCAAATCTGCGCATGGGGGCGAAA

GACTTATCGAACCATCTAGTAGCTGGTTACCGCCGAAGTTTCCCTCAGGATAGCAGTGTT

G-TATTCAGTTTTATGAGGTAAAGCGAATGATTAGGGACTCGGGGGCTATATATTGCCTT

CATCCATTCTCAAACTTTAAATATGTAAGAAGCCCTTGTTACTTAATTGAACGTGGGCAT

TCGAATGTATCAACACTAGTGGGCCATTTTTGGTAAGCAGAACTGGCGATGCGGGATGAA

CCGAACGCGAGGTTAAGGTGCCAGAGTAGACGCTCATCAGACACCACAAAAGGTGTTAGT

ACATCTTGACAGCAGGACGGTGGCCATGGAAGTCGGAATCCGCTAAGGACTGTGTAACAA

CTCACCTGCCGAATGTACTAGCCCTGAAAATGGATGGCGCTCAAGCGTCTCACCCATACC

TCGCCCTTAGGGTAGAAACGATGCCCTAAGGAGTAGGCGGCCGTGGAGGTTAGTGACGAA

GCNNNNNNN

>Eutypa_lata_UCR_EL1

AGAAACCAACAGGGATTGCCCTAGTAACGGCGAGTGAAGCGGCAACAGCTCAAATTTGAA

ATCTGGCCTTCGGGTCCGAGTTGTAATTTGTAGAGGATGCTTTTGGTGCAGTGCCTTCCG

AGTTCCTTGGAACAGGACGCCTTAGAGGGTGAGAGCCCCGTACGGTTGGACACTAAGCCT

CTGTAAAGCTCCTTCGACGAGTCGAGTAGTTTGGGAATGCTGCTCTAAATGGGAGGTAAA

TTTCTTCTAAAGCTAAATACCGGCCAGAGACCGATAGCGCACAAGTAGAGTGATCGAAAG

ATGAAAAGTACTTTGAAAAGAGGGTTAAATAGCACGTGAAATTGTTGAAAGGGAAGCGTT

TATGACCAGACCTTTGCCGGGCGGATCATCCGGTGTTCTCACCGGTGCACTTCGCTCGGC

TTAGGCCAGCATCGATTTCTGGAGGGGGACAAAGACCATGGGAACGTAGCTCTTCGGGGA

GTGTTATAGCCCTAGGTGTAATACCCTTCCGAGGATCGAGGTTCGCGCTTCGCAAGGATG

CTGGCGTAATGGTCATCAACGACCCGTCTTGAAACACGGACCAAGGAGTCGAACATTTAT

GCGAGTGTTTGGGTGTTAAACCCTCACGCGTAATGAAAGTGAACGGAGGTGAGAGCCCT-

TTGGGTGCATCATCGACCGATCCTGATGTATTCGGAAGGATTTGAGTAAGAGCATAACTG

TTCGGACCCGAAAGATGGTGAACTATGCGTGGATAGGGTGAAGCCAGAGGAAACTCTGGT

GGAGGCTCGCAGCGGTTCTGACGTGCAAATCGATCGTCAAATCTGCGCATGGGGGCGAAA

GACTTATCGAACCATCTAGTAGCTGGTTACCGCCGAAGTTTCCCTCAGGATAGCAGTGTT

G-TATTCAGTTTTATGAGGTAAAGCGAATGATTAGGGACTCGGGGGCGCTATATTGCCTT

CATCCATTCTCAAACTTTAAATATGTAAGAAGCCCTTGTTACTTAATTGAACGTGGGCAT

TCGAATGTATCAACACTAGTGGGCCATTTTTGGTAAGCAGAACTGGCGATGCGGGATGAA

CCGAACGCGAGGTTAAGGTGCCAGAGTAGACGCTCATCAGACACCACAAAAGGTGTTAGT

ACATCTTGACAGCAGGACGGTGGCCATGGAAGTCGGAATCCGCTAAGGACTGTGTAACAA

CTCACCTGCCGAATGTACTAGCCCTGAAAATGGATGGCGCTCAAGCGTCTCACCCATACC

TCGCCCTTAGGGTAGAAACGATGCCCTAAGGAGTAGGCGGCCGTGGAGGTTAGTGACGAA

GCCTAGGGC

>Entoleuca_mammata_J.D.R._100

------------------------------------------------------------

------------------------------------------------------------

------------------------------------------------------------

------------------------------------------------------------

------------------------------------------------------------

------------------------------------------------------------

------------------------------------------------------------

------------------------------------------------------------

------------------------------------------------------------

------------------------------------------------------------

------------------------------------------------------------

------------------------------------------------------------

------------------------------------------------------------

------------------------------------------------------------

------------------------------------------------------------

------------------------------------------------------------

------------------------------------------------------------

------------------------------------------------------------

------------------------------------------------------------

------------------------------------------------------------

------------------------------------------------------------

------------------------------------------------------------

---------

>Rosellinia_corticium_MUCL_51693

NNNNNNNNNNNNNNATTGCCCCAGTAACGGCGAGTGAAGCGGCAACAGCTCAAATTTGAA

ATCTGGCCCTCGGGTCCGAGTTGTAATTTGTAGAGGATGCTTTTGGCGCGGTGCCTTCCG

AGTTCCCTGGAACGGGACGCCTTAGAGGGTGAGAGCCCCGTACGGTTGGACACCAAGCCT

CTGTAAAGCGCCTTCGACGAGTCGAGTAGTTTGGGAATGCTGCTCTAAATGGGAGGTAAA

TTTCTTCTAAAGCTAAATATTGGCCAGAGACCGATAGCGCACAAGTAGAGTGATCGAAAG

ATGAAAAGCACTTTGAAAAGAGGGTTAAACAGCACGTGAAATTGTTGAAAGGGAAGCGTT

TGCGACCAGACCTCTCCCCGGCGGATCATCCGGCGTTCTCGCCGGTGCACTTCGCCGGGC

TGAGGCCAGCATCGGTTTCCGCGGGGGGACAAAAGCGGGGGGAACGTAGCTCCCTCGGGA

GTGTTATAGCCCCCCGCATAATACCCTCGCGGGGACCGAGGACCGCGCTTCGCAAGGATG

CTGGCGTAATGGTCGTCAACGACCCGTCTTGAAACACGGACCAAGGAGTCGAACATTTGT

GCGAGTGTTTGGGTGTCAAACCCTCACGCGTAATGAAGGTGAACGTAGGTGAGAGCCTTC

ACGGGCGCATCATCGACCGATCCTGATGTCTTCGGATGGATTTGAGTAAGAGCATAACTG

TTCGGACCCGAAAGATGGTGAACTATGCGTGGATAGGGTGAAGCCAGAGGAAACTCTGGT

GGAGGCTCGCAGCGGTTCTGACGTGCAAATCGATCGTCAAATCTGCGCATGGGGGCGAAA

GACTTATCGAACCATCTAGTAGCTGGTTACCGCCGAAGTTTCCCTCAGGATAGCAGTGTT

GTTCTTCAGTTTTATGAGGTAAAGCGAATGATTAGGGACTCGGGGGCGCTCTTTAGCCTT

CATCCATTCTCAAACTTTAAATATGTAAGAAGCCCTTGTTGCTTAGTTGAACGTGGGCAT

TCGAATGTACCAACACTAGTGGGCCATTTTTGGTAAGCAGAACTGGCGATGCGGGATGAA

CCGAACGCGGGGTTAAGGTGCCGGAGTGGACGCTCATCAGACACCACAAAAGGTGTTAGC

ACATTTAGACAATAGGACGGTGGCCATGGAAGTCGGAATCCGCTAAGGACTGTGTAACAA

CTCACCTATCGAATGTGCTAGCCCTGAAAATGGATGGCGCTCAAGCGTCCCACCCATACC

TCGCCCTCGGGGTAGGATCGGTGCCCCGAGGAGTAGGCGGCCGTGGGGGTCAGTGACGAA

GCCTAGGNN

>Xylaria_hypoxylon_CBS_122620

NNNNNNNNNNNNNNNNNNNNNNNNNNNNNNNNAGTGAAGCGGCAACAGCTCAAATTTGAA

ATCTGGCTTTCGGGTCCGAGTTGTAATTTGTAGAGGATGCTTTTGGCGCGGTGCCTTCCG

AGTTCCCTGGAACGGGACGCCTTAGAGGGTGAGAGCCCCGTACGGTTGGACACCAAGCCT

CTGTAAAGCTCCTTCGACGAGTCGAGTAGTTTGGGAATGCTGCTCTAAATGGGAGGTAAA

TTTCTTCTAAAGCTAAATATTGGCCAGAGACCGATAGCGCACAAGTAGAGTGATCGAAAG

ATGAAAAGCACTTTGAAAAGAGGGTTAAATAGCACGTGAAATTGTTGAAAGGGAAGCGTT

TGCGACCAGACCTTTTCTTAGCGGATCATCCGGTGTTATCACCGGTGCACTTCGCTAAGT

TTAGGCCAGCATCGGTTTCTGTAGGGGGATAAAAGCCTTGGGAACGTAGCTCCTTCGGGA

GTGTTATAGCCCTTTGCATAATACCCTTCTGGGGACCGAGGACCGCGCTATGCAAGGATG

CTGGCATAATGGTCGTCAACGACCCGTCTTGAAACACGGACCAAGGAGTCGAACATTTAT

GCGAGTGTTTGGGTGTTAAACCCTCACGCGTAATGAAAGTGAACGGAGGTGAGAGCCCTT

ACGGGTGCATCATCGACCGATCCTGATGTCTTCGGATGGATTTGAGTAAGAGCATAACTG

TTCGGACCCGAAAGATGGTGAACTATGCGTGGATAGGGTGAAGCCAGAGGAAACTCTGGT

GGAGGCTCGCAGCGGTTCTGACGTGCAAATCGATCGTCAAATCTGCGCATGGGGGCGAAA

GACTTATCGAACCATCTAGTAGCTGGTTACCGCCGAAGTTTCCCTCAGGATAGCAGTGTT

GTTCTTCAGTTTTATGAGGTAAAGCGAATGATTAGGGACTCGGGGGCGCTTTTTTGCCTT

CATCCATTCTCAAACTTTAAATATGTAAGAAGCCCTTGTTACTTAATTGAACGTGGGCAT

TCGAATGTACCAACACTAGTGGGCCATTTTTGGTAAGCAGAACTGGCGATGCGGGATGAA

CCGAACGCGGGGTTAAGGTGCCGGAGTGGACGCTCATCAGACACCACAAAAGGTGTTAGC

ACATTTAGACAATAGGACGGTGGCCATGGAAGTCGGAATCCGCTAAGGACTGTGTAACAA

CTCACCTATCGAATGTGCTAGCCCTGAAAATGGATGGCGCTCAAGCGTCCCACCCATACC

TCGCCCTCAGGGTAGAAACGATGCCCTGAGGAGTAGGCGGCNNNNNNNNNNNNNNNNNNN

NNNNNNNNN

>Xylaria_arbuscula_CBS_126415

NNNNNNNNNNNNNNNNNNNNNNNNNNNNGGCGAGTGAAGCGGCAACAGCTCAAATTTGAA

ATCTGGCCTTCGGGTCCGAGTTGTAATTTGTAGAGGATGCTTTTGGCGCGGTGCCTTCCG

AGTTCCCTGGAACGGGACGCCTTAGAGGGTGAGAGCCCCGTACGGTTGGACACCAAGCCT

CTGTAAAGCTCCTTCGACGAGTCGAGTAGTTTGGGAATGCTGCTCTAAATGGGAGGTAAA

TTTCTTCTAAAGCTAAATATTGGCCAGAGACCGATAGCGCACAAGTAGAGTGATCGAAAG

ATGAAAAGCACTTTGAAAAGAGGGTTAAATAGCACGTGAAATTGTTGAAAGGGAAGCGTT

TGCGACCAGACTTTTCCCTAGCGGATCATCCGGTGTTCTCACCGGTGCACTTCGCTAGGT

TAAGGCCAGCATCGGTTTCTGTAGGGGGATAAAAGCTTGGGGAATGTAGCTCCCTCGGGA

GTGTTATAGCCTCTTGTATAATACCCTTACGGGGACCGAGGACCGCGCTTTGCAAGGATG

CTGGCGTAATGGTTGTCAACGACCCGTCTTGAAACACGGACCAAGGAGTCGAACATTTGT

GCGAGTGTTTGGGTGTTAAACCCTCACGCGTAATGAAAGTGAACGGAGGTGAGAGCCCTT

ACGGGTGCATCATCGACCGATCCTGATGTCTTCGGATGGATTTGAGTAAGAGCATAACTG

TTCGGACCCGAAAGATGGTGAACTATGCGTGGATAGGGTGAAGCCAGAGGAAACTCTGGT

GGAGGCTCGCAGCGGTTCTGACGTGCAAATCGATCGTCAAATCTGCGCATGGGGGCGAAA

GACTTATCGAACCATCTAGTAGCTGGTTACCGCCGAAGTTTCCCTCAGGATAGCAGTGTT

GTTCTTCAGTTTTATGAGGTAAAGCGAATGATTAGGGACTCGGGGGCGCTTTTTAGCCTT

CATCCATTCTCAAACTTTAAATATGTAAGAAGCCCTTGTTACTTAATTGAACGTGGGCAT

TCGAATGTACCAACACTAGTGGGCCATTTTTGGTAAGCAGAACTGGCGATGCGGGATGAA

CCGAACGCGGGGTTAAGGTGCCGGAGTGGACGCTCATCAGACACCACAAAAGGTGTTAGC

ACATTTAGACAATAGGACGGTGGCCATGGAAGTCGGAATCCGCTAAGGACTGTGTAACAA

CTCACCTATCGAATGTGCTAGCCCTGAAAATGGATGGCGCTCAAGCGTCCCACCCATACC

CCGCCCTCAGGGTAGAAACGATGCCCTGAGGAGTAGGCGGCCGTGGAGGTCAGTGACGAA

GCCTAGGGC

>Oligostoma_insidiosum_CBS_147288

AGAAACCAACAGGGATTGCCCTAGTAACGGCGAGTGAAGCGGCAACAGCTCAAATTTGAA

ATCTGGCCCTCGGGTCCGAGTTGTAATTTGTAGAGGATGCTTTTGGCGCGGTGCCTTCCG

AGTTCCCTGGAACGGGACGCCTTAGAGGGTGAGAGCCCCGTACGGTTGGACACCAAGCCT

CTGTAAAGCTCCTTCGACGAGTCGAGTAGTTTGGGAATGCTGCTCTAAATGGGAGGTAAA

TTTCTTCTAAAGCTAAATATTGGCCAGAGACCGATAGCGCACAAGTAGAGTGATCGAAAG

ATGAAAAGCACTTTGAAAAGAGGGTTAAACAGCACGTGAAATTGTTGAAAGGGAAGCGTT

TGCGACCAGACCTTTTCCTAGCGGATCATCCGGTGTTCTCACCGGTGCACTTCGCTTGGT

TTAGGCCAGCATCGGTTTCTGTAGGGGGACAAAAGCCCTGGGAACGTAGCTCCCTCGGGA

GTGTTATAGCCCTCTGCATAATACCCTTACGGGGACCGAGGACCGCGCTTCGCAAGGATG

CTGGCGTAATGGTCGTCAACGACCCGTCTTGAAACACGGACCAAGGAGTCGAACATTTGT

GCGAGTGTTTGGGTGTTAAACCCTCACGCGCAATGAAGGTGAACGGAGGTGAGAGCCCTC

ACGGGTGCATCATCGACCGATCCTGATGTCTTCGGATGGATTTGAGTAAGAGCATAACTG

TTCGGACCCGAAAGATGGTGAACTATGCGTGGATAGGGTGAAGCCAGAGGAAACTCTGGT

GGAGGCTCGCAGCGGTTCTGACGTGCAAATCGATCGTCAAATCTGCGCATGGGGGCGAAA

GACTTATCGAACCATCTAGTAGCTGGTTACTGCN--------------------------

------------------------------------------------------------

------------------------------------------------------------

------------------------------------------------------------

------------------------------------------------------------

------------------------------------------------------------

------------------------------------------------------------

------------------------------------------------------------

---------

>Xylaria_laevis_HAST_419

------------------------------------------------------------

------------------------------------------------------------

------------------------------------------------------------

------------------------------------------------------------

------------------------------------------------------------

------------------------------------------------------------

------------------------------------------------------------

------------------------------------------------------------

------------------------------------------------------------

------------------------------------------------------------

------------------------------------------------------------

------------------------------------------------------------

------------------------------------------------------------

------------------------------------------------------------

------------------------------------------------------------

------------------------------------------------------------

------------------------------------------------------------

------------------------------------------------------------

------------------------------------------------------------

------------------------------------------------------------

------------------------------------------------------------

------------------------------------------------------------

---------

>Xylaria_longipes_CBS_148.73

AGAAACCAACAGGGATTGCCCTAGTAACGGCGAGTGAAGCGGCAACAGCTCAAATTTGAA

ATCTGGCCCTCGGGTCCGAGTTGTAATTTGTAGAGGATGCTTTTGGCGCGGTGCCTTCCG

AGTTCCCTGGAACGGGACGCCTTAGAGGGTGAGAGCCCCGTACGGTTGGACACCAAGCCT

CTGTAAAGCTCCTTCGACGAGTCGAGTAGTTTGGGAATGCTGCTCTAAATGGGAGGTAAA

TTTCTTCTAAAGCTAAATATTGGCCAGAGACCGATAGCGCACAAGTAGAGTGATCGAAAG

ATGAAAAGCACTTTGAAAAGAGGGTTAAATAGCACGTGAAATTGTTGAAAGGGAAGCGTT

TGCGACCAGACCTTTTCCTAGGGGATCATCCGGTGTTCTCACCGGTGCACTTCCCTAGGT

TCAGGCCAGCATCGGTTTCTGTAGGGGGATAAAAGCTCTGGGAACGTAGCTCCCTCGGGA

GTGTTATAGCCCTCTGCATAATACCCTTACGGGGACCGAGGACCGCGCTTCGCAAGGATG

CTGGCGTAATGGTCGTCAACGACCCGTCTTGAAACACGGACCAAGGAGTCGAACATTTGT

GCGAGTGTTTGGGTGTTAAACCCTCACGCGTAATGAAAGTGAACGGAGGTGAGAGCCCTT

ACGGGTGCATCATCGACCGATCCTGATGTCTTCGGATGGATTTGAGTAAGAGCATAACTG

TTCGGACCCGAAAGATGGTGAACTATGCGTGGATAGGGTGAAGCCAGAGGAAACTCTGGT

GGAGGCTCGCAGCGGTTCTGACGTGCAAATCGATCGTCAAATCTGCGCATGGGGGCGAAA

GACTTATCGAACCATCTAGTAGCTGGTTACCGCCGAAGT---------------------

------------------------------------------------------------

------------------------------------------------------------

------------------------------------------------------------

------------------------------------------------------------

------------------------------------------------------------

------------------------------------------------------------

------------------------------------------------------------

---------

>Xylaria_digitata_HAST_919

------------------------------------------------------------

------------------------------------------------------------

------------------------------------------------------------

------------------------------------------------------------

------------------------------------------------------------

------------------------------------------------------------

------------------------------------------------------------

------------------------------------------------------------

------------------------------------------------------------

------------------------------------------------------------

------------------------------------------------------------

------------------------------------------------------------

------------------------------------------------------------

------------------------------------------------------------

------------------------------------------------------------

------------------------------------------------------------

------------------------------------------------------------

------------------------------------------------------------

------------------------------------------------------------

------------------------------------------------------------

------------------------------------------------------------

------------------------------------------------------------

---------

>Leptomassaria_simplex_CBS_147282

AGAAACCAACAGGGATTGCCCTAGTAACGGCGAGTGAAGCGGCAACAGCTCAAATTTGAA

ATCTGGCCCTCGGGTCCGAGTTGTAATTTGTAGAGGATGCTTTTGGCGCGGTGCCTTCCG

AGTTCCCTGGAACGGGACGCCTTAGAGGGTGAGAGCCCCGTACGGTTGGACACCAAGCCT

CTGTAAAGCTCCTTCGACGAGTCGAGTAGTTTGGGAATGCTGCTCTAAATGGGAGGTAAA

TTTCTTCTAAAGCTAAATATTGGCCAGAGACCGATAGCGCACAAGTAGAGTGATCGAAAG

ATGAAAAGCACTTTGAAAAGAGGGTTAAATAGCACGTGAAATTGTTGAAAGGGAAGCGTT

TGCGACCAGACCTTTTCCTGGCGGATCATCCGGTGTTCTCACCGGTGCACTTCGCCTGGT

TTAGGCCAGCATCGGTTTCTGTAGGGGGATAAAAGCTCTGGGAACGTAGCTCTTTCGGGA

GTGTTATAGCCCTCTGCATAATACCCTTACGGGGACCGAGGACCGCGCTTCGCAAGGATG

CTGGCGTAATGGTCGTCAACGACCCGTCTTGAAACACGGACCAAGGAGTCGAACATTTGT

GCGAGTGTTTGGGTGTTAAACCCTCACGCGTAATGAAAGTGAACGGAGGTGAGAGCCCTT

ACGGGTGCATCATCGACCGATCCTGATGTCTTCGGATGGATTTGAGTAAGAGCATAACTG

TTCGGACCCGAAAGATGGTGAACTATGCGTGGATAGGGTGAAGCCAGAGGAAACTCTGGT

GGAGGCTCGCAGCGGTTCTGACGTGCAAATCGATCGTCAAATCTGCGCATGGGGGCGAAA

GACTTATCGAACCAT---------------------------------------------

------------------------------------------------------------

------------------------------------------------------------

------------------------------------------------------------

------------------------------------------------------------

------------------------------------------------------------

------------------------------------------------------------

------------------------------------------------------------

---------

>Stilbohypoxylon_elaeicola_Y.M.J._173

------------------------------------------------------------

------------------------------------------------------------

------------------------------------------------------------

------------------------------------------------------------

------------------------------------------------------------

------------------------------------------------------------

------------------------------------------------------------

------------------------------------------------------------

------------------------------------------------------------

------------------------------------------------------------

------------------------------------------------------------

------------------------------------------------------------

------------------------------------------------------------

------------------------------------------------------------

------------------------------------------------------------

------------------------------------------------------------

------------------------------------------------------------

------------------------------------------------------------

------------------------------------------------------------

------------------------------------------------------------

------------------------------------------------------------

------------------------------------------------------------

---------

>Xylaria_ianthinovelutina_HAST_553

------------------------------------------------------------

------------------------------------------------------------

------------------------------------------------------------

------------------------------------------------------------

------------------------------------------------------------

------------------------------------------------------------

------------------------------------------------------------

------------------------------------------------------------

------------------------------------------------------------

------------------------------------------------------------

------------------------------------------------------------

------------------------------------------------------------

------------------------------------------------------------

------------------------------------------------------------

------------------------------------------------------------

------------------------------------------------------------

------------------------------------------------------------

------------------------------------------------------------

------------------------------------------------------------

------------------------------------------------------------

------------------------------------------------------------

------------------------------------------------------------

---------

>Xylaria_polymorpha_MUCL_49884

NNNNNNNNNNNNNNATTGCCCTAGTAACGGCGAGTGAAGCGGCAACAGCTCAAATTTGAA

ATCTGGCCCTCGGGTCCGAGTTGTAATTTGTAGAGGATGCTTTTGGCGCGGTGCCTTCCG

AGTTCCCTGGAACGGGACGCCTTAGAGGGTGAGAGCCCCGTATGGTTGGACACCAAGCCT

CTGTATAGCTCCTTCGACGAGTCGAGTAGTTTGGGAATGCTGCTCTAAATGGGAGGTAAA

TTTCTTCTAAAGCTAAATATTGGCCAGAGACCGATAGCGCACAAGTAGAGTGATCGAAAG

ATGAAAAGCACTTTGAAAAGAGGGTTAAATAGCACGTGAAATTGTTGAAAGGGAAGCGTT

TGCGACCAGACCTTTTCCTAGCGGATCAGCCGGTGTTCTCACCGGTGCACTTCGCTAGGT

CTAGGCCAGCATCGGTTTCCGTAGGGGGATAAAAGCCCTGGGAACGTAGCTCCTCCGGGA

GTGTTATAGCCCTCTGCATAATACCCTTACGGGGACCGAGGACCGCGCTTCGCAAGGATG

CTGGCGTAATGGTCGTCAACGACCCGTCTTGAAACACGGACCAAGGAGTCGAACATTTGT

GCGAGTGTTTGGGTGTTAAACCCTCACGCGTAATGAAAGTGAACGGAGGTGAGAGCCCTT

ACGGGTGCATCATCGACCGATCCTGATGTCTTCGGATGGATTTGAGTAAGAGCATAACTG

TTCGGACCCGAAAGATGGTGAACTATGCGTGGATAGGGTGAAGCCAGAGGAAACTCTGGT

GGAGGCTCGCAGCGGTTCTGACGTGCAAATCGATCGTCAAATCTGCGCATGGGGGCGAAA

GACTTATCGAACCATCTAGTAGCTGGTTACCGCCGAAGTTTCCCTCAGGATAGCAGTGTT

GTTCTTCAGTTTTATGAGGTAAAGCGAATGATTAGGGACTCGGGGGCGCTTTTTTGCCTT

CATCCATTCTCAAACTTTAAATATGTAAGAAGCCCTTGTTACTTAATTGAACGTGGGCAT

TCGAATGTACCAACACTAGTGGGCCATTTTTGGTAAGCAGAACTGGCGATGCGGGATGAA

CCGAACGCGGGGTTAAGGTGCCGGAGTGGACGCTCATCAGACACCACAAAAGGTGTTAGC

ACATTTAGACAATAGGACGGTGGCCATGGAAGTCGGAATCCGCTAAGGACTGTGTAACAA

CTCACCTATCGAATGTGCTAGCCCTGAAAATGGATGGCGCTCAAGCGTCCCACCCATACC

TCGCCCTCAGGGTAGAAACGATGCCCTGAGGAGTAGGCGGCCGTGGGGGTTAGTGACGAA

GCCTANNNN

>Xylaria_atrosphaerica_HAST_91111214

------------------------------------------------------------

------------------------------------------------------------

------------------------------------------------------------

------------------------------------------------------------

------------------------------------------------------------

------------------------------------------------------------

------------------------------------------------------------

------------------------------------------------------------

------------------------------------------------------------

------------------------------------------------------------

------------------------------------------------------------

------------------------------------------------------------

------------------------------------------------------------

------------------------------------------------------------

------------------------------------------------------------

------------------------------------------------------------

------------------------------------------------------------

------------------------------------------------------------

------------------------------------------------------------

------------------------------------------------------------

------------------------------------------------------------

------------------------------------------------------------

---------

>Amphirosellinia_nigrospora_HAST_91092308

------------------------------------------------------------

------------------------------------------------------------

------------------------------------------------------------

------------------------------------------------------------

------------------------------------------------------------

------------------------------------------------------------

------------------------------------------------------------

------------------------------------------------------------

------------------------------------------------------------

------------------------------------------------------------

------------------------------------------------------------

------------------------------------------------------------

------------------------------------------------------------

------------------------------------------------------------

------------------------------------------------------------

------------------------------------------------------------

------------------------------------------------------------

------------------------------------------------------------

------------------------------------------------------------

------------------------------------------------------------

------------------------------------------------------------

------------------------------------------------------------

---------

>Dematophora_necatrix_CBS_349.36

AGAAACCAACAGGGATTGCCTCAGTAACGGCGAGTGAAGCGGCAACAGCTCAAATTTGAA

ATCTGGCCCTCGGGTCCGAGTTGTAATTTGTAGAGGATGCTTTTGGCGCGGTGCCTTCCG

AGTTCCCTGGAACGGGACGCCTTAGAGGGTGAGAGCCCCGTACGGTTGGACACCAAGCCT

CTGTAAAGCTCCTTCGACGAGTCGAGTAGTTTGGGAATGCTGCTCTAAATGGGAGGTAAA

TTTCTTCTAAAGCTAAATATTGGCCAGAGACCGATAGCGCACAAGTAGAGTGATCGAAAG

ATGAAAAGCACTTTGAAAAGAGGGTTAAATAGCACGTGAAATTGTTGAAAGGGAAGCGTT

TGCGACCAGACCTTTTCCTGGCGGATCATCCGGTGTTCTCACCGGTGCACTTCGCCAGGT

CGAGGCCAGCATCGGTTTCCGCGGGGGGATAAAAGCTCTGGGAACGTAGCTCCTCCGGGA

GTGTTATAGCCCTCTGCACAATACCCCCGCGGGGACCGAGGACCGCGCTCTGCAAGGATG

CTGGCGTAATGGTTGTCAACGACCCGTCTTGAAACACGGACCAAGGAGTCGAACATTTGT

GCGAGTGTTTGGGTGTCAAACCCTCACGCGTAATGAAGGTGAACGTAGGTGAGAGCCCTT

ACGGGTGCATCATCGACCGATCCTGATGTCTTCGGATGGATTTGAGTAAGAGCATAACTG

TTCGGACCCGAAAGATGGTGAACTATGCGTGGATAGGGTGAAGCCAGAGGAAACTCTGGT

GGAGGCTCGCAGCGGTTCTGACGTGCAAATCGATCGTCAAATCTGCGCATGGGGGCGAAA

GACTTATCGAACCATCTAGTAGCTGGTTACNNNNNNNNNNNNNNNNNNNNNNNNNNNNNN

NNNNNNNNNNNNNNNNNNNNNNNNNNNNNNNNNNNNNNNNNNNNNNNNNNNNNNNNNNNN

NNNNNNNNNNNNNNNNNNNNNNNNNNNNNNNNNNNNNNNNNNNNNNNNNNNNNNNNNNNN

NNNNNNNNNNNNNNNNNNNNNNNNNNNNNNNNNNNNNNNNNNNNNNNNNNNNNNNNNNNN

NNNNNNNNNNNNNNNNNNNNNNNNNNNNNNNNNNNNNNNNNNNNNNNNNNNNNNNNNNNN

NNNNNNNNNNNNNNNNNNNNNNNNNNNNNNNNNNNNNNNNNNNNNNNNNNNNNNNNNNNN

NNNNNNNNNNNNNNNNNNNNNNNNNNNNNNNNNNNNNNNNNNNNNNNNNNNNNNNNNNNN

NNNNNNNNNNNNNNNNNNNNNNNNNNNNNNNNNNNNNNNNNNNNNNNNNNNNNNNNNNNN

NNNNNNNNN

>Xylaria_oxyacanthae_859_JDR

------------------------------------------------------------

------------------------------------------------------------

------------------------------------------------------------

------------------------------------------------------------

------------------------------------------------------------

------------------------------------------------------------

------------------------------------------------------------

------------------------------------------------------------

------------------------------------------------------------

------------------------------------------------------------

------------------------------------------------------------

------------------------------------------------------------

------------------------------------------------------------

------------------------------------------------------------

------------------------------------------------------------

------------------------------------------------------------

------------------------------------------------------------

------------------------------------------------------------

------------------------------------------------------------

------------------------------------------------------------

------------------------------------------------------------

------------------------------------------------------------

---------

>Albicollum_vincensii_CBS_147286

AGAAACCAACAGGGATTGCCCTAGTAACGGCGAGTGAAGCGGCAACAGCTCAAATTTGAA

ATCTGGCCCTCGGGTCCGAGTTGTAATTTGTAGAGGATGTTTTTGGCGCGGTGCCTTCCG

AGTTCCCTGGAACGGGACGCCTTAGAGGGTGAGAGCCCCGTACGGTTGGACACCAAGCCT

CTGTAAAGCACCTTCGACGAGTCGAGTAGTTTGGGAATGCTGCTCTAAATGGGAGGTAAA

TTTCTTCTAAAGCTAAATATAGGCCAGAGACCGATAGCGCACAAGTAGAGTGATCGAAAG

ATGAAAAGCACTTTGAAAAGAGGGTTAAATAGCACGTGAAATTGTTGAAAGGGAAGCGTT

TGCGACCAGACCTTTTCCTAGCGGATCATCCGGTGTTCTCACCGGTGCACTTCGCTAGGT

TTAGGCCAGCATCGGTTTCCGTAGGGGGATAAAAGCTCGGGGAACGTAGCTCCCTCGGGA

GTGTTATAGCCCCTTGTATAATACCCTTACGGTGACCGAGGACCGCGCCTTGCTAGGATG

CTGGCGTAATGGTCGTTAACGACCCGTCTTGAAACACGGACCAAGGAGTCGAACATTTGT

GCGAGTGTTTGGGTGTCAAACCCTCACGCGTAATGAAAGTAAACGGAGGTGAGAGCCCTT

ACGGGTGCATCATCGACCGATCCTGATGTCTTCGGATGGATTTGAGTAGGAGCATAACTG

TTCGGACCCGAAAGATGGTGAACTATGCGTGGATAGGGTGAAGCCAGAGGAAACTCTGGT

GGAGGCTCGCAGCGGTTCTGACGTGCAAATCGATCGTCAAATCTGCGCATGGGGGCGAAA

GACTTATCGAACCAT---------------------------------------------

------------------------------------------------------------

------------------------------------------------------------

------------------------------------------------------------

------------------------------------------------------------

------------------------------------------------------------

------------------------------------------------------------

------------------------------------------------------------

---------

>Albicollum_longisporum_CBS_147283

AGAAACCAACAGGGATTGCCCTAGTAACGGCGAGTGAAGCGGCAACAGCTCAAATTTGAA

ATCTGGCTTTCGGGTCCGAGTTGTAATTTGTAGAGGATGTTTTTGGCGCGGTGCCTTCCG

AGTTCCCTGGAACGGGACGCCGTAGAGGGTGAGAGCCCCGTACGGTTGGATACTAAGCCT

CTGTAAAACTCCTTCGACGAGTCGAGTAGTTTGGGAATGCTGCTCTAAATGGGAGGTAAA

TTTCTTCTAAAGCTAAATATAGGCCAGAGACCGATAGCGCACAAGTAGAGTGATCGAAAG

ATGAAAAGCACTTTGAAAAGAGGGTTAAACAGCACGTGAAATTGTTGAAAGGGAAGCGTT

TGCGACCAGACCTTTCCCTAGCGGATCATCCGGTGTTCTCACCGGTGCACTTCGCTAGGT

TTAGGCCAGCATCGGTTTCCGTAGGGGGATAAAAGCTCGGGGAACGTAGCTCCCTCGGGA

GTGTTATAGCCCCTTGTATAATACCTTTGTGGGGACCGAGGACCGCGCCTTGCAAGGATG

TTGGCGTAATGGTCGTTAACGACCCGTCTTGAAACACGGACCAAGGAGTCGAACATTTGT

GCGAGTATTTGGGTGTCAAACCCTTATGCGTAATGAAAGTAAACGTAGGTGAGAGCTCTT

GCGGGCGCATCATCGACCGATCTTGATGTCTTCGGATGGATTTGAGTAAGAGCATAACTG

TTCGGACCCGAAAGATGGTGAACTATGCGTGGATAGGGTGAAGCCAGAGGAAACTCTGGT

GGAGGCTCGCAGCGGTTCTGACGTGCAAATCGATCGTCAAATCTGCGCATGGGGGCGAAA

GACTTATCGAACCAT---------------------------------------------

------------------------------------------------------------

------------------------------------------------------------

------------------------------------------------------------

------------------------------------------------------------

------------------------------------------------------------

------------------------------------------------------------

------------------------------------------------------------

---------

>Stromatoneurospora_phoenix_BCC_82040

--------------------------ACGGCGAGTGAAGCGGCAATAGCTCAAATTTGAA

ATCTGGCCTTCGGGCCCGAGTTGTAATTTGTAGAGGATGCTTTTGGCGCGGTGCCTTCCG

AGTTCCCTGGAACGGGACGCCTTAGAGGGTGAGAGCCCCGTACGGTTGGACACCAAGCCT

CTGTAAAGCTCCTTCGACGAGTCGAGTAGTTTGGGAATGCTGCTCTAAATGGGAGGTAAA

TTTCTTCTAAAGCTAAATATTGGCCAGAGACCGATAGCGCACAAGTAGAGTGATCGAAAG

ATGAAAAGCACTTTGAAAAGAGGGTTAAATAGCACGTGAAATTGTTGAAAGGGAAGCGTT

TGCGACCAGACCTTTTCCTAGCGGATCATCCGGTGTTCTCACCGGTGCACTTCGCTAGGT

TGAGGCCAGCATCGGTTTCCGTAGGGGGATAAAGGCAGGGGGAATGTAGCTCCTTCGGGA

GTGTTATAGCCCCCTGCGTAATACCCTTACGGGGACCGAGGACCGCGCTTCGCAAGGATG

CTGGCATAATGGTCGTCAACGACCCGTCTTGAAACACGGACCAAGGAGTCGAACATTTGT

GCGAGTGTTTGGGTGTTAAACCCTCACGCGTAATGAAAGTGAACGGAGGTGAGAGCCCTT

ACGGGTGCATCATCGACCGATCCTGATGTCTTCGGATGGATTTGAGTAAGAGCATAACTG

TTCGGACCCGAAAGATGGTGAACTATGCGTGGATAGGGTGAAGCCAGAGGAAACTCTGGT

GGAGGCTCGCAGCGGTTCTGACGTGCAAATCGATCGTCAAATCTGCGCA-----------

------------------------------------------------------------

------------------------------------------------------------

------------------------------------------------------------

------------------------------------------------------------

------------------------------------------------------------

------------------------------------------------------------

------------------------------------------------------------

------------------------------------------------------------

---------

>Sarcoxylon_compunctum_CBS_359.61

NNNNNNNNNNNNNNNNNNNNNNNNNNNNNNNNNNNNNNNNNNNNNNNNNNNNNNNNNNNN

NNNNNNNNNNNNNNNNNNNNNNNNNNNNNNNNNNNNNNNNNNNNNNNNNNNNNNNNNNNN

NNNNNNNNNNNNNNNNNNNNNNNNNNNNNNNNNNNNNNNNNNNNNNNNNNNNNNNNNNNN

NNNNNNNNNNNNNNNNNNNNNNNNNNNNNNNNNNNNNNNNNNNNNNNNNNNNNNNNNNNN

NNNNNNNNNNNNNNNNNNNNNNNNNNNNNNNNNNNNNNNNNNNNNNNNNNNNNNNNNNNN

NNNNNNNNNNNNNNNNNNAGAGGGTTAAATAGCACGTGAAATTGTTGAAAGGGAAGCGTT

TGCGACCAGACTTTTTCCTGGCGGATCATCCGGTGTTCTCACCGGTGCACTTCGCCTGGT

AAAGGCCAGCATCGGTTTCGGTAGGGGGATAAAAGCCTTGGGAACGTAGCTCTTTCGGGA

GTGTTATAGCCCCTGGCATAATACCCTTACGGGGACCGAGGACCGCGCTTCGCAAGGATG

CTGGCATAATGGTCGTCAACGACCCGTCTTGAAACACGGACCAAGGAGTCGAACATTTGT

GCGAGTGTTTGGGTGTTAAACCCTCACGCGTAATGAAAGTGAACGGAGGTGAGAGCCCTT

ACGGGTGCATCATCGACCGATCCTGATGTCTTCGGATGGATTTGAGTAAGAGCATAACTG

TTCGGACCCGAAAGATGGTGAACTATGCGTGGATAGGGTGAAGCCAGAGGAAACTCTGGT

GGAGGCTCGCAGCGGTTCTGACGTGCAAATCGATCGTCAAATCTGCGCATGGGGGCGAAA

GACTTATCGAACCATCTAGTAGCTGGTTACCGCCGAAGTTTCCCTCAGGATAGCAGTGTT

GTTCTTCAGTTTTATGAGGTAAAGCGAATGATTAGGGACTCGGGGGCTATTTTTTGCCTT

CATCCATTCTCAAACTTTAAATATGTAAGAAGCCCTTGTTACTTAATTGAACGTGGGCAT

TCGAATGTACCAACACTAGTGGGCCATTTTTGGTAAGCAGAACTGGCGATGCGGGATGAA

CCGAACGCGGGGTTAAGGTGCCGGAGTGGACGCTCATCAGACACCACAAAAGGTGTTAGC

ACATTTAGACAATAGGACGGTGGCCATGGAAGTCGGAATCCGCTAAGGACTGTGTAACAA

CTCACCTATCGAATGTGCTAGCCCTGAAAATGGATGGCGCTCAAGCGTCCCACCCATACC

TCGCCCTCAGGGTAGAAACGATGCCCTGAGGAGTAGGCGGCCGTGGGGGTCAGTGACGAA

GCCTAGGNN

>Podosordaria_mexicana_WSP176

------------------------------------------------------------

------------------------------------------------------------

------------------------------------------------------------

------------------------------------------------------------

------------------------------------------------------------

------------------------------------------------------------

------------------------------------------------------------

------------------------------------------------------------

------------------------------------------------------------

------------------------------------------------------------

------------------------------------------------------------

------------------------------------------------------------

------------------------------------------------------------

------------------------------------------------------------

------------------------------------------------------------

------------------------------------------------------------

------------------------------------------------------------

------------------------------------------------------------

------------------------------------------------------------

------------------------------------------------------------

------------------------------------------------------------

------------------------------------------------------------

---------

>Poronia_punctata_CBS_656.78

NNNNNNNNNNNNNNNNNNNNNNNNNNNNNNNNNNNNNNNNNNNNNNNNNNNNNNNNNNNN

NNNNNNNCCTCGGGTCCGAGTTGTAATTTGTAGAGGATGCTTTTGGCGCGGTGCCTTCCG

AGTTCCCTGGAACGGGACGCCTTAGAGGGTGAGAGCCCCGTACGGTTGGACACCAAGCCT

CTGTAAAGCTCCTTCGACGAGTCGAGTAGTTTGGGAATGCTGCTCTAAATGGGAGGTAAA

TTTCTTCTAAAGCTAAATATTGGCCAGAGACCGATAGCGCACAAGTAGAGTGATCGAAAG

ATGAAAAGCACTTTGAAAAGAGGGTTAAATAGCACGTGAAATTGTTGAAAGGGAAGCGTT

TGCGACCAGACCTTTTCCTGGCGGATCATCCGGTGTTCTCACCGGTGCACTTCGCCAGGT

TGAGGCCAGCATCGGTTTCTGCAGGGGGATAAAAGCCTTGGGAACGTAGCTCCTTCGGGA

GTGTTATAGCCCCTGGCATAATACCCTTGCGGGGACCGAGGACCGCGCTTCGCAAGGATG

CTGGCATAATGGTCGTCAACGACCCGTCTTGAAACACGGACCAAGGAGTCGAACATTTGT

GCGAGTGTTTGGGTGTTAAACCCTCACGCGTAATGAAAGTGAACGGAGGTGAGAGCCCTT

ACGGGTGCATCATCGACCGATCCTGATGTCTTCGGATGGATTTGAGTAAGAGCATAACTG

TTCGGACCCGAAAGATGGTGAACTATGCGTGGATAGGGTGAAGCCAGAGGAAACTCTGGT

GGAGGCTCGCAGCGGTTCTGACGTGCAAATCGATCGTCAAATCTGCGCATGGGGGCGAAA

GACTTATCGAACCATCTAGTAGCTGGTTACCGCCGAAGTTTCCCTCAGGATAGCAGTGTT

GTTCTTCAGTTTTATGAGGTAAAGCGAATGATTAGGGACTCGGGGGCTATTTTTTGCCTT

CATCCATTCTCAAACTTTAAATATGTAAGAAGCCCTTGTTACTTAATTGAACGTGGGCAT

TCGAATGTACCAACACTAGTGGGCCATTTTTGGTAAGCAGAACTGGCGATGCGGGATGAA

CCGAACGCGGGGTTAAGGTGCCGGAGTGGACGCTCATCAGACACCACAAAAGGTGTTAGC

ACATTTAGACAATAGGACGGTGGCCATGGAAGTCGGAATCCGCTAAGGACTGTGTAACAA

CTCACCTATCGAATGTGCTAGCCCTGAAAATGGATGGCGCTCAAGCGTCCCACCCATACC

TCGCCCTCAGGGTAGAAACGATGCCCTGAGGAGTAGGCGGCCGTGGGNNNNNNNNNNNNN

NNNNNNNNN

>Entalbostroma_erumpens_ICMP_21152

------------------------------------------------------------

------------------------------------------------------------

------------------------------------------------------------

------------------------------------------------------------

------------------------------------------------------------

------------------------------------------------------------

------------------------------------------------------------

------------------------------------------------------------

------------------------------------------------------------

------------------------------------------------------------

------------------------------------------------------------

------------------------------------------------------------

------------------------------------------------------------

------------------------------------------------------------

------------------------------------------------------------

------------------------------------------------------------

------------------------------------------------------------

------------------------------------------------------------

------------------------------------------------------------

------------------------------------------------------------

------------------------------------------------------------

------------------------------------------------------------

---------

>Induratia_apiospora_ATCC_60639

------------------------------------------------------------

------------------------------------------------------------

-----------ACGGGACGCCTTAGAGGGTGAGAGCCCCGTACGGTTGGACACTAAGCCT

TTGTAAAGCTCCTTCGACGAGTCGAGTAGTTTGGGAATGCTGCTCTAAATGGGAGGTAAA

TTTCTTCTAAAGCTAAATACCGGCCAGAGACCGATAGCGCACAAGTAGAGTGATCGAAAG

ATGAAAAGCACTTTGAAAAGAGGGTTAAATAGCACGTGAAATTGTTGAAAGGGAAGCGTT

TACGACCAGACTTTCTCTAGGTGGATCATCCGGTGTTCTCACTGGTGCACTTCGCCTAGT

TTAGGCCAGCATCGGTTTTCGTAGGGGGATAAAAGCTTGGGGAAAGTAGCTCTCTCGGGA

GTGTTATAGCCCTTTGTATAATACCTTTATGGGGACCGAGGATCGCGCTCTGCAAGGATG

CTGGCATAATGGTCGTCAACGACCCGTCTTGAAACACGGACCAAGGAGTCGAACATTTGT

GCGAGTGTTTGGGTGTTAAACCCTCACGCGTAATGAAAGTGAACGGAGGTGAGAGCCTTT

ACGGGTGCATCATCGACCGATCCTGATGTCTTCGGATGGATTTGAGTAAGAGCATAACTG

TTCGGACCCGAAAGATGGTGAACTATGCGTGGATAGGGTGAAGCCAGAGGAAACTCTGGT

GGAGGCTCGCAGCGGTTCTGACGTGCAAATCGATCGTCAAATCTGCGCATGGGGGCGAAA

GACTAATCGAACCAT---------------------------------------------

------------------------------------------------------------

------------------------------------------------------------

------------------------------------------------------------

------------------------------------------------------------

------------------------------------------------------------

------------------------------------------------------------

------------------------------------------------------------

---------

>Barrmaelia_rhamnicola_BR

AGAAACCAACAGGGATTGCCCTAGTAACGGCGAGTGAAGCGGCAACAGCTCAAATTTGAA

ATCTGGCTCTCGGGTCCGAATTGTAATTTGTAGAGGATGCTTTTGGCGCGGTGCCTTCCG

AGTTCCCTGGAACGGGACGCCTTAGAGGGTGAGAGCCCCGTACGGTTGGACACCAAGCCT

CTGTAAAGCTCCTTCGACGAGTCGAGTAGTTTGGGAATGCTGCTCTAAATGGGAGGTAAA

TTTCTTCTAAAGCTAAATACCGGCCAGAGACCGATAGCGCACAAGTAGAGTGATCGAAAG

ATGAAAAGTACTTTGAAAAGAGGGTTAAATAGTACGTGAAATTGTTGAAAGGGAAGCGTT

TACGACCAGACTTTTCCCAGGCGGATCATCCGGTGTTCTCACCGGTGCACTTCGCCTGGT

TTAGGCCAGCATCGGTTTTCGTAGGGGGATAAAAGCTTCGGGAAAGTAGCTCCCTCGGGA

GTGTTATAGCCCGTCGTATAATACCCTTATGGGGACCGAGGATCGCGCTCTGCAAGGATG

CTGGCATAATGGTCGTCAACGACCCGTCTTGAAACACGGACCAAGGAGTCGAACATTTGT

GCGAGTGTTTGGGTGTTAAACCCTCACGCGTAATGAAAGTGAACGGAGGTGAGAGCCCTT

ACGGGTGCATCATCGACCGATCCTGATGTCTTCGGATGGATTTGAGTAAGAGCATAACTG

TTCGGACCCGAAAGATGGTGAACTATGCGTGGATAGGGTGAAGCCAGAGGAAACTCTGGT

GGAGGCTCGCAGCGGTTCTGACGTGCAAATCGATCGTCAAATCTGCGCATGGGGGCGAAA

GACTAATCGAACCAT---------------------------------------------

------------------------------------------------------------

------------------------------------------------------------

------------------------------------------------------------

------------------------------------------------------------

------------------------------------------------------------

------------------------------------------------------------

------------------------------------------------------------

---------

>Barrmaelia_macrospora_CBS_142768

AGAAACCAACAGGGATTGCCCTAGTAACGGCGAGTGAAGCGGCAACAGCTCAAATTTGAA

ATCTGGCTCTCGGGTCCGAATTGTAATTTGTAGAGGATGCTTTTGGCGCGGTGCCTTCCG

AGTTCCCTGGAACGGGACGCCTTAGAGGGTGAGAGCCCCGTACGGTTGGACACCAAGCCT

CTGTAAAGCTCCTTCGACGAGTCGAGTAGTTTGGGAATGCTGCTCTAAATGGGAGGTAAA

TTTCTTCTAAAGCTAAATACCGGCCAGAGACCGATAGCGCACAAGTAGAGTGATCGAAAG

ATGAAAAGTACTTTGAAAAGAGGGTTAAATAGTACGTGAAATTGTTGAAAGGGAAGCGTT

TACGACCAGACTTTTCCCAGGCGGATCATCCGGTGTTCTCACCGGTGCACTTCGCCTGGT

TTAGGCCAGCATCGGTTTTCGTAGGGGGATAAAAGCTTCGGGAAAGTAGCTCCTCCGGGA

GTGTTATAGCCCGTTGCATAATACCCTTATGGGGACCGAGGATCGCGCTCTGCAAGGATG

CTGGCATAATGGTCGTCAACGACCCGTCTTGAAACACGGACCAAGGAGTCGAACATTTGT

GCGAGTGTTTGGGTGTTAAACCCTCACGCGTAATGAAAGTGAACGGAGGTGAGAGCCCTT

ACGGGTGCATCATCGACCGATCCTGATGTCTTCGGATGGATTTGAGTAAGAGCATAACTG

TTCGGACCCGAAAGATGGTGAACTATGCGTGGATAGGGTGAAGCCAGAGGAAACTCTGGT

GGAGGCTCGCAGCGGTTCTGACGTGCAAATCGATCGTCAAATCTGCGCATGGGGGCGAAA

GACTAATCGAACCAT---------------------------------------------

------------------------------------------------------------

------------------------------------------------------------

------------------------------------------------------------

------------------------------------------------------------

------------------------------------------------------------

------------------------------------------------------------

------------------------------------------------------------

---------

>Barrmaelia_rappazii_CBS_142771

AGAAACCAACAGGGATTGCCCTAGTAACGGCGAGTGAAGCGGCAACAGCTCAAATTTGAA

ATCTGGCTCTCGGGTCCGAATTGTAATTTGTAGAGGATGCTTTTGGCGCGGTGCCTTCCG

AGTTCCCTGGAACGGGACGCCTTAGAGGGTGAGAGCCCCGTACGGTTGGACACCAAGCCT

CTGTAAAGCTCCTTCGACGAGTCGAGTAGTTTGGGAATGCTGCTCTAAATGGGAGGTAAA

TTTCTTCTAAAGCTAAATACCGGCCAGAGACCGATAGCGCACAAGTAGAGTGATCGAAAG

ATGAAAAGTACTTTGAAAAGAGGGTTAAATAGTACGTGAAATTGTTGAAAGGGAAGCGTT

TACGACCAGACTTTTCCCAGGCGGATCATCCGGTGTTCTCACCGGTGCACTTCGCCTGGT

TTAGGCCAGCATCGGTTCTCGTAGGGGGATAAAAGCTTCGGGAAAGTAGCTCCCTCGGGA

GTGTTATAGCCCGTTGCATAATACCCTTATGGGGACCGAGGATCGCGCTCTGCAAGGATG

CTGGCATAATGGTCGTCAACGACCCGTCTTGAAACACGGACCAAGGAGTCGAACATTTGT

GCGAGTGTTTGGGTGTTAAACCCTCACGCGTAATGAAAGTGAACGGAGGTGAGAGCCCTT

ACGGGTGCATCATCGACCGATCCTGATGTCTTCGGATGGATTTGAGTAAGAGCATAACTG

TTCGGACCCGAAAGATGGTGAACTATGCGTGGATAGGGTGAAGCCAGAGGAAACTCTGGT

GGAGGCTCGCAGCGGTTCTGACGTGCAAATCGATCGTCAAATCTGCGCATGGGGGCGAAA

GACTAATCGAACCAT---------------------------------------------

------------------------------------------------------------

------------------------------------------------------------

------------------------------------------------------------

------------------------------------------------------------

------------------------------------------------------------

------------------------------------------------------------

------------------------------------------------------------

---------

>Barrmaelia_oxyacanthae_CBS_142770

AGAAACCAACAGGGATTGCCCTAGTAACGGCGAGTGAAGCGGCAACAGCTCAAATTTGAA

ATCTGGCTCTCGGGTCCGAATTGTAATTTGTAGAGGATGCTTTTGGCGCGGTGCCTTCCG

AGTTCCCTGGAACGGGACGCCTTAGAGGGTGAGAGCCCCGTACGGTTGGACACCAAGCCT

CTGTAAAGCTCCTTCGACGAGTCGAGTAGTTTGGGAATGCTGCTCTAAATGGGAGGTAAA

TTTCTTCTAAAGCTAAATACCGGCCAGAGACCGATAGCGCACAAGTAGAGTGATCGAAAG

ATGAAAAGTACTTTGAAAAGAGGGTTAAATAGTACGTGAAATTGTTGAAAGGGAAGCGTT

TACGACCAGACTTTTCCCAGGCGGATCATCCGGTGTTCTCACCGGTGCACTTCGCCTGGT

TTAGGCCAGCATCGGTTTTCGTAGGGGGATAAAAGCTTCGGGAAAGTAGCTCCCTCGGGA

GTGTTATAGCCCGTTGCATAATACCCTTATGGGGACCGAGGATCGCGCTCTGCAAGGATG

CTGGCATAATGGTCGTCAACGACCCGTCTTGAAACACGGACCAAGGAGTCGAACATTTGT

GCGAGTGTTTGGGTGTTAAACCCTCACGCGTAATGAAAGTGAACGGAGGTGAGAGCCCTT

ACGGGTGCATCATCGACCGATCCTGATGTCTTCGGATGGATTTGAGTAAGAGCATAACTG

TTCGGACCCGAAAGATGGTGAACTATGCGTGGATAGGGTGAAGCCAGAGGAAACTCTGGT

GGAGGCTCGCAGCGGTTCTGACGTGCAAATCGATCGTCAAATCTGCGCATGGGGGCGAAA

GACTAATCGAACCAT---------------------------------------------

------------------------------------------------------------

------------------------------------------------------------

------------------------------------------------------------

------------------------------------------------------------

------------------------------------------------------------

------------------------------------------------------------

------------------------------------------------------------

---------

>Barrmaelia_moravica_CBS_142769

AGAAACCAACAGGGATTGCCCTAGTAACGGCGAGTGAAGCGGCAACAGCTCAAATTTGAA

ATCTGGCTCTCGGGTCCGAATTGTAATTTGTAGAGGATGCTTTTGGCGCGGTGCCTTCCG

AGTTCCCTGGAACGGGACGCCTCAGAGGGTGAGAGCCCCGTACGGTTGGACACCAAGCCT

CTGTAAAGCTCCTTCGACGAGTCGAGTAGTTTGGGAATGCTGCTCTAAATGGGAGGTAAA

TTTCTTCTAAAGCTAAATACCGGCCAGAGACCGATAGCGCACAAGTAGAGTGATCGAAAG

ATGAAAAGTACTTTGAAAAGAGGGTTAAATAGTACGTGAAATTGTTGAAAGGGAAGCGTT

TACGACCAGACTTTTCCCAGGCGGATCATCCGGTGTTCTCACCGGTGCACTTCGCCTGGT

TTAGGCCAGCATCGGTTTTCGTAGGGGGATAAAAGCTTCGGGAAAGTAGCTCCCTCGGGA

GTGTTATAGCCTGTTGCATAATACCCTTATGGGGACCGAGGATCGCGCTCTGCAAGGATG

CTGGCATAATGGTCGTCAACGACCCGTCTTGAAACACGGACCAAGGAGTCGAACATTTGT

GCGAGTGTTTGGGTGTTAAACCCTCACGCGTAATGAAAGTGAACGGAGGTGAGAGCCCTT

ACGGGTGCATCATCGACCGATCCTGATGTCTTCGGATGGATTTGAGTAAGAGCATAACTG

TTCGGACCCGAAAGATGGTGAACTATGCGTGGATAGGGTGAAGCCAGAGGAAACTCTGGT

GGAGGCTCGCAGCGGTTCTGACGTGCAAATCGATCGTCAAATCTGCGCATGGGGGCGAAA

GACTAATCGAACCAT---------------------------------------------

------------------------------------------------------------

------------------------------------------------------------

------------------------------------------------------------

------------------------------------------------------------

------------------------------------------------------------

------------------------------------------------------------

------------------------------------------------------------

---------

>Entosordaria_perfidiosa_CBS_142773

AGAAACCAACAGGGATTGCCCTAGTAACGGCGAGTGAAGCGGCAACAGCTCAAATTTGAA

ATCTGGCCCTCGGGTCCGAATTGTAATTTGTAGAGGATGCTTTTGGCGCGGTGCCTTCCG

AGTTCCCTGGAACGGGACGCCTTAGAGGGTGAGAGCCCCGTACGGTTGGACACCAAGCCT

CTGTAAAGCTCCTTCGACGAGTCGAGTAGTTTGGGAATGCTGCTCTAAATGGGAGGTAAA

TTTCTTCTAAAGCTAAATACCGGCCAGAGACCGATAGCGCACAAGTAGAGTGATCGAAAG

ATGAAAAGCACTTTGAAAAGAGGGTTAAATAGCACGTGAAATTGTTGAAAGGGAAGCGTT

TACGACCAGACTTTTTCCAGGCGGATCATCCGGTGTTCTCACCGGTGCACTTCGCCTGGT

TTAGGCCAGCATCGGTTTTCGTAGGGGGATAAAAGCTTCGGGAAAGTAGCTCCCTCGGGA

GTGTTATAGCCCGTTGCATAATACCCTTATGGGGACCGAGGATCGCGCTCTGCAAGGATG

CTGGCATAATGGTCGTCAACGACCCGTCTTGAAACACGGACCAAGGAGTCGAACATTTGT

GCGAGTGTTTGGGTGTTAAACCCTCACGCGTAATGAAAGTGAACGGAGGTGAGAGCCCTT

ATGGGTGCATCATCGACCGATCCTGATGTCTTCGGATGGATTTGAGTAAGAGCATAACTG

TTCGGACCCGAAAGATGGTGAACTATGCGTGGATAGGGTGAAGCCAGAGGAAACTCTGGT

GGAGGCTCGCAGCGGTTCTGACGTGCAAATCGATCGTCAAATCTGCGCATGGGGGCGAAA

GACTAATCGAACCAT---------------------------------------------

------------------------------------------------------------

------------------------------------------------------------

------------------------------------------------------------

------------------------------------------------------------

------------------------------------------------------------

------------------------------------------------------------

------------------------------------------------------------

---------

>Entosordaria_quercina_CBS_142774

AGAAACCAACAGGGATTGCCCTAGTAACGGCGAGTGAAGCGGCAACAGCTCAAATTTGAA

ATCTGGCCCTCGGGTCCGAATTGTAATTTGTAGAGGATGCTTTTGGCGCGGTGCCTTCCG

AGTTCCCTGGAACGGGACGCCTTAGAGGGTGAGAGCCCCGTACGGTTGGACACCAAGCCT

CTGTAAAGCTCCTTCGACGAGTCGAGTAGTTTGGGAATGCTGCTCTAAATGGGAGGTAAA

TTTCTTCTAAAGCTAAATACCGGCCAGAGACCGATAGCGCACAAGTAGAGTGATCGAAAG

ATGAAAAGCACTTTGAAAAGAGGGTTAAATAGCACGTGAAATTGTTGAAAGGGAAGCGTT

TACGACCAGACTTTTTCCAGGCGGATCATCCGGTGTTCTCACCGGTGCACTTCGCCTGGT

TTAGGCCAGCATCGGTTTTCGTGGGGGGATAAAAGCTTCGGGAAAGTAGCTCCCTCGGGA

GTGTTATAGCCCGTTGCATAATACCCCTGTGGGGACCGAGGATCGCGCTCTGCAAGGATG

CTGGCGTAATGGTCGTCAACGACCCGTCTTGAAACACGGACCAAGGAGTCGAACATTTGT

GCGAGTGTTTGGGTGTTAAACCCTCACGCGTAATGAAAGTGAACGGAGGTGAGAGCCCTT

ACGGGTGCATCATCGACCGATCCTGATGTCTTCGGATGGATTTGAGTAAGAGCATAACTG

TTCGGACCCGAAAGATGGTGAACTATGCGTGGATAGGGTGAAGCCAGAGGAAACTCTGGT

GGAGGCTCGCAGCGGTTCTGACGTGCAAATCGATCGTCAAATCTGCGCATGGGGGCGAAA

GACTAATCGAACCAT---------------------------------------------

------------------------------------------------------------

------------------------------------------------------------

------------------------------------------------------------

------------------------------------------------------------

------------------------------------------------------------

------------------------------------------------------------

------------------------------------------------------------

---------

>Xylaria_apoda_HAST_90080804

------------------------------------------------------------

------------------------------------------------------------

------------------------------------------------------------

------------------------------------------------------------

------------------------------------------------------------

------------------------------------------------------------

------------------------------------------------------------

------------------------------------------------------------

------------------------------------------------------------

------------------------------------------------------------

------------------------------------------------------------

------------------------------------------------------------

------------------------------------------------------------

------------------------------------------------------------

------------------------------------------------------------

------------------------------------------------------------

------------------------------------------------------------

------------------------------------------------------------

------------------------------------------------------------

------------------------------------------------------------

------------------------------------------------------------

------------------------------------------------------------

---------

>Nemania_primolutea_HAST_91102001

------------------------------------------------------------

------------------------------------------------------------

------------------------------------------------------------

------------------------------------------------------------

------------------------------------------------------------

------------------------------------------------------------

------------------------------------------------------------

------------------------------------------------------------

------------------------------------------------------------

------------------------------------------------------------

------------------------------------------------------------

------------------------------------------------------------

------------------------------------------------------------

------------------------------------------------------------

------------------------------------------------------------

------------------------------------------------------------

------------------------------------------------------------

------------------------------------------------------------

------------------------------------------------------------

------------------------------------------------------------

------------------------------------------------------------

------------------------------------------------------------

---------

>Nemania_uda_CBS_148422

AGAAACCAACAGGGATTGCCCTAGTAACGGCGAGTGAAGCGGCAACAGCTCAAATTTGAA

ATCTGGCCCCCGGGTCCGAGTTGTAATTTGCAGAGGATGCTTTTGGCGCGGTGCCTTCCG

AGTTCCCTGGAACGGGACGCCTTAGAGGGTGAGAGCCCCGTACGGTTGGACACCAAGCCT

CTGTAAAGCTCCTTCGACGAGTCGAGTAGTTTGGGAATGCTGCTCTAAATGGGAGGTAAA

TTTCTTCTAAAGCTAAATATTGGCCAGAGACCGATAGCGCACAAGTAGAGTGATCGAAAG

ATGAAAAGCACTTTGAAAAGAGGGTTAAACAGCACGTGAAATTGTTGAAAGGGAAGCGTT

TGCGACCAGACCTTTTCCTGGCGGATCATCCGGTGTTTCCACCGGTGCACTTCGCCAGGT

CGAGGCCAGCATCGGTCTCTGTAGGGGGATAAAAGCCCAGGGAACGTAGCTCCTCAGGGA

GTGTTATAGCCCTCCGCATAATACCCTTATGGGGACCGAGGACCGCGCCTTGCAAGGATG

CTGGCGTAATGGTTGTCAACGACCCGTCTTGAAACACGGACCAAGGAGTCGAACATTTGT

GCGAGTGTTTGGGTGTTAAACCCTCACGCGTAATGAAAGTGAACGGAGGTGAGAGCCCTT

ACGGGTGCATCATCGACCGATCCTGATGTCTTCGGATGGATTTGAGTAAGAGCATAACTG

TTCGGACCCGAAAGATGGTGAACTATGCGTGGATAGGGTGAAGCCAGAGGAAACTCTGGT

GGAGGCTCGCAGCGGTTCTGACGTGCAAATCGATCGTCAAATCTGCGCATGGGGGCGAAA

GACTTATCGAACCATCTAGTAGCTGGTTACCGC---------------------------

------------------------------------------------------------

------------------------------------------------------------

------------------------------------------------------------

------------------------------------------------------------

------------------------------------------------------------

------------------------------------------------------------

------------------------------------------------------------

---------

>Nemania_ethancrensonii_CBS_148337

AGAAACCAACAGGGATTGCCCTAGTAACGGCGAGTGAAGCGGCAACAGCTCAAATTTGAA

ATCTGGCCCTCGGGTCCGAGTTGTAATTTGCAGAGGATGCTTTTGGCGCGGTGCCTTCCG

AGTTCCCTGGAACGGGACGCCTTAGAGGGTGAGAGCCCCGTACGGTTGGACACCAAGCCT

CTGTAAAGCTCCTTCGACGAGTCGAGTAGTTTGGGAATGCTGCTCTAAATGGGAGGTAAA

TTTCTTCTAAAGCTAAATATTGGCCAGAGACCGATAGCGCACAAGTAGAGTGATCGAAAG

ATGAAAAGCACTTTGAAAAGAGGGTTAAACAGCACGTGAAATTGTTGAAAGGGAAGCGTT

TGCGACCAGACCTTTTCCTGGAGGATCATCCGGTGTTTCCACCGGTGCACTTCGCCAGGT

CGAGGCCAGCATCGGTTTTCGCGGGGGGACAAAAGCTCTGGGAACGTAGCTCCTCCGGGA

GTGTTATAGCCCTCTGCATAATGCCCTCGCGGGGACCGAGGACCGCGCCTTGCAAGGATG

CTGGCGTAATGGTCGTCAACGACCCGTCTTGAAACACGGACCAAGGAGTCGAACATTTGT

GCGAGTGTTTGGGTGTCAAACCCTCACGCGTAATGAAAGTGAACGGAGGTGAGAGCCCTT

TCGGGTGCATCATCGACCGATCCTGATGTCTTCGGATGGATTTGAGTAAGAGCATAACTG

TTCGGACCCGAAAGATGGTGAACTATGCGTGGATAGGGTGAAGCCAGAGGAAACTCTGGT

GGAGGCTCGCAGCGGTTCTGACGTGCAAATCGATCGTCAAATCTGCGCATGGGGGCGAAA

GACTTATCGAACCATCTAGTAGCTGGTTACCGC---------------------------

------------------------------------------------------------

------------------------------------------------------------

------------------------------------------------------------

------------------------------------------------------------

------------------------------------------------------------

------------------------------------------------------------

------------------------------------------------------------

---------

>Clypeosphaeria_mamillana_CBS_140735

AGAAACCAACAGGGATTGCCCTAGTAACGGCGAGTGAAGCGGCAACAGCTCAAATTTGAA

ATCTGGCCCTAGGGTCCGAGTTGTAATTTGTAGAGGATGCTTTTGGCGCGGTGCCTTCCG

AGTTCCCTGGAACGGGACGCCTTAGAGGGTGAGAGCCCCGTACGGTTGGACACCAAGCCT

CTGTAAAGCTCCTTCGACGAGTCGAGTAGTTTGGGAATGCTGCTCTAAATGGGAGGTAAA

TTTCTTCTAAAGCTAAATACCGGCCAGAGACCGATAGCGCACAAGTAGAGTGATCGAAAG

ATGAAAAGCACTTTGAAAAGAGGGTTAAATAGCACGTGAAATTGTTGAAAGGGAAGCGTT

TGCGACCAGACCTTTTCCTAGCGGATCATCCGGTGTTTTCACCGGTGCACTTCGCTAGGT

TTAGGCCAGCATCGGTTTCTGTGGGGGGATAAAAGCTCGGGGAACGTGGCTCCCTCGGGA

GTGTTATAGCCCCTTGCATAATACCCTCACGGGGACCGAGGACCGCGCTTTGCAAGGATG

CTGGCATAATGGTCGTCAACGACCCGTCTTGAAACACGGACCAAGGAGTCGAACATTTGT

GCGAGTGTTTGGGTGTTAAACCCTCACGCGTAATGAAAGTGAACGGAGGTGAGAGCCCTT

ACGGGTGCATCATCGACCGATCCTGATGTCTTCGGATGGATTTGAGTAAGAGCATAACTG

TTCGGACCCGAAAGATGGTGAACTATGCGTGGATAGGGTGAAGCCAGAGGAAACTCTGGT

GGAGGCTCGCAGCGGTTCTGACGTGCAAATCGATCGTCAAATCTGCGCATGGGGCGGAAA

GACTAATC----------------------------------------------------

------------------------------------------------------------

------------------------------------------------------------

------------------------------------------------------------

------------------------------------------------------------

------------------------------------------------------------

------------------------------------------------------------

------------------------------------------------------------

---------

>Digitodochium_amoenum_CBS_147285

AGAAACCAACAGGGATTGCCCTAGTAACGGCGAGTGAAGCGGCAACAGCTCAAATTTGAA

ATCTGGCCTTCGGGCCCGAGTTGTAATTTGTAGAGGATGATTTTGGCGCGGTGCCTTCCG

AGTTCCCTGGAACGGGACGCCTTAGAGGGTGAGAGCCCCGTACGGTTGGACGCTAAGCCT

CTGTAAATCTCCTTCGACGAGTCGAGTAGTTTGGGAATGCTGCTCTAAATGGGAGGTAAA

TTTCTTCTAAAGCTAAATACCGGCCAGAGACCGATAGCGCACAAGTAGAGTGATCGAAAG

ATGAAAAGCACTTTGAAAAGAGGGTTAAATAGCACGTGAAATTGTTGAAAGGGAAGCGTT

TACGACCAGACCTTTTCCTAGCGGATCATCCGGTGTTCTCACCGGTGCACTTCGCTAGGC

TTAGGCCAGCATCGGTTTCTATAGGGGGATAAAAGCTTAGGGAATGTGGCTCCTTCGGGA

GTGTTATAGCCCTCTGCATAATACCCTTACGGGGACCGAGGACCGCGCTTTGCAAGGATG

CTGGCATAATGGTCGTCAACGACCCGTCTTGAAACACGGACCAAGGAGTCGAACATTTGT

GCGAGTGTTTGGGTGTCAAACCCTCACGCGTAATGAAAGTGAACGGAGGTGAGAGCCCTT

ACGGGTGCATCATCGACCGATCCTGATGTCTTCGGATGGATTTGAGTAAGAGCATAACTG

TTCGGACCCGAAAGATGGTGAACTATGCGTGGATAGGGTGAAGCCAGAGGAAACTCTGGT

GGAGGCTCGCAGCGGTTCTGACGTGCAAATCGATCGTCAAATCTGCGCATGGGGGCGAAA

GACTTATCGAACCATCTAGTAGC-------------------------------------

------------------------------------------------------------

------------------------------------------------------------

------------------------------------------------------------

------------------------------------------------------------

------------------------------------------------------------

------------------------------------------------------------

------------------------------------------------------------

---------

>Occultitheca_rosae_HKAS_102393

------------------------------------------------------------

------------------------------------------------------------

------------------------------------------------------------

--------CTCCTTCGACGAGTCGAGTAGTTTGGGAATGCTGCTCTAAATGGGAGGTAAA

TTTCTTCTAAAGCTAAATACCGGCCAGAGACCGATAGCGCACAAGTAGAGTGATCGAAAG

ATGAAAAGCACTTTGAAAAGAGGGTTAAATAGCACGTGAAATTGTTGAAAGGGAAGCGTT

TGCGACCAGACCTTTTCCTAGCGGATCATCCGGTGTTCTCACCGGTGCACTTCGCTTGGC

TTAGGCCAGCATCGGTTTCTGTAGGGGGATAAAAGCTCTGGGAATGTGGCTCCCTCGGGA

GTGTTATAGCCCTCTGCATAATACCCTTACGGGGACCGAGGACCGCGCTTCGCAAGGATG

CTGGCATAATGGTTGTCAACGACCCGTCTTGAAACACGGACCAAGGAGTCGAACATTTGT

GCGAGTGTTTGGGTGTTAAACCCTCACGCGTAATGAAAGTGAACGGAGGTGAGAGCCCTT

TGGGGTGCATCATCGACCGATCCTGATGTCTTCGGATGGATTTGAGTAAGAGCATAACTG

TTCGGACCCGAAAGATGGTGAACTATGCGTGGATAGGGTGAAGCCAGAGGAAACTCTGGT

GGAGGCTCGCAGCGGTTCTGACGTGCAAATCGATCGTCAAATCTGCGCATGGGGGCGAAA

GACTTATCGAACCAT---------------------------------------------

------------------------------------------------------------

------------------------------------------------------------

------------------------------------------------------------

------------------------------------------------------------

------------------------------------------------------------

------------------------------------------------------------

------------------------------------------------------------

---------

>Magnostiolata_mucida_MFLU_19_2133

AGAAACCAACAGGGATTGCCCTAGTAACGGCGAGTGAAGCGGCAACAGCTCAAATTTGAA

ATCTGGCCCTCGGGTCCGAGTTGTAATTTGTAGAGGATGCTTTTGGCGCGGTGCCTTCCG

AGTTCCCTGGAACGGGACGCCTTAGAGGGTGAGAGCCCCGTACGGTTGGACACCAAGCCT

CTGTAAAGCTCCTTCGACGAGTCGAGTAGTTTGGGAATGCTGCTCTAAATGGGAGGTAAA

TTTCTTCTAAAGCTAAATACCGGCCAGAGACCGATAGCGCACAAGTAGAGTGATCGAAAG

ATGAAAAGCACTTTGAAAAGAGGGTTAAATAGCACGTGAAATTGTTGAAAGGGAAGCGTT

TGCGACCAGACCTCTTCCCAGCGGATCATCCGGTGTTCTCACCGGTGCACTTCGCTGGGT

TTAGGCCAGCATCGGTTTCCGCCGGGGGATAAAAGTTAGGGGAACGTAGCTCCCTCGGGA

GTGTTATAGCCCCTTTCATAATACCCCGTCGGGGACCGAGGACCGCGCTTCGCAAGGATG

CTGGCATAATGGTCGTCAACGACCCGTCTTGAAACACGGACCAAGGAGTCGAACATTTGT

GCGAGTGTTTGGGTGTTAAACCCTCACGCGTAATGAAGGTGAACGTAGGTGAGAGCCCTT

GCGGGCGCATCATCGACCGATCCTGATGTCTTCGGATGGATTTGAGTAAGAGCATAACTG

TTCGGACCCGAAAGATGGTGAACTATGCGTGGATAGGGTGAAGCCAGAGGAAACTCTGGT

GGAGGCTCGCAGCGGTTCTGACGTGCAAATCGATCGTCAAATCTGCGCATGGGGGCGAAA

GACTTATCGAACCATCTA------------------------------------------

------------------------------------------------------------

------------------------------------------------------------

------------------------------------------------------------

------------------------------------------------------------

------------------------------------------------------------

------------------------------------------------------------

------------------------------------------------------------

---------

>Anthostomelloides_krabiensis_MFLUCC_15_0678

AGAAACCAACAGGGATTGCCCTAGTAACGGCGAGTGAAGCGGCAACAGCTCAAATTTGAA

ATCTGGCCCTCGGGTCCGAGTTGTAATTTGTAGAGGATGCTTTTGGCGCGGTGCCTTCCG

AGTTCCCTGGAACGGGACGCCTTAGAGGGTGAGAGCCCCGTACGGTTGGACACCAAGCCT

CTGTAAAGCTCCTTCGACGAGTCGAGTAGTTTGGGAATGCTGCTCTAAATGGGAGGTAAA

TTTCTTCTAAAGCTAAATACCGGCCAGAGACCGATAGCGCACAAGTAGAGTGATCGAAAG

ATGAAAAGCACTTTGAAAAGAGGGTTAAATAGCACGTGAAATTGTTGAAAGGGAAGCGTT

TGCGACCAGACCTCTGCCTGGCGGATCATCCGGTGTTCTCACCGGTGCACTTCGCCTGGC

TTAGGCCAGCATCGGTTTCCGGGGGGGGATAAAGGCTCTGGGAATGTAGCTCCCTCGGGA

GTGTTATAGCCCTCTGCGTAATACCCTCCCGGGGACCGAGGACCGCGCTTTGCAAGGATG

CTGGCATAATGGTCGTCAACGACCCGTCTTGAAACACGGACCAAGGAGTCGAACATTTGT

GCGAGTGTTTGGGTGTCAAACCCTCACGCGTAATGAAAGTGAACGGAGGTGAGAGCCCTT

GCGGGCGCATCATCGACCGATCCTGATGTCTTCGGATGGATTTGAGTAAGAGCATAACTG

TTCGGACCCGAAAGATGGTGAACTATGCGTGGATAGGGTGAAGCCAGAGGAAACTCTGGT

GGAGGCTCGCAGCGGTTCTGACGTGCAAATCGATCGTCAAATCTGCGCATGGGGGCGAAA

GACTTATCGAACCATCTAGTAGCTGGTTACCGC---------------------------

------------------------------------------------------------

------------------------------------------------------------

------------------------------------------------------------

------------------------------------------------------------

------------------------------------------------------------

------------------------------------------------------------

------------------------------------------------------------

---------

>Linosporopsis_ischnotheca_CBS_145761

AGAAACCAACAGGGATTGCCTCAGTAACGGCGAGTGAAGCGGCAACAGCTCAAATTTGAA

ATCTGGCCCTCGGGTCCGAGTTGTAATTTGCAGAGGATACTTTTGGCGCGGTGCCTTCCG

AGTTCCCTGGAACGGGACGCCTTAGAGGGTGAGAGCCCCGTACGGTCGGACACCAAGCCT

CTGTAAAGTTCCTTCGACGAGTCGAGTAGTTTGGGAATGCTGCTCTAAATGGGAGGTATA

TTTCTTCTAAAGCTAAATACCGGCCAGAGACCGATAGCGCACAAGTAGAGTGATCGAAAG

ATGAAAAGCACTTTGAAAAGAGGGTTAAATAGCACGTGAAATTGTTGAAAGGGAAGCGTT

TGCGACCAGACCTTCGCCCGGCGGATCATCCGGCGTTCTCGCCGGTGCACTTCGCCGGGC

CTAGGCCAGCATCGGTCTCTGCAGGGGGATAAAAGCTCTGGGAACGTGGCTCCTCCGGGA

GTGTTATAGCCCTCTGCATAATACCCTTGCGGGGACCGAGGACCGCGCTTCGCAAGGATG

CTGGCATAATGGTCGTCAACGACCCGTCTTGAAACACGGACCAAGGAGTCGAACATTTAT

GCGAGTGTTTGGGTGTCAAACCCTTACGCGCAATGAAAGTGAACGGAGGTGGGAGCCCCC

CGGGGCGCACCATCGACCGATCCTGATGTCTTCGGATGGATTTGAGTAAGAGCATAACTG

TTCGGACCCGAAAGATGGTGAACTATGCGTGGATAGGGTGAAGCCAGAGGAAACTCTGGT

GGAGGCTCGCAGCGGTTCTGACGTGCAAATCGATCGTCAAATCTGCGCATGGGGGCGAAA

GACTTATCGAACCATCTAGTAGCTGGTTACCGC---------------------------

--NNNNNNNNNNNNNNNNNNNNNNNNNNNNNNNNNNNNNNNNNNNNNNNNNNNNNNNNNN

NNNNNNNNNNNNNNNNNNNNNNNNNNNNNNNNNNNNNNNNNNNNNNNNNNNNNNNNNNNN

NNNNNNNNNNNNNNNNNNNNNNNNNNNNNNNNNNNNNNNNNNNNNNNNNNNNNNNNNNNN

NNNNNNNNNNNNNNNNNNNNNNNNNNNNNNNNNNNNNNNNNNNNNNNNNNNNNNNNNNNN

NNNNNNNNNNNNNNNNNNNNNNNNNNNNNNNNNNNNNNNNNNNNNNNNNNNNNNNNNNNN

NNNNNNNNNNNNNNNNNNNNNNNNNNNNNNNNNNNNNNNNNNNNNNNNNNNNNNNNNNNN

NNNNNNNNNNNNNNNNNNNNNNNNNNNNNNNNNNNNNNNNNNNNNNNNNNNNNNNNNNNN

NNNNNNNNN

>Linosporopsis_ochracea_CBS_145999

AGAAACCAACAGGGATTGCCCTAGTAACGGCGAGTGAAGCGGCAACAGCTCAAATTTGAA

ATCTGGCCCTCGGGTCCGAGTTGTAATTTGTAGAGGATACTTTTGGCGCGGTGCCTTCCG

AGTTCCCTGGAACGGGACGCCTTAGAGGGTGAGAGCCCCGTACGGTTGGACACCAAGCCT

CTGTAAAGTTCCTTCGACGAGTCGAGTAGTTTGGGAATGCTGCTCTAAATGGGAGGTATA

TTTCTTCTAAAGCTAAATACTGGCCAGAGACCGATAGCGCACAAGTAGAGTGATCGAAAG

ATGAAAAGCACTTTGAAAAGAGGGTTAAATAGCACGTGAAATTGTTGAAAGGGAAGCGTT

TGCGACCAGACCTTTTCCCGGCGGATCATCCGGCGTTCTCGCCGGTGCACTTCGCCGGGC

TTAGGCCAGCATCGGTCTCTGCAGGGGGATAAAAGCTCTGGGAACGTGGCTCCTCCGGGA

GTGTTATAGCCCTCTGCATAATACCCTTGCGGGGACCGAGGACCGCGCTTCGCAAGGATG

CTGGCATAATGGTCGTCAACGACCCGTCTTGAAACACGGACCAAGGAGTCGAACATTTGT

GCGAGTGTTTGGGTGTCAAACCCTCACGCGTAATGAAAGTGAACGGAGGTGGGAGCCTTT

A--GGCGCACCATCGACCGATCCTGATGTCTTCGGATGGATTTGAGTAAGAGCATAACTG

TTCGGACCCGAAAGATGGTGAACTATGCGTGGATAGGGTGAAGCCAGAGGAAACTCTGGT

GGAGGCTCGCAGCGGTTCTGACGTGCAAATCGATCGTCAAATCTGCGCATGGGGGCGAAA

GACTTATCGAACCATCTAGTAGCTGGTTACCGC---------------------------

--NNNNNNNNNNNNNNNNNNNNNNNNNNNNNNNNNNNNNNNNNNNNNNNNNNNNNNNNNN

NNNNNNNNNNNNNNNNNNNNNNNNNNNNNNNNNNNNNNNNNNNNNNNNNNNNNNNNNNNN

NNNNNNNNNNNNNNNNNNNNNNNNNNNNNNNNNNNNNNNNNNNNNNNNNNNNNNNNNNNN

NNNNNNNNNNNNNNNNNNNNNNNNNNNNNNNNNNNNNNNNNNNNNNNNNNNNNNNNNNNN

NNNNNNNNNNNNNNNNNNNNNNNNNNNNNNNNNNNNNNNNNNNNNNNNNNNNNNNNNNNN

NNNNNNNNNNNNNNNNNNNNNNNNNNNNNNNNNNNNNNNNNNNNNNNNNNNNNNNNNNNN

NNNNNNNNNNNNNNNNNNNNNNNNNNNNNNNNNNNNNNNNNNNNNNNNNNNNNNNNNNNN

NNNNNNNNN

>Emarcea_castanopsidicola_CBS_117105

------------------------------------AAGCGGCAACAGCTCAAATTTGAA

ATCTGGCCTTCTGGTCCGAGTTGTAATTTGTAGAGGATGATTTTGGCGCGGTGCCTTCCG

AGTTCCCTGGAACGGGACGCCTTAGAGGGTGAGAGCCCCGTACGGTTGGTCGCTAAGCCT

CTGTAAATCTCCTTCGACGAGTCGAGTAGTTTGGGAATGCTGCTCTAAATGGGAGGTAAA

TTTCTTCTAAAGCTAAATACCGGCCAGAGACCGATAGCGCACAAGTAGAGTGATCGAAAG

ATGAAAAGCACTTTGAAAAGAGGGTTAAATAGCACGTGAAATTGTTGAAAGGGAAGCATT

TATTACCAGACCTTTTCGTTGTGGATCATGTGGTGTTTTCACCGCTGCACTTCGCTTCGT

TCAGGCCAGCATCGGTTTTTGTAGGGGGATAAAAGCTTTGGGAACGTAGCTCCCTCGGGA

GTGTTATAGCCCTTTGCATAATACCCTTACGGGGACCGAGGACCGCGCTTCGCAAGGATG

CTGGCATAATGGTAATCAATGACCCGTCTTGAAACACGGACCAAGGAGTCGAACATTTGT

GCGAGTGTTTGGGTGTTAAACCCTCACGCGTAATGAAAGTGAACGTAGGTGAGAGCCCTT

ACGGGTGCATCATCGACCGATCTTGATGTCTTCGGATGGATTTGAGTAAGAGCATAACTG

TTCGGACCCGAAAGATGGTGAACTATGCGTGGATAGGGTGAAGCCAGAGGAAACTCTGGT

GGAGGCTCGCAGCGGTTCTGACGTGCAAATCGATCGTCAAATCTGCGCATGGGGGCGAAA

GACTTATCGAACCAT---------------------------------------------

------------------------------------------------------------

------------------------------------------------------------

------------------------------------------------------------

------------------------------------------------------------

------------------------------------------------------------

------------------------------------------------------------

------------------------------------------------------------

---------

>Emarcea_eucalyptigena_CBS_139908

----------------TGCCCTAGTAACGGCGAGTGAAGCGGCAACAGCTCAAATTTGAA

ATCTGGCCTTCTGGTCCGAGTTGTAATTTGTAGAGGATGATTTTGGCGCGGTGCCTTCCG

AGTTCCCTGGAACGGGACGCCTTAGAGGGTGAGAGCCCCGTACGGTTGGTCGCTAAGCCT

CTGTAAATCTCCTTCGACGAGTCGAGTAGTTTGGGAATGCTGCTCTAAATGGGAGGTAAA

TTTCTTCTAAAGCTAAATACCGGCCAGAGACCGATAGCGCACAAGTAGAGTGATCGAAAG

ATGAAAAGCACTTTGAAAAGAGGGTTAAATAGCACGTGAAATTGTTGAAAGGGAAGCATT

TATTACCAGACCTTTTCGTTGTGGATCATGTGGTGTTTTCACCGCTGCACTTCGCTTCGT

TCAGGCCAGCATCGGTTTTCGTAGGGGGATAAAAGCTTTGGGAACGTAGCTCCCTCGGGA

GTGTTATAGCCCTTTGCATAATACCCTTACGGGGACCGAGGACCGCGCTTCGCAAGGATG

CTGGCATAATGGTAATCAATGACCCGTCTTGAAACACGGACCAAGGAGTCGAACATTTGT

GCGAGTGTTTGGGTGTTAAACCCTCACGCGTAATGAAAGTGAACGTAGGTGAGAGCCCTT

ACGGGTGCATCATCGACCGATCTTGATGTCTTCGGATGGATTTGAGTAAGAGCATAACTG

TTCGGACCCGAAAGATGGTGAACTATGCGTGGATAGGGTGAAGCCAGAGGAAACTCTGGT

GGAGGCTCGCAGCGGTTCTGACGTGCAAATCGATCGTCAAATCTGCGCATGGGGGCGAAA

GACTTATCGAACCAT---------------------------------------------

------------------------------------------------------------

------------------------------------------------------------

------------------------------------------------------------

------------------------------------------------------------

------------------------------------------------------------

------------------------------------------------------------

------------------------------------------------------------

---------

>Kretzschmaria_deusta_CBS_163.93

NNNNNNNNNNNNNNATTGCCCTAGTAACGGCGAGTGAAGCGGCAACAGCTCAAATTTGAA

ATCTGGCCTTCGGGTCCGAGTTGTAATTTGCAGAGGATGCTTTTGGCGCGGTGCCTTCCG

AGTTCCCTGGAATGGGACGCCTTAGAGGGTGAGAGCCCCGTACGGTTGGACACTAAGCCT

CTGTAAAGCTCCTTCGACGAGTCGAGTAGTTTGGGAATGCTGCTCTAAATGGGAGGTAAA

TTTCTTCTAAAGCTAAATATTGGCCAGAGACCGATAGCGCACAAGTAGAGTGATCGAAAG

ATGAAAAGCACTTTGAAAAGAGGGTTAAATAGCACGTGAAATTGTTGAAAGGGAAGCGTT

TGCGACCAGACCTTTTCCTGGCGGATCATCCGGTGTTCTCACCGGTGCACTTCGCCAGGT

CGAGGCCAGCATCGGTTTTCGTAGGGGGATAAAAGCCTGAGGAACGTAGCTCCCTCGGGA

GTGTTATAGCCCCTCGCATAATACCTTTACGGGGACCGAGGACCGCGCCTTGCAAGGATG

CTGGCATAATGGTTGTCAACGACCCGTCTTGAAACACGGACCAAGGAGTCGAACATTTGT

GCGAGTGTTTGGGTGTTAAACCCTCACGCGTAATGAAAGTGAACGGAGGTGAGAGCCCCT

CGGGGTGCATCATCGACCGATCCTGATGTCTTCGGATGGATTTGAGTAAGAGCATAACTG

TTCGGACCCGAAAGATGGTGAACTATGCGTGGATAGGGTGAAGCCAGAGGAAACTCTGGT

GGAGGCTCGCAGCGGTTCTGACGTGCAAATCGATCGTCAAATCTGCGCATGGGGGCGAAA

GACTTATCGAACCATCTAGTAGCTGGTTACCGCCGAAGTTTCCCTCAGGATAGCAGTGTT

GTTCTTCAGTTTTATGAGGTAAAGCGAATGATTAGGGACTCGGGGGCGCTTTTTAGCCTT

CATCCATTCTCAAACTTTAAATATGTAAGAAGCCCTTGTTACTTAATTGAACGTGGGCAT

TCGAATGTACCAACACTAGTGGGCCATTTTTGGTAAGCAGAACTGGCGATGCGGGATGAA

CCGAACGCGGGGTTAAGGTGCCGGAGTGGACGCTCATCAGACACCACAAAAGGTGTTAGC

ACATTTAGACAATAGGACGGTGGCCATGGAAGTCGGAATCCGCTAAGGACTGTGTAACAA

CTCACCTATCGAATGTGCTAGCCCTGAAAATGGATGGCGCTCAAGCGTCCCACCTATACC

TCGCCCTCAGGGTAGGACCGATGCCCTGAGGAGTAGGCGGCCGTGGGGGTCAGTGACGAA

NNNNNNNNN

>Collodiscula_japonica_CBS_124266

AGAAACCAACAGGGATTGCCCTAGTAACGGCGAGTGAAGCGGCAACAGCTCAAATTTGAA

ATCTGGCCCTCGGGTCCGAGTTGTAATTTGTAGAGGATGCTTCTGGCGCGGTGCCTTCCG

AGTTCCCTGGAACGGGACGCCTTAGAGGGTGAGAGCCCCGTACGGTTGGACACCAAGCCT

CTGTGAAGCTCCTTCGACGAGTCGAGTAGTTTGGGAATGCTGCTCTAAATGGGAGGTAAA

TTTCTTCTAAAGCTAAATATTGGCCAGAGACCGATAGCGCACAAGTAGAGTGATCGAAAG

ATGAAAAGCACTTTGAAAAGAGGGTTAAATAGCACGTGAAATTGTTGAAAGGGAAGCGTT

TGCGACCAGACCTCTTCCTGGCGGATCATCCGGTGTTCTCACCGGTGCACTCCGCCAGGT

CTAGGCCAGCATCGGTTTCCGCAGGGGGATAAAAGCTCCAGGAACGTAGCTCCCTCGGGA

GTGTTATAGCCTTCTGCATAATACCCTTGCGGGGACCGAGGACCGCGCTTCGCAAGGATG

CTGGCGTAATGGTCGTCAACGACCCGTCTTGAAACACGGACCAAGGAGTCGAACATTTGT

GCGAGTGTTTGGGTGTCAAACCCTCACGCGTAATGAAAGTGAACGGAGGTGAGAGCCCTT

ACGGGTGCATCATCGACCGATCCTGATGTCTTCGGATGGATTTGAGTAAGAGCATAACTG

TTCGGACCCGAAAGATGGTGAACTATGCGTGGATAGGGTGAAGCCAGAGGAAACTCTGGT

GGAGGCTCGCAGCGGTTCTGACGTGCAAATCGATCGTCAAATCTGCGCATGGGGGCGAAA

GACTTATCGAACNNNNNNNNNNNNNNNNNNNNNNNNNNNNNNNNNNNNNNNNNNNNNNNN

NNNNNNNNNNNNNNNNNNNNNNNNNNNNNNNNNNNNNNNNNNNNNNNNNNNNNNNNNNNN

NNNNNNNNNNNNNNNNNNNNNNNNNNNNNNNNNNNNNNNNNNNNNNNNNNNNNNNNNNNN

NNNNNNNNNNNNNNNNNNNNNNNNNNNNNNNNNNNNNNNNNNNNNNNNNNNNNNNNNNNN

NNNNNNNNNNNNNNNNNNNNNNNNNNNNNNNNNNNNNNNNNNNNNNNNNNNNNNNNNNNN

NNNNNNNNNNNNNNNNNNNNNNNNNNNNNNNNNNNNNNNNNNNNNNNNNNNNNNNNNNNN

NNNNNNNNNNNNNNNNNNNNNNNNNNNNNNNNNNNNNNNNNNNNNNNNNNNNNNNNNNNN

NNNNNNNNNNNNNNNNNNNNNNNNNNNNNNNNNNNNNNNNNNNNNNNNNNNNNNNNNNNN

NNNNNNNNN

>Daldinia_concentrica_CBS_113277

NNNNNNNNNNNNNNNNGCCCTAGTTAACGGCGAGTGAAGCGGCAACAGCTCAAATTTGAA

ATCTGGCCCTAGGGTCCGAGTTGTAATTTGTAGAGGATGCTTTTGGTTAGGTGCCTTCTG

AGTTCCCTGGAACGGGACGCCAGAGAGGGTGAGAGCCCCGTACGGTTGGACACCGAGCCT

CTATATAGCTCCTTCGACGAGTCGAGTAGTTTGGGAATGCTGCTCTAAATGGGAGGTAAA

TTTCTTCTAAAGCTAAATACCGGCCAGAGACCGATAGCGCACAAGTAGAGTGATCGAAAG

ATGAAAAGTACTTTGAAAAGAGGGTTAAATAGCACGTGAAATTGTTGAAAGGGAAGCGTT

TGCGACCAGACTTTTTCCAGGCGGATCATCCGGTGTTCTCACCGGTGCACTTCGCCTGGT

TTAGGCCAGCATCGGTTCTCTTAGGGGGATAAAGGCCTGGGGAACGTAGCTCCTTCGGGA

GTGTTATAGCCCCTTGCGTAATACCCTTCGGGGGACCGAGGAACGCGCATCGCAAGGATG

CTGGCGTAATGGTCGTCAACGACCCGTCTTGAAACACGGACCAAGGAGTCGAACATTTGT

GCGAGTGTTTGGGTGTTAAACCCTCACGCGTAATGAAAGTGAACGGAGGTGAGAGCCCTT

CGGGGTGCATCATCGACCGATCCTGATGTCTTCGGATGGATTTGAGTAAGAGCATAACTG

TTCGGACCCGAAAGATGGTGAACTATGCGTGGATAGGGTGAAGCCAGAGGAAACTCTGGT

GGAGGCTCGCAGCGGTTCTGACGTGCAAATCGATCGTCAAATCTGCGCATGGGGGCGAAA

GACTTATCGAACCATCTAGTAGCTGGTTACCGCCGAAGTTTCCCTCAGGATAGCAGTGTT

G-TCTTCAGTTTTATGAGGTAAAGCGAATGATTAGGGACTCGGGGGCGCTATATTGCCTT

CATCCATTCTCAAACTTTAAATATGTAAGAAGCCCTTGTTACTTAATTGAACGTGGGCAT

TCGAATGTACCAACACTAGTGGGCCATTTTTGGTAAGCAGAACTGGCGATGCGGGATGAA

CCGAACGCGGGGTTAAGGTGCCAGAGTGGACGCTCATCAGACACCACAAAAGGTGTTAGT

ACATCTTGACAGCAGGACGGTGGCCATGGAAGTCGGAATCCGCTAAGGACTGTGTAACAA

CTCACCTGCCGAATGTACTAGCCCTGAAAATGGATGGCGCTCAAGCGTCTCACCCATACC

TCGCCCTTAGGGTAGAAACGATGCCCTAAGGAGTAGGCGGCCGTGGAGGTTAGTGACGAA

GCCTAGGNN

>Entonaema_liquescens_ATCC_46302

NNNNNNNNNNNNNNATTGCCCTAGTAACGGCGAGTGAAGCGGCAACAGCTCAAATTTGAA

ATCTGGCCCTAGGGTCCGAGTTGTAATTTGTAGAGGATGCTTTTGGTGAGGTGCCTTCCG

AGTTCCCTGGAACGGGACGCCAGAGAGGGTGAGAGCCCCGTACGGTTGGACGCCAAACCT

CTATATAGCTCCTTCGACGAGTCGAGTAGTTTGGGAATGCTGCTCTAAATGGGAGGTAAA

TTTCTTCTAAAGCTAAATACCGGCCAGAGACCGATAGCGCACAAGTAGAGTGATCGAAAG

ATGAAAAGCACTTTGAAAAGAGGGTTAAATAGCACGTGAAATTGTTGAAAGGGAAGCGTT

TGCGACCAGACTTTTTCCAGGCGGATCATCCGGTGTTCTCACCGGTGCACTTCGCCTGGT

TTAGGCCAGCATCGGTTTCCTTAGGGGGATAAAGGCTTGGGGAACGTAGCTCCTTCGGGA

GTGTTATAGCCCCTTGCGTAATACCCCTCGGGGGACCGAGGAACGCGCTTCGCAAGGATG

CTGGCGTAATGGTCGTCAACGACCCGTCTTGAAACACGGACCAAGGAGTCGAACATTTGT

GCGAGTGTTTGGGTGTCAAACCCTCACGCGTAATGAAAGTGAACGGAGGTGAGAGCCCTT

ACGGGTGCATCATCGACCGATCCTGATGTCTTCGGATGGATTTGAGTAAGAGCATAACTG

TTCGGACCCGAAAGATGGTGAACTATGCGTGGATAGGGTGAAGCCAGAGGAAACTCTGGT

GGAGGCTCGCAGCGGTTCTGACGTGCAAATCGATCGTCAAATCTGCGCATGGGGGCGAAA

GACTTATCGAACCATCTAGTAGCTGGTTACCGCCGAAGTTTCCCTCAGGATAGCAGTGTT

G-TCTTCAGTTTTATGAGGTAAAGCGAATGATTAGGGACTCGGGGGCGCTATATTGCCTT

CATCCATTCTCAAACTTTAAATATGTAAGAAGCCCTTGTTACTTAATTGAACGTGGGCAT

TCGAATGTACCAACACTAGTGGGCCATTTTTGGTAAGCAGAACTGGCGATGCGGGATGAA

CCGAACGCGGGGTTAAGGTGCCAGAGTGGACGCTCATCAGACACCACAAAAGGTGTTAGT

ACATCTTGACAGCAGGACGGTGGCCATGGAAGTCGGAATCCGCTAAGGACTGTGTAACAA

CTCACCTGCCGAATGTACTAGCCCTGAAAATGGATGGCGCTCAAGCGTCTCACCCATACC

TCGCCCTTAGGGTAGAAACGATGCCCTAAGGAGTAGGCGGCCGTGGAGGTTAGTGACGAA

NNNNNNNNN

>Ruwenzoria_pseudoannulata_MUCL_51394

NNNNNNNNNNNNNNNNNNNNNNNNNNNNNNNNNNNNNNNNNNNNNNNGCTCAAATTTGAA

ATCTGGCCCTAGGGTCCGAGTTGTAATTTGTAGAGGATGCTTTTGGCGAGGTGCCTTCCG

AGTTCCCTGGAACGGGACGCCGGAGAGGGTGAGAGCCCCGTACGGTTGGACGCCGAGCCT

CTATATAGCTCCTTCGACGAGTCGAGTAGTTTGGGAATGCTGCTCTAAATGGGAGGTAAA

TTTCTTCTAAAGCTAAATACCGGCCAGAGACCGATAGCGCACAAGTAGAGTGATCGAAAG

ATGAAAAGCACTTTGAAAAGAGGGTTAAATAGCACGTGAAATTGTTGAAAGGGAAGCGTT

TGCGACCAGACTTTTTCCGGGCGGATCATCCGGGGTTTTCCCCGGTGCACTTCGCCCGGT

TTAGGCCAGCATCGGTTTCCTTAGGGGGATAAAGGCTTGGGGAACGTAGCTCCCTCGGGA

GTGTTATAGCCCCTTGCGTAATACCCTTCGGGGGACCGAGGAACGCGCTTCGCAAGGATG

CTGGCGTAATGGTCGTCAACGACCCGTCTTGAAACACGGACCAAGGAGTCGAACATTTGT

GCGAGTGTTTGGGTGTCAAACCCTCACGCGTAATGAAAGTGAACGGAGGTGAGAGCCCTT

ACGGGTGCATCATCGACCGATCCTGATGTCTTCGGATGGATTTGAGTAAGAGCATAACTG

TTCGGACCCGAAAGATGGTGAACTATGCGTGGATAGGGTGAAGCCAGAGGAAACTCTGGT

GGAGGCTCGCAGCGGTTCTGACGTGCAAATCGATCGTCAAATCTGCGCATGGGGGCGAAA

GACTTATCGAACCATCTAGTAGCTGGTTACCGCCGAAGTTTCCCTCAGGATAGCAGTGTT

G-TCTTCAGTTTTATGAGGTAAAGCGAATGATTAGGGACTCGGGGGCGCTATATTGCCTT

CATCCATTCTCAAACTTTAAATATGTAAGAAGCCCTTGTTACTTAATTGAACGTGGGCAT

TCGAATGTACCAACACTAGTGGGCCATTTTTGGTAAGCAGAACTGGCGATGCGGGATGAA

CCGAACGCGGGGTTAAGGTGCCAGAGTGGACGCTCATCAGACACCACAAAAGGTGTTAGT

ACATCTTGACAGCAGGACGGTGGCCATGGAAGTCGGAATCCGCTAAGGACTGTGTAACAA

CTCACCTGCCGAATGTACTAGCCCTGAAAATGGATGGCGCTCAAGCGTCTCACCCATACC

TCGCCCTTAGGGNNNNNNNNNNNNNNNNNNNNNNNNNNNNNNNNNNNNNNNNNNNNNNNN

NNNNNNNNN

>Hypomontagnella_monticulosa_MUCL_54604

NNNNNNNNNNNNNNNNNNNCCTAGTAACGGCGAGTGAAGCGGCAACAGCTCAAATTTGAA

ATCTGGCCTTCGGGTCCGAATTGTAATTTGTAGAGGATGCTTTGGGTGCGGTACCTTCCG

AGTTCCCTGGAACGGGACGCCGGAGAGGGTGAGAGCCCCGTACGGTTGGATACCAAGCCT

ATGTATAGCTCCTTCGACGAGTCGAGTAGTTTGGGAATGCTGCTCTAAATGGGAGGTAAA

TTTCTTCTAAAGCTAAATACCGGCCAGAGACCGATAGCGCACAAGTAGAGTGATCGAAAG

ATGAAAAGCACTTTGAAAAGAGGGTTAAATAGCACGTGAAATTGTTGAAAGGGAAGCGTT

TGCGACCAGACCTTTTCCAGGCGGATCATCCGGTGTTCTCACCGGTGCACTTCGCCTGGT

TTAGGCCAGCATCGGTTTTCTTAGGGGGATAAAGGTTTAGGGCACGTAGCTCCTTCGGGA

GTGTTATAGCCCTCTACGTAATACCCTTCGGGGGACCGAGGACCGCGCATTGCAAGGATG

CTGGCGTAATGGTCGTCAACGACCCGTCTTGAAACACGGACCAAGGAGTCGAACATTTGT

GCGAGTGTTTGGGTGTTAAACCCTCACGCGTAATGAAAGTGAACGGAGGTGAGAGCCCTT

CGGGGTGCATCATCGACCGATCCTGATGTCTTCGGATGGATTTGAGTAAGAGCATAACTG

TTCGGACCCGAAAGATGGTGAACTATGCGTGGATAGGGTGAAGCCAGAGGAAACTCTGGT

GGAGGCTCGCAGCGGTTCTGACGTGCAAATCGATCGTCAAATCTGCGCATGGGGGCGAAA

GACTTATCGAACCATCTAGTAGCTGGTTACCGCCGAAGTTTCCCTCAGGATAGCAGTGTT

G-TCTTCAGTTTTATGAGGTAAAGCGAATGATTAGGGACTCGGGGGCGCTATATTGCCTT

CATCCATTCTCAAACTTTAAATATGTAAGAAGCCCTTGTTACTTAGTTGAACGTGGGCAT

TCGAATGTACCAACACTAGTGGGCCATTTTTGGTAAGCAGAACTGGCGATGCGGGATGAA

CCGAACGTGGGGTTAAGGTGCCAGAGTGGACGCTCATCAGACACCACAAAAGGTGTTAGT

ACATCTTGACAGCAGGACGGTGGCCATGGAAGTCGGAATCCGCTAAGGACTGTGTAACAA

CTCACCTNCCGAATGTACTAGCCCTGAAAATGGATGGCGCTCAAGCGTCTCACCCATACC

TCACCCTTAGGGTAGAAACGATGCCCTAAGGAGTAGGCGNNNNNNNNNNNNNNNNNNNNN

NNNNNNNNN

>Jackrogersella_multiformis_CBS_119016

NNNNNNNNNNNNNNNNNNNNNNNNNNNNNNNNNNNNNNNNNNNNNNNNNNNNNNNNNNNN

NNNNNNNCCTAGGGTCCGAGTTGTAATTTGCAGAGGATGCTTTTGGTGCGGTGCCTTCCG

AGTTCCCTGGAACGGGACGCCGGAGAGGGTGAGAGCCCCGTACGGTTGGACACCTACCCT

ATACATAGCTCCTTCGACGAGTCGAGTAGTTTGGGAATGCTGCTCTAAATGGGAGGTAAA

TTTCTTCTAAAGCTAAATACCGGCCAGAGACCGATAGCGCACAAGTAGAGTGATCGAAAG

ATGAAAAGCACTTTGAAAAGAGGGTTAAATAGCACGTGAAATTGTTGAAAGGGAAGCGTT

TGCGACCAGACCTTTTCCAGGCGGATCATCCGGCGTTCTCGCCGGTGCACTCCGCCTGGT

CTAGGCCAGCATCGGTTTCCTTAGGGGGATAAAGGCCTGGGGAACGTAGCTCTTCAGGGA

GTGTTATAGCCCCTAGCGTAATACCCTTCAGGGGACCGAGGACCGCGCTTCGCAAGGATG

CTGGCGTAATGGTCGTCAACGACCCGTCTTGAAACACGGACCAAGGAGTCGAACATTTGT

GCGAGTGTTTGGGTGTCAAACCCTCACGCGTAATGAAAGTGAACGGAGGTGAGAGCCCTT

ACGGGTGCATCATCGACCGATCCTGAAGTCTTCGGATGGATTTGAGTAAGAGCATAACTG

TTCGGACCCGAAAGATGGTGAACTATGCGTGGATAGGGTGAAGCCAGAGGAAACTCTGGT

GGAGGCTCGCAGCGGTTCTGACGTGCAAATCGATCGTCAAATCTGCGCATGGGGGCGAAA

GACTTATCGAACCATCTAGTAGCTGGTTACCGCCGAAGTTTCCCTCAGGATAGCAGTGTT

G-TCTTCAGTTTTATGAGGTAAAGCGAATGATTAGGGACTCGGGGGCGCTATTTTGCCTT

CATCCATTCTCAAACTTTAAATATGTAAGAAGCCCTTGTTGCTTAATTGAACGTGGGCAT

TCGAATGTATCAACACTAGTGGGCCATTTTTGGTAAGCAGAACTGGCGATGCGGGATGAA

CCGAACGCGGGGTTAAGGTGCCAGAGTGGACGCTCATCAGACACCACAAAAGGTGTTAGT

ACATCTTGACAGCAGGACGGTGGCCATGGAAGTCGGAATCCGCTAAGGACTGTGTAACAA

CTCACCTGCCGAATGTACTAGCCCTGAAAATGGATGGCGCTCAAGCGTCTCACCCATACC

TCGCCCTTAGGGTAGAAACGATGCCCTAAGGAGTAGGCGGCCGNNNNNNNNNNNNNNNNN

NNNNNNNNN

>Spiririma_gaudefroyi_CBS_147284

AGAAACCAACAGGGATTGCCCTAGTAACGGCGAGTGAAGCGGCAACAGCTCAAATTTGAA

ATCTGGCTCTAGGGCCCGAGTTGTAATTTGTAGAGGATGATTCTGGCGCGGCGCCTTCCG

AGTTCCCTGGAACGGGACGCCTTAGAGGGTGAGAGCCCCGTACGGTTGGACGCTAAGCCT

CTGTGAATCACCTTCGACGAGTCGAGTAGTTTGGGAATGCTGCTCTAAATGGGAGGTAAA

TTTCTTCTAAAGCTAAATACCGGCCAGAGACCGATAGCGCACAAGTAGAGTGATCGAAAG

ATGAAAAGCACTTTGAAAAGAGGGTTAAATAGCACGTGAAATTGTTGAAAGGGAAGCATT

TGCTACCAGACCTTTTCCCTGCGGATCATGTGGTGTTATCACCGCTGCACTTCGCTTGGT

TCAGGCCAGCATCGGTCTTTGTAGGGGGATAAAAGCCTTAGGAACGTAGCTCCCTCGGGA

GTGTTATAGCCTCTTGCATAATACCCTTACGGGGACCGAGGACCGCGCTTCGCAAGGATG

CTGGCATAATGGTAGTCAATGACCCGTCTTGAAACACGGACCAAGGAGTCGAACATTTGT

GCGAGTGTTTGGGTGTCAAACCCTCACGCGTAATGAAAGTGAACGTAGGTGAGAGCCCTC

GCGGGTGCATCATCGACCGATCTTGATGTCTTCGGATGGATTTGAGTAAGAGCATAACTG

TTCGGACCCGAAAGATGGTGAACTATGCGTGGATAGGGTGAAGCCAGAGGAAACTCTGGT

GGAGGCTCGCAGCGGTTCTGACGTGCAAATCGATCGTCAAATCTGCGCATGGGGGCGAAA

GACTTATCGAACCAT---------------------------------------------

------------------------------------------------------------

------------------------------------------------------------

------------------------------------------------------------

------------------------------------------------------------

------------------------------------------------------------

------------------------------------------------------------

------------------------------------------------------------

---------

>Muscodor_thailandica_MFLUCC_17_2669

AG--ACCAACAGGGATTGCCCTAGTAACGGCGAGTGAAGCGGCAACAGCTCAAATTTGAA

ATCTGGCTCTAGGGCCCGAGTTGTAATTTGCAGAGGATGATTTTGGCGCGGTGCCTTCCG

AGTTCCCTGGAACGGGACGCCTTAGAGGGTGAGAGCCCCGTACGGTTGGACACCAAGCCT

CTGTAAATCTCCTTCGACGAGTCGAGTAGTTTGGGAATGCTGCTCTAAATGGGAGGTAAA

TTTCTTCTAAAGCTAAATACCGGCCAGAGACCGATAGCGCACAAGTAGAGTGATCGAAAG

ATGAAAAGCACTTTGAAAAGAGGGTTAAATAGCACGTGAAATTGTTGAAAGGGAAGCATT

TACTACCAGACCTTCTCCCTGCGGATCATGCGGTGTTTTCACCGCTGCACTTCGCTTGGT

TTAGGCTAGCATCGGTTTTCGTAGGGGGATAAAAGCATTAGGAACGTAGCTCCCTCGGGA

GTGTTATAGCCTTTTGCATAATACCCTTATGGGGACCGAGGACCGCGCTTTGCAAGGATG

CTGGCATAATGGTAGTCAATGACCCGTCTTGAAACACGGACCAAGGAGTCGAACATTTGT

GCGAGTGTTTGGGTGTTAAACCCTCACGCGTAATGAAAGTGAACGTAGGTGAGAGCCCTT

ACGGGTGCATCATCGACCGATCTTGATGTCTTCGGATGGATTTGAGTAAGAGCATAACTG

TTCGGACCCGAAAGATGGTGAACTATGCGTGGATAGGGTGAAGCCAGAGGAAACTCTGGT

GGAGGCTCGCAGCGGTTCTGACGTGCAAATCGATCGTCAAATCTGCGCATGGGGGCGAAA

GACTTATCGAACCAT---------------------------------------------

------------------------------------------------------------

------------------------------------------------------------

------------------------------------------------------------

------------------------------------------------------------

------------------------------------------------------------

------------------------------------------------------------

------------------------------------------------------------

---------

>Muscodor_ziziphi_MFLUCC_17_2662

AG--ACCAACAGGGATTGCCCTAGTAACGGCGAGTGAAGCGGCAACAGCTCAAATTTGAA

ATCTGGCTCTAGGGCCCGAGTTGTAATTTGCAGAGGATGATTTTGGCGCGGTGCCTTCCG

AGTTCCCTGGAACGGGACGCCTTAGAGGGTGAGAGCCCCGTACGGTTGGACACCAAGCCT

CTGTAAATCTCCTTCGACGAGTCGAGTAGTTTGGGAATGCTGCTCTAAATGGGAGGTAAA

TTTCTTCTAAAGCTAAATACCGGCCAGAGACCGATAGCGCACAAGTAGAGTGATCGAAAG

ATGAAAAGCACTTTGAAAAGAGGGTTAAATAGCACGTGAAATTGTTGAAAGGGAAGCATT

TACTACCAGACCTTCTCCCTGCGGATCATGCGGTGTTTTCACCGCTGCACTTCGCTTGGT

TTAGGCTAGCATCGGTTTTCGTAGGGGGATAAAAGCATTAGGAACGTAGCTCCCTCGGGA

GTGTTATAGCCTTTTGCATAATACCCTTATGGGGACCGAGGACCGCGCTTTGCAAGGATG

CTGGCATAATGGTAGTCAATGACCCGTCTTGAAACACGGACCAAGGAGTCGAACATTTGT

GCGAGTGTTTGGGTGTTAAACCCTCACGCGTAATGAAAGTGAACGTAGGTGAGAGCCCTT

ACGGGTGCATCATCGACCGATCTTGATGTCTTCGGATGGATTTGAGTAAGAGCATAACTG

TTCGGACCCGAAAGATGGTGAACTATGCGTGGATAGGGTGAAGCCAGAGGAAACTCTGGT

GGAGGCTCGCAGCGGTTCTGACGTGCAAATCGATCGTCAAATCTGCGCATGGGGGCGAAA

GACTTATCGAACCAT---------------------------------------------

------------------------------------------------------------

------------------------------------------------------------

------------------------------------------------------------

------------------------------------------------------------

------------------------------------------------------------

------------------------------------------------------------

------------------------------------------------------------

---------

>Muscodor_equiseti_JCM_18233

------------------------------------------------------------

------------------------------------------------------------

------------------------------------------------------------

------------------------------------------------------------

------------------------------------------------------------

------------------------------------------------------------

------------------------------------------------------------

------------------------------------------------------------

------------------------------------------------------------

------------------------------------------------------------

------------------------------------------------------------

------------------------------------------------------------

------------------------------------------------------------

------------------------------------------------------------

------------------------------------------------------------

------------------------------------------------------------

------------------------------------------------------------

------------------------------------------------------------

------------------------------------------------------------

------------------------------------------------------------

------------------------------------------------------------

------------------------------------------------------------

---------

>Muscodor_vitigena_MONT_P_15

------------------------------------------------------------

------------------------------------------------------------

------------------------------------------------------------

------------------------------------------------------------

------------------------------------------------------------

------------------------------------------------------------

------------------------------------------------------------

------------------------------------------------------------

------------------------------------------------------------

------------------------------------------------------------

------------------------------------------------------------

------------------------------------------------------------

------------------------------------------------------------

------------------------------------------------------------

------------------------------------------------------------

------------------------------------------------------------

------------------------------------------------------------

------------------------------------------------------------

------------------------------------------------------------

------------------------------------------------------------

------------------------------------------------------------

------------------------------------------------------------

---------

>Muscodor_suturae_MSUB_2380

------------------------------------------------------------

------------------------------------------------------------

------------------------------------------------------------

------------------------------------------------------------

------------------------------------------------------------

------------------------------------------------------------

------------------------------------------------------------

------------------------------------------------------------

------------------------------------------------------------

------------------------------------------------------------

------------------------------------------------------------

------------------------------------------------------------

------------------------------------------------------------

------------------------------------------------------------

------------------------------------------------------------

------------------------------------------------------------

------------------------------------------------------------

------------------------------------------------------------

------------------------------------------------------------

------------------------------------------------------------

------------------------------------------------------------

------------------------------------------------------------

---------

>Muscodor_coffeana_COAD_1842

------------------------------------------------------------

------------------------------------------------------------

------------------------------------------------------------

------------------------------------------------------------

------------------------------------------------------------

------------------------------------------------------------

------------------------------------------------------------

------------------------------------------------------------

------------------------------------------------------------

------------------------------------------------------------

------------------------------------------------------------

------------------------------------------------------------

------------------------------------------------------------

------------------------------------------------------------

------------------------------------------------------------

------------------------------------------------------------

------------------------------------------------------------

------------------------------------------------------------

------------------------------------------------------------

------------------------------------------------------------

------------------------------------------------------------

------------------------------------------------------------

---------

>Muscodor_yucatanensis_MEXU_25511

------------------------------------------------------------

------------------------------------------------------------

------------------------------------------------------------

------------------------------------------------------------

------------------------------------------------------------

------------------------------------------------------------

------------------------------------------------------------

------------------------------------------------------------

------------------------------------------------------------

------------------------------------------------------------

------------------------------------------------------------

------------------------------------------------------------

------------------------------------------------------------

------------------------------------------------------------

------------------------------------------------------------

------------------------------------------------------------

------------------------------------------------------------

------------------------------------------------------------

------------------------------------------------------------

------------------------------------------------------------

------------------------------------------------------------

------------------------------------------------------------

---------

>Muscodor_sp._SMH_1255

AGAACCCAACAGGGATTGCCTTAGTAACGGCGAGTGAAGCGGCAACAGCTCAAATTTGAA

ATCTGGCTCTAGGGCCCGAGTTGTAATTTGCAGAGGATGATTTTGGCGCGGTGCCTTCCG

AGTTCCCTGGAACTGGACGCCTTAGAGGGTGAGAGCCCCGTACGGTTGGACACCAAGCCT

CTGTAAATCTCCTTCGACGAGTCGAGTAGTTTGGGAATGCTGCTCTAAATGGGAGGTAAA

TTTCTTCTAAAGCTAAATACCGGCCAGAGACCGATAGCGCACAAGTAGAGTGATCGAAAG

ATGAAAAGCACTTTGAAAAGAGGGTTAAATAGCACGTGAAATTGTTGAAAGGGAAGCATT

TACTACCAGACCTTCTCCCTGCGGATCATGCGGTGTTCTCACCGCTGCACTTCGCTTGGT

TTAGGCCAGCATCGGTTTTCGTAGGGGGATAAAAGCTCTAGGAACGTAGCTCCCTCGGGA

GTGTTATAGCCTTCTGCATAATACCCCTCCGGGGACCGAGGACCGCGCTTTGCAAGGATG

CTGGCATAATAATAGTCAATGACCCGTCTTGAAACACGGACCAAGGAGTCGAACATTTGT

GCGAGTGTTTGGGTGTTAAACCCTCACGCGTAATGAAAGTGAACGTA-GTGAGAGCTCTT

GCGGGCGCATCATCGACCGATCTTGATGTCTTCGGATGGATTTGAGTAAGAGCATAACTG

TTCGGACCCGAAAGATGGTGAACTATGCGTGGATAGGGTGAAGCCAGAGGAAACTCTGGT

GGAGGCTCGCAGCGGTTCTGACGTGCAAATCGATCGTCAAATCTGCGCATGGGGGCGAAA

GACTTATCGAACCATCTAGTAGCTGGTTACCGCCGAAGTTTCCCTCAGGATAGCAGTGTT

GATCTTCAGTTTTATGAGGTAAAGCGAATGATTAGGGACTCGGGGGCTAATTTTTGCCTT

CATCCATTCTCAAACTTTAAATATGTAAGAAGCCTTTGTTACTTAATTGAACGTGGGCAT

TCGAATGTACCAACACTAGTGGGCCATTTTTGGTAAGCAGAACTGGCG------------

------------------------------------------------------------

------------------------------------------------------------

------------------------------------------------------------

------------------------------------------------------------

---------

>Muscodor_brasiliensis_LGMF_1256

------------------------------------------------------------

------------------------------------------------------------

------------------------------------------------------------

------------------------------------------------------------

------------------------------------------------------------

------------------------------------------------------------

------------------------------------------------------------

------------------------------------------------------------

------------------------------------------------------------

------------------------------------------------------------

------------------------------------------------------------

------------------------------------------------------------

------------------------------------------------------------

------------------------------------------------------------

------------------------------------------------------------

------------------------------------------------------------

------------------------------------------------------------

------------------------------------------------------------

------------------------------------------------------------

------------------------------------------------------------

------------------------------------------------------------

------------------------------------------------------------

---------

>Muscodor_alba_9_6

-----------------GCCCTAGTAACGGCGAGTGAAGCGGCAACAGCTCAAATTTGAA

ATCTGGCTCTAGGGCCCGAGTTGTAATTTGCAGAGGATGATTTTGGCGCGGTGCCTTCCG

AGTTCCCTGGAACGGGACGCCTTAGAGGGTGAGAGCCCCGTACGGTTGGACACCAAGCCT

CTGTAAATCTCCTTCGACGAGTCGAGTAGTTTGGGAATGCTGCTCTAAATGGGAGGTAAA

TTTCTTCTAAAGCTAAATACCGGCCAGAGACCGATAGCGCACAAGTAGAGTGATCGAAAG

ATGAAAAGCACTTTGAAAAGAGGGTTAAATAGCACGTGAAATTGTTGAAAGGGAAGCATT

TACTACCAGACCTTCTCCCTGCGGATCATGCGGTGTTCTCACCGCTGCACTTCGCTTGGT

TTAGGCCAGCATCGGTTTTCGTAGAGGGATAAAAGCTTTAGGAACGTAGCTCCCTCGGGA

GTGTTATAGCCTTTTGCATAATACCTTTACGGGGACCGAGGACCGCGCTTTGCAAGGATG

CTGGCATAATGGTAGTCAATGACCCGTCTTGAAACACGGACCAAGGAGTCGAACATTTAT

GCGAGTGTTTGGGTGTTAAACCCTCACGCGTAATGAAAGTGAACGTAGGTGAGAGCCCTT

ACGGGTGCATCATCGACCGATCTTGATGTCTTCGGATGGATTTGAGTAAGAGCATAACTG

TTCGGACCCGAAAGATGGTGAACTATGCGTGGATAGGGTGAAGCCAGAGGAAACTCTGGT

GGAGGCTCGCAGCGGTTCTGACGTGCAAATCGATCGTCAAATCTGCGCATGGGGGCGAAA

GACTTATCGA--------------------------------------------------

------------------------------------------------------------

------------------------------------------------------------

------------------------------------------------------------

------------------------------------------------------------

------------------------------------------------------------

------------------------------------------------------------

------------------------------------------------------------

---------

>Muscodor_alba_MONT_620

------------------------------------------------------------

------------------------------------------------------------

------------------------------------------------------------

------------------------------------------------------------

------------------------------------------------------------

------------------------------------------------------------

------------------------------------------------------------

------------------------------------------------------------

------------------------------------------------------------

------------------------------------------------------------

------------------------------------------------------------

------------------------------------------------------------

------------------------------------------------------------

------------------------------------------------------------

------------------------------------------------------------

------------------------------------------------------------

------------------------------------------------------------

------------------------------------------------------------

------------------------------------------------------------

------------------------------------------------------------

------------------------------------------------------------

------------------------------------------------------------

---------

>Muscodor_crispans_MONT_2347

------------------------------------------------------------

------------------------------------------------------------

------------------------------------------------------------

------------------------------------------------------------

------------------------------------------------------------

------------------------------------------------------------

------------------------------------------------------------

------------------------------------------------------------

------------------------------------------------------------

------------------------------------------------------------

------------------------------------------------------------

------------------------------------------------------------

------------------------------------------------------------

------------------------------------------------------------

------------------------------------------------------------

------------------------------------------------------------

------------------------------------------------------------

------------------------------------------------------------

------------------------------------------------------------

------------------------------------------------------------

------------------------------------------------------------

------------------------------------------------------------

---------

>Muscodor_musae_JCM_18230

------------------------------------------------------------

------------------------------------------------------------

------------------------------------------------------------

------------------------------------------------------------

------------------------------------------------------------

------------------------------------------------------------

------------------------------------------------------------

------------------------------------------------------------

------------------------------------------------------------

------------------------------------------------------------

------------------------------------------------------------

------------------------------------------------------------

------------------------------------------------------------

------------------------------------------------------------

------------------------------------------------------------

------------------------------------------------------------

------------------------------------------------------------

------------------------------------------------------------

------------------------------------------------------------

------------------------------------------------------------

------------------------------------------------------------

------------------------------------------------------------

---------

>Muscodor_oryzae_JCM_18231

------------------------------------------------------------

------------------------------------------------------------

------------------------------------------------------------

------------------------------------------------------------

------------------------------------------------------------

------------------------------------------------------------

------------------------------------------------------------

------------------------------------------------------------

------------------------------------------------------------

------------------------------------------------------------

------------------------------------------------------------

------------------------------------------------------------

------------------------------------------------------------

------------------------------------------------------------

------------------------------------------------------------

------------------------------------------------------------

------------------------------------------------------------

------------------------------------------------------------

------------------------------------------------------------

------------------------------------------------------------

------------------------------------------------------------

------------------------------------------------------------

---------

>Muscodor_rosea_MONT_2098

------------------------------------------------------------

------------------------------------------------------------

------------------------------------------------------------

------------------------------------------------------------

------------------------------------------------------------

------------------------------------------------------------

------------------------------------------------------------

------------------------------------------------------------

------------------------------------------------------------

------------------------------------------------------------

------------------------------------------------------------

------------------------------------------------------------

------------------------------------------------------------

------------------------------------------------------------

------------------------------------------------------------

------------------------------------------------------------

------------------------------------------------------------

------------------------------------------------------------

------------------------------------------------------------

------------------------------------------------------------

------------------------------------------------------------

------------------------------------------------------------

---------

>Muscodor_kashayum_NFCCI_2947

------------------------------------------------------------

------------------------------------------------------------

------------------------------------------------------------

------------------------------------------------------------

------------------------------------------------------------

------------------------------------------------------------

------------------------------------------------------------

------------------------------------------------------------

------------------------------------------------------------

------------------------------------------------------------

------------------------------------------------------------

------------------------------------------------------------

------------------------------------------------------------

------------------------------------------------------------

------------------------------------------------------------

------------------------------------------------------------

------------------------------------------------------------

------------------------------------------------------------

------------------------------------------------------------

------------------------------------------------------------

------------------------------------------------------------

------------------------------------------------------------

---------

>Muscodor_tigerensis_NFCCI_3172

------------------------------------------------------------

------------------------------------------------------------

------------------------------------------------------------

------------------------------------------------------------

------------------------------------------------------------

------------------------------------------------------------

------------------------------------------------------------

------------------------------------------------------------

------------------------------------------------------------

------------------------------------------------------------

------------------------------------------------------------

------------------------------------------------------------

------------------------------------------------------------

------------------------------------------------------------

------------------------------------------------------------

------------------------------------------------------------

------------------------------------------------------------

------------------------------------------------------------

------------------------------------------------------------

------------------------------------------------------------

------------------------------------------------------------

------------------------------------------------------------

---------

>Muscodor_cinnanomi_BCC_38842

------------------------------------------------------------

------------------------------------------------------------

------------------------------------------------------------

------------------------------------------------------------

------------------------------------------------------------

------------------------------------------------------------

------------------------------------------------------------

------------------------------------------------------------

------------------------------------------------------------

------------------------------------------------------------

------------------------------------------------------------

------------------------------------------------------------

------------------------------------------------------------

------------------------------------------------------------

------------------------------------------------------------

------------------------------------------------------------

------------------------------------------------------------

------------------------------------------------------------

------------------------------------------------------------

------------------------------------------------------------

------------------------------------------------------------

------------------------------------------------------------

---------

>Muscodor_camphorae_NFCCI_3236

------------------------------------------------------------

------------------------------------------------------------

------------------------------------------------------------

------------------------------------------------------------

------------------------------------------------------------

------------------------------------------------------------

------------------------------------------------------------

------------------------------------------------------------

------------------------------------------------------------

------------------------------------------------------------

------------------------------------------------------------

------------------------------------------------------------

------------------------------------------------------------

------------------------------------------------------------

------------------------------------------------------------

------------------------------------------------------------

------------------------------------------------------------

------------------------------------------------------------

------------------------------------------------------------

------------------------------------------------------------

------------------------------------------------------------

------------------------------------------------------------

---------

>Muscodor_ghoomensis_NFCCI_3234

------------------------------------------------------------

------------------------------------------------------------

------------------------------------------------------------

------------------------------------------------------------

------------------------------------------------------------

------------------------------------------------------------

------------------------------------------------------------

------------------------------------------------------------

------------------------------------------------------------

------------------------------------------------------------

------------------------------------------------------------

------------------------------------------------------------

------------------------------------------------------------

------------------------------------------------------------

------------------------------------------------------------

------------------------------------------------------------

------------------------------------------------------------

------------------------------------------------------------

------------------------------------------------------------

------------------------------------------------------------

------------------------------------------------------------

------------------------------------------------------------

---------

>Muscodor_indica_NFCCI_3235

------------------------------------------------------------

------------------------------------------------------------

------------------------------------------------------------

------------------------------------------------------------

------------------------------------------------------------

------------------------------------------------------------

------------------------------------------------------------

------------------------------------------------------------

------------------------------------------------------------

------------------------------------------------------------

------------------------------------------------------------

------------------------------------------------------------

------------------------------------------------------------

------------------------------------------------------------

------------------------------------------------------------

------------------------------------------------------------

------------------------------------------------------------

------------------------------------------------------------

------------------------------------------------------------

------------------------------------------------------------

------------------------------------------------------------

------------------------------------------------------------

---------

>Muscodor_suthepensis_JCM_18232

------------------------------------------------------------

------------------------------------------------------------

------------------------------------------------------------

------------------------------------------------------------

------------------------------------------------------------

------------------------------------------------------------

------------------------------------------------------------

------------------------------------------------------------

------------------------------------------------------------

------------------------------------------------------------

------------------------------------------------------------

------------------------------------------------------------

------------------------------------------------------------

------------------------------------------------------------

------------------------------------------------------------

------------------------------------------------------------

------------------------------------------------------------

------------------------------------------------------------

------------------------------------------------------------

------------------------------------------------------------

------------------------------------------------------------

------------------------------------------------------------

---------

>Muscodor_darjeelingensis_NFCCI_3095

------------------------------------------------------------

------------------------------------------------------------

------------------------------------------------------------

------------------------------------------------------------

------------------------------------------------------------

------------------------------------------------------------

------------------------------------------------------------

------------------------------------------------------------

------------------------------------------------------------

------------------------------------------------------------

------------------------------------------------------------

------------------------------------------------------------

------------------------------------------------------------

------------------------------------------------------------

------------------------------------------------------------

------------------------------------------------------------

------------------------------------------------------------

------------------------------------------------------------

------------------------------------------------------------

------------------------------------------------------------

------------------------------------------------------------

------------------------------------------------------------

---------

>Muscodor_strobelii_NFCCI_2907

------------------------------------------------------------

------------------------------------------------------------

------------------------------------------------------------

------------------------------------------------------------

------------------------------------------------------------

------------------------------------------------------------

------------------------------------------------------------

------------------------------------------------------------

------------------------------------------------------------

------------------------------------------------------------

------------------------------------------------------------

------------------------------------------------------------

------------------------------------------------------------

------------------------------------------------------------

------------------------------------------------------------

------------------------------------------------------------

------------------------------------------------------------

------------------------------------------------------------

------------------------------------------------------------

------------------------------------------------------------

------------------------------------------------------------

------------------------------------------------------------

---------

>Muscodor_yunnanensis_CGMCC_3.18908

------------GGATTGCCCTAGT-ACGGCGAGTGAAGCGGCAACAGCTCAAATTTGAA

ATCTGGCTTTCGGGCCCGAGTTGTAATTTGCAGAGGATGATTTTGGCGCGGTGCCTTCCG

AGTTCCCTGGAACGGGACGCCTTAGAGGGTGAGAGCCCCGTACGGTTGGACACCAAGCCT

CTGTAAATCTCCTTCGACGAGTCGAGTAGTTTGGGAATGCTGCTCTAAATGGGAGGTAAA

TTTCTTCTAAAGCTAAATACCGGCCAGAGACCGATAGCGCACAAGTAGAGTGATCGAAAG

ATGAAAAGCACTTTGAAAAGAGGGTTAAATAGCACGTGAAATTGTTGAAAGGGAAGCATT

TACTACCAGACCTTTTCCTAGCGGATCATGTGGTGTTCTCACCGCTGCACTTCGCTAGGT

TGAGGCCAGCATCGGTTTTCGTAGGGGGACAAAAGCCTTAGGAACGTAGCTCCCTCGGGA

GTGTTATAGCCTTTGGCATAATACCCTTACGGGGACCGAGGACCGCGCTTTGCAAGGATG

CTGGCATAATGGTAGTCAATGACCCGTCTTGAAACACGGACCAAGGAGTCGAACATTTGT

GCGAGTGTTTGGGTGTTAAACCCTCACGCGTAATGAAAGTGAACGTAGGTGAGAGCCCTT

ACGGGTGCATCATCGACCGATCTTGATGTCTTCGGATGGATTTGAGTAAGAGCATAACTG

TTCGGACCCGAAAGATGGTGAACTATGCGTGGATAGGGTGAAGCCAGAGGAAACTCTGGT

GGAGGCTCGCAGCGGTTCTGACGTGCAAATCGATCGTCAAATCTGCGCATGGGGGCGAAA

GACTTATCGAACCAT---------------------------------------------

------------------------------------------------------------

------------------------------------------------------------

------------------------------------------------------------

------------------------------------------------------------

------------------------------------------------------------

------------------------------------------------------------

------------------------------------------------------------

---------

>Muscodor_fengyangensis_CGMCC_2862

-----------------GCCCTAGTAACGGCGAGTGAAGCGGCAACAGCTCAAATTTGAA

ATCTGGCTCTCGGGCCCGAGTTGTAATTTGTAGAGGATGATTTTGGCGCGGTGCCTTCCG

AGTTCCCTGGAACGGGACGCCTTAGAGGGTGAGAGCCCCGTACGGTTGGACACCAAGCCT

CTGTAAATCTCCTTCGACGAGTCGAGTAGTTTGGGAATGCTGCTCTAAATGGGAGGTAAA

TTTCTTCTAAAGCTAAATACCGGCCAGAGACCGATAGCGCACAAGTAGAGTGATCGAAAG

ATGAAAAGCACTTTGAAAAGAGGGTTAAATAGCACGTGAAATTGTTGAAAGGGAAGCATT

TACTACCAGACCTCTGCCCTGCGGATCATGTGGTGTTCTCACCGCTGCACTTCGCTTGGT

TTAGGCCAGCATCGGTTTTTGTAGGGGGATAAAAGCCTTAGGAACGTAGCTCCCTCGGGA

GTGTTATAGCCTTTTGCATAATACCCTTACGGGGACCGAGGACCGCGCTTCGCAAGGATG

CTGGCATAATGGTAGTCAATGACCCGTCTTGAAACACGGACCAAGGAGTCGAACATTTGT

GCGAGTGTTTGGGTGTTAAACCCTCACGCGTAATGAAAGTGAACGTAGGTGAGAGCCCTT

ACGGGTGCATCATCGACCGATCTTGATGTCTTCGGATGGATTTGAGTAAGAGCATAACTG

TTCGGACCCGAAAGATGGTGAACTATGCGTGGATAGGGTGAAGCCAGAGGAAACTCTGGT

GGAGGCTCGCAGCGGTTCTGACGTGCAAATCGATCGTCAAATCTGCGCATGGGGGCGAAA

GACTTATCGA--------------------------------------------------

------------------------------------------------------------

------------------------------------------------------------

------------------------------------------------------------

------------------------------------------------------------

------------------------------------------------------------

------------------------------------------------------------

------------------------------------------------------------

---------

>Camillea_obularia_ATCC_28093

NNNNNNNNNNNNNNATTGCCCTAGTAACGGCGAGTGAAGCGGCAACAGCTCAAATTTGAA

ATCTGGCCTTCGGGTCCGAATTGTAATTTGCAGAGGATGCTTTTGGCGCGGTGCCTTCCG

AGTTCCCTGGAACGGGACGCCTTAGAGGGTGAGAGCCCCGTACGGTTGGACACCAAGCCT

CTGTAAAGCTCCTTCGACGAGTCGAGTAGTTTGGGAATGCTGCTCTAAATGGGAGGTAAA

TTTCTTCTAAAGCTAAATACCGGCCAGAGACCGATAGCGCACAAGTAGAGTGATCGAAAG

ATGAAAAGCACTTTGAAAAGAGGGTTAAATAGCACGTGAAATTGTTGAAAGGGAAGCGTT

TACGGCCAGACCTTTTCCTGGCGGATCATCCGGTGTTCTCACCGGTGCACTTCGCCTGGT

TTAGGCCAGCATCGGCTTCTGTAGGGGGATAAAAGCAGTGGGAAAGTAGCTCCTTCGGGA

GTGTTATAGCCCTAAGCATAATACCTTTACGGGGGCCGAGGACCGCGCTTTGCAAGGATG

CTGGCGTAATGGTCGTCAACGACCCGTCTTGAAACACGGACCAAGGAGTCGAACATTTGT

GCGAGTGTTTGGGTGTTAAACCCTCACGCGTAATGAAAGTGAACGGAGGTGAGAGCCCTT

TAGGGTGCATCATCGACCGATCCTGATGTCTTCGGATGGATTTGAGTAAGAGCATAACTG

TTCGGACCCGAAAGATGGTGAACTATGCGTGGATAGGGTGAAGCCAGAGGAAACTCTGGT

GGAGGCTCGCAGCGGTTCTGACGTGCAAATCGATCGTCAAATCTGCGCATGGGGGCGAAA

GACTTATCGAACCATCTAGTAGCTGGTTACCGCCGAAGTTTCCCTCAGGATAGCAGTGTT

G-TCTTCAGTTTTATGAGGTAAAGCGAATGATTAGGGACTCGGGGGCGCTTATTAGCCTT

CATCCATTCTCAAACTTTAAATATGTAAGAAGCTCTTGTTGCTTAATTGAACGTGAGCAT

TCGAATGTACCAACACTAGTGGGCCATTTTTGGTAAGCAGAACTGGCGATGCGGGATGAA

CCGAACGCGGGGTTAAGGTGCCGGAGTGGACGCTCATCAGACACCACAAAAGGTGTTAGT

ACATCTAGACAGCAGGACGGTGGCCATGGAAGTCGGAATCCGCTAAGGACTGTGTAACAA

CTCACCTGCCGAATGTACTAGCCCTGAAAATGGATGGCGCTCAAGCGTCCCACCCATACC

TCGCCCTCAGGGTAGAAACGATGCCCTGAGGAGTAGGCGGCCGTGGAGGTTAGTGACGAA

GCCTAGGNN

>Obolarina_dryophila_MUCL_49882

AGAAACCAACAGGGATTGCCCTAGTAACGGCGAGTGAAGCGGCAACAGCTCAAATTTGAA

ATCTGGCTTTCGGGTCCGAGTTGTAATTTGCAGAGGATGCTTTTGGCGAGGTGCCTTCCG

AGTTCCCTGGAACGGGACGCCTTAGAGGGTGAGAGCCCCGTACGGTTGGACACCGAGCCT

CTGTAAAGCTCCTTCGACGAGTCGAGTAGTTTGGGAATGCTGCTCTAAATGGGAGGTAAA

TTTCTTCTAAAGCTAAATACCGGCCAGAGACCGATAGCGCACAAGTAGAGTGATCGAAAG

ATGAAAAGCACTTTGAAAAGAGGGTTAAATAGCACGTGAAATTGTTGAAAGGGAAGCGTT

TACGGCCAGACCTTCTCCTGGCGGATCATCTGGTGTTCTCACCAGTGCACTTCGCCAGGT

CTAGGCCAGCATCGGCTTCCGTAGGGGGATAAAAGCAGTGGGAAAGTAGCTCCCTAGGGA

GTGTTATAGCCCTAAGCATAATACCCTTACGGGGGCCGAGGACCGCGCTCTGCAAGGANN

NNNNNNNNNNNNNNNNNNNNNNNNNNNNNNNNNNNNNNNNNNNNNNNNNNNNNNNNNNNN

NNNNNNNNNNNNNNNNNNNNNNNNNNNNNNNNNNNNNNNNNNNNNNNNNNNNNNNNNNNN

NNNNNNNNNNNNNNNNNNNNNNNNNNNNNNNNNNNNNNNNNNNNNNNNNNNNNNNNNNNN

NNNNNNNNNNNNNNNNNNNNNNNNNNNNNNNNNNNNNNNNNNNNNNNNNNNNNNNNNNNN

NNNNNNNNNNNNNNNNNNNNNNNNNNNNNNNNNNNNNNNNNNNNNNNNNNNNNNNNNNNN

NNNNNNNNNNNNNNNNNNNNNNNNNNNNNNNNNNNNNNNNNNNNNNNNNNNNNNNNNNNN

NNNNNNNNNNNNNNNNNNNNNNNNNNNNNNNNNNNNNNNNNNNNNNNNNNNNNNNNNNNN

NNNNNNNNNNNNNNNNNNNNNNNNNNNNNNNNNNNNNNNNNNNNNNNNNNNNNNNNNNNN

NNNNNNNNNNNNNNNNNNNNNNNNNNNNNNNNNNNNNNNNNNNNNNNNNNNNNNNNNNNN

NNNNNNNNNNNNNNNNNNNNNNNNNNNNNNNNNNNNNNNNNNNNNNNNNNNNNNNNNNNN

NNNNNNNNNNNNNNNNNNNNNNNNNNNNNNNNNNNNNNNNNNNNNNNNNNNNNNNNNNNN

NNNNNNNNNNNNNNNNNNNNNNNNNNNNNNNNNNNNNNNNNNNNNNNNNNNNNNNNNNNN

NNNNNNNNNNNNNNNNNNNNNNNNNNNNNNNNNNNNNNNNNNNNNNNNNNNNNNNNNNNN

NNNNNNNNN

>Biscogniauxia_marginata_MFLUCC_12_0740

NNNNNNNNNNNNNNNNNNNNNNNNNNNNNNNNNNNNNAGCGGCAACAGCTCAAATTTGAA

ATCTGGCCCTAGGGTCCGAGTTGTAATTTGTAGAGGATGCTTTTGGCGCGGTGCCTTCCG

AGTTCCCTGGAACGGGACGCCTTAGAGGGTGAGAGCCCCGTACGGTTGGACACTAAGCCT

CTGTAAAGCTCTTTCAACGAGTCGAGTAGTTTGGGAATGCTGCTCTAAATGGGAGGTAAA

TTTCTTCTAAAGCTAAATACCGGCCAGAGACCGATAGCGCACAAGTAGAGTGATCGAAAG

ATGAAAAGTACTTTGAAAAGAGGGTTAAATAGCACGTGAAATTGTTGAAAGGGAAGCGTT

TACGGCCAGATCTTTTCCTGGCGGATCATCCTGTGTTCTCACCGGTGCACTCCGCTAGGT

TTAGGCCAGCATCGGCTTCTGTAGGGGGATAAAAGCCCTGGGAAAGTAGCTCCCTCGGGA

GTGTTATAGCCCTAAGCATAATACCCTTACGGGGGCCGAGGACCGCGCTCTGCAAGGATG

CTGGCGTAATGGTCGTTAACGACCCGTCTTGAAACACGGACCAAGGAGTCGAACATTTGT

GCGAGTGTTTGGGTGTTAAACCCTCACGCGTANNNNNNNNNNNNNNNNNNNNNNNNNNNN

NNNNNNNNNNNNNNNNNNNNNNNNNNNNNNNNNNNNNNNNNNNNNNNNNNNNNNNNNNNN

NNNNNNNNNNNNNNNNNNNNNNNNNNNNNNNNNNNNNNNNNNNNNNNNNNNNNNNNNNNN

NNNNNNNNNNNNNNNNNNNNNNNNNNNNNNNNNNNNNNNNNNNNNNNNNNNNNNNNNNNN

NNNNNNNNNNNNNNNNNNNNNNNNNNNNNNNNNNNNNNNNNNNNNNNNNNNNNNNNNNNN

NNNNNNNNNNNNNNNNNNNNNNNNNNNNNNNNNNNNNNNNNNNNNNNNNNNNNNNNNNNN

NNNNNNNNNNNNNNNNNNNNNNNNNNNNNNNNNNNNNNNNNNNNNNNNNNNNNNNNNNNN

NNNNNNNNNNNNNNNNNNNNNNNNNNNNNNNNNNNNNNNNNNNNNNNNNNNNNNNNNNNN

NNNNNNNNNNNNNNNNNNNNNNNNNNNNNNNNNNNNNNNNNNNNNNNNNNNNNNNNNNNN

NNNNNNNNNNNNNNNNNNNNNNNNNNNNNNNNNNNNNNNNNNNNNNNNNNNNNNNNNNNN

NNNNNNNNNNNNNNNNNNNNNNNNNNNNNNNNNNNNNNNNNNNNNNNNNNNNNNNNNNNN

NNNNNNNNNNNNNNNNNNNNNNNNNNNNNNNNNNNNNNNNNNNNNNNNNNNNNNNNNNNN

NNNNNNNNN

>Graphostroma_platystoma_CBS_270.87

AGAAACCAACAGGGATTGCCCTAGTAACGGCGAGTGAAGCGGCAACAGCTCAAATTTGAA

ATCTGGCTCTAGGGTCCGAATTGTAATTCGTAGAGGATGCTTTTGGCGCGGTCACTTCCG

AGTTCCCTGGAACGGGACGCCTTAGAGGGTGAGAGCCCCGTACGGTTGGATANCAAGCCT

CTGTAAAGCTCCTTCGACGAGTCGAGTAGTTCGGGAATGCTGCTCTAAATGGGAGGTAAN

TTTCTTCTNAAGCTAAATNCCGGCCAGAGNCCGATAGCGCACAAGTAGAGTGATCGAAAG

ATGAAAAGCACTTTGAANAGAGGGTTAAATAGCACGTGAAATTGNTGAAAGGGAAGCGTT

TNCGGNCAGACCTTTTCCCAGCGGNTCATNCAGTGNTTTCACTGGTGCACTCTGCTGGGT

TTAGNCCAGCATCGGCTTCTGTAGGGGGATAAAAGCCNNGGGAAAGTAGNTCCCTCGGGA

NTGTTATNNNNCTAGGCNTNANNCNNTNNCGGGGGCCGAGGNNCGCNCTCTGCAAGGANG

CTGGCGTAATGGTCGTCANCGACCCGTCTTGAAACACGGACCANGGAGTCGAACATTTGT

GCGAGTGTTTGGNTGTTAAACCCTCACGCGTAATGAAAGTGAACGGAGGTGAGAGCCCTT

CGGGGTGCATCATCGACCGATCCTGATGTCTTNGGATGGATTTGAGTAGGAGCATTAATG

TTCGGACCCGAAAGATGGTGANCTATGCGTGGATAGGGTGAAGCCAGAGGAAACTCTGGT

GGAGGCTCGCAGCGGNTCTGACGTGCAAATCGATCGTCAAATCNGCGCATGGGGGCGAAA

GACTTATCGAACCATCTAGTAGCTGGTTACCGCCGAAGTTTCCCTCAGGATAGCAGTGTT

GTTCTTCAGTTTTATGAGGTAAAGCGAATGATTAGGGACTCGGGGGCGCTTATTAGCCTT

CATCCATTCTCAAACTTTAAATATGTAAGAAGCCCTTGTTGCTTAGTTGAACGTGGGCAT

TCGAATGTACCAACACTAGTGGGCCATTTTTGGTAAGCAGAACTGNCGATGCGGGATGAA

CCGAACGCGGGGTTAAGGTGCCGGAGTGGACGCTCATCAGACACCACAAAAGGTGTTAAT

ACATCCAGACAGCCGGACGGTGGCCATGGAAGTCGGAATCCGCTAAGGACTGTGTAACAA

CTCACCGGCCGAATGTATTAGCCCTGAAAATGGATGNNGCTCAAGCGTCCCACCCATACC

TCGNCCTCAGGGNAGAAACGATGCCCTGAGGAGTAGGCGGCCGTGGAGGTTAGTGNNNNN

NNNNNNNNN

>Astrocystis_concavispora_MFLUCC_14.0174

NGACACCAACAGGGATTGCCCCAGTAGCGGCGAGCGAAGCGGCAACAGCTCAAATTTGAA

ATCTGGCCCTCGGGTCCGAGTTGTAATTTGTAGAGGATGCTCCTGGCGCGGTGCCTTCCG

AGTTCCCTGGAACGTTACGCCTGAGAGGGTGAGAGCCCCGTACGGTTGGACGCCAGGCCT

CTGTGCAGCTCCTTCGACGAGTCGAGTAGTTTGGGAATGCTGCTCTAAATGCAAGGTAAA

TTGCTTCTAGAGCTAAATATTGGCCAGAGACCGATAGCGCACAAGTAGAGTGATCGAAAG

ATGAAAAGCACTTTGAAAAGAGGGTTACCCAGCACGTGAAATTGTTGAAAGGGAAGCGTG

TGCGACCAGACCTCTTCCTGGCGGATCATCCGGCGTTCTCGCCGGTGCACTTCGCCAGGT

TCAGGCCAGCATCGGCTCTCGTCAGTCGACATAAGCTCTGGGAACGTAGCTCCCTCGCGA

GTGTTATAGCCCTCTGCACAATACCCTTGCGTAGCCCGAGGACCGCGCTTCGCAAGGATG

CTGGCGTAATGGTCGTCAACGACCCGTCTTGAGACACGGACCAAGGAGTCGAACATGTGT

GCGAGTGTTTGGGTGTCAAACCCTCACGCGTAATGAAGGTGAACGTAGGTGAGAGCCCTC

GCGGGCGCATCATCGACCGATCCTGATGTCTTCGGATGGATTTGAGTAAGAGCATGACTG

TTCGGAACCGATAGATGGTGAACTATGCGTGGATAGGGTGAAGCCAGAGGAAACTCTGGT

GGAGGCTCGCAGCGGTTCTGACGTGCAAATCGATCGTCAAATCTGCGCATGCTCGCGAAA

GACTTATCGAACCATCTAGTNNNNNNNNNNNNNNNNNNNNNNNNNNNNNNNNNNNNNNNN

NNNNNNNNNNNNNNNNNNNNNNNNNNNNNNNNNNNNNNNNNNNNNNNNNNNNNNNNNNNN

NNNNNNNNNNNNNNNNNNNNNNNNNNNNNNNNNNNNNNNNNNNNNNNNNNNNNNNNNNNN

NNNNNNNNNNNNNNNNNNNNNNNNNNNNNNNNNNNNNNNNNNNNNNNNNNNNNNNNNNNN

NNNNNNNNNNNNNNNNNNNNNNNNNNNNNNNNNNNNNNNNNNNNNNNNNNNNNNNNNNNN

NNNNNNNNNNNNNNNNNNNNNNNNNNNNNNNNNNNNNNNNNNNNNNNNNNNNNNNNNNNN

NNNNNNNNNNNNNNNNNNNNNNNNNNNNNNNNNNNNNNNNNNNNNNNNNNNNNNNNNNNN

NNNNNNNNNNNNNNNNNNNNNNNNNNNNNNNNNNNNNNNNNNNNNNNNNNNNNNNNNNNN

NNNNNNNNN

>Rhopalostroma_angolense_CBS_126414

NNNNNNNNNNNNNNNNNNNNNNNNNNNNNNNNNNNGAAGCGGCAACAGCTCAAATTTGAA

ATCTGGCCCTAGGGTCCGAGTTGTACTTTGTAGAGGATGCTTTTGGCGAGGTGCCTTCCG

AGTTCCCTGGAACGGGACGCCGGAGAGGGTGAGAGCCCCGTACGGTTGGACGCCGAGCCT

CTGTATAGCTCCTTCGACGAGTCGAGTAGTTTGGGAATGCTGCTCTAAATGGGAGGTAAA

TTTCTTCTAAAGCTAAATACCGGCCAGAGACCGATAGCGCACAAGTAGAGTGATCGAAAG

ATGAAAAGCACTTTGAAAAGAGGGTTAAATAGCACGTGAAATTGTTGAAAGGGAAGCGTT

TGCGACCAGACTTTTTCCGGGGGGATCATCCGGCGTTCTCGCCGGTGCACTTCCCCCGGT

CGAGGCCAGCATCGGTTTCCTTAGGGGGATAAAGGCCCGGGGAACGTGGCTCCTTCGGGA

GTGTTATAGCCCCGGGCGTAATACCCCTCGGGGGACCGAGGAACGCGCTCTGCAAGGATG

CTGGCGTAATGGTCGTCAACGACCCGTCTTGAAACACGGACCAAGGAGTCGAACATTTGT

GCGAGTGTTTGGGTGTCAAACCCTCACGCGTAATGAAAGTGAACGGAGGTGAGAGCCCTC

GCGGGTGCATCATCGACCGATCCTGATGTCTTCGGATGGATTTGAGTAAGAGCATAACTG

TTCGGACCCGAAAGATGGTGAACTATGCGTGGATAGGGTGAAGCCAGAGGAAACTCTGGT

GGAGGCTCGCAGCGGTTCTGACGTGCAAATCGATCGTCAAATCTGCGCATGGGGGCGAAA

GACTTATCGAACCATCTAGTAGCTGGTTACCGCCGAAGTTTCCCTCAGGATAGCAGTGTT

GTTCTTCAGTTTTATGAGGTAAAGCGAATGATTAGGGACTCGGGGGCGCTATATTGCCTT

CATCCATTCTCAAACTTTAAATATGTAAGAAGCCCTTGTTACTTAGTTGAACGTGGGCAT

TCGAATGTACCAACACTAGTGGGCCATTTTTGGTAAGCAGAACTGGCGATGCGGGATGAA

CCGAACGCGGGGTTAAGGTGCCAGAGTGGACGCTCATCAGACACCACAAAAGGTGTTAGT

ACATCTTGACAGCAGGACGGTGGCCATGGAAGTCGGAATCCGCTAAGGACTGTGTAACAA

CTCACCTGCCGAATGTACTAGCCCTGAAAATGGATGGCGCTCAAGCGTCTCACCCATACC

TCGCCCTTAGGGTAGAAACGATGCCCTAAGGAGTAGGCGGCCGTGGAGGTTAGTGACGAA

GCCTAGGGC

>Annulohypoxylon_truncatum_CBS_140778

AGAAACCAACAGGGATTGCCCTAGTAACGGCGAGTGAAGCGGCAACAGCTCAAATTTGAA

ATCTGGCCCTCGGGTCCGAGTTGTAATTTGCAGAGGATGCTTTTGGTGCGGTGCCTTCCG

AGTTCCCTGGAACGGGACGCCAGAGAGGGTGAGAGCCCCGTACGGTTGGACACCTACCCT

ATATATAGCTCCTTCGACGAGTCGAGTAGTTTGGGAATGCTGCTCTAAATGGGAGGTAAA

TTTCTTCTAAAGCTAAATACCGGCCAGAGACCGATAGCGCACAAGTAGAGTGATCGAAAG

ATGAAAAGCACTTTGAAAAGAGGGTTAAATAGCACGTGAAATTGTTGAAAGGGAAGCGTT

TGCGACCAGACTTTTTCCAGGCGGATCATCCGGTGTTCTCACCGGTGCACTTCGCCTGGT

TTAGGCCAGCATCGGTTTCCTTAGGGGGATAAAGGCCTGGGGAACGTAGCTCTCTAGGGA

GTGTTATAGCCCCTCGCGTAATACCCCTCGGGGGACCGAGGACCGCGCNNNNNNNNNNNN

NNNNNNNNNNNNNNNNNNNNNNNNNNNNNNNNNNNNNNNNNNNNNNNNNNNNNNNNNNNN

NNNNNNNNNNNNNNNNNNNNNNNNNNNNNNNNNNNNNNNNNNNNNNNNNNNNNNNNNNNN

NNNNNNNNNNNNNNNNNNNNNNNNNNNNNNNNNNNNNNNNNNNNNNNNNNNNNNNNNNNN

NNNNNNNNNNNNNNNNNNNNNNNNNNNNNNNNNNNNNNNNNNNNNNNNNNNNNNNNNNNN

NNNNNNNNNNNNNNNNNNNNNNNNNNNNNNNNNNNNNNNNNNNNNNNNNNNNNNNNNNNN

NNNNNNNNNNNNNNNNNNNNNNNNNNNNNNNNNNNNNNNNNNNNNNNNNNNNNNNNNNNN

NNNNNNNNNNNNNNNNNNNNNNNNNNNNNNNNNNNNNNNNNNNNNNNNNNNNNNNNNNNN

NNNNNNNNNNNNNNNNNNNNNNNNNNNNNNNNNNNNNNNNNNNNNNNNNNNNNNNNNNNN

NNNNNNNNNNNNNNNNNNNNNNNNNNNNNNNNNNNNNNNNNNNNNNNNNNNNNNNNNNNN

NNNNNNNNNNNNNNNNNNNNNNNNNNNNNNNNNNNNNNNNNNNNNNNNNNNNNNNNNNNN

NNNNNNNNNNNNNNNNNNNNNNNNNNNNNNNNNNNNNNNNNNNNNNNNNNNNNNNNNNNN

NNNNNNNNNNNNNNNNNNNNNNNNNNNNNNNNNNNNNNNNNNNNNNNNNNNNNNNNNNNN

NNNNNNNNNNNNNNNNNNNNNNNNNNNNNNNNNNNNNNNNNNNNNNNNNNNNNNNNNNNN

NNNNNNNNN

>Hypoxylon_fragiforme_MUCL_51264

NNNNNNNNNNNNNNNNNNNNNNNNNNNNNNNNNNNNNNNNNNNNNNNNNNNNNNNNNNNA

ATCTGGCCTCGTGGTCCGAGTTGTAATTTGTAGAGGATGCTTTTGGTGCGGTGCCTTCCG

AGTTCCCTGGAACGGGACGCCAGAGAGGGTGAGAGCCCCGTACGGTTGGACACCTACCCT

ATATATAGCTCCTTCGACGAGTCGAGTAGTTTGGGAATGCTGCTCTAAATGGGAGGTAAA

TTTCTTCTAAAGCTAAATACCGGCCAGAGACCGATAGCGCACAAGTAGAGTGATCGAAAG

ATGAAAAGCACTTTGAAAAGAGGGTTAAATAGCACGTGAAATTGTTGAAAGGGAAGCGTT

TGCGACCAGACTTTTTCCAGGGGGATCATCCGGTGTTCTCACCGGTGCACTCCGCCTGGT

TTAGGCCAGCATCGGTTCTCTTAGGGGGATAAAGGCTTGGGGAACGTAGCTCCTTCGGGA

GTGTTATAGCCCCTTGCGTAATACCTTC-GGGGGACCGAGGATCGCGCTCTGCAAGGATG

CTGGCGTAATGGTCGTCAACGACCCGTCTTGAAACACGGACCAAGGAGTCGAACATTTGT

GCGAGTGTTTGGGTGTTAAACCCTCACGCGTAATGAAAGTGAACGGAGGTGAGAGCCCTT

ACGGGTGCATCATCGACCGATCCTGATGTCTTCGGATGGATTTGAGTAAGAGCATAACTG

TTCGGACCCGAAAGATGGTGAACTATGCGTGGATAGGGTGAAGCCAGAGGAAACTCTGGT

GGAGGCTCGCAGCGGTTCTGACGTGCAAATCGATCGTCAAATCTGCGCNNNNNNNNNNNN

NNNNNNNNNNNNNNNNNNNNNNNNNNNNNNNNNNNNNNNNNNNNNNNNNNNNNNNNNNNN

NNNNNNNNNNNNNNNNNNNNNNNNNNNNNNNNNNNNNNNNNNNNNNNNNNNNNNNNNNNN

NNNNNNNNNNNNNNNNNNNNNNNNNNNNNNNNNNNNNNNNNNNNNNNNNNNNNNNNNNNN

NNNNNNNNNNNNNNNNNNNNNNNNNNNNNNNNNNNNNNNNNNNNNNNNNNNNNNNNNNNN

NNNNNNNNNNNNNNNNNNNNNNNNNNNNNNNNNNNNNNNNNNNNNNNNNNNNNNNNNNNN

NNNNNNNNNNNNNNNNNNNNNNNNNNNNNNNNNNNNNNNNNNNNNNNNNNNNNNNNNNNN

NNNNNNNNNNNNNNNNNNNNNNNNNNNNNNNNNNNNNNNNNNNNNNNNNNNNNNNNNNNN

NNNNNNNNNNNNNNNNNNNNNNNNNNNNNNNNNNNNNNNNNNNNNNNNNNNNNNNNNNNN

NNNNNNNNN

>Hypocreodendron_sanguineum_J.D.R._169

------------------------------------------------------------

------------------------------------------------------------

------------------------------------------------------------

------------------------------------------------------------

------------------------------------------------------------

------------------------------------------------------------

------------------------------------------------------------

------------------------------------------------------------

------------------------------------------------------------

------------------------------------------------------------

------------------------------------------------------------

------------------------------------------------------------

------------------------------------------------------------

------------------------------------------------------------

------------------------------------------------------------

------------------------------------------------------------

------------------------------------------------------------

------------------------------------------------------------

------------------------------------------------------------

------------------------------------------------------------

------------------------------------------------------------

------------------------------------------------------------

---------

>Thamnomyces_dendroidea_CBS_123578

NNNNNNNNNNNNNNATTGCCCTAGTAACGGCGAGTGAAGCGGCAACAGCTCAAATTTGAA

ATCTGGCCCTAGGGTCCGAGTTGTACTTTGCAGAGGATGCTATGGGCGAGGCGCCTTCCG

AGTTCCCTGGAACGGGACGCCAGAGAGGGTGAGAGCCCCGTACGGTGGGACGCGTCGCCT

GCGTATAGCTCCTTCGACGAGTCGAGTAGTTTGGGAATGCTGCTCTAAACGGGAGGTAAA

TTTCTTCTAAAGCTAAATACCGGCCAGAGACCGATAGCGCACAAGTAGAGTGATCGAAAG

ATGAAAAGCACTTTGAAAAGAGGGTTAAACAGCACGTGAAATCGTTGAAAGGGAAGCGTT

TGCGACCAGACGTCGCCTAGGGGGATCATCCGGCAGCCCTGCCGGTGCACTTCCGCTAGG

CGAGGCCAGCGTCGGCTTCCTCAGGGGGATAAAGGCCCGGGACACGTATCTCCTTCGGGA

GTGTTATAGCCCCGGGCGTACTACCCCTGAGGGGACCGAGGAACGCGTTCTGCAAGGACG

CTGGCGTAATGGTCGTCAATGACCCGTCTTGAAACACGGACCAAGGAGTCGAACGTTTAT

GCGAGTGTACGGGTGTCAAACCCTGGCGCGTAATGAAGGTGAACGGAGGTGAGAGCCCTT

CGGGGTGCATCATCGACCGATCCTGATGTCTTCGGATGGATTTGAGTAAGAGCATATGCG

TTCGGACCCGAAAGATGGTGAACTATGCGCGGATAGGGTGAAGCCAGAGGAAACTCTGGT

GGAGGCTCGCAGCGGTTCTGACGTGCAAATCGATCGTCAAATCTGCGCATGGGGGCGAAA

GACTTATCGAACCATCTAGTAGCTGGTTACCGCCGAAGTTTCCCTCAGGATAGCAGTGTT

G-TCTTCAGTTTTATGAGGTAAAGCGAATGATTAGGGACTCGGGGGCGCTATACTGCCTT

CATCCATTCTCAAACTTTCAATATGTAAGAAGCCCCCGTTGCTTAGCTGAACGGGGGCCT

TCGAATGTTGCAACACTAGTGGGCCATTTTTGGTAAGCAGAACTGGCGATGCGGGATGAA

CCGAACGTGGGGTTAAGGTGCCAGAGTAGACGCTCATCAGATACCACAAAAGGTGTTAGT

ACATCTTGACAGCAGGACGGTGGCCATGGAAGTCGGAATCCGCTAAGGACTGTGTAACAA

CTCACCTGCCGAATGTACTAGCCCTGAAAATGGATGGCGCTCAAGCGTCTCACCTATACC

CCGCCCTTAGGGTAGAAACGAGGCCCTAAGGAGTAGGCGGCCGTGGAGGTCAGTGACGAA

GCCTAGGNN

>Pyrenopolyporus_hunteri_MUCL_52673

NNNNNNNNNNNNNNNNNNNNNNNNNNNNNNNNNNNNNNNNNNNNNNNNNNNNNNNNNNNN

NNNNNNNNNNNNNNNNNNNNNNNNNNNNNNNNNNNNNNGCTTTGGGCGCGGCGCCTTCCA

AGTTCCCTAGAACGGGACGCCTTAGAGGGTGAGAGCCCCGTACGGTTGGACGCCTAGCCT

ATGTATAGCTCCTTCGACGAGTCGAGTAGTTTGGGAATGCTGCTCTAAATGGGAGGTAAA

TTTCTTCTAAAGCTAAATACCGGCCAGAGACCGATAGCGCACAAGTAGAGTGATCGAAAG

ATGAAAAGTACTTTGAAAAGAGGGTTAAATAGCACGTGAAATTGTTGAAAGGGAAGCGTT

TGCGACCAGACCTTTTCCAGGCGGATCATCCGGCGTTCTCGCCGGTGCACTTCGCCTGGT

TTAGGCCAGCATCGGTTTTCCCAGGGGGATAAAGGCGGTGGGAACGTAGCTCTTTCGGGA

GTGTTATAGCCCGCCGCGTAATACCCTTGGGGGGACCGAGGACCGCGCTTCGCAAGGATG

CTGGCGTAATGGTCGTCAACGACCCGTCTTGAAACACGGACCAAGGAGTCGAACATTTGT

GCGAGTGTTTGGGTGTCAAACCCTCACGCGTAATGAAAGTGAACGGAGGTGAGAGCCCCT

GCGGGTGCATCATCGACCGATCCTGATGTCTTCGGATGGATTTGAGTAAGAGCATAACTG

TTCGGACCCGAAAGATGGTGAACTATGCGTGGATAGGGTGAAGCCAGAGGAAACTCTGGT

GGAGGCTCGCAGCGGTTCTGACGTGCAAATCGATCGTCAAATCTGCGCATGGGGGCGAAA

GACTTATCGAACCATCTAGTAGCTGGTTACCGCCGAAGTTTCCCTCAGGATAGCAGTGTT

G-TCTTCAGTTTTATGAGGTAAAGCGAATGATTAGGGACTCGGGGGCGCTATTTTGCCTT

CATCCATTCTCAAACTTTAAATATGTAAGAAGCCCTTGTTACTTAGTTGAACGTGGGCAT

TCGAATGTACCAACACTAGTGGGCCATTTTTGGTAAGCAGAACTGGCGATGCGGGATGAA

CCGAACGCGGGGTTAAGGTGCCAGAGTGGACGCTCATCAGACACCACAAAAGGTGTTAGT

ACATCTTGACAGCAGGACGGTGGCCATGGAAGTCGGAATCCGCTAAGGACTGTGTAACAA

CTCACCTGCCGAATGTACTAGCCCTGAAAATGGATGGCGCTCAAGCGTCTCACCCATACC

TCGCCNNNNNNNNNNNNNNNNNNNNNNNNNNNNNNNNNNNNNNNNNNNNNNNNNNNNNNN

NNNNNNNNN

>Rostrohypoxylon_terebratum_CBS_119137

NNNNNNNNNNNNNNNNNGCCCTAGTAACGGCGAGTGAAGCGGCAAAAGCTCAAATTTGAA

ATCTGGCCCTAGGGTCCGAGTTGTAATTTGCAGAGGATGCTTTCGGTGCGGTGCCTTCCG

AGTTCCCTGGAACGGGACGCCATAGAGGGTGAGAGCCCCGTACGGTTGGACGCCTAGCCT

CTATAAAGCTCCTTCGACGAGTCGAGTAGTTTGGGAATGCTGCTCTAAATGGGAGGTAAA

TTTCTTCTAAAGCTAAATACCGGCCAGAGACCGATAGCGCACAAGTAGAGTGATCGAAAG

ATGAAAAGCACTTTGAAAAGAGGGTTAAATAGCACGTGAAATTGTTGAAAGGGAAGCGTT

TGCGACCAGACCTTCTCCGGGGGGATCACCCGCTGTTCTCAGCGGTGCACTTCCCTCGGT

TTAGGCCAGCATCGGTTCTCTTAGGGGGATAAAGGCCTGGGGCACGTAGCTCTTTAGGGA

GTGTTATAGCCCCTGGCGTAATACCTTTCAGGGGACCGAGGACCGCGCTTCGCAAGGATG

CTGGCGTAATGGTCGTCAACGACCCGTCTTGAAACACGGACCAAGGAGTCGAACATTTGT

GCGAGTGTTTGGGTGTTAAACCCTCACGCGTAATGAAAGTGAACGGAGGTGAGAGCCTT-

-AGGGTGCATCATCGACCGATCCTGATGTCTTCGGATGGATTTGAGTAAGAGCATAACTG

TTCGGACCCGAAAGATGGTGAACTATGCGTGGATAGGGTGAAGCCAGAGGAAACTCTGGT

GGAGGCTCGCAGCGGTTCTGACGTGCAAATCGATCGTCAAATCTGCGCATGGGGGCGAAA

GACTTATCGANNNNNNNNNNNNNNNNNNNNNNNNNNNNNNNNNNNNNNNNNNNNNNNNNN

NNNNNNNNNNNNNNNNNNNNNNNNNNNNNNNNNNNNNNNNNNNNNNNNNNNNNNNNNNNN

NNNNNNNNNNNNNNNNNNNNNNNNNNNNNNNNNNNNNNNNNNNNNNNNNNNNNNNNNNNN

NNNNNNNNNNNNNNNNNNNNNNNNNNNNNNNNNNNNNNNNNNNNNNNNNNNNNNNNNNNN

NNNNNNNNNNNNNNNNNNNNNNNNNNNNNNNNNNNNNNNNNNNNNNNNNNNNNNNNNNNN

NNNNNNNNNNNNNNNNNNNNNNNNNNNNNNNNNNNNNNNNNNNNNNNNNNNNNNNNNNNN

NNNNNNNNNNNNNNNNNNNNNNNNNNNNNNNNNNNNNNNNNNNNNNNNNNNNNNNNNNNN

NNNNNNNNNNNNNNNNNNNNNNNNNNNNNNNNNNNNNNNNNNNNNNNNNNNNNNNNNNNN

NNNNNNNNN

# *rpb2*

>Lopadostoma_turgidum_CBS_133207

-------------------------------TGCTAGCCAAGCTCTTCCGCAACATCGTG

CGTAGGTTGACCCAGGAGATCACTATGCATCTGAAGCGCTGTGTTGAGCAAGGCAAGCAC

TTCCAGCTGCCTATGGCCGTCAAGCCTCAGATCGTCACCAACGGGCTGAAATACTCCCTC

GCCACTGGTAATTGGGGTGACCAGAAAAAGGCAATGAGCTCCACCGCTGGTGTCTCGCAG

GTCCTGAACAGATACACCTTCTCCTCTACCTTGTCACATTTGAGGAGAACCAACACTCCT

GTTGGACGAGACGGCAAGCTCGCAAAGCCTCGCCAGCTTCACAACACGCACTGGGGTCTG

GTGTGCCCGGCGGAAACGCCCGAGGGTCAGGCTTGTGGTCTGGTCAAGAACTTGTCTCTC

ATGTGCTCCATCAGCGTGGGAACGTCGACCGAGCCTATCATCGACTACATGATTACTCGG

AACATGGAGGTTTTGGAAGAGTACGAACCGCTTCGGTATCCCAATGCTACCAAGATCTTC

CTGAACGGTTCTTGGATTGGTGTGCACCAAGACGCCAAGGCTCTTGTCAGAGATGTCCAG

CAGCTGCGCCGAAATAACCAGATCCCTGCCGAGGTATCCTTGATCCGCGACATTCGCGAT

CGTGAATTCAAGATTTTCTCAGACGCTGGTCGAGTCATGCGCCCCCTCTTCGTCGTCGAA

CAGGAGGACAACCCCGAGACTGGTGTGGAGAAGGGTACCCTGGTGCTTACCAAGGACATG

GTGCGA------------CGGCTCGAAGTAGACCAGAGTCTACCCGCCGGAAGT------

---GACCAG---TTTTTCGGCTGGCAGGGCTTGGTGAACGAAGGTGTTATTGAGTACCTC

GACGCTGAGGAGGAAGAGACGGCCATGATTTGCATGACGCCGGAAGATCTCGAAATCTAC

CGCATGTCCAAATTGGGCCACGAGATATCAACT------------GACAAC---------

------GGAGACGACCTGAATAAGCGACTCAAGACGAAGATCAATCCGACTACGCACATG

TATACGCATTGCGAGATCCATCCTAGCATGCTTCTAGGTATTTGTGCCAGTATCATCCCC

TTCCCAGACCATAATCAGGTACGCATCCCGCTTTAGATCCTTG-----CTAAAC---CTA

T-CATA-----------------------------

>Creosphaeria_sassafras_ST.MA._14087

------------------------------------------------------------

----------------------------------AGCGATGCGTCGAGCAAGGCAAGCAC

TTCCAGTTGCCTATGGCCGTCAAACCCCAGATTGTCACCAACGGCCTGAAATACTCGCTC

GCCACCGGCAACTGGGGTGATCAGAAGAAGGCGATGAACACCACCGCCGGTGTCTCCCAG

GTCCTGAACAGATACACATTCTCGTCCACGCTGTCACATCTGAGAAGAACCAACACCCCC

ATCGGGCGAGACGGCAAGCTCGCAAAGCCCCGTCAGCTCCACAACACGCACTGGGGTCTG

GTGTGCCCGGCAGAAACCCCCGAGGGCCAGGCCTGCGGTCTGGTCAAGAACTTGTCTCTC

ATGTGCTCCATCAGCGTGGGAACGTCGACCGAGCCCATCATCGACTACATGATCACCCGG

AACATGGAGGTATTGGAGGAATATGAACCCCTCCGCTACCCGAACGCCACCAAGATCTTC

TTGAACGGTTCCTGGATTGGCGTACATCAGGACGCCAAGGCTCTCGTCCGAGATGTCCAG

CAGCTGCGCCGAAACAACCAGATCCCTGCTGAGGTATCGCTGGTCCGCGACATCCGCGAT

CGCGAGTTCAAGATATTCTCGGATGCCGGCCGCGTCATGCGCCCCGTCTTCGTAGTCGAG

CAGGAGGACAACCCCGAGACGGGTGTCGAAAAGGGTACCCTGGTGCTCACCAAGGACATG

GTGCGA------------CGACTCGAGATAGACCAGACTCTGCCCCCGGGAAGC------

---GATGAA---TTCTTCGGCTGGCAGGGCCTGGCGAACGAAGGCGTCATCGAGTATCTG

GATGCCGAGGAGGAGGAGACGGCCATGATCTGCATGACGCCCGAGGACCTCGAGATATAC

CGCATGTCGAAGCTGGGCTACAACATGTCGACC------------GACACC---------

------GGGGACGAGCCCAACAAGCGGCTGAAGACGAAGATCAACCCGACCACGCACATG

TACACGCACTGCGAGATCCATCCCAGTATGCTTCTAGGTATCTGTGCTAGTATCATCCCC

TTCC--------------------------------------------------------

-----------------------------------

>Diatrype_disciformis_CBS_197.49

------------------------------------------------------------

------------------------------------------------------------

------------------------------------------------------------

------------------------------------------------------------

-------------ACACCTTTTCGTCTACCTTATCACATTTGCGGAGAACCAACACACCT

ATCGGAAGAGATGGAAAGCTCGCTAAGCCTCGCCAGCTTCACAACACACATTGGGGTTTA

GTCTGCCCGGCAGAGACGCCTGAGGGTCAGGCCTGTGGCCTGGNGAAGAACCTGTCCNTG

ATGTGCTCCATCAGTGTTGGTACATCCACCGAGCCCATCATCGACTACATGATTACTAGA

AATATGGAGGTTCTCGAAGAATACGATGCCGCACGCTATCCTAATGCAACCAAGATCTTC

CTGAACGGCTCCTGGATCGGTGTGCACCAGGATCCTAAGTCTCTCGTCAAGGATGTTCAG

CAGCTGCGACGAACGAACCAGATCCCTGCTGAGGTCTCCTTGATCCGTGATATTCGCGAT

CGTGAGTTCAAGATCTTCTCGGATGCCGGTCGAGTTATGCGTCCGCTGTTCGTCGTCGAG

CAAGAGGATGACCCCGAGAGGAATGCCGAGAAGGGTACCTTGGTTCTCACCAAGGACATG

GTGCGT------------CGGCTTGAGATAGATCAGACGCTCCCACCGGGAAGC------

---GACGAG---TTCTTCGGTTGGCAAGGCCTCGTAAACGAAGGTGTTATCGAGTATCTT

GATGCCGAGGAGGAAGAGACGGCCATGATCTGTATGACTCCGGAAGATCTTGATCTCTTC

AAGATCGCCAAGGCTGGCCAGCCAGTCAACGAG------------GACAAC---------

------ACAGACGAACCTAATAAGCGAGTCAAGACAAAGATGAGCATGACCACTCACACG

TACACGCATTGCGAGATTCATCCCAGTATGCTCTTGGGCATTTGCGCCAGTATTATTCCT

TTCCCCGATCATAATCAAGTATGCACGCTTGCTTTTGATTGATGCTTATTGACA---TTC

AAAATAGTCTCCTCGAAATACCTATCAATCGGCTA

>Eutypa_lata_UCR_EL1

GGCAAGAAGCGTCTTGATCTTGCCGGCCCCTTGCTTGCTAAGCTGTTCCGGAACATCATA

CGCCGACTGACGCAGGAGATAACTATGCATTTGAAGCGTTGTATTGATCAGAACAAGTTG

TTCCAGATCCACATGGCTGTCAAACCTCAGATAGTAACGAACGGTTTGAAGTACTCGCTG

GCTACTGGAAATTGGGGTGATCAGAAGAAGGCGATGAGTTCAACTGCAGGTGTGTCGCAG

GTCCTGAACAGATATACCTTCTCGTCTACTCTATCACATTTGAGGAGAACGAACACGCCT

ATTGGAAGAGATGGGAAGCTCGCCAAGCCTCGCCAACTTCATAACACACATTGGGGCTTG

GTCTGCCCGGCTGAAACGCCCGAGGGCCAGGCCTGTGGTCTGGTGAAAAACCTGTCTTTG

ATGTGCTCCATCAGTGTGGGCACATCAACCGAGCCCATCATCGACTACATGATTACCCGG

AACATGGAGGTCTTGGAAGAGTATGACGCTGCACGCTACCCTAATGCTACCAAGATCTTC

TTGAACGGCTCTTGGATCGGTGTGCACCAGGACCCTAAGTCTCTCGTCAAGGATGTTCAA

CAGCTGCGACGAACTAACCAGATTCCCGCCGAAGTCTCCTTGATCCGCGATATCCGCGAC

CGTGAGTTCAAGATCTTCTCGGATGCTGGACGCGTTATGCGCCCACTGTTTGTCGTTGAA

CAAGAGGATGACCCCGAGAGGGGTGCCGAGAAGGGTACCTTGGTGCTTACGAAGGATATG

GTGCGT------------CGGCTTGAGATGGATCAGACGCTTCCACCAGGAAGC------

---GATGAA---CTATTCGGCTGGCAAGGTCTCGTGCACGAAGGTGTTATTGAGTATCTT

GATGCCGAGGAAGAGGAGACGGCCATGATCTGTATGACACCGGAAGATCTTGAGATTTTC

CGGCTGGCCAAGCGAGGCGAGCCGGTTAACGAG------------GACATC---------

------ATGGATGAACCTAATAAGCGAGTCAAGACAAGGTTAAACAAGACCACACACACA

TATACGCATTGCGAGATCCACCCCAGTATGCTCTTGGGAATTTGCGCCAGTATCATTCCC

TTCCCCGATCATAACCAGGTATGCACGCT--C--ATTATTCATGCTAACT---T---TTG

CATCCAGTCGCCGCGTAATACTTATCAATCGGCTA

>Entoleuca_mammata_J.D.R._100

GGCAAGAAGCGACTGGACCTCGCCGGTCCTTTGCTCGCCAAGCTCTTCCGCAACATAGTG

CGCCGGATGACTCAGGAGGTCACCTCCCATCTGAAGCGAAGCATCGAGCAGGGCAAGCAG

TTCAATATTGCTTTGGCCGTCAAGTCAAACATCATCACGAGTGGACTGAAGTATTCTCTT

GCCACCGGAAACTGGGGTGATCAGAAGAAGGCCATGAGCTCTACGGCTGGTGTGTCTCAG

GTCCTCAACCGATACACATTTGCGTCCACTCTATCCCATCTGCGGAGAACAAATACCCCC

GTGGGCAGAGATGGTAAACTGGCCAAGCCGCGGCAGCTTCACAACACACATTGGGGCCTC

GTGTGCCCGGCCGAGACACCCGAGGGTCAGGCTTGTGGTCTGGTCAAGAATCTGTCCCTC

ATGTGCTCCGTTAGCGTGGGCACTTCGACGGAACCAATTATAGAATATATGATTTCCCGA

AATATGGAGGTTCTAGAAGAGTACGAACCCCAACGATACCCCAACGCTACGAAGATCTTT

CTCAACGGGTCCTGGATCGGTGTTCACCAGGATCCGAAGGCTCTCGTTAAAGACGTTCAA

CAGTTGCGCCGAACAAACCAGATCCCGGCCGAGGTATCTCTGGTTCGTGATATCCGTGAT

CGCGAATTCAAAATCTTTTCGGACGCCGGTCGTGTCATGCGTCCATTGTTCGTGGTTGAA

CAGGAGGATGATCCGGATAACGGTATCGAGAAGAACACGCTCGTCCTAACGAAAGACATG

GTCCGC------------AGGCTGGAGATTGACCAGACTCTCCCACCAGAAAGT------

---GACGAA---TATTTCGGCTGGCAAGGCTTGGTCAACGCCGGTGTCATCGAATATATG

GATGCCGAGGAAGAAGAAACTGCCATGATCTCCATGGCTCCTGAAGACTTGGAGGCATTC

AGGAGATCCAAATTAGGTCTCCCTAGTGGCGACCCAGAA------TACGCC---------

------ATGACCAACCCAAACAGACGGTTGAACACGAGGATCAACCCGACAACCCACGGG

TATACCCATTGCGAAACTCACCCAAGTATGCTCCTCGGCATTTGTGCCAGTATTATCCCC

TTCCCCGATCATAACCAGGTAAGCCTCTTACCATTCCATCCATAGCCACCGTGGACACGA

GTTATAGTCACCCCGAAACACGTACCAGTCTGCTA

>Rosellinia_corticium_MUCL_51693

------------------------------------------------------------

------------------------------------------------------------

------------------------------------------------------------

------------------------------------------------------------

----------------CATTCGCGTCCACTCTATCCCATCTGCGGAGAACGAATACCCCC

GTGGGCAGAGACGGTAAACTAGCCAAGCCGCGGCAACTTCATAACACACATTGGGGCCTC

GTGTGTCCGGCCGAGACACCCGAGGGCCAGGCCTGTGGTCTGGTCAAGAATCTGTCCCTC

ATGTGCTCCGTCAGTGTGGGCACGTCGACGGAACCCATTATAGAATACATGATTTCTCGA

AATATGGAGGTGCTGGAAGAGTACGAGCCCCAACGGTACCCCAATGCCACGAAGATCTTC

CTCAACGGATCGTGGATCGGTGTTCACCAGGATCCGAAGGCTCTCGTGAAGGACGTTCAA

CAGCTGCGCCGAACAAACCAGATCCCGGCCGAAGTGTCGCTGATTCGTGATATCCGTGAT

CGCGAATTCAAAATCTTCTCGGACGCCGGTCGTGTCATGCGTCCACTGTTTGTAGTTGAG

CAGGAAGATGATCCGGATAATGGTGTCCAGAAGAACACGCTCGTCCTAACGAAAGACATG

GTCCGT------------AGGCTGGAGATCGACCAGACTCTCCCACCAGAGAGT------

---GACGAA---TATTTCGGTTGGCAAGGCTTGGTCAACGCCGGTGTCATTGAATACATG

GATGCCGAGGAAGAAGAAACCGCCATGATCTCCATGGCCCCCGAAGACCTGGAGGCTTTC

AGGAGATCCAAATTGGGTCTCCCGAGTGGCGACCCAGAA------TACGCC---------

------ATGACCAACCCAAACAGACGGTTGAACACGAGGATCAATCCAACTACCCACGGA

TATACCCATTGCGAAATTCACCCAAGTATGCTCCTCGGTATCTGTGCCAGCATTATTCCC

TTCCCCGATCATAACCAGGTAAGCTCCTCACCATTCCATCCATAACCAGCATCAATACTA

ACAACAAG---------------------------

>Xylaria_hypoxylon_CBS_122620

------------------------------------------------------------

------------------------------------------------------------

------------------------------------------------------------

------------------------------------------------------------

------------------------------TTGTCGCATTTGCGAAGAACGAATACTCCA

GGTGGTAGAGACGGTAAGCTTGCGAAACCACGGCAGCTCCACAACAGCCACTGGGGTCTC

GTCTGCCCGGCCGAGACCCCCGAAGGTCAGGCTTGTGGCCTAGTCAAAAACCTGTCCCTC

ATGTGCTCCATCAGTGTTGGTACGTCCACGGAACCTATTATCGATTATATGATATCGCGT

AATATGGAGGTTTTAGAGGAGTACGACCATCACAGATACCCTAATGCCACCAAGATCTTT

CTCAATGGTGCATGGATCGGCGTCCACCAGGATCCTAAGTCTCTTGTGAGGGATGTGCAA

CAATTGCGCCGAACGAATCAGATTCCCGCTGAAGTATCCTTGATTCGAGATATTCGTGAC

CGCGAATTCAAAATCTTCTCGGATGCCGGCCGTGTCATGCGGCCCCTGTATGTGGTTGAG

CAGGAGGATGACGCTGAGAATGGTGTCGAGAAGGGCACACTGGTCTTAACAAAGGACATG

GTTCGG------------AGACTTGAGATCGACCAGACCCTCCCACCCGGAAGT------

---GACGAA---TACTTCGGATGGCATGGCCTGGTTCATGCTGGTGTCATCGAATACATG

GATGCTGAAGAGGAGGAGACTGCGATGATTTGCATGACCCCCGAGGACTTGGAAAGTTTC

CGATGCTCCAAGTTAGGTTTACCAGATCCTTTCAACAAC------GAGGAC---------

------ACTTTCGCCCCAAACAAGCGGCTGAAGACGAGAATAAACCCGACAACCCATATG

TACACTCACTGTGAAATCCACCCGAGCATGCTTCTTGGCATTTGTGCCAGTATCATCCCC

TTCCCCGATCATAATCAAGTAAGTGCCACGCAATCCGTATCGCCGCAATTATTGATCCCA

GTTACAGTCACCCCGAAACACATACCA--------

>Xylaria_arbuscula_CBS_126415

------------------------------------------------------ATTGTT

CGGCGGATGACCCAGGAGGTTCTGTCGCACCTGAAGCGGAGCATCGAGCAAGGCAAGCAA

TTCAATATTGCACTAGCTGTGAAGTCTAACATAATCACGAGTGGGTTGAAGTACTCACTC

GCTACCGGCAACTGGGGTGACCAGAAAAAGGCAATGAGCTCCACAGCCGGTGTTTCGCAA

GTGTTGAATCGATACACATTCGCCTCTACCTTGTCACACTTGCGAAGAACAAATACCCCA

GTCGGTAGAGATGGCAAGCTTGCCAAACCCCGACAACTTCACAATACCCACTGGGGGCTT

GTCTGTCCAGCCGAGACCCCAGAAGGACAGGCATGTGGTTTGGTCAAAAACTTGTCGCTC

ATGTGCTCTATCAGCGTCGGTACATCAACGGATCCTATTATAGAATATATGATCCTTAGG

AATATGGAAGTGCTAGAAGAGTATGATCCTGGTAGGTATCCCAACGCCACCAAGGTGTTT

CTTAATGGTGCATGGATCGGCGTCCACCAGGATCCCAAGGCTCTAGTTAAGGATGTGCAA

CAATTGCGCCGAACAAACCAGATCCCAGCTGAAGTATCCCTCATCCGGGATATTCGCGAC

CGTGAGTTCAAGATTTTCAGTGACGCCGGTCGCGTCATGCGCCCTCTGTTCGTAGTCGAG

CAAGAAGACGACGCTGAAAGGGGCATTGAGAAAAGCACGCTCGTTTTGACCAAAGATATG

GTTCGG------------CGGCTTGAGGAAGACCAGAGCCTTCCACCCGGACAC------

---GAAGAT---TACTATGGATGGCAAAGTTTGGTTAATGCCGGTGTGATTGAATATATG

GACGCTGAAGAAGAGGAGACGGCGATGATCTGCATGACCCCGGAAGACCTAGAGAGTTTC

CGATGCAGCAAGTTGGGTCTAGCAGATCCTCACAACAAC------GATGAT---------

------GTCTTTGCTCCCAACAAGCGGCTGAAGACGAGGATAAATCCGACCACACACATG

TACACTCACTGTGAAATTCATCCGAGCATGCTTCTAGGCATTTGTGCCAGTATCATTCCC

TTCCCGGATCACAACCAAGTAAGTATCAGATAACTATGCTTATGCCAACTGC--------

-----------------------------------

>Oligostoma_insidiosum_CBS_147288

NNNNNNNNNNNNNTGGATCTTGCTGGACCTTTGCTCGCCAAACTTTTCCGCAACATAGCT

CGCCGCATGACCCAGGAGGTCTTGTCACATCTCAAGCGAAGTATCGAGCAAGGCAAGCAG

TTCAATATTGCCCTGGCCGTCAAATCGAATATCATCACGAGCGGATTGAAATATTCTCTC

GCCACAGGCAACTGGGGTGATCAGAAAAAGGCCATGAGTTCTACTGCTGGCGTTTCGCAG

GTGTTGAACAGATACACCTTCGCATCTACGCTCTCACATTTACGTAGAACAAATACACCG

GTTGGTAGAGATGGCAAGCTTGCTAAACCGCGGCAGCTTCACAACACCCACTGGGGCCTG

GTCTGCCCGGCCGAGACACCTGAAGGTCAGGCTTGTGGTTTAGTGAAAAACCTGTCACTT

ATGTGCTCTATCAGCGTGGGTACGTCGACGGAGCCTATTATAGAATATATGATTTCGCGA

AATATGGAGGTCCTGGAAGAGTATGAACCCCAAAGGTACCCGCATGCCACCAAGATCTTT

CTCAACGGATCATGGATTGGTATCCATCAAGATCCAAAAGCTCTCGTCAGAGATGTTCAA

CAATTGCGTCGGACGAATCAGATTCCGGCTGAAGTATCCTTGATTCGGGATATTCGGGAT

CGTGAATTCAAGATATTCTCAGATGCTGGCCGTGTCATGCGGCCATTGTTTGTGGTCGAG

CAAGACGATGATCCTGACAACCATATCGAGAAAGGCACACTGGTCCTGAAAAAGGACATG

GTCCGG------------CGGCTCGAGATTGACCAGACCCTTCCACCCGGAAGT------

---GATGAA---TATTTCGGATGGGATGGCTTGGTCAATGCCGGTGTCATCGAATACATG

GATGCAGAGGAGGAAGAAACCGCCATGATTTGCATGACCCCCGAAGACTTGGAGGCTTTC

AGGCTGACCAAGCTGGGCCAATTGGATCCTGACGCTGAA------TCCAAT---------

------TTAAACGAGCCGAATAAACGACTAAAGACAAAGATGAATCCGACGACTCATACA

TACACGCATTGTGAAATTCACCCAAGTATGCTTCTTGGTATCTGCGCCAGCATCATTCCC

TTCCCTGATCATAACCAGGTAAGTTCTCTGCAGTCGGTAGCATCGTCGTCGTTAATCCCA

GTTCTAGTCACCCGAAAACAAATACCAGTCCGCCN

>Xylaria_laevis_HAST_419

GGTAAGAAGCGGCTAGACCTTGCCGGACCTCTGCTCGCCAAGCTCTTTCGCAACATAGCA

CGTCGGATGACTCAGGAGGTTTTATCACATCTCAAGCGAAGTATTGAGCAAGGCAAGCAG

TTCAATATTGCCCTCGCCGTCAAGTCAAATATCATTACGAGCGGCTTGAAGTATTCTCTC

GCTACTGGCAACTGGGGTGATCAGAAGAAGGCCATGAGCTCCACTGCTGGTGTTTCGCAG

GTGTTGAACAGATACACATTCGCATCTACCTTGTCACATTTACGAAGAACGAATACTCCG

GTTGGCAGAGATGGTAAGCTTGCCAAGCCACGGCAGCTTCACAACACCCATTGGGGTCTG

GTCTGCCCGGCCGAGACGCCTGAAGGCCAGGCTTGTGGCTTAGTCAAGAACCTTTCCCTA

ATGTGTTCCATCAGTGTGGGTACGTCGACGGAACCTATTATAGAATATATGATTTCGCGA

AATATGGAGGTTCTGGAAGAGTATGAACCTCAAAGGTACCCACATGCCACTAAGATTTTT

CTCAATGGATCGTGGATTGGTATTCACCAAGATCCGAAAGCCCTCGTCAGAGACGTTCAG

CAGTTGCGACGGACGAATCAGATTCCGGCTGAGGTATCTCTAATTCGGGATATCCGTGAT

CGCGAATTCAAGATCTTCTCAGATGCCGGCCGTGTCATGCGGCCCTTGTTTGTGGTCCAG

CAAGAAGATGATCCTGACAACGGTATCGAAAAGGGCACATTAGTCTTGACAAAAGACATG

GTCCGA------------CGGCTTGAAATTGATCAAACACTTCCACCTGGAAGC------

---GAAGAA---TATTTCGGATGGCAAGGCTTGGTCAATGCCGGTGTCATTGAATATATG

GATGCTGAGGAGGAAGAAACAGCGATGATTTGCATGACTCCTGAAGACCTGGAAGCTTTC

AGAATGACTAAGCTGGGCCTGCCGGATCCTGACGCAGAA------TCTAGC---------

------TTGAATGCGCCCAACAAACGACTCAAGACGAGGATGAATCCAACAACTCATACG

TACACCCATTGTGAAATTCATCCCAGTATGCTTCTTGGTATTTGTGCCAGCATCATTCCC

TTCCCTGATCACAATCAGGTAAGTGTTATATAGTCCATTGCACCGTAGTGACTGACCGCA

GTTATAGTCACCTCGAAATACGTACCAGTCCGCTA

>Xylaria_longipes_CBS_148.73

------------------------------------------------------------

-----------------------------------------------------------N

NNCAATATTGCCCTAGCCGTCAAGTCAAATATCATCACAAGTGGATTGAAGTATTCTCTC

GCTACAGGTAACTGGGGTGATCAAAAGAAGGCCATGAGCTCCACTGCCGGCGTTTCGCAG

GTGTTAAACAGATACACATTCGCATCTACCTTGTCACATTTGCGACGAACGAATACTCCG

GTTGGCAGAGATGGCAAGCTTGCCAAGCCACGGCAGCTTCACAACACCCACTGGGGCCTC

GTCTGCCCAGCCGAGACGCCTGAAGGTCAGGCTTGTGGTCTAGTCAAAAACCTGTCCCTT

ATGTGCTCCATCAGCGTGGGTACGTCGACGGAACCTATTATAGAATATATGATTTCGCGA

AATATGGAGGTTCTGGAAGAGTATGAACCTCAAAGGTACCCACACGCTACGAAGATCTTT

CTCAATGGATCATGGATCGGTATCCACCAAGATCCAAAGGCGCTCGTCAGAGATGTTCAA

CAGCTGCGCCGGACGAATCAGATTCCGGCTGAAGTATCATTGATTCGGGAGATTCGGGAT

CGCGAATTCAAAATCTTCTCAGATGCTGGCCGTGTCATGCGGCCCTTGTTTGTGGTCGAG

CAAGAAGATGATCCTGACAACAATATCGAGAAGGGCACACTAGTCTTGAAAAAGGACATG

GTCCGG------------CGGCTTGAGATTGACCAAACCCTCCCACCCGGAAGT------

---GATGAA---TATTTCGGATGGCAAGGCTTGGTCAATGCCGGTGTCATTGAATATATG

GATGCCGAGGAAGAAGAAACTGCCATGATTTGCATGACTCCCGAAGACTTGGAGGCTTTC

AGACTGACCAAGTTGGGCCTGATGGAGCCTGACACAGAA------TCAAAC---------

------ATGAACGAGCCTAATAAGCGACTGAAGACAAAGATGAATCCGACAACCCATACG

TACACCCATTGTGAAATTCACCCTAGTATGCTTCTTGGTATTT-----------------

------------------------------------------------------------

-----------------------------------

>Xylaria_digitata_HAST_919

GGCAAAAAGCGACTGGACCTTGCCGGGCCTTTGCTCGCCAAACTCTTCCGCAACATAGCT

CGTCGGATGACGCAGGAGGTCTTGTCGCATCTCAAGCGAAGTATCGAGCAAGGCAAGCAG

TTCAATATTGCCCTAGCCGTCAAATCAAATATTATCACGAGCGGATTGAAATATTCTCTC

GCTACAGGCAACTGGGGTGATCAGAAGAAGGCCATGAGCTCTACTGCTGGCGTTTCGCAG

GTGTTGAACAGATACACATTCGCATCTACGCTTTCACATTTACGAAGAACAAATACTCCA

GTTGGCAGAGATGGTAAGCTTGCCAAACCACGGCAGCTTCACAACACCCACTGGGGTCTG

GTCTGCCCGGCCGAGACGCCTGAGGGTCAGGCTTGTGGTTTGGTCAAAAACCTGTCACTT

ATGTGCTCCATCAGCGTGGGTACGTCGACGGAACCTATTATAGAATATATGATTTCGCGA

AATATGGAGGTTCTGGAAGAGTATGAACCTCTAAGGTACCCACATGCTACGAAGATCTTC

CTCAACGGATCGTGGATCGGTATCCACCAAGATCCAAAAGCTCTCGTCAGGGATGTTCAA

CAATTGCGCCGAACGAATCAGATTCCGGCTGAAGTATCCCTGATTCGGGATATTCGTGAT

CGCGAATTTAAGATCTTTTCAGATGCTGGTCGTGTCATGCGGCCCTTGTTCGTGGTTGAG

CAAGAAGATGATCCTGACAATGGTATTGAGAAGGGCACACTAGTCTTGAAAAAAGACATG

GTCCGG------------CGGCTTGAGATTGACCAAACCCTCCCACCTGGAAGC------

---GATGAA---TATTTCGGATGGCAAGGCTTAGTCAATGCCGGTGTCATCGAATATATG

GATGCCGAGGAAGAAGAAACTGCCATGATTTGCATGACTCCCGAAGACTTGGAAGCTTTC

AGAATGACCAAGTTGGGCCAGTTGGATCCTGACGCAGAA------TCAAAC---------

------CTGAACGAGCCGAATAAGCGACTAAAGACAAAGATGAACCCGACTACCCACACG

TACACCCATTGTGAAATTCACCCAAGTATGCTTCTTGGTATTTGTGCCAGCATCATTCCC

TTCCCCGATCATAACCAGGTAAGTGCTATGCAGTTGATTGCATCGCGGTCGTTAATCCCA

GTTATAGTCACCCCGAAACACCTACCAGTCCGCTA

>Leptomassaria_simplex_CBS_147282

NNNNNNNNNNNNNTGGACCTTGCTGGACCTTTGCTCGCCAAACTCTTCCGTAACATAGCT

CGTCGGATGACACAGGAGGTATTGTCGCATCTCAAGCGAAGTATCGAGCAAGGCAAGCAG

TTCAATATTGCCCTAGCCGTCAAATCCAATATTATCACGAGCGGGTTGAAATATTCTCTT

GCTACAGGTAACTGGGGTGATCAGAAGAAGGCCATGAGCTCTACTGCTGGCGTTTCGCAG

GTGTTGAACAGATACACTTTCGCATCTACGCTTTCCCATTTGCGAAGAACAAATACTCCG

GTTGGCAGAGATGGTAAGCTTGCTAAACCACGGCAGCTTCACAACACCCACTGGGGCCTG

GTGTGCCCGGCCGAGACACCTGAAGGTCAGGCGTGTGGTTTGGTGAAAAACTTGTCACTT

ATGTGCTCTATCAGCGTGGGTACGTCGACGGAACCTATTATAGAATATATGATTTCGCGA

AATATGGAGGTTCTGGAAGAGTATGAACCTCAAAGGTACCCACATGCTACGAAGATCTTT

CTCAACGGATCATGGATCGGTATCCACCAAGATCCAAAAGCTCTTGTCAGGGATGTTCAA

CAACTGCGTAGGACGAATCAGATTCCGGCTGAAGTATCTCTGATTCGGGATATTCGGGAT

CGTGAATTCAAGATCTTCTCAGATGCTGGCCGTGTCATGCGGCCCTTGTTTGTGGTCGAG

CAAGATGATGATCCTGACAACCATATTGAAAAGGGCACACTGGTCTTGAAAAAGGACATG

GTCCGG------------CGGCTTGAAATCGACCAGACCCTTCCACCCGGAAGC------

---GATGAA---TATTTCGGATGGGACGGCTTGGTCAATGCCGGTGTCATCGAATACATG

GATGCCGAGGAAGAAGAAACCGCCATGATTTGCATGACCCCCGAGGATTTGGAGGCTTTC

AGACTGACCAAGTTGGGTCAATTGGATCCCGACGCAGAA------TCAAAT---------

------TTGAACGAGCCGAATAAGCGACTGAAGACAAAGATGAATCCGACGACCCATACG

TACACTCATTGTGAGATTCACCCAAGTATGCTTCTTGGTATCTGTGCCAGCATCATTCCC

TTCCCCGATCATAATCAGGTAAGTTCCCTGCAGTCGATCGCATCACCGTCGTTAATCACA

GTTATAGTCACCCGAAAACAAATACCAGTCCGCCN

>Stilbohypoxylon_elaeicola_Y.M.J._173

GGCAAGAAGCGACTGGATCTCGCTGGACCTTTGCTTGCCAAACTCTTCCGCAATATAGTG

CGTCGGATGACCCAGGAAGTATTGTCCCATCTTAAGCGAAGCATCGAACAAGGCAAACAG

TTCAACATTGCGCTGGCCGTTAAGTCCAACATCATAACGAGCGGACTGAAGTATTCTCTC

GCTACGGGCAACTGGGGTGATCAGAAGAAGGCTATGAGCTCCACTGCTGGTGTTTCGCAA

GTGCTGAATCGTTACACGTTTGCCTCTACTCTGTCACATTTACGAAGAACAAACACTCCA

GTAGGCCGAGATGGCAAGCTGGCCAAGCCACGGCAGCTTCACAATACGCACTGGGGTCTT

GTCTGTCCAGCCGAGACACCAGAAGGTCAAGCTTGTGGCCTAGTCAAGAACCTGTCTCTG

ATGTGCTCTGTGAGCGTGGGTACCTCGACGGAACCGATTATAGAATATATGATTTCTCGA

AACATGGAGGTGTTAGAAGAGTATGACCCTCCAAGGTACCCACATGCCACAAAGATCTTC

CTCAACGGATCATGGATTGGTGTCCATCAAGATCCTAAGGCTCTCGTAAGAGACGTCCAA

CAGCTACGTCGGACGAACCAAATCCCTGCTGAAGTCTCCTTGATACGGGATATTCGTGAT

CGCGAATTCAAGATATTCTCAGACGCTGGCCGTGTCATGCGACCATTGTTTGTGGTCGAG

CAAGATGACGATCCCGAGAATGGTGTTGAGAAAGGCACATTGGTCCTGACGAAAGACTTG

GTCCGG------------AGGCTGGAGATCGACCAGACGCTAGCACCCGGAAGT------

---GAAGAG---TACTTCGGATGGCAGGGCCTAGTCAATGCGGGCGTTATCGAATATATG

GATGCCGAGGAAGAAGAAACCGCCATGATATGCATGACACCCGAGGACTTGGAAGCTTAT

CGAATGGCAAAACTGGGCTACCCTAACCCTGACGCCGAC------AGCAAC---------

------CTCAACGAACCCAATAAACGACTCAAGACAAAGATTAATCCGACCACCCACACG

TACACCCATTGCGAAATCCACCCGAGTATGCTTCTTGGCATTTGTGCCAGCATCATCCCC

TTCCCTGACCACAACCAGGTAGGTACCACACGTTTCATTTTCCAGCAGTTATTAACTCCA

AT-CTAGTCACCGCGAAACACATACCAATCTGCTA

>Xylaria_ianthinovelutina_HAST_553

GGCAAGAAGCGACTGGATCTTGCTGGACCTTTGCTTGCCAAACTCTTCCGCAATATAGTG

CGTCGGATGACTCAGGAAGTATTGTCCCATCTCAAGCGGAGCATCGAACAAGGCAAACAG

TTCAACATCGCGCTGGCCGTTAAGTCGAACATCATTACGAGCGGACTGAAGTATTCTCTC

GCTACAGGCAACTGGGGTGATCAGAAGAAGGCCATGAGCTCCACTGCTGGTGTTTCGCAA

GTGCTGAATCGTTACACATTTGCCTCTACTCTGTCGCATTTGCGAAGAACAAACACTCCA

GTAGGCCGAGATGGCAAACTGGCCAAGCCACGGCAGCTTCACAATACACACTGGGGTCTC

GTCTGTCCAGCTGAGACTCCAGAAGGCCAAGCTTGTGGTCTAGTCAAGAACCTGTCTCTG

ATGTGCTCTGTGAGTGTGGGTACCTCGACGGAACCTATTATAGAATATATGATTTCTCGA

AATATGGAGGTGTTAGAAGAGTACGACCCTCCCAGGTACCCACATGCCACGAAGATCTTC

CTCAACGGATCATGGATTGGTGTCCATCAAGATCCAAAGGCCCTCGTGAGAGACGTTCAA

CAACTACGCCGGACAAACCAGATTCCGGCTGAAGTATCCTTGATTCGGGATATTCGTGAC

CGCGAATTCAAGATATTTTCAGACGCTGGCCGTGTCATGCGACCACTGTTTGTGGTTGAG

CAAGATGACGATCCCGAGAACGGTGTCGAGAAAGGCACATTGGTCCTGACGAAGGACTTG

GTCCGA------------AGGCTCGAGATCGATCAGACTCTGGCACCGGGGAGT------

---GAAGAG---TATTTCGGATGGCAGGGCCTCGTCAATGCCGGTGTTATCGAATACATG

GATGCTGAGGAAGAAGAAACTGCAATGATATGCATGACACCCGAGGACTTGGAAGCTTAC

CGAATGGCAAAACTGGGTTACCCTAATCCTGACGCTGAC------AGCAAC---------

------CTCAACGAACCCAACAAGAGACTCAAGACAAAAATCAATCCGACCACCCACACG

TACACTCATTGCGAAATCCACCCGAGTATGCTTCTTGGCATTTGTGCTAGCATCATCCCC

TTCCCTGACCACAACCAAGTAAGTGTCATACGTTTCATTTTCTCACAGTTGTTAACCCCA

GT-TTAGTCACCACGAAACACCTACCAATCTGCCA

>Xylaria_polymorpha_MUCL_49884

------------------------------------------------------------

--------------GGAGGTATTGTCGCATCTCAAGCGAAGTATCGAGCAAGGCAAGCAG

TTCAATATTGCCCTAGCCGTCAAGTCAAACATCATTACGAGCGGATTGAAGTATTCTCTC

GCTACAGGCAACTGGGGTGATCAGAAGAAGGCGATGAGCTCTACTGCTGGCGTTTCACAG

GTGTTGAACAGATACACATTCGCTTCTACCTTGTCGCATTTACGAAGAACAAATACGCCA

GTTGGCAGAGATGGTAAGCTTGCCAAGCCACGGCAGCTTCACAACACGCACTGGGGTTTA

GTCTGCCCAGCCGAAACGCCTGAGGGCCAGGCTTGTGGTTTAGTCAAAAACCTGTCCCTT

ATGTGCTCGATCAGTGTGGGTACGTCGACGGAACCAATTATAGAATATATGATTTCGCGA

AATATGGAGGTTCTGGAAGAGTATGAACCTCAAAGGTACCCACACGCGACGAAGATCTTT

CTCAACGGATCATGGATCGGTATTCACCAAGATCCTAAAGCTCTCGTCAGGGATGTGCAA

CAGCTACGCCGGACGAATCAGATCCCAGCTGAAGTGTCCCTAATTCGAGAAATTCGTGAT

CGCGAATTTAAGATCTTCTCAGATGCTGGCCGTGTCATGCGACCATTGTTCGTGGTCGAG

CAAGAAGATGGTGCTGACAACGGTGTCGAGAAGGGCTCCCTCGTCTTGACAAAAGATATG

GTCCGG------------CGACTGGAGATTGACCAAACCCTGCCGCCCGGAAGC------

---GATGAA---TATTTCGGATGGCAAGGTTTGGTCAACGCCGGTGTCATCGAATATATG

GATGCCGAGGAAGAAGAAACTGCCATGATTTGCATGACTCCTGAAGACTTGGAGGCTTTC

AGGCTCACTAAGTTAGGCATACCGGACCCTGACGCAGAA------TCCAAC---------

------CTCGACCAGCCAAATAAGCGACTAAAGACAAAACTGAACCCGACGACCCATACG

TACACGCATTGTGAAATTCACCCAAGCATGCTTCTTGGCATTTGTGCCAGCATTATCCCC

TTCCCCGATCATAACCAGGTAAGTGACA--------------------------------

-----------------------------------

>Xylaria_atrosphaerica_HAST_91111214

GGCAAAAAGCGACTTGACCTCGCTGGACCCTTGCTCGCCAAACTCTTTCGCAACATAGCA

CGACGAATGACGCAGGAAGTATTATCGCACCTCAAGCGAAGTATCGAGCAAGGCAAGCAG

TTCAATATTGCCCTAGCCGTCAAGTCAAATATCATCACAAGCGGATTGAAGTATTCTCTC

GCTACAGGCAACTGGGGTGATCAGAAGAAGGCGATGAGCTCTACTGCTGGCGTTTCACAG

GTGCTGAACAGATACACATTCGCATCTACCTTGTCACATTTACGAAGAACGAATACGCCA

GTTGGCAGAGATGGTAAGCTGGCCAAGCCTCGGCAGCTTCACAACACGCACTGGGGTTTG

GTCTGCCCAGCCGAAACGCCTGAGGGCCAGGCTTGTGGTTTAGTCAAAAATCTGTCCCTT

ATGTGCTCTATCAGCGTGGGTACGTCGACGGAACCAATTATAGAATATATGATTTCGCGA

AATATGGAGGTTCTGGAAGAGTATGAACCTCAAAGGTATCCAAATGCGACCAAGATATTT

CTCAACGGATCATGGATCGGCATCCACCAAGATCCTAAAGCGCTCGTGAGGGATGTGCAA

CAACTACGCCGGACGAACCAGATTCCGGCTGAAGTGTCCCTGATCCGAGAAATTCGCGAT

CGCGAATTTAAGATCTTCTCAGATGCTGGCCGTGTCATGCGACCATTGTTCGTGGTTGAG

CAAGAAGATGGTGCTGACAACGGTGTCGAGAAAGGCTCCCTCGTCTTGACAAAAGACATG

GTCCGG------------CGACTGGAGCTTGACTCAACCCTGCCACCCGGAAGC------

---GATGAA---TATTTCGGATGGCAAGGTTTGGTCAACGCCGGTGTCATCGAGTATATG

GATGCCGAGGAAGAAGAAACTGCCATGATTTGCATGACTCCTGAAGACTTAGAGGCTTTC

AGGCTCACTAAGTTAGGCATACCGGACCCCGACGCGGAA------TCCAAC---------

------CTCGACCAGCCTAATAGGCGACTAAAGACAAAGCTAAACCCGACGACCCATACG

TACACGCATTGTGAAATTCACCCGAGTATGCTTCTTGGCATTTGTGCCAGCATCATCCCC

TTTCCCGATCATAACCAGGTAAGTGACATGAAGTTGATTGCATCGCGGTCATTAACCACA

GTTATAGTCACCTCGAAATACGTATCAGTCTGCCA

>Amphirosellinia_nigrospora_HAST_91092308

GGCAAGAAGCGACTAGACCTTGCAGGTCCCTTGCTCGCCAAACTCTTCCGCAACATAGCG

CGTCGGATGACTCAGGAGGTTCTATCGCATCTTAAGCGAAGTATCGAGCAAGGCAAGCAG

TTCAACATTGCCCTCGCCGTCAAGTCCAATATCATCACGAGCGGATTGAAATACTCTCTC

GCGACCGGCAACTGGGGTGATCAGAAGAAGGCCATGAGCTCTACAGCCGGTGTGTCACAG

GTATTGAACAGATACACATTCGCATCGACTTTGTCACATTTGCGAAGAACGAATACGCCA

GTTGGGCGAGATGGCAAGCTTGCCAAGCCACGGCAACTTCACAACACGCACTGGGGTCTC

GTCTGCCCGGCTGAGACACCCGAAGGTCAGGCTTGTGGTCTAGTCAAGAACCTGTCCCTT

ATGTGCTCTATCAGCGTGGGTACGTCGACGGAACCTATTATAGAATATATGATTTCGCGT

AATATGGAGGTTCTGGAAGAGTATGAACCTCAAAGGTACCCACATGCTACCAAGATCTTC

CTCAACGGATCGTGGATCGGTATTCACCAAGATCCAAAATCTCTCGTCAGGGATGTTCAG

CAACTCCGTAGGACTAATCAGATACCGGCTGAAGTCTCCTTGATTCGGGACATTCGCGAT

CGCGAGTTTAAAATCTTCTCAGATGCCGGCCGTGTGATGCGGCCTTTGTTCGTGGTCGAG

CAAGAAGATGATGCCGACAACGACACTATTAAGGGCACACTGGTCTTGAAAAAGGACATG

GTCCGG------------CGGCTGGAGATTGACCAAACGCTCCCACCGGGAAGC------

---GATGAA---TATTTCGGATGGCAAGGCCTAGTTAATGCCGGCGTCATCGAATATATG

GACGCCGAGGAGGAAGAGACTGCTATGATTTGCATGACCCCGGAAGATTTAGAGGCTTTT

AGGATGGCCAAGATGGGCATTCCGGATCCTGATGCAGAA------TCAAGC---------

------CTGACCGCGCCGAATAAGCGACTCAAGACAAAGATGAATCCGACGACCCATACG

TACACCCATTGTGAAATTCACCCAAGTATGCTTCTTGGCATTTGTGCCAGCATCATCCCC

TTCCCCGATCATAACCAGGTATGTGCGATGCGGTTGATTATGTGACAGATGCTGACCCCG

ACTTTAGTCACCCCGAAACACCTACCAATCCGCCA

>Dematophora_necatrix_CBS_349.36

-----------------------------------------GCTCTTCCGCAACATAGTG

CGTCGGATGACTCAGGAGGTTCTATCGCACTTAAAGCGAAGTATCGAGCAAGGCAAGCAG

TTTAATATTGCCCTGGCCGTCAAGTCCAACATCATCACGAGCGGACTGAAGTATTCTCTT

GCCACGGGTAACTGGGGTGATCAGAAAAAGGCGATGAGCTCTACCGCCGGTGTGTCGCAG

GTTTTAAACCGATACACATTCGCATCCACCCTATCTCATTTGCGGAGAACCAATACGCCA

GTTGGTAGAGACGGCAAACTCGCCAAGCCGCGCCAGCTTCACAACACCCACTGGGGTCTC

GTCTGCCCGGCCGAGACACCCGAAGGCCAGGCTTGCGGCCTTGTCAAGAATCTATCTCTG

ATGTGCTCCGTTAGCGTGGGTACTTCGACGGAGCCGATTATCGAGTATATGATTTCTCGA

AATATGGAGGTTCTAGAGGAGTACGAACCCCAACGGTACCCTAATGCGACTAAGATCTTC

CTGAACGGATCATGGATTGGCGTCCACCAAGACCCGAAGGCGCTCGTGAGGGATGTCCAA

CAATTACGCCGGACGAACCAGATCCCGGCCGAGGTATCATTGATTCGGGAAATTCGTGAT

CGCGAATTCAAAATTTTCTCAGATGCCGGTCGTGTGATGCGGCCTCTATTCGTGGTAGAG

CAGGAGGATGATACCGATCGCGGTATTACGAAAGGCACGCTGGTCTTGACGAAAGACATG

GTTCGG------------AGACTTGAGATTGATCAGTCCCTACCACCGGGAAGC------

---GATGAG---TACTTTGGATGGCAGGGTTTGGTCAATTCTGGTGTCATCGAATACATG

GATGCCGAGGAAGAAGAGACGGCCATGATTTCCATGACTCCCGAGGATCTCGAGGCACAC

AGACTGGCCAGACTAGGTATCCCGAATTCTGACGCGGAC------TACAAT---------

------ATGAACAATCCGAACAGACGACTGAAGACCAAGATAAACCCAACGACTCACACA

TACACCCACTGCGAAATTCATCCCAGTATGCTTCTTGGCATCTGCGCCAGTATCATTCCC

TTCCCTGATCATANCCAAGTGAGCGCGACGTTTCTCTTTTCGTCACAG------------

-----------------------------------

>Xylaria_oxyacanthae_859_JDR

GGCAAAAAACGACTAGACCTGGCAGGGCCTTTACTCGCCAAGCTCTTCCGCAACATAGTA

CGTCGGATGACTCAAGAGGTCCTCTCACATCTCAAACGCAGCATCGAACAAGGTAAACAG

TTCAACATTGCCCTAGCCGTCAAGTCAAACATTATCACGAGCGGATTGAAATATTCCCTC

GCCACTGGCAATTGGGGTGACCAGAAGAAGGCCATGAGCTCCACGGCCGGCGTTTCGCAG

GTGCTAAACCGATACACATTTGCATCTACTTTGTCACATTTGAGAAGAACGAATACCCCC

GTTGGCAGAGATGGCAAGCTTGCCAAGCCACGGCAGCTTCACAACACTCACTGGGGTCTC

GTCTGCCCGGCTGAAACCCCCGAAGGTCAGGCTTGCGGTCTAGTCAAGAACCTGTCCCTA

ATGTGCTCCATCAGCGTTGGCACCTCCACAGAACCGATTATCGACTATATGACGTTTCGA

AATATGGAGGTTCTGGAAGAGTATGAGCCTCTGAGGTACCCTCATGCCACGAAGATCTTC

CTTAATGGATCATGGGTCGGTGTTCATCAAGATCCCAAGGCCCTCGTGAGGGACGTTCAA

CAATTGCGCCGAACGAATCAGATACCGGCTGAGGTATCCTTGATCAGGGATATTCGTGAC

CGCGAATTCAAGATCTTCTCGGATGCAGGCCGTGTCATGAGGCCCTTGTTCGTGGTTGAG

CAAGAGGACGATCCAGAGAATGGCGTCAACAAGGGCTCATTGATCTTGACGAAAGACATG

GTCCGG------------CGGCTTGAGATTGACCAGACGCTCCCACCTGGAAGT------

---GACGAG---TATTTCGGATGGCAGGGATTGGTCAATGCAGGTGTAATTGAATATATG

GATGCCGAGGAAGAAGAGACTGCAATGATTTGCATGACCCCCGAAGACTTGGAGGCTTAT

CGCCAGTCCAGATTGGGTGTCACGCATCCTGAT------------GACAAC---------

------CTGGACAATCCAAACAAGCGTCTGAAGACAAAGATCAATCCTACAACCCACACT

TATACACACTGCGAAATCCACCCCAGCATGCTTCTTGGCATATGTGCCAGTATTATCCCA

TTTCCTGATCATAACCAAGTAAGTGACATTTCAACTATCTCGTCGCAGTGTTTAATATGA

CCTCTAGTCGCCCCGTAACACATACCAATCCGCTA

>Albicollum_vincensii_CBS_147286

NNNNNNNNNNNNNTGGATCTCGCTGGCCCTCTGCTCGCAAAACTCTTCCGCAGTATTGTG

CGTCGGATGACACAGGAAGTCTTGTCGCACTTGAAGCGAAGTATCGAGCAGGGCAAGCAA

TTCAATATTGCCTTAGCCGTCAAGTCGAATATTATCACGAGCGGTCTGAAATACTCTCTC

GCCACAGGTAACTGGGGTGATCAGAAGAAGGCCATGAGCTCTACTGCTGGTGTGTCACAG

GTGCTGAACCGATACACATTCGCCTCCACTCTGTCACATTTGCGAAGAACAAATACCCCC

GTCGGTAGAGATGGTAAGCTCGCCAAGCCCCGGCAGCTTCACAACACGCATTGGGGCCTC

GTTTGCCCTGCCGAGACGCCCGAAGGTCAGGCTTGTGGCCTGGTCAAAAATTTGTCCCTA

ATGTGCTCCATTAGCGTCGGCACGTCGACGGAGCCCATTATAGATTATATGATTTCCCGC

AACATGGAGGTTCTGGAAGAGTACGAGCCCCAGAGGTATCCTCATGCCACCAAGATCTTT

CTCAATGGATCGTGGATCGGCGTACACCAAGACCCGAAAGCTCTTGTCCGGGACGTTCAG

CAACTGCGCCGTACTAATCAGATCCCGTCCGAAGTATCCCTAATTCGGGATATTCGTGAT

CGGGAATTCAAGATCTTCTCGGACGCTGGGCGTGTCATGCGGCCTTTGTTCGTTGTCGAA

CAAGAAGAGGACGATGAGAATAATAAACACAAGGGATCACTAGTCTTGACGAAGGACATG

GTCCGG------------AGACTTGAACTTGATCAGACTCTCCCACCTGGAAGT------

---GATGAG---TATTACGGATGGTCAGGCTTGGTGAATGCTGGTGTCATCGAATATTTG

GATGCCGAGGAAGAGGAAACTGCGATGATCTGCATGACTCCAGAGGATCTGGAGACTTAC

CGGTTGGCTAAGTTGGGTTATGAGGTCGAGCCG------------GACAAC---------

------AGCGACGACCCTAATAAGCGACTGAAGACGAAAACGAATCCCACAACCCACATG

TATACCCATTGCGAAATCCACCCCAGTATGCTACTTGGCATTTGTGCCAGTATCATTCCT

TTCCCCGATCATAACCAGGTACGTGACCTACTATCTGTCTTACCGCAGTCGCTAATCGCA

GATATAGTCTCCCGAAAACACGTACCATTCAGCTN

>Albicollum_longisporum_CBS_147283

NNNNNNNNNNNNNTGGATCTCGCTGGTCCTCTGCTCGCAAAACTCTTTCGCAGTATTGTG

CGTCGGATGACCCAGGAGGTATTGTCGCACCTGAAGCGAAGTATCGAGCAGGGCAAGCAA

TTTAATATTGCCTTGGCCGTCAAGTCAAACATTATTACGAGCGGGTTGAAATACTCTCTT

GCTACAGGTAACTGGGGCGATCAGAAGAAGGCCATGAGCTCTACCGCCGGTGTCTCGCAG

GTGCTGAACCGCTACACATTTGCATCTACCCTATCACATTTGCGAAGAACAAATACGCCC

GTTGGTAGAGATGGTAAGCTCGCCAAGCCACGGCAACTTCACAACACACATTGGGGCCTT

GTTTGCCCTGCCGAGACGCCCGAGGGTCAGGCTTGTGGCCTTGTCAAAAACTTGTCGCTC

ATGTGCTCTATTAGCGTTGGCACGTCGACGGAACCCATTATAGATTATATGATTTCCCGA

AACATGGAAGTCCTGGAGGAGTACGAGCCCCAGAGATATCCTCATGCCACCAAGATATTC

CTGAATGGATCATGGATCGGCGTACATCAAGATCCAAAAGCTCTCGTCAGGGACGTTCAG

CAACTGCGCCGTACAAATCAGATCCCCGCTGAAGTGTCTGTAATTCGCGATATTCGTGAT

CGCGAATTCAAAATCTTCTCAGATGCTGGGCGTGTTATGCGGCCTCTATTCGTTGTCGAA

CAAGAAGGCGACGATGATAACATTGATAAGAAGGGATCATTAGTCTTGACAAAAGACATG

GTCCGG------------AGACTTGAACTTGATCAGACCCTCCCACCTGGAAGT------

---GACGAG---TATTTTGGATGGCCAGGCTTGGTGAATGCTGGTGTTATCGAATATTTG

GATGCCGAGGAAGAAGAAACTGCAATGATTTGCATGACTCCCGAAGATCTGGAAACATAT

CGATTGGCCAAGTTGGGTTACCAGGTCGAGGCC------------GACAAC---------

------GGCGATGACCCAAACAAGCGACTGAAGACAAAAACGAATCCTACAACTCACATG

TATACCCATTGCGAAATCCACCCGAGTATGCTACTTGGCATTTGTGCCAGTATCATCCCT

TTCCCCGATCACAATCAGGTAAGTGGTGTACTTCCTGTTATAATGTAGTTACTAATCGAG

ACTATAGTCTCCCGAAAATACGTACCAGTCAGCTN

>Stromatoneurospora_phoenix_BCC_82040

------------------------------------------------------------

------------------------------------------------------------

------------------------------------------------------------

------------------------------------------------------------

------------------------------------------------------------

------------------------------------------------------------

---------------------------CAGGCTTGTGGTCTGGTCAAAAATCTATCACTG

ATGTGCTCTATAAGCGTGGGAACTTCCACAGAACCTATCATAGAATATATGATTTCGCGT

AACATGGAAGTACTTGAGGAGTATGAACCCCAGCGGTATCCCCACGCCACCAAGATCTTC

CTCAACGGGTCCTGGATCGGCGTCCACCAAGACGCAAGGGCCCTCGTCCGAGACGTCCAG

CAACTACGCCGAGCGAACCAGATTCCCGCCGAGGTGTCCTTGATTCGAGATATTCGTGAT

CGCGAGTTCAAGATCTTCTCAGATGCTGGGCGTGTCATGCGGCCCCTGTTCGTTGTCGAA

CAGGAGGACGATTTGGAGAACAACATCCAAAAGGGGTCGCTAGCCTTGACTAAAGATATG

GTGCGG------------AGACTGGAGATCGATGCGACCCTCCCACCTGGGAGC------

---GACGAG---TACTTTGGATGGCAAGGCTTAGTCAATGCTGGTGTTATCGAATATCTG

GATGCCGAAGAGGAGGAGACTGCCATGATCTGCATGACTCCCGAAGATCTGGAAAGCTAT

CGTCAGGCTAAAATGGGCACTCTTGAGCAGGAA------------GATACC---------

------AGCGAGTCGCTGAACAAGCGGCTGAAGACGAAGATGAACCCGTCAACACACATG

TACACCCATTGCGAAATTCACCCGAGCATGCTTCTGGGCATCTGTGCCAGTATCATCCCC

TTC---------------------------------------------------------

-----------------------------------

>Sarcoxylon_compunctum_CBS_359.61

------------------------------------------------------------

------------------------------------------------------------

------------------------------------------------------------

------------------------------------------------------------

-----TGATCGATACACATTTGCATCCACATTATCCCATTTGCGGAGAACGAACACCCCC

GTCGGCAGAGATGGCAAACTGGCTAAGCCGCGTCAGCTTCACAACACACATTGGGGCCTC

GTCTGCCCAGCCGAAACGCCAGAAGGTCAAGCTTGTGGTCTAGTCAAGAACCTGTCGCTC

ATGTGCTCAATCAGTGTCGGAACATCCACGGAACCTATTATAGAATATATGATTTCGCGA

AATATGGAGGTTCTTGAGGAGTATGAGCCTGCACGATACCCTCACGCCACTAAGATCTTC

TTGAATGGGTCCTGGATCGGCGTTCATCAGGATGCAAGGGCTCTTGTCAGAGACGTCCAG

CAGCTACGACGAGCAAACCAGATTCCAGCTGAGGTCTCCTTGATCCGGGATATCCGTGAC

CGGGAATTCAAGATCTTCTCGGATGCTGGTCGTGTTATGCGCCCCTTGTTTGTAGTCGAG

CAGGAGGACGATGCTGAAAACGGCGTTCAGAAAGGTACGCTGGCCTTGAGCAAAGACATG

GTTCGC------------AGGCTCGAGCTCGATCAGACCCTGCCACCAGACAGT------

---GACGAG---TATTTTGGATGGCAGGGTCTAGTAAATGCCGGTGTGATCGAGTACCTG

GATGCCGAGGAAGAGGAGACGGCAATGATCTGCATGACTCCCGAAGACCTGGAAATCTAT

CGGCTGGCTAAGATGGGTCATGTTGAAGATCAA------------GCCAAG---------

------AGTGACGAACCCAACAGACGACTGAAGACCAAGACGAATCCTACCACACACATG

TATACTCACTGCGAAATCCACCCAAGCATGCTTCTGGGCATCTGTGCCAGTATCATCCCC

NTCCCTGATCATAATCAGGTAAGCAGAACCCCTTTTTTGTTGACCTGGCTGCTAACCCCA

G----------------------------------

>Podosordaria_mexicana_WSP176

GGCAAGAAGCGACTGGATCTTGCCGGACCTCTGTTGGCGAAGCTGTTCCGAAATATCGTA

CGCCGCATGACGCAAGAGGTTCTGTCGCACCTGAAGCGAAGTATCGAGCAGGGAAAGCAG

TTCAACATTGCTTTGGCTGTCAAGTCCAACATTATTACGAGCGGGCTGAAGTACTCTCTC

GCCACGGGCAATTGGGGTGATCAGAAGAAGGCCATGAGCTCCACCGCTGGTGTTTCGCAA

GTCTTGAACCGGTACACGTTCGCATCGACGCTGTCCCATTTGCGCAGAACGAACACGCCC

GTTGGCAGAGACGGTAAATTGGCCAAGCCTCGTCAGCTTCACAACACGCATTGGGGTCTC

GTCTGCCCGGCCGAGACGCCCGAAGGCCAAGCGTGTGGTCTAGTCAAGAACCTGTCGCTT

ATGTGCTCAGTAAGCGTCGGAACCTCGACCGAACCGATTATAGAATACATGATTTCGCGA

AACATGGAAGTTCTCGAGGAGTATGAGCCTGCCCGTTACCCCCACGCAACGAAGATCTTC

CTGAATGGGTCTTGGATCGGTGTCCATCAGGACGCAAGGGCCCTTGTCAGAGATGTCCAG

CAACTACGGCGAGCGAACCAGATCCCAGCTGAGGTGTCCTTGATCAGGGATATCCGTGAT

CGGGAATTCAAAATATTTTCAGATGCCGGTCGTGTCATGCGGCCCGTCTTCGTGGTTGAG

CAGGAGGATGACCCCGACAACGGCGTCCATAAAAACACGTTGGCGCTGACCAAGGACATG

GTTCGC------------AGACTCGAGATCGATCAGACCCTAGCGCCCGGCAGT------

---GCGGAG---TATTTCGGGTGGCAAGGTCTGGTGAATTCCGGGGCGATCGAGTACCTC

GATGCCGAGGAAGAGGAGACCGCCATGATCTGCATGACGCCTGAAGACCTCGAGACTTTT

CGGATCATGAAACTGGGCGGTCCCGAAGAACCA------------CCCAAC---------

---CCCAATGACGAGCCCAACAAGCGGCTGAAGACTAAAATCAACCCGACGACACACAGT

TATACCCATTGCGAGATTCATCCGAGCATGCTGCTCGGGATATGCGCTAGCATCATTCCC

TTCCCCGATCATAACCAGGTAAGTAGAAGCGTTTTGCTGCTGCTCCGTCTGCTAACAACC

GCCTTAGTCGCCCCGAAATACCTATCAGTCGGCTA

>Poronia_punctata_CBS_656.78

-----------------------------------NGGNNNNNTTTTCCGCAACATTGTC

CGGCGGATGACGCAAGAGGTACTATCCCATTTGAAGCGAAGTATCGAGCAGGGGAAGCAG

TTCAACATCGCCTTGGCCGTCAAGTCGAATATCATCACGAGCGGACTGAAATATTCTCTT

GCGACAGGTAACTGGGGCGATCAGAAAAAGGCTATGAGTTCCACCGCCGGCGTTTCGCAG

GTGCTCAACCGATATACCTTTGCGTCCACCCTATCACATTTGCGACGGACGAACACCCCG

GTGGGTCGGGATGGCAAACTGGCCAAGCCCCGGCAGCTCCATAACACTCACTGGGGGCTC

GTCTGTCCGGCAGAGACGCCAGAAGGCCAGGCTTGCGGTCTAGTCAAGAATCTTTCTTTG

ATGTGCTCCATCAGTGTGGGGACTTCCACGGAGCCGATCATCGAGTACATGATTTCGCGG

AACATGGAAGTTCTCGAGGAATATGAACCACAGAGGTATCCACACGCAACCAAGATCTTC

CTGAACGGTTCGTGGATCGGCGTTCACCAAGATGCGAGAGCGCTCGTGAGAGACGTTCAA

CAACTTCGCCGCGCAAACCAGATTCCCGCCGAGGTGTCCTTGATTCGAGACATCCGTGAC

CGCGAGTTCAAGATCTTCTCAGATGCCGGGCGCGTCATGCGTCCCCTATTTGTGGTCGAG

CAAGAAGATGACCCCGAGAACCACATCCAGAAGGGCTCCCTTGCGTTGACCAAAGATATG

GTCCGG------------ANGCTCGAAATCGACCAGACGCTACCGCCTGGGAGC------

---GATGAG---TATTTCGGATGGCAGGGTTTGGTGAATGCNGGNGTAA-----------

------------------------------------------------------------

------------------------------------------------------------

------------------------------------------------------------

------------------------------------------------------------

------------------------------------------------------------

-----------------------------------

>Entalbostroma_erumpens_ICMP_21152

------------------------------------GCTAAGCTCTTCCGCAACATTGTT

CGTCGGATGACTCAGGAGGTCCTATCCCACCTGAAGCGAAGTATCGAGCAAGGCAAGCAG

TTCAACATCGCGTTGGCCGTCAAATCGAATATCATCACCAGTGGTCTCAAATACTCCCTC

GCCACCGGAAACTGGGGAGACCAGAAGAAGGCGATGAGTTCTACTGCCGGTGTCTCGCAG

GTGCTGAATCGATACACTTTCGCGTCGACTCTGTCGCATTTGCGAAGAACAAACACTCCT

GTTGGTCGAGACGGCAAGCTTGCTAAACCACGGCAACTTCACAACACCCATTGGGGTCTC

GTCTGCCCTGCCGAGACCCCCGAGGGCCAGGCCTGTGGTCTAGTCAAGAACCTGTCCCTC

ATGTGCTCCATAAGCGTCGGTACTTCGACAGAACCAATTATTGACTACATGATTCTACGG

AACATGGAAGTTTTGGAAGAGTATGACCATCACAGGTATCCCAACGCGACAAAGGTGTTC

CTGAACGGTGCATGGATAGGCGTTCATCAGGATCCCAAGGCTCTGGTGAAGGACGTACAA

GAGCTGCGCCGAACGAATCAGATTCCAGCGGAGGTCTCCCTCATCAGAGATATCCGCGAC

CGCGAGTTCAAAATCTTCTCCGATGCAGGCCGTGTCATGCGGCCCCTGTATGTGGTCAAT

CAGGAGGATGATGCTGACAAGGGCGTTGAGAAATGTACACTAGTTTTGACCAAAGACATG

GTCAGG------------AGACTCGAGATTGACCAGACACTCCCACCTGGGAGT------

---GATGAA---TACTTTGGTTGGCAAGGCTTGGTGAATGCCGGTGTCATTGAATACATG

GACGCTGAGGAAGAGGAAACTGCCATGATATGCATGACCCCCGAAGACCTGGAAAGCTTC

CGGTGTACCAAGCTGGGTTTAGCAGATCCCTTCAACCGG------GACGAC---------

------GTCTTTGCGCCGAATAAGCGGCTAAAGACGAGAATTAACCCCACGACTCACTCG

TACACCCATTGCGAAATCCATCCCAGCATGCTTTTGGGCATTTGCGCCAGTATCATCCCC

TTCCCCGATCACAATCAGGTAAGGACCATGTAATCCGCTCTGTGGCAATCACTGATCCTC

AGTT-------------------------------

>Induratia_apiospora_ATCC_60639

GGCAAGAAACGTTTGGATCTCGCGGGTCCTCTCCTCGCCAAGCTCTTCCGCAATATCATT

CGCCGGATGACCCAAGAGACCTTGTCGCACTTGAAGCGTAGCATTGAGCAGACTAAAAAT

TTTGATATATGGTTAGCTGTAAAGGCGCCCATCGTTACCAACGGTCTGAAGTACTCTCTA

GCTACAGGCAATTGGGGCGACCAGAAGAAAGCCATGAGTTCCACGGCCGGTGTTTCGCAG

GTGCTGAACCGGTACACGTTCGCCTCCACCCTCTCTCATTTGAGAAGGACGAACACTCCC

GTCGGAAGAGACGGCAAGCTTGCCAAACCACGACAGCTTCACAACACGCATTGGGGTCTG

GTCTGTCCGGCTGAGACGCCTGAAGGTCAAGCTTGTGGCCTGGTGAAGAATCTCTCTCTC

ATGTGCTCCATCAGCGTTGGTACATCAACAGAGCCTATTATTGACTACATGATCACCCGA

AATATGGAAGTCCTTGAGGAGTATGAGCCGACGAGATACCCCAACGCTACCAAGATCTTC

CTCAATGGCTCCTGGATCGGTGTTCACCAGGACCCGAAGGCACTCGTCAAAGATGTCCAG

CAATTGCGACGAACGAACCAGATCCCAGCTGAAGTGTCCTTGGTTCGCGATATTCGAGAT

CGTGAATTCAAGATTTTTTCTGATGCAGGCCGGGTGATGCGACCACTATTCGTGGTGGAA

CAGGAAGACATTCCCGAGACTAGCGTTGAGAAGGGCCAGCTGGTTCTTACCAAGGACATG

GTATCA------------AGGCTCGAGATAGACCAGACTCTTCCCCCGGGAAGC------

---GAAGAA---TACTTTGGGTGGCAGGGTCTGGTGAATGCAGGCGTCGTCGAATACCTC

GACGCCGAGGAGGAAGAAACCGCGATGATATGCATGACGCCGGAAGACCTAGAGATCTAT

CGGAGGCAGAAGGTCGGCAATTACGAAGCTGAG------------GACAAC---------

------TCAAATGACCTGAACAAGCGGCTCAAAACCAGAGTTAACCCTACAACTCATATG

TACACACACTGCGAGATTCATCCCAGTATGCTTCTCGGCATTTGTGCGAGCATTATTCCC

TTCCCCGATCATAACCAGGTAAGGATA-T----ATAAGTTCTTTAGGGTTACTAACCATA

CTTGTAGTCCCCCCGAAA-----------------

>Barrmaelia_rhamnicola_BR

----------------------------------------AGTTATTCCGCAACATAGTC

CGACGGATGACTCAGGAGGTTTTGTCACAACTGAAGCGCAGCATCGAGCAGAACAAGCGG

TTCGACATTGCGCTGGCTGTCAAGTCCACCATCATCACCAACGGTCTGAAGTACTCTCTC

GCTACAGGCAACTGGGGCGACCAGAAGAAGGCCATGAGCTCTACCGCTGGTGTGTCGCAG

GTGCTGAACCGATACACATTTGCCTCGACTCTGTCCCATTTGAGACGGACCAACACCCCC

GTTGGGAGAGATGGCAAGCTTGCCAAACCACGGCAGCTTCACAATACGCACTGGGGACTG

GTCTGTCCGGCGGAGACGCCTGAAGGTCAAGCTTGTGGGCTGGTGAAGAATCTTTCCCTC

ATGTGCTCCATCAGCGTGGGCACATCAACGGAACCGATCATCGACTATATGATTACTCGG

AACATGGAAGTCCTAGAAGAGTACGAACCCACGCGATATCCCAATGCTACCAAGATTTTC

CTTAATGGCTCCTGGATCGGTGTTCATCAGGATCCGAAATCCCTCGTCCGGGACGTGCAG

CAGCTGCGCCGAACCAACCAGATCCCGGCTGAGGTATCTCTTGTCCGCGACATTCGAGAT

CGAGAGTTCAAAATCTTCTCTGATGCAGGGCGAGTCATGCGACCATTGTTCGTTGTCGAA

CAAGAGGGCGACGATGAAAATGGCGTTACCAAAGGCGCACTCGCGCTCACGAAGGACATG

GTGTCG------------AGACTCGAGATTGACGCCACTCTCCCACCTAGGAGC------

---GAGGAG---TACTTTGGGTGGCAAGGCCTGGTGAACGCGGGTGTCGTCGAATATCTT

GATGCTGAAGAAGAAGAGACGGCGATGATATGTATGACGCCCGAAGATCTTGAGATATAT

CGAAAGATGAGGACTGGTGAGGCGGTTGAGCAA------------GACGGT---------

------ACCGACGACCTTAACAAGCGCCTCAAAACCAAGATCAATCCAACGACGCATATG

TACACACACTGCGAAATCCATCCAAGCATGCTTCTGGGTATTTGCGCGAGCATCATCCCC

TTCCCCGATCATAACCAGGTAAGCGCACTTCCTACATTCCGTATGACCATTATC---ACA

-----------------------------------

>Barrmaelia_macrospora_CBS_142768

-------------------------GTCCTCTCCTTGCCAAGTTATTCCGCAACATAGTC

CGACGGATGACTCAGGAGGTTTTGTCACAACTGAAGCGCAGCATCGAGCAGAACAAGCGG

TTCGACATTGCGCTGGCTGTCAAGTCCACCATCATCACCAACGGTCTGAAGTACTCTCTC

GCTACAGGCAATTGGGGCGACCAGAAGAAGGCCATGAGCTCTACCGCTGGTGTGTCGCAG

GTGCTGAACCGATACACATTTGCCTCGACTCTGTCCCATTTGAGGCGGACCAACACCCCC

GTCGGGAGAGACGGCAAGCTTGCCAAACCACGGCAACTTCACAATACGCACTGGGGACTG

GTCTGTCCGGCGGAGACGCCTGAAGGTCAAGCTTGTGGGCTGGTGAAGAATCTTTCCCTC

ATGTGCTCCATCAGCGTGGGCACATCAACGGAACCGATCATCGACTATATGATTACTCGG

AACATGGAAGTCCTAGAGGAGTACGAACCCACGCGATATCCCAATGCTACCAAGATTTTC

CTTAATGGCTCCTGGATCGGTGTTCATCAGGATCCGAAATCCCTCGTCCGGGACGTGCAG

CAGCTGCGTCGAACCAACCAGATCCCGGCTGAGGTATCTCTTGTCCGCGACATTCGAGAT

CGAGAGTTCAAAATCTTCTCTGATGCAGGGCGAGTCATGCGACCATTGTTCGTTGTTGAA

CAAGAGGGCGACGATGAAAATGGCGTTACCAAAGGCGCACTCGCGCTCACGAAGGACATG

GTGTCG------------AGACTCGAGATTGACGCCACTCTCCCACCTGGGAGC------

---GAGGGG---TACTTTGGGTGGCAAGGCCTGGTGAACGCGGGTGTCGTCGAATACCTT

GATGCTGAAGAGGAAGAGACGGCGATGATATGTATGACGCCCGAAGATCTCGAAATATAT

CGAAAGATGAGGACTGGTGAGGCGGTTGAGCCA------------GAGGGT---------

------ACCGACGATCTTAACAAGCGCCTCAAAACCAAGATCAATCCGACGACGCATATG

TACACACACTGCGAAATCCATCCAAGCATGCTTCTGGGTATTTGCGCGAGCATCATCCCC

TTCCCCGATCATAACCAGGTAAGCACACTTCTTGCATTCCGTATGACCA-TATC---ACA

-----------------------------------

>Barrmaelia_rappazii_CBS_142771

-------------------------GTCCTCTCCTTGCCAAGTTATTCCGCAACATAGTC

CGACGGATGACTCAGGAGGTTTTGTCACAGCTGAAGCGCAGCATCGAGCAGAACAAGCGG

TTCGACATTGCGCTGGCTGTCAAGTCCACCATCATCACCAACGGTCTGAAGTACTCTCTC

GCTACAGGCAATTGGGGCGACCAGAAGAAGGCCATGAGCTCTACCGCTGGTGTGTCGCAG

GTGCTGAACCGATACACATTTGCCTCGACTCTATCCCATTTGAGACGGACCAACACCCCC

GTCGGGAGAGACGGCAAGCTTGCCAAACCACGGCAGCTTCACAATACGCACTGGGGGCTG

GTCTGTCCGGCGGAGACGCCTGAAGGTCAAGCTTGTGGGCTGGTGAAGAATCTTTCCCTC

ATGTGCTCCATCAGCGTGGGCACATCAACGGAACCGATCATCGACTATATGATTACTCGG

AACATGGAAGTCCTGGAAGAGTACGAACCTACGCGATATCCCAATGCTACCAAGATTTTC

CTTAATGGCTCCTGGATCGGTGTTCATCAGGATCCGAAATCCCTCGTCCGGGACGTGCAG

CAGCTGCGCCGAACCAACCAGATCCCGGCTGAGGTATCGCTTGTCCGCGACATTCGAGAT

CGAGAGTTCAAAATCTTCTCTGATGCAGGGCGAGTCATGCGACCATTGTTCGTTGTTGAA

CAAGAGGGCGACGATGAAAATGGCGTTACCAAAGGCGCACTCGCGCTCACGAAAGACATG

GTGTCG------------AGACTCGAGATTGACGCCACTCTCCCACCTGGGAGC------

---GAGGAG---TACTTTGGGTGGCAAGGCCTGGTGAACGCGGGTGTCGTCGAATACCTT

GATGCTGAAGAAGAAGAGACGGCGATGATATGTATGACGCCCGAAGATCTTGAGATATAT

CGAAAGATGAGGACTGGTGAGGCGGTTGAGCAA------------GACGGT---------

------ACCGACGATCTTAACAAGCGCCTCAAAACCAAGATCAATCCGACGACGCATATG

TACACACACTGCGAAATCCATCCAAGCATGCTTCTGGGTATTTGCGCGAGCATCATCCCC

TTCCCCGATCATAACCAGGTAAGCACACTTCCTGCATTCCGTATGACCATTATC---ACA

------GTCCCCTCGAAACACGTATCATCCGGCCT

>Barrmaelia_oxyacanthae_CBS_142770

-------------------------GTCCTCTCCTCGCCAAGTTATTCCGCAACATAGTC

CGACGGATGACTCAGGAGGTTTTGTCACAACTCAAGCGCAGCATCGAGCAGAACAAGCGG

TTCGACATTGCGCTGGCTGTCAAGTCCACCATCATCACCAACGGTCTGAAGTACTCTCTC

GCTACAGGCAATTGGGGCGACCAGAAGAAGGCCATGAGCTCTACCGCTGGTGTGTCACAG

GTGCTGAACCGATACACATTTGCCTCGACTCTATCCCATTTGAGACGGACCAACACCCCC

GTCGGGAGAGACGGCAAGCTTGCCAAACCACGGCAGCTTCACAATACGCACTGGGGACTG

GTCTGTCCGGCGGAGACGCCTGAAGGTCAAGCTTGTGGGCTGGTGAAGAATCTTTCCCTC

ATGTGCTCCATCAGCGTGGGCACATCAACGGAACCGATCATCGACTATATGATTACTCGG

AACATGGAAGTCCTAGAGGAGTACGAACCCACGCGATATCCCAATGCTACCAAGATTTTC

CTCAATGGCTCCTGGATCGGTGTTCATCAGGATCCGAAATCCCTCGTCCGGGACGTGCAG

CAGCTGCGTCGAACCAACCAGATCCCGGCTGAGGTATCTCTTGTCCGCGACATTCGAGAT

CGAGAGTTCAAAATCTTCTCTGATGCAGGGCGAGTCATGCGACCATTGTTCGTTGTTGAA

CAAGAGGGCGACGAGGAAAATGGCGTTACCAAAGGCGCACTCGCGCTCACGAAGGACATG

GTGTCG------------AGACTCGAGATTGACGCCACTCTCCCACCTGGGAGC------

---GAGGGG---TACTTTGGGTGGCAAGGCCTGGTGAACGCGGGTGTCGTCGAATATCTT

GATGCTGAAGAAGAAGAGACGGCGATGATATGTATGACACCCGAAGATCTTGAGATATAC

CGAAAGATGCGGACTGGTGAGGCGGTCGAGCAA------------GACAGT---------

------ACCGACGACCTTAACAAGCGTCTCAAAACCAAGATCAATCCAACGACGCATATG

TACACACACTGCGAAATCCATCCAAGCATGCTTCTGGGTATTTGCGCGAGCATCATCCCC

TTTCCCGATCATAACCAGGTAAGCGCACTTCCTGCATTCCGTATGACCA-TATC---ACA

-----------------------------------

>Barrmaelia_moravica_CBS_142769

-------------------------GTCCTCTCCTTGCCAAGTTATTCCGCAACATAGTC

CGACGGATGACTCAGGAGGTTTTGTCACAACTGAAGCGCAGCATCGAGCAGAACAAGCGG

TTCGACATTGCGCTGGCTGTCAAGTCCACCATCATCACCAACGGTCTGAAGTACTCTCTC

GCTACAGGCAATTGGGGCGACCAGAAGAAGGCCATGAGCTCTACCGCTGGTGTGTCGCAG

GTGCTGAACCGATACACATTTGCCTCGACTCTGTCCCATTTGAGGCGGACCAACACCCCC

GTCGGGAGAGACGGCAAGCTTGCCAAACCACGGCAACTTCACAATACGCACTGGGGACTG

GTCTGTCCGGCGGAGACGCCTGAAGGTCAAGCTTGTGGGCTGGTGAAGAATCTTTCCCTC

ATGTGCTCTATCAGCGTGGGCACATCAACGGAACCGATCATCGACTATATGATTACTCGG

AACATGGAAGTCCTGGAGGAGTACGAACCCACGCGATATCCCAATGCTACCAAGATTTTC

CTCAATGGCTCCTGGATCGGTGTTCATCAGGATCCGAAATCCCTCGTCCGGGACGTGCAG

CAGCTGCGTCGAACCAACCAGATCCCGGCTGAGGTATCTCTTGTCCGCGACATTCGAGAT

CGAGAGTTCAAAATCTTCTCTGATGCAGGGCGAGTCATGCGACCATTGTTCGTTGTTGAA

CAAGAGGGCGACGATGAAAATGGCGTTACCAAAGGCGCACTCGCGCTCACGAAGGACATG

GTGTCG------------AGACTCGAGATTGACGCCACTCTCCCACCTGGGAGC------

---GAGGGG---TACTTTGGGTGGCAAGGCCTGGTGAACGCGGGTGTGGTCGAATACCTT

GATGCTGAAGAAGAAGAGACGGCGATGATATGTATGACGCCCGAAGATCTTGAGATATAT

CGAAAGATGAGGACTGGTGAGGCGGTTGAGCAA------------GACGGT---------

------ACCGACGATCTCAACAAGCGCCTCAAAACCAAGATCAATCCGACGACGCACATG

TACACACACTGCGAAATCCATCCAAGCATGCTTCTGGGTATTTGCGCGAGCATCATCCCC

TTCCCCGATCATAACCAGGTAAGCACACTTCCTGCATTCCGTATGACCATTATC---ACA

------GTCTCCTCGAAC-----------------

>Entosordaria_perfidiosa_CBS_142773

----------------------------------------AGCTATTCCGCAATATAGTA

CGCCGGATGACTCAGGAGGTGATGTCACAGCTGAAGCGCAGTATCGACCAAAACAAGCGG

TTTGATATTGCGCTGGCTGTCAAATCCACCATCATCACCAACGGTCTGAAGTACTCTCTT

GCCACGGGCAACTGGGGCGACCAGAAGAAGGCCATGAGCTCTACCGCGGGTGTGTCGCAA

GTGCTGAACCGATACACATTCGCCTCCACTCTGTCCCATTTGAGACGGACCAACACTCCC

GTCGGAAGAGACGGCAAGCTCGCCAAACCACGACAACTTCACAACACGCACTGGGGACTG

GTCTGTCCAGCGGAGACGCCCGAAGGTCAAGCTTGTGGTCTAGTGAAGAACCTATCCCTC

ATGTGCTCCATCAGCGTGGGTACATCAACGGAACCCATCATCGACTATATGATTACTAGA

AATATGGAAGTCCTCGAAGAGTACGAACCCACGCGATATCCCAATGCTACTAAGATCTTT

CTCAATGGCTCCTGGATTGGTGTCCATCAGGATCCGAAGTCGCTCGTCCGAGATGTGCAG

CAGCTGCGCCGAACAAACCAGATCCCGGCTGAGGTATCTCTAGTCCGCGACATTCGAGAT

CGAGAATTCAAGATTTTCTCGGACGCAGGGCGAGTCATGCGACCATTGTTCGTTGTCGAA

CAAGAGGGCGACGAGGAGAATGGCATCAATAAAGGCTCACTCGCTCTCACTAAGGAGATG

GTGCAA------------AGACTGGAGATTGACACCACTCTCCCGCCCGGGAGC------

---GACGAG---TATTTTGGGTGGCAAGGTCTGGTCAATGCGGGTGTCGTCGAATATCTC

GATGCTGAAGAGGAAGAGACTGCGATGATATGCATGACGCCCGAAGATCTTGAGATATAT

CGAAAGATGAAGACTGGTGAGGCGGTTGAGCAG------------GACACT---------

------ACCGACGACTTGAACAAGCGACTCAAAACCAAGATCAACCCGACAACGCACATG

TACACACACTGCGAAATCCATCCAAGCATGCTTTTGGGTATTTGCGCGAGCATTATCCCC

TTCCCCGATCATAACCAGGTAAGCACACTTTCTATATTTCGTATAATTATTATC---CCA

-----------------------------------

>Entosordaria_quercina_CBS_142774

-------------------------GTCCTCTCCTTGCCAAGCTATTCCGCAACATAGTA

CGCCGGATGACTCAGGAGGTCATGGCACAATTGAAACGCAGCATCGACCAGAACAAGCGG

TTTGATATTGCGCTGGCTGTCAAATCCACCATCATCACCAACGGTCTGAAGTACTCTCTT

GCCACAGGCAACTGGGGCGACCAGAAGAAGGCCATGAGCTCTACCGCGGGTGTGTCGCAG

GTGCTGAATCGATACACATTCGCCTCCACTCTGTCCCATTTGAGACGGACCAACACTCCC

GTCGGAAGAGACGGCAAGCTCGCCAAACCACGACAGCTTCACAACACGCACTGGGGACTG

GTCTGTCCAGCGGAGACGCCCGAAGGTCAAGCTTGTGGTCTGGTGAAGAACCTGTCCCTC

ATGTGCTCTATCAGCGTGGGTACATCAACGGAACCCATCATCGACTATATGATTACTAGA

AATATGGAAGTCCTCGAAGAGTACGAACCCACGCGATATCCCAATGCTACTAAGATCTTT

CTCAATGGCTCCTGGATTGGTGTCCATCAAGATCCGAAGTCGCTCGTCCGAGATGTGCAG

CAGCTGCGCCGAACAAACCAGATCCCGGCTGAGGTATCTCTAGTCCGCGACATTCGAGAT

CGAGAATTCAAGATTTTCTCGGACGCAGGGCGAGTCATGCGACCATTGTTCGTTGTCGAA

CAAGAGGGCGACGAGGAGAATGGCATCAATAAAGGCTCACTCGCTCTCACTAAGGAGATG

GTGCAA------------AGACTGGAGATTGACACCACTCTCCCGCCCGGGAGC------

---GATGAG---TATTTTGGATGGCAAGGTCTGGTCAATGCGGGTGTCGTCGAATATCTC

GATGCTGAAGAGGAAGAGACTGCGATGATATGCATGACGCCCGAAGATCTTGAGATATAT

CGAAAGATGAAGACTGGTGAGGCGGTTGAGCAA------------GATGCT---------

------ACCGACGACTTGAACAAGCGACTCAAAACCAAGATCAATCCGACAACGCACATG

TACACACACTGCGAAATCCATCCAAGCATGCTTTTGGGTATTTGCGCGAGCATTATCCCC

TTCCCCGATCATAACCAGGTAAGCACACTTTCTATATTTTGTATAACTGTTATC---CCA

------GTCTCC-----------------------

>Xylaria_apoda_HAST_90080804

GGTAAGAAGCGGTTGGATCTTGCTGGGCCCTTACTTGCCAAACTCTTCCGTAACATAGTT

CGTCGGATGACTCAGGAGGTTTTATCCCATCTCAAGCGGAGCATTGAGCAAGGCAAGCAG

TTCAATATTGCACTAGCAGTTAAGTCAAACATCATCACGAGCGGGCTGAAGTATTCTCTC

GCTACAGGCAACTGGGGCGATCAGAAGAAGGCCATGAGCTCTACTGCTGGTGTTTCGCAA

GTGCTGAACCGATACACATTCGCATCCACATTGTCGCATTTGCGAAGGACAAACACCCCA

GTGGGCCGAGATGGCAAGCTTGCGAAGCCGCGGCAGCTTCACAACACCCACTGGGGTCTC

GTCTGCCCTGCTGAAACGCCCGAAGGCCAGGCGTGTGGCCTAGTTAAAAATCTGTCTCTT

ATGTGCTCTGTCAGCGTGGGTACCTCGACGGAACCTATTATCGAATACATGATTTCCCGA

AATATGGAAGTCTTGGAGGAGTACGAACCTCAAAGATACCCAAATGCCACCAAGATTTTT

CTCAATGGATCATGGATCGGTGTTCATCAAGATCCAAAGGCTCTCGTTAAAGACGTTCAA

CAACTACGCCGGACGAACCAGATTCCAGCGGAAGTATCCTTAATTCGGGATATTCGTGAT

CGCGAATTCAAGATCTTCTCAGACGCCGGCCGTGTCATGCGGCCACTATTTGTGGTCGAG

CAAGATGGCGACCCCGACAAGGATATTGAGAAGGGTACACTGGTTTTGACAAAAGACATG

GTCCGG------------CGACTCGAGATTGACCAGACCTTACCACCGGGAAGC------

---GACGAG---TATTTCGGATGGCAAGGCCTAGTCAATGCTGGTGTCATCGAATATATG

GATGCCGAAGAAGAAGAAACTGCAATGATTTGCATGACGCCCGAAGACTTAGAAGCGTAC

CGACTATCCAAATTGGGCCTTGCGGATCATGATGCTGAA------AACAAC---------

------CTCAACGAACCTAATAAACGACTGAAGACGAAGATCAACCCGACAACTCACACA

TATACGCATTGCGAAATTCACCCAAGTATGCTTCTTGGCATTTGTGCCAGTATCATCCCC

TTCCCCGATCATAACCAGGTAAGTGCCACACGTTCCATTTTGTCACAGACATTAACCTCG

AC-CTAGTCGCCCCGAAACACGTACCAGTCTGCTA

>Nemania_primolutea_HAST_91102001

GGCAAGAAGCGATTAGATCTCGCCGGACCTTTGCTCGCCAAACTCTTCCGCAACATAGTT

CGTCGGATGACTCAGGAAGTACTATCGCATCTCAAGCGAAGTATCGAGCAAGGCAAACAG

TTCAATATCGCCTTAGCCGTCAAGTCAAACATCATTACGAGCGGGCTGAAGTATTCTCTT

GCGACTGGAAACTGGGGTGATCAGAAGAAGGCCATGAGCTCCACCGCCGGTGTTTCGCAG

GTATTGAACCGGTACACATTCGCATCTACCTTGTCGCATTTGCGAAGAACGAACACCCCG

GTCGGCAGAGACGGTAAGCTCGCCAAGCCACGGCAGCTTCACAACACCCACTGGGGACTT

GTGTGTCCGGCCGAGACGCCCGAAGGCCAGGCTTGTGGTCTGGTCAAAAACCTGTCCCTC

ATGTGTTCTATCAGCGTAGGCACTTCAACGGAACCCATCATAGAGTATATGATTTCCCGG

AATATGGAGGTACTGGAGGAATATGAACCACAAAGGTATCCCCACGCAACCAAGATCTTT

CTCAACGGATCATGGATCGGTGTCCATCAAGATCCGAAAGCCTTGGTAAGAGATGTTCAG

CAACTGCGCCGAACGAATCAGATTCCGGCTGAAGTGTCCTTGATTCGAGATATCCGTGAC

CGTGAATTCAAGATCTTCTCAGATGCCGGCCGCGTCATGCGGCCCTTGTACGTGGTTGAG

CAAGAGGACGATCCCGACAATGGTATTCAGAAGGGCACACTAGTCTTAACTAAAAACATT

GTCCGA------------CGACTGGAACTCGACCAAACTCTCCCACCCGGAAGT------

---GAGGAT---TATTTCGGATGGCAAGGCTTAGTCAATGCTGGTGTCATCGAATATATG

GACGCCGAAGAAGAGGAGACTGCCATGATTTGCATGACTCCTGAAGACCTAGAGGCGTAT

CGGATGGCCAAGATGGGCATTCCCAATCCTGACGCAGAA------TACAAC---------

------GTCAACAACCCCAATAAACGACTAAAGACGAAGATCAATCCAACGACCCACACG

TATACCCATTGCGAAATTCACCCAAGTATGCTTCTTGGCATTTGTGCCAGTATCATCCCA

TTCCCCGATCATAACCAGGTAAATACAAGGTAATCTATCTCATTGCAATTGCTAATTTAG

GTTTTAGTCCCCCCGAAACACGTACCAGTCTGCCA

>Nemania_uda_CBS_148422

GGTAAGAAGCGATTGGACCTTGCGGGACCTTTGCTGGCCAAACTCTTCCGCAACATCGTT

CGTCGGATGACGCAGGAAGTTCTGTCACACCTCAAACGAAGTATCGAACAAGGCAAGCAA

TTCAATATTGCCTTGGCCGTGAAGTCGAACATCATTACGAGCGGACTCAAGTACTCTCTC

GCTACAGGAAACTGGGGCGATCAGAAGAAGGCCATGAGCTCCACCGCTGGTGTTTCGCAG

GTGCTGAATCGATACACGTTCGCTTCTACTCTATCACATTTGCGGCGAACGAATACCCCG

GTTGGCCGAGATGGTAAGCTTGCCAAGCCACGGCAGCTTCACAACACCCACTGGGGCCTG

GTATGCCCGGCCGAGACGCCCGAAGGTCAAGCTTGTGGCCTCGTCAAGAACCTATCCCTG

ATGTGCTCCATCAGCGTGGGAACTTCGACGGAACCAATTATAGAATATATGATTTCTCGA

AATATGGAGGTTCTCGAGGAGTACGAACCTCAAAGGTACCCACATGCCACGAAGATATTC

CTCAACGGATCATGGATTGGCATCCATCAGGATCCAAAGGCTCTTGTTAGGGACGTTCAA

CAGTTGCGCCGAACAAACCAGATTCCAGCTGAAGTATCCTTGATTCGAGATATCCGCGAC

CGCGAATTCAAGATCTTCTCAGACGCCGGTCGTGTCATGCGACCCCTATACGTAGTCGAA

CAAGACGATGATCCTGAAAACGGCATCCAGAAGGGTACGCTCGTCTTGAACAAAAACATG

GTTCGG------------CGGCTTGAGATTGACCAGACCCTCCCACCTGGAAGT------

---GAAGAC---TACTTTGGATGGCAGGGCCTGGTCAATGCTGGCGTTATCGAATACATG

GACGCTGAAGAAGAAGAAACTGCAATGATTTGCATGACTCCCGAAGACTTGGAAGCTTAT

CGGATGGCTAGGATGGGTATACCCAACTCCGATGCAGAA------TACGAT---------

------GACGCTCACCCAAACAAGCGGCTCAAGACAAAGATAAACCCGACGACTCACACG

TATACTCATTGTGAAATTCACCCAAGTATGCTTCTGGGCATTTGTGCCAGCATCATTCCA

TTTCCTGATCATAACCAAGTAAGTCTCATATGGCCCATTCCGTTTCATGTATTAATCCCT

TTGCTAGTCTCCCCGAAACACATATCAATCCGCTA

>Nemania_ethancrensonii_CBS_148337

GGCAAGAAGCGTTTGGATTTGGCCGGACCTTTGCTTGCCAAGCTCTTTCGCAACATCGTT

CGTCGGTTGCATCAAGAGGTCCTGACCCACCTGAAACGCAGTGTTGAACAGGGCAAGCAG

TTCAATATCGCTTTAGCTGTTAAGTCAAACATCATTACGAACGGACTCAAATACTCTCTC

GCTACAGGAAACTGGGGTGATCAAAAGAAGGCCATGAGCTCTACCGCTGGTGTTTCGCAG

GTGCTGAACCGATACACATTTGCCTCCACTCTATCCCATCTGCGGCGAACGAATACTCCT

GTTGGCAGAGATGGTAAGCTTGCCAAGCCACGGCAGCTTCACAACACCCACTGGGGTCTC

GTCTGCCCGGCTGAGACCCCTGAAGGCCAGGCTTGTGGCCTCGTCAAAAATTTATCGCTG

ATGTGCTCCGTCAGCGTGGGGACTTCGACGGAACCAATCATAGAATATATGATTTCACGA

AATATGGAGGTGCTCGAGGAATACGAACCTCGGAATTATCCCAACGCCACAAAGATCTTC

CTCAATGGCTCATGGGTTGGCGTGCATCAAGATGCGAAAGCGCTTGTTAGAGATGTTCAG

CAGTTGCGCCGAACGAACCAGATCCCAGCTGAGGTATCTTTGATTCGGGATATCCGCGAC

AGAGAATTCAAGATTTTCTCGGACGCCGGCCGTGTCATGCGGCCTCTGTACGTAGTCGAA

CAAGAGGATGATCCTGAAAACGGCATCCAGAAGGGCACGCTCGTCTTGACCAAAGACATT

GTTCGG------------CGGCTTGAGATCGACCAGACCCTGCCACCTGACAGT------

---GAGGAT---TATTTTGGATGGCAAGGTCTGGTCAATGCTGGCGCTATCGAATACATG

GACGCTGAAGAAGAAGAGACTGCCATGATTTGCATGACTCCCGAAGACTTGGAAGCTTAT

CGAATGGCCCAGATGGGCATACCGAACCCCGACGCAGAA------TACAAG---------

------GCCACCTCTCCAAACAAGCGACTCAAGACAAAGATCAACCCGACGACTCACACG

TATACTCATTGTGAAATTCACCCAAGCATGCTTCTTGGCATCTGTGCCAGCATTATTCCG

TTTCCTGATCATAACCAAGTAAGTACAATTTCGCCGGTCTCGTTCCATATATTAATCTCT

TTGCTAGTCTCCCCGAAACACGTATCAATCTGCCA

>Clypeosphaeria_mamillana_CBS_140735

------------------------------CTGCTCGCCAAGCTCTTCCGCAACATTGCC

AGACGCATGACACAAGAGGTCTTGTCCCAGCTCAAACGGAGTGTTGAGCAAGGCAAGCAG

TTCAACATAGCCCTTGCCGTCAAATCAAACATCATCACGGCTGGTCTCAAGTACTCTCTT

GCCACGGGTAACTGGGGTGACCAGAAGAAAGCAATGAGCTCGACTGCCGGCGTGTCGCAA

GTCCTGAACCGGTACACATTCGCCTCCACATTGTCCCATTTGCGAAGAACGAACACTCCA

GTCGGTCGTGATGGCAAGCTAGCCAAACCCCGCCAGCTTCACAACACCCACTGGGGCTTG

GTATGCCCCGCCGAGACACCCGAGGGTCAAGCTTGTGGTCTAGTCAAGAACCTATCCTTG

ATGTGCTCCGTCAGCGTGGGTACATCGACTGAACCCATCATCGCCTACATGATGAGCCGG

GACATGGGCCTCTTGGAGGAATACGAGCCACAAATATACCCGAACGCTACCAAGATCTTT

CTAAATGGATCGTGGATTGGCGTTCACCACGATCCCAAGTCACTCGTCAAGGATATTCAG

ATGCTGCGGCGTACTAATCAGATCCCAGCTGAAGTGTCTCTGGTGCGAGATATTCGTGAT

CGCGAGTTCAAGATCTTCTCGGATGCCGGTCGTGTTATGCGACCCCTGTTCGTCGTGGAG

CAAGAGGAC------GGGATAGAAGCCAACCGAGGATCTTTGGCGTTGACAAAAGACTTG

GTCCGT------------CGGCTAGAGGGAGACTCGGAGCTTGAGCCTGGAAGC------

---GATGAG---TACTTCGGATGGCAAGGACTCGTCAATGCCGGTGCCATCGAATACGTT

GACGCGGAAGAAGAGGAAACTGCGATGATCTGCATGACTCCTGAGGATCTGGAGAACTAT

CGTCAAGCTAAATTGGGCAATGAAATCGCGGAG------------GATCTG---------

------ACCGAGGCCCCTAACAAACGGTTAAAGACCAAGATGAACCCGACAACGCACATG

TACACCCATTGCGAAATTCACCCCAGCATGCTTCTTGGCATCTGTGCCAGTATCATTCCC

TTCCCAGATCACAACCAGGTATGTGACGTTCGCATAGTGCTAACGAATATCATAGTCTCC

CTCG-----------TAACATTAACCAGTCTTGCC

>Digitodochium_amoenum_CBS_147285

TGGTTACCGCNNNTCGACCTTGCTGGTCCCCTGCTTGCCAAGCTTTTCCGCAATATTGCT

CGACGCATGACGCAGGAGGTCTTGTCGCACCTCAAACGGAGCATCGAGCAAGGCAAGCAG

TTCAACATTGCTCTTGCTGTCAAATCAAACATTATCACCAGTGGCCTGAAGTACTCTCTC

GCAACAGGTAATTGGGGTGACCAGAAGAAAGCAATGAGCTCGACGGCGGGCGTGTCGCAA

GTGCTGAACCGGTATACCTTTGCGTCTACACTATCACATTTGCGAAGAACAAACACCCCC

GTGGGCCGTGATGGCAAGCTCGCGAAACCTCGCCAGCTTCACAATACACATTGGGGTCTG

GTGTGCCCTGCGGAAACACCCGAAGGACAGGCCTGTGGCCTTGTGAAGAATTTATCTCTG

ATGTGCTCAGTGAGCGTAGGCACCTCGACAGAGCCCATTATCGACTATATGATGCGCCGA

GATATGGGTCTCTTGGAAGAGTATGAGCCTCAGAGATATCCGAACGCTACCAAGATCTTC

CTGAACGGATCGTGGATCGGTGTTCATCATGATCCGAAGGCACTTGTCAGAGACATCCAG

CACCTTCGTCGAACAAACCAAATTCCGGCCGAGGTCTCCCTAGTGCGCGACATTCGTGAT

AGAGAGTTCAAGATTTTCTCAGATGCAGGCCGCGTCATGCGACCTTTGTTTGTTGTTGAA

CAGGAGGAC------ACCCTCGAAGCCAATAGAGGCTCACTAGCGCTGACAAAAGACTTG

ATACGA------------CGGCTGGAGGGCGATGGAGATCTTGAGCCTGGAAGC------

---GACCAG---TACTTCGGATGGCAAGGGCTCGTTAATGCCGGTGCTATTGAGTATCTA

GACGCTGAAGAGGAAGAGACGGCGATGATCTGCATGACCCCTGAGGACTTGGAGAACTAT

CGCCTTGCTAAACTAGGTCATGAAGTTGTGGAT------------GATGTG---------

------ACCGATGCACCTAACAAACGGTTGAAGACCCGGATGAACCCTACAACACACATG

TACACTCACTGTGAGATTCATCCCAGTATGCTGCTTGGTATTTGTGCGAGCATCATTCCA

TTCCCCGACCATAATCAGGTATGTGCTGTTGTCAT-GTGC--A-G--T-TACTAATCTCT

ACTGTAGTCCTCCCGTAACAAATACCAGTCA----

>Occultitheca_rosae_HKAS_102393

------------------------------------------------------------

------------------------------------------------------------

------------------------------------------------------------

------------------------------------------------------------

------------------------------------------------------------

--CGGTCGTGATGGCAAGCTCGCCAAACCTCGTCAACTTCACAACACGCACTGGGGTCTA

GTGTGTCCTGCAGAAACCCCTGAAGGACAAGCTTGTGGCTTGGTGAAGAATCTATCCTTG

ATGTGCTCCGTCAGTGTGGGTACATCGACAGAGCCCATCATTGACTACATGATGCGTCGA

GACATGGGTCTATTGGAGGAGTACGAGCCTCAGAGATACCCCAACGCAACCAAGATCTTC

CTTAATGGCTCATGGATTGGTGTTCATCACGACCCCAAGGCGCTCGTCAAAGATATTCAG

CAGCTGCGTCGAACCAACCAGATCCCGGCTGAAGTGTCGCTGGTACGTGATATTCGTGAT

CGCGAGTTCAAGATTTTCTCGGATGCCGGCCGCGTTATGCGACCCTTATTTGTTGTCGAG

CAAGAAGAC------GGTCTCAACACGGTCAAGGGCTCACTGGCGCTAACCAAAGATCTG

GTACGT------------CGGCTCGAGGGTGACACTGATCTTGATTCTGGAAGC------

---GACCAG---TACTTCGGGTGGCAAGGGCTCGTCAATGCCGGCGCGATCGAATATCTG

GATGCCGAAGAGGAAGAGACGGCCATGATATGCATGACCCCCGAGGACTTGGAAAACTAC

CGTCTTGCTAAGACGGGTGAAGCAGTTGAGGAC------------GATCTG---------

------ACCGACGCCCCTAACAAACGGTTGAAGACCAGGATGAACCCCACAACGCACATG

TACACTCATTGTGAGATCCATCCCAGCATGCTTCTCGGTATCTGTGCCAGCATCATTCCA

TTCCCCGATCATAATCA-------------------------------------------

-----------------------------------

>Magnostiolata_mucida_MFLU_19_2133

------------------------------------------------------------

------------------------------------------------------------

------------------------------ATCATCACCAGCGGCCTCAAGTACTCTCTG

GCCACGGGCAACTGGGGTGACCAGAAGAAAGCCATGAGCTCCACGGCGGGTGTGTCTCAA

GTGCTCAACCGATATACCTTTGCGTCGACCCTGTCACATTTGCGGAGGACGAACACGCCC

GTGGGCCGCGACGGCAAGCTCGCGAAGCCTCGGCAGCTCCACAACACGCACTGGGGCCTC

GTATGCCCTGCGGAGACTCCGGAAGGGCAGGCCTGTGGTCTCGTGAAGAACCTGTCGTTG

ATGTGCTCGGTCAGCGTGGGTACGTCGACGGAGCCCATCATCGACTACATGATGCGCCGA

GAAATGGGTCTCCTGGAGGAGTACGAGCCCCAGCATAGCCCGAACGCCACCAAGATCTTC

CTCAACGGATCGTGGATCGGGGTTCACAACGACCCGAAGGCGCTCGTCAAGGATATCCAG

CACCTCCGCCGGATCAACCAGATCCCGTCAGAAGTTTCCCTGGTGCGCGACATTCGTGAC

CGCGAGTTCAAGATCTTCTCTGATGCCGGTCGTGTCATGCGACCCCTGTTTGTGGTGGAG

CAAGAGGAC------AGCCTGGAAGCGACCAAGGGCTGTCTGGCGCTGACGAAAGACATG

GTGCAA------------CGGCTGGAAGGCGACAGCGAGCTCGGGCGCGGCAGC------

---GAAGAG---TACTTCGGATGGCAGGGCTTGGTCAATGCCGGTGCCATAGAGTATCTG

GATGCGGAAGAAGAAGAGACGGCGATGATCTGCATGACGCCCGAGGACCTGGAGATCTAC

CGTCTTTCCAAAGCTGGCCAGGTGGTGCAGGAC------------GATCTG---------

------ACCAAGGCCCCTAACAAGCGGTTGAAGACGAAGATGAACCCGACAACGCACATG

TACACGCACTGCGAGATCCACCCCAGCATGCTTCTGGGCATCTGCGCCAGTATCATTCCA

TTCCCGGACCACAATCA-------------------------------------------

-----------------------------------

>Anthostomelloides_krabiensis_MFLUCC_15_0678

------------------------------------------------------ATCGCT

CGGCGCATGACTCAAGACGTCCTCTCGACCCTCAAGCGAAGCATCGAGCAAGGAAAGCAG

TTCAACATTGCTCTCGCCGTCAAGAGTAACATTATCACCAGTGGCTTGAAATACTCGCTG

GCGACGGGTAACTGGGGTGATCAGAAGAAGGCGGCAAGCTCGACAGCTGGTGTGTCGCAA

GTGCTCAATCGCTACACCTTCGCGTCCACGCTCTCGCACTTGAGACGGACGAACACTCCC

GTCGGTCGTGATGGCAAGCTCGCGAAGCCCCGTCAGCTGCACAATACCCACTGGGGCCTC

GTGTGCCCAGCAGAGACGCCCGAGGGCCAGGCTTGTGGTTTGGTGAAGAATCTGTCGTTA

ATGTGCTCGGTCAGTGTGGGCACTTCGACGGAACCCATCATTGACTACATGATGCGCCGG

GACATGGGGCTGCTCGAAGAGTACGAGCCCCAGATCTACCCCAATGCCACCAAGATCTTT

TTGAACGGATCGTGGATTGGTGTCCACCACGAGCCTAAGGCGCTCGTGAGGGACATTCAG

CTCCTGCGTCGAACTAATCAGATCCCTGCCGAGGTCTCTCTAGTTCGTGACATCCGTGAT

CGAGAGTTCAAGATCTTTTCGGACGCCGGTCGCGTCATGCGTCCCCTGTTCGTCGTGGAG

CAGGAGGGT------GATCTCGAGATCCCCAAAGGATCTCTCACGCTCACGAAGGATATG

ATTCGG------------AGGCTAGATGCCGATGCAGAGCTTGTGCGTGGAAGC------

---GACCAG---TATTTCGGCTGGCAAGGTCTGGTCAACTCGGGTGCCATCGAGTACCTC

GACGCCGAGGAGGAGGAGACAGCAATGATCTGCATGACTCCCGAGGACCTTGAGAACTAC

CGCCTGGCCAGGATGGGCCACGAGGTGGTCGAA------------GACCCA---------

------ATGGAGGCGCCTAACAAGCGTCTCAAGACCAAGATGAACCCTACCACGCACATG

TACACGCATTGCGAGATCCATCCTAGCATGCTCCTTGGCATCTGCGCCAGCATCATCCCG

TTCCCGGATCATAACCAGGTAAGAGCTGTTTGGAGTGCGC-CCCGC---TAA-CCTCGAC

CAC--------------------------------

>Linosporopsis_ischnotheca_CBS_145761

NNNNNNNNNAGACTTGATCTCGCCGGACCTCTATTAGCCAAGTTATTCCGTAATATCGTC

CGGCGCATGACGCAGGAAGTCTTGTCCAACTTGAAGCGAAGTATTGAGCAAGGCAAACAG

TTCAACATCGCGCTGGCTGTCAAGTCGAACATCATCACAAGTGGTCTGAAGTACTCTCTG

GCCACAGGCAACTGGGGCGATCAGAAAAAGGCCATGAGTTCAACAGCAGGCGTGTCTCAG

GTGCTCAACCGATACACATTTGCCTCCACACTCTCACATTTACGACGAACAAATACGCCC

GTGGGTAGAGACGGCAAGCTGGCCAAGCCACGCCAGCTCCACAACACTCATTGGGGTCTT

GTGTGCCCAGCAGAAACACCCGAAGGTCAAGCCTGTGGTTTGGTGAAGAATCTCTCACTC

ATGTGCTCCGTCAGCGTAGGCACGCCTTCGGAACCTATTATAGATTATATGATCAGTAGG

AACATGGAAGTCTTGGAGGAGTACGAACCCCAAAGATATCCCAACGCTACCAAGATCTTC

CTCAACGGATCATGGATCGGTGTACACCAAGAACCAAAGCTCCTTGTCAGGGATGTTCAA

AAGCTACGACGGTCGAACCGAATCCCAGCCGAGGTCTCGTTGGTGCGCGATATTCGTGAT

CGCGAATTCAAAATTTTCTCGGATGCGGGACGCGTCATGCGCCCCCTGTTTGTTGTAGAG

CAGGAAGAT------GGTCCTGAGACTACCAAGGGCTCACTAGCCATAACAAAAGAAATG

ATTCAG------------AGGCTGGAAGCAGACGCGGCGCTCCCACCGGGGCAC------

---GAAGAC---TATTATGGATGGCCAGGCGTGGTTGAAGCGGGAGCCATTGAGTACTTG

GATGCTGAAGAGGAAGAAACTGCTATGATATGCATGACTCCGGAAGACCTGGATTCTTTC

CGGCTGTCCAAGGCGGGATACGACCTAGAGGCT------------GAGAAC---------

------GTCGATGAACCGAATAGACGACTCAAAACAAAACCTAACCCAACTACTCACATG

TACACACATTGCGAGATTCACCCAAGCATGCTTCTCGGTATCTGTGCCAGTATTATTCCT

TTCCCAGATCACAACCAGGTAAGAGGAGACAGTTTTGTTTTT--GCCACTAA-TATGAGT

CTCACAGTCTCCGCGAAATACATACCAATTC----

>Linosporopsis_ochracea_CBS_145999

NNNNNNNNNAGACTCGATCTCGCCGGACCTCTCCTGGCCAAATTGTTCCGCAACATCGTC

AAGCGCATGACGCAGGAAGTAACGGCCCAATTGAAGCGCAGCATTGAGCAGGGCAAGCAG

TTCAACATCGCTTTGGCTGTCAAATCGAATATCATCACAAGTGGCCTGAAGTATTCCCTC

GCCACAGGCAACTGGGGCGATCAGAAGAAAGCTATGAGCTCTACGGCAGGTGTATCTCAG

GTGCTCAATCGATACACATTCGCCTCCACACTTTCCCATTTACGCAGAACCAACACACCC

GTAGGCAGAGACGGCAAGCTGGCCAAGCCTCGTCAGCTTCATAACACCCATTGGGGTCTT

GTGTGCCCGGCAGAAACACCCGAGGGCCAGGCTTGTGGCTTGGTGAAAAATCTGTCCCTG

ATGTGCTCTGTCAGCGTAGGCACGCCATCAGAACCTATTATCGACTACATGATCAGCAGG

AACATGGAAGTCCTGGAAGAGTACGAACCGCAAAGATACCCCAACGCAACCAAGATCTTC

CTCAACGGGTCGTGGATTGGTGTACACCAAGAACCAAAGCTTCTCGTCAGTGATGTTCAG

AAGCTGCGACGGTCGAACAGGATTCCGGCTGAGGTCTCGTTGATCCGCGACATTCGCGAT

CGCGAGTTCAAAATTTTCTCCGATGCGGGACGCGTCATGCGTCCCTTACTGGTTGTAGAG

CAGGAGGAT------GGTCCCGAGACCAAGAAAGGTCACCTGGCCCTCACAAAGGAAATG

ATCAAGGACCTTCAAGCCGGGCTAGACATCGACGCGTCAGGTTCGGCGGGTAGAGATGCG

GTCCAAGAT---TATCCTGGGTGGCAAGGTCTTGTTAACGCTGGAGCTATTGAGTATCTG

GATGCCGAGGAAGAGGAGACTGCCATGATATGCATGACCCCTGAAGACCTGGAAAACTTC

CGATTGGCGAAGGAGGGCGTAATATACGACGAG------------GACAAT---------

------CTCGATGAACCCAACAGACGACTGAAGACCAAACCAAATCCGACGACTCATACT

TACACCCATTGTGAAATTCACCCCAGTATGCTGCTCGGCATTTGTGCCAGTATAATTCCC

TTCCCAGATCACAACCAGGTAAGGGGAAATATTGTTGTTTTAACGATACTAA-CATCAAT

CAAAAAGTCTCCGCGTAACACAT------------

>Emarcea_castanopsidicola_CBS_117105

------------------------------------------------------------

-----------------------------------------------------------A

TTCAATATTGCGCTCGCCGTTAAGTCAAATATTATCACTAGTGGCCTGAAGTACTCGCTC

GCCACAGGAAACTGGGGTGACCAGAAAAAGGCAATGAGCTCTACGGCTGGTGTGTCACAG

GTGCTCAACCGATATACCTTCGCCTCTACGCTCTCTCACTTGCGGAGAACCAACACGCCT

GTGGGAAGAGATGGCAAGCTGGCCAAGCCACGCCAGCTCCACAACACACATTGGGGTCTC

GTCTGTCCCGCCGAGACGCCCGAAGGCCAGGCTTGTGGTCTGGTCAAGAATCTGTCACTC

ATGTGCTCAGTGAGTGTCGGCACGTCCACGGAGCCTATCATCGAGTACATGGAGTCTCGC

AACATGGAAATTCTTGAAGAGTATGAGCCTACGCGATACCCGAACGCTACGAAGATCTTC

CTGAACGGATCATGGATTGGCGTCCATCATGACCCTAAGTCGCTCGTCAGGGATGTCCAA

CAACTCCGTCGGAACAATCAAATTCCATTCGAGGTGTCATTGGTCCGTGATATTCGTGAC

CGTGAATTCAAGATCTTTTCCGACGCAGGGCGGGTTATGCGCCCCCTTTACGTCGTAGAG

CAAGAGGAT------ACAGCGACCGCTGAGAAGGGCCAGCTCGCACTCACTCAGGATCTT

GTCGAC------------AAACTCCGAAACGACAATGAATTGGACCCTGAAGACGCGTAT

---ATGAGG------TTCGGTTGGGCCGGCTTGGTCGATGCGGGTGTCATCGAGTTCCTA

GACGCCGAAGAAGAGGAAACAGCTATGATTTGCATGACACCTGAAGATCTTGAATCGTTC

CGAGCCCACAAGGCAAACCCAAAGACTGAGGAGGAGATGCACGCCGAAAAGCTGAGA---

GAGCAAGAGGCGGCGCCCAACCAAAGGTTGAAGACAAGAATCAACCCTACGACTCACATG

TATACCCATTGTGAGATCCATCCCAGCATGCTTCTCGGCATTTGCGCTAGTATCATTCCT

TTCCCCGATCACAATCAGGTAAG--------------TTTCACCCCGGCCGT-CATC---

-TGGTTG-------------TATACTGACT-----

>Emarcea_eucalyptigena_CBS_139908

------------------------------------------------------------

------------------GTGTTGTCGCACCTTAAGCGGAGCATTGAGCAGGGCAAGCAA

TTCAATATTGCGCTCGCCGTTAAGTCAAATATTATCACTAGTGGTCTGAAGTACTCGCTC

GCCACAGGAAACTGGGGCGACCAGAAGAAGGCAATGAGCTCTACGGCTGGTGTGTCACAG

GTGCTCAACCGATATACCTTCGCCTCTACGCTCTCTCACTTGCGGAGAACCAACACCCCT

GTGGGAAGAGACGGCAAGCTGGCCAAGCCACGCCAGCTCCATAACACACATTGGGGTCTC

GTCTGTCCCGCCGAGACGCCCGAAGGCCAGGCTTGTGGTCTGGTCAAGAATCTGTCACTC

ATGTGCTCAGTGAGTGTCGGCACGTCCACGGAGCCTATCATCGAGTACATGGAGTCTCGC

AACATGGAAATTCTTGAAGAGTATGAGCCTACGCGATACCCGAACGCTACGAAGATCTTC

CTGAACGGATCATGGATTGGCGTCCATCATGACCCTAAGTCGCTCGTCAGGGATGTCCAA

CAACTCCGTCGGAACAATCAAATTCCATTCGAGGTGTCATTGGTCCGTGATATTCGTGAC

CGTGAATTCAAGATCTTCTCCGACGCAGGGCGGGTTATGCGCCCCCTTTACGTCGTGGAG

CAAGAGGAT------ACAGCGACCGCTGAGAAGGGCCAGCTCGCACTCACTCAGGATCTT

GTCGAC------------AAACTCCGAAACGACAATGAATTGGACCCTGAAGACGCGTAT

---ATGAGG------TTTGGTTGGGCCGGCTTGGTCGATGCGGGTGTCATCGAGTTCCTA

GACGCCGAAGAAGAGGAAACAGCTATGATTTGCATGACACCTGAAGATCTTGAATCGTTC

CGAGCCCACAAGGCAAACCCAAAGACTGAGGAGGAGATGCACGCCGAAAAGCTGAGA---

GAGCAAGAGGCGGCGCCCAACCAAAGGTTGAAGACAAGAATCAACCCTACGACTCACATG

TATACCCATTGTGAGATCCATCCCAGCATGCTTCTTGGCATTTGCGCCAGTATCATTCCT

TTCCCCGATCACAATCAGGTAAG--------------TTTCATCCCGGCCGT-CATC---

-TGGTTG-------------TATACTGACT-----

>Kretzschmaria_deusta_CBS_163.93

------------------------------------------------------------

------------------------------------------------------------

------------------------------------------------------------

------------------------------------------------------------

--------------------CGCATCTACCCTATCACATTTGCGAAGAACGAATACCCCT

GTTGGTCGAGACGGCAAACTTGCCAAACCACGGCAGCTTCACAATACTCACTGGGGTCTT

GTCTGCCCGGCCGAGACCCCCGAAGGTCAGGCTTGTGGTCTAGTCAAAAACCTATCCCTC

ATGTGCTCTATCAGCGTCGGCACATCGACAGAGCCTATTATAGATTATATGATTCTACGC

AATATGGAAGTGTTAGAAGAATATGATCATCATAGGTATCCCAACGCCACCAAGGTGTTT

CTCAATGGCGCATGGATTGGCGTTCATCAGGACCCTAAGGCGCTCGTGAAAGATGTGCAA

GAATTGCGCCGGACGAATCAGATCCCAGCCGAAGTATCCCTCATTCGAGATATTCGTGAC

CGCGAGTTCAAAATTTTCTCCGACGCCGGCCGTGTGATGCGGCCCTTGTTCGTAGTCGAA

CAAGAGGATGATTCCGACAAGGGTATCGAGAAGGGCACGCTGGTCTTGACCAAAGACATG

GTCCGG------------AGACTTGAGATTGACCAAACCCTCGCACCCGGAAGT------

---GACGAG---TACTTCGGATGGCAAGGCCTGGTCAATGCTGGTGTCATCGAATATATG

GATGCTGAGGAAGAGGAGACTGCCATGATTTGCATGACTCCCGAGGACCTAGAGAGTTAC

CGATGCATCAAGTTAGGTTTGCCGGATCCTTTCAACAGG------GACGAT---------

------GTCTTCGCCCCGAACAAGCGGCTGAAGACGAAGATAAATCCGACAACCCACATG

TACACTCATTGTGAAATTCACCCCAGCATGCTTCTCGGCATTTGTGCCAGCATCATCCCC

TTCCCCGATCATAACCAAGTAAGTACCCCGCGATCCCTTCCGTCGCAGT-----------

-----------------------------------

>Collodiscula_japonica_CBS_124266

-----------------------------------------GCTCTTCCGCAACATAGCA

CGTCGGATGACTCAAGAGGTCCTATCACAGCTTAAGCGAAGTATCGAGCAAGGCAAGCAG

TTCAATATTGCTCTTGCCGTCAAGTCGAATATTATCACGAGCGGGTTGAAGTACTCACTC

GCGACAGGCAACTGGGGTGACCAGAAGAAGGCCATGAGTTCCACGGCCGGTGTTTCACAG

GTGCTGAACAGATACACATTCGCATCCACCTTGTCACATTTGCGAAGGACGAACACTCCG

GTTGGCAGAGACGGCAAGCTTGCCAAGCCACGGCAGCTTCATAACACACATTGGGGTCTG

GTGTGCCCAGCCGAGACCCCCGAAGGCCAAGCTTGTGGTTTGGTCAAAAACCTGTCCCTC

ATGTGTTCCATCAGCGTTGGTACTTCAACAGAACCTATTATAGAGTATATGATCTCTCGA

AATATGGAGGTGCTGGAAGAGTACGAGCCCCTTCGATACCCGAACGCTACGAAGATCTTC

CTCAACGGGTCATGGGTCGGCATTCATCAAGATCCTAAATCCCTCGTCAGGGATATCCAG

CAATTGCGTCGAACGAATGGAATCCCGGCTGAGGTATCCCTAATCCGAGATATACGTGAT

CGTGAATTCAAGATCTTTTCAGACGCCGGCCGTGTCATGCGGCCCTTGTTTGTGGTTGAG

ACAGAAGAGGACCAGGACGACAGG---AAGAAAGGCATGTTAGTCCTTACAAAAGATATG

GTCAGG------------CGGCTTGAGATCGATCAGACTCTTCCACCTGGAAGC------

---CCCGAG---TATTTTGGATGGGAAGGATTAGTAAACGCCGGTGTGATCGAATACATG

GATGCCGAAGAAGAAGAAACTGCCATGATCTGCATGACGCCTGAAGACTTGGAGGCCTTC

AGAAAGACCAAGACGGGAGAGGCGGACCCTGACGCAGAA------AACAAC---------

------CTGCTGGAGCCCAACAAGCGGCTGAAGACAAGAATGAACCCGACCACTCACACG

TACACGCATTGCGAAATCCACCCCAGCATGCTTCTTGGTATCTGTGCCAGCATCATACCT

TTCCCTGATCACAACCAAGTAGGTCTCAGGTCGTCCATCACATACATCTCACTACTC---

-----------------------------------

>Daldinia_concentrica_CBS_113277

------------------------------------------------------------

------------------------------------------------------------

------------------------------------------------------------

------------------------------------------------------------

---TCTGACCGATACACGTTCGCTTCGACTCTTTCTCATCTAAGGCGGACGAACACGCCT

ATTGGAAGAGATGGAAAACTTGCGAAACCTCGACAGCTGCATAATACCCATTGGGGTCTG

GTCTGTCCGGCCGAAACGCCCGAAGGCCAAGCTTGTGGGTTAGTGAAAAATCTGTCGCTT

ATGTGCTCTATCAGCGTGGGTACGTCAACGGATCCTATCGTAGACTATATGATTACTAGG

AATATGGAAGTCTTGGAGGAATACGAACCTATGCGATACCCTAATGCTACCAAGATCTTC

CTCAACGGGTCTTGGATCGGTGTGCACCAGGATCCCAAGTCTCTAGTTAGAGACGTCCAG

CAACTTCGTCGGGCTAACCAGATCCCCTCTGAAGTGTCACTAGTTCGCGATATCCGTGAT

CGCGAGTTCAAGATCTTCTCAGATGCTGGTCGTGTCATGCGACCCTTATTTGTTGTGCAG

CAAGAGGATAATCCCGAGGCTGGTACTACGAAGGGCTCGTTAGCTCTCAACAAGGAGATG

ATCCAG------------AGGCTGGAGGCAGATGTCGAGCTAGACCCCGAAAGC------

---GAGGAA---TACTTTGGTTGGCAAGGCCTCGTTAACGAGGGGGTTATCGAGTATCTC

GACGCCGAAGAAGAAGAGACGGCTATGATTTGCATGACTCCTGAAGATTTAGAAACCTAC

CGGATGAGCAAGCTCGGATACGATGTATCGCAG------------GATAAC---------

------GGCGATGAGATTAACAAGCGTCTAAAGACTAAAGTGAATCCTACGACGCACATG

TATACACATTGCGAGATCCATCCCAGCATGCTCCTGGGTATCTGCGCAAGCATCATTCCC

TTCCCGGACCACAATCAAGTACGTAATGCCTTAACCCTTGTGTCTGTCACTAACC-----

-----------------------------------

>Entonaema_liquescens_ATCC_46302

------------------------------------------------------------

------------------------------------------------------------

------------------------------------------------------------

------------------------------------------------------------

----TTGACCGATACACTTTCGCCTCGACCCTCTCCCATCTAAGGCGGACGAACACACCT

ATCGGAAGAGACGGGAAGCTCGCAAAGCCTCGACAACTGCACAATACCCATTGGGGTCTG

GTCTGTCCGGCCGAAACGCCCGAAGGCCAAGCCTGTGGGCTGGTGAAGAATTTGTCGCTT

ATGTGCTCGATCAGTGTCGGTACCTCAACGGATCCTATCGTAGACTATATGATTACTAGG

AATATGGAAGTCTTGGAGGAATACGAACCCATGAGATACCCTAACGCCACCAAGATTTTC

CTTAATGGATCTTGGATCGGTGTGCACCAGGATCCTAAGTCTCTGGTGAGAGATGTCCAG

CAGCTTCGTCGGGCCAACCAGATCCCCTACGAAGTGTCGCTGGTTCGCGATATCCGTGAT

CGCGAGTTCAAGATCTTCTCGGATGCCGGCCGTGTTATGCGACCCTTATTTGTGGTGCAG

CAAGAGGACAATCCCGAGGCTGAAACTATGAAGGGCTCCCTGGCTCTCAATAAAGAGATG

ATCCAG------------AGACTGGAGGCGGATGTCGACCTGGACCCGGAAAGC------

---GAAGAA---TATTTTGGTTGGCAAGGCCTGGTCAACGAGGGAGTTATTGAGTACCTT

GATGCGGAGGAAGAAGAAACTGCTATGATTTGCATGACACCCGAAGATTTGGAAACCTAT

CGGCTGTCCAAGCTCGGATATGATGTGTCCCAG------------GACAAC---------

------GGGGATGAGATTAACAAGCGACTAAAGACTAAGGTGAATCCCACGACGCACATG

TATACGCATTGTGAGATTCATCCTAGTATGCTCCTGGGTATCTGCGCGAGCATCATTCCT

TTCCCAGACCACAACCAGGTATGTATGCTTATTATTATTATTAGCTGCTAACGT------

-----------------------------------

>Ruwenzoria_pseudoannulata_MUCL_51394

------------------------------------------------------------

------------------------------------------------------------

------------------------------------------------------------

--------------------CCAGAAGAAAGCGATGAGCTCCACGGCTGGTGTGTCACAG

GTTTTGAACCGATACACTTTCGCCTCGACCCTCTCCCATCTAAGGCGTACCAACACGCCT

ATCGGAAGAGATGGAAAGCTCGCAAAGCCTCGACAGCTGCACAACACACATTGGGGTTTG

GTCTGTCCGGCCGAAACGCCCGAAGNCCAAGCCTGTGGGCTGGTGAAGAACTTGTCGCTG

ATGTGCTCCATCAGTGTTGGTACCTCAACGGATCCTATCGTAGACTATATGATTACTAGG

AATATGGAAGTCTTGGAGGAATACGAACCCATGAGGTACCCTAACGCCACCAAGATCTTC

CTTAACGGATCTTGGATCGGTGTGCACCAGGATCCCAAGTCTCTGGTGAGAGATGTCCAG

CAGCTTCGTCGGGCTAACCAGATCCCCTCGGAAGTGTCGCTGGTTCGCGATATCCGTGAT

CGTGAGTTCAAGATCTTCTCGGATGCCGGTCGTGTTATGCGGCCCTTATTTGTGGTGCAG

CAAGAGGATAATATCGAGGCCGGCACTTCAAAGGGCACGCTTGCTCTTAATAAAGAGATG

ATCCAG------------AGGCTAGAGGCTGATGTCGACTTGGATCCGGAGAGT------

---GAGGAA---TATTTTGGTTGGCAAGGCCTAGTCAACGAGGGTGTTATTGAGTACCTC

GATGCGGAGGAAGAAGAAACCGCTATGATTTGCATGACACCCGAAGATTTGGAAACTTAT

CGGATGTCCAAACTCGGATATGATGTGTCCCAG------------GACAAT---------

------GGAGACGAGATTAACAAGCGGCTTAAGACTAAGGTGAATCCCACAACGCACATG

TATACGCATTGCGAGATTCATCCTAGTATGCTCCTGGGTATCTGCGCGAGCATCATTCCC

TTCCCAGACCACAACCAGGTATGTAATGCTACAATTTTTA--------------------

-----------------------------------

>Hypomontagnella_monticulosa_MUCL_54604

------------------------------------------------------------

---------------------------------------------------AACAGACGT

TTCCAGATCGAACTCGCCGCTNAGCCCGCCATCATCACCAACGGGCTCAAGTACTCGCTG

GCCACGGGTAACTGGGGTGACCAGAAGAAGGCCATGAGCTCGACTGCCGGTGTATCACAG

GTCTTGAATCGATATACGTTTGCGTCGACCTTGTCTCACTTGAGGCGAACGAATACACCC

ATTGGAAGAGATGGGAAGCTCGCGAAGCCTCGACAGCTCCACAACACCCATTGGGGTTTG

GTCTGCCCGGCCGAGACGCCCGAAGGCCAGGCTTGTGGTTTGGTGAAGAACTTGTCCTTG

ATGTGCTCAATCAGCGTGGGTACATCGACAGATCCTATAGTAGACTACATGATTACGAGG

AACATGGAAGTCTTGGAGGAGTATGAGCCAATGCGATACCCTAACGCCACCAAGATCTTC

TTGAATGGTTCTTGGATCGGTGTGCACCAGGATCCCAAGAGCCTCGTCCGAGACGTCCAG

CAGCTGCGCCGAACGAATCAGATCCCTTCCGAAGTATCGCTGGTCCGCGATATTCGTGAC

CGCGAATTCAAGATCTTTTCCGATGCAGGCCGTGTCATGCGTCCCTTATTTGTCGTGCAG

CAAGAGGATGACGAAGCCAACGGTATCACCAAAGGCTCGTTAGCGCTGAATAAGAATATG

ATTCAG------------CGACTAGAGGCGGATGCCGATATCGACCCGAAGAGT------

---GAGGAG---TATTTCGGCTGGCAAGGCCTTGTCGACGAGGGAGCTATCGAATTCCTC

GACGCCGAGGAAGAGGAGACCGCCATGATCTGCATGACGCCCGAGGATCTGGAAATCTAC

CGCCAGAGCAAAGCCGGCATCGAAGTATCCCAG------------GATAAT---------

------GGTGACGAAATCAACAAGCGACTCAAGACCAAGTTGAACCCAACGACACATATG

TATACGCATTGTGAGATCCATCCCAGCATGCTTCTAGGTATCTGTGCGAGCATCATTCCC

TTCCCGGACCACAATCAGGTATGC-A----------------------------------

-----------------------------------

>Jackrogersella_multiformis_CBS_119016

------------------------------------------------------------

----------------------------------------------------------GT

TTCCNAATCGAACTAGCTGCCAAGCCCGCCATCATCACTAACGGTTTGAAATATTCTCTC

GCTACAGGTAACTGGGGTGATCAGAAAAAGGCGATGAGCTCGACGGCTGGTGTATCACAA

GTCTTGAACCGCTACACTTTCTCATCAACTCTTTCCCATTTGAGAAGGACAAACACACCT

ATCGGAAGAGACGGGAAGCTCGCCAAGCCACGACAGCTTCATAACACTCACTGGGGCCTG

GTGTGTCCGGCCGAGACGCCCGAAGGCCAAGCCTGCGGGCTGGTGAAGAATCTGTCGCTC

ATGTGTTCCATCAGCGTGGGAACCTCGACGGATCCTATTGTCGATTATATGATAACGAGA

AATATGGAAGTCCTCGAGGAGTATGAGCCGATGAGATATCCCAACGCCACTAAGATCTTT

CTGAACGGTTCTTGGATCGGTGTTCACCAGGACCCCAAGTCCCTCGTTCGAGACGTACAG

ACGCTCCGCCGTAACAACCAAATTCCTTCTGAAGTCTCGTTGGTTCGCGATATCCGCGAT

CGTGAATTCAAGATCTTCTCGGATGCCGGTCGTGTGATGCGTCCTCTGTTCCGTGTACAA

CAGGATGATCTTCCCGAGGAGGGCATAAACAAGGGCACCTTAGCTCTAACCAAGGATATG

ATCCAG------------CGTTTAGAGGCCGACCTTGATTTGGATCCGGATAGT------

---GAAGAA---TACTTCGGTTGGCAAGGACTAGTCAACGAGGGTGTTGTTGAATTCCTT

GATGCCGAGGAAGAGGAGACAGCCATGATTTGCATGACGCCCGAGGATTTAGAAAACTTC

CGACTGGCCAAGGCTGGATACCAGGTAGTTCTA------------GACAAC---------

------GGAGACGAGGTGAACAAGCGATTGAAGACTAAGTTCAACCCGTCAACGCACATG

TACACTCATTGCGAAATCCACCCCAGTATGCTCCTAGGCATCTGCGCAAGCATCATTCCG

TTCCC-------------------------------------------------------

-----------------------------------

>Spiririma_gaudefroyi_CBS_147284

NNNNNNNNNNNNNTGGATTGGGCTGGTCCTCTGCTGGCCAAGCTCTTCCGCAACATCGTC

CGGCGCATGACGCAAGAAGTCTTGTCTCACCTGAAGCGAAGCATCGAACAGGGCAAGCAG

TTCAACATCGCCCTGGCCGTCAAGTCCAACATCATCACGAGCGGTCTCAAGTACTCGCTC

GCCACAGGCAACTGGGGCGATCAGAAGAAAGCTATGAGTTCGACCGCAGGTGTATCCCAG

GTGCTCAACAGATACACCTTTGCGTCCACGCTCTCGCATTTGCGAAGAACCAACACGCCC

GTGGGCCGAGATGGTAAGCTCGCCAAACCCCGCCAGCTTCACAACACCCACTGGGGTCTG

GTTTGCCCTGCCGAAACACCCGAGGGTCAGGCTTGTGGTCTGGTGAAGAACTTGTCCCTC

ATGTGCTCCGTCAGTGTCGGTACTTCGACAGAGCCTATCATAGACTACATGGCCTCTCGA

AATATGGAAATTCTGGAAGAGTACGAGCCCCAGCGCTATCCTAACGCCACTAAGATCTTT

CTCAACGGCTCATGGATCGGTGTACACCATGATCCGAAGTCTCTTGTAAGAGATGTCCAG

CAGCTGCGTCGAACCAACCAGATCCCTGCAGAAGTGTCCTTGGTTCGCGACATTCGTGAC

CGCGAATTCAAGATCTTTTCAGACGCCGGGCGAGTCATGCGACCTTTGTTTGTCGTTGAA

CAAGAAGAC------ACGCGTAACCGCGTGAAAGGACAATTGGCCCTGACTAAAGACATG

TGTCTG------------AGGATCGAGGCCGACACATCGCCTGACAATATAGCTAAA---

---GGCGAA---TATTATGGCTGGGAGGGCTTGGTCGACGATGGTGCGATCGAGTTTTTG

GACGCCGAGGAAGAAGAGACGGCCATGATCTGCATGACGCCCGAAGATCTTGAGAATTAC

CGTCTTACCAAACTTGGGCAGGGTTATGGCGAC------------GAGAAC---------

---CCTGCGGACGCGCCTAACAAGCGCCTGAAAACCAAGATCAACCCTACCACTCACATG

TACACCCATTGCGAGATTCATCCGAGCATGCTTTTAGGTATCTGCGCGAGTATAATACCC

TTCCCAGACCATAATCAGGTAAGTGCAGTTGCTGCCTTCTGACCGATACTAA--ATCCCA

ATGACAGTCTCCCCGTAATACCTAATAAT-----N

>Muscodor_thailandica_MFLUCC_17_2669

----------------------------------------------------ACATTGTC

GGACGCATGACGCAAGAAGTCCTGTCTCACTTGAAGCGAAGCATCGAACAGGGCAAGCAG

TTCAACATCGCCCTGGCCGTTAAGTCTAACATCATTACCGCCGGGCTCAAGTACTCCCTC

GCCACCGGCAACTGGGGCGACCAGAAGAAGGCCATGTCCTCGACCGCAGGTGTCTCCCAG

GTGCTGAACAGATACACCTTTGCGTCCACCCTCTCGCATCTGCGGAGGACCAACACTCCG

GTCGGCCGAGATGGCAAGCTTGCCAAGCCCCGCCAGCTTCACAACACGCATTGGGGCCTG

GTCTGTCCTGCTGAGACGCCCGAGGGCCAGGCCTGTGGTCTGGTCAAGAACTTGTCTCTC

ATGTGCTCCGTCAGTGTCGGCACTTCGACAGAGCCCATCATCGAGTACATGGCCTCGCGA

AACATGGAGATTCTGGAAGAGTACGAACCCCAGCGCTATCCCAACGCCACCAAGATCTTT

CTCAACGGCTCATGGATCGGTGTACATCACGACCCCAAGGCTCTGGTGAGAGACGTCCAG

CAGCTGCGCCGAAGCAGTCAGATCCCTCCAGAAGTATCTTTGGTTCGTGACATTCGTGAT

CGTGAGTTCAAGATCTTTTCGGATGCGGGCCGAGTCATGCGGCCCTTGTACGTTGTGGAA

CAAGAGGAT------ACGCGAACTGCCGTCAAGGGACAACTGGCCCTGGACAGAGCCATG

CTCCAG------------AAACTCATAGATGACGGAGACCCTGACAAGGTAGCTGAG---

---AGAGGGTACTACTACGGCTGGAAGGGCTTGGTCGACGACGGTGCGATCGAGTATTTG

GACGCCGAGGAAGAAGAGACGGCCATGATTTGCATGACACCCGAGGATCTTGAAAACTAC

CGACTTACCAAACTTGGAGAGGGCGGCAGCGAC------------GAGAAC---------

---CCTGCCGACGCGCCTAATAAGCGCCTGAAGACCAAGATCAACCCCACTACTCACATG

TACACTCATTGTGAAATTCATCCGAGCATGCTGTTGGGCATCTGCGCAAGTATAATACCC

TTCCCAGATCATAATCAGGTAAGTGCAGCC------------------------------

-----------------------------------

>Muscodor_ziziphi_MFLUCC_17_2662

------------------------------------------------------------

---------------------------------------------------GGCAAGCAG

TTCAACATCGCCCTGGCCGTTAAGTCTAACATCATTACCGCCGGGCTCAAGTACTCCCTC

GCCACCGGCAACTGGGGCGACCAGAAGAAAGCCATGTCCTCGACCGCCGGTGTCTCCCAG

GTGCTGAACAGATACACCTTTGCGTCCACACTCTCGCATCTGCGGAGGACCAACACTCCG

GTCGGCCGAGATGGCAAGCTTGCCAAGCCCCGCCAGCTTCACAACACGCATTGGGGCCTG

GTCTGTCCTGCTGAGACGCCCGAGGGCCAGGCCTGTGGTCTGGTCAAGAACTTGTCTCTC

ATGTGCTCCGTCAGTGTCGGTACTTCGACTGAGCCCATCATCGAGTACATGGCCTCGCGA

AACATGGAGATTCTGGAAGAGTACGAACCCCAGCGCTATCCCAACGCCACTAAGATCTTT

CTCAACGGCTCATGGATCGGTGTACACCACGACCCCAAGGCTCTGGTGAGAGATGTCCAG

CAGCTGCGCCGAAGCAGTCAGATCCCTCCAGAAGTATCTTTGGTTCGTGACATTCGTGAT

CGTGAGTTCAAGATCTTTTCGGATGCGGGCCGAGTCATGCGGCCCTTGTACGTTGTGGAA

CAAGAGGAT------ACGCGAACTGCCGTCAAGGGACAACTGGCCCTGGACAGAGCCATG

CTCCAG------------AAACTCATAGATGACGGAGACCCTGACAAGGTAGCTGAG---

---AGAGGGTACTACTACGGCTGGAAGGGCTTGGTCGACGACGGTGCGATCGAGTATTTA

GACGCCGAGGAAGAAGAGACGGCCATGATTTGCATGACACCCGAGGATCTTGAAAACTAC

CGACTTACCAAACTTGGAGAGGGCGGCAGCGAC------------GAGAAT---------

---CCTGCCGACGCGCCTAATAAGCGCCTGAAGACCAAGATCAACCCCACTACTCACATG

TACACACATTGTGAAATTCATCCGAGCATGCTGTTGGGCATCTGCGCAAGTATAATACCC

TTCCCAGATCATAATCAGGTAAGTGCAACCACGGTTTGTCGCCCGATCGTG---------

-----------------------------------

>Muscodor_equiseti_JCM_18233

------------------------------------------------------------

------------------------------------------------------------

------------------------------------------------------------

------------------------------------------------------------

------------------------------------------------------------

------------------------------------------------------------

------------------------------------------------------------

------------------------------------------------------------

------------------------------------------------------------

------------------------------------------------------------

------------------------------------------------------------

------------------------------------------------------------

------------------------------------------------------------

------------------------------------------------------------

------------------------------------------------------------

------------------------------------------------------------

------------------------------------------------------------

------------------------------------------------------------

------------------------------------------------------------

------------------------------------------------------------

-----------------------------------

>Muscodor_vitigena_MONT_P_15

------------------------------------------------------------

------------------------------------------------------------

------------------------------------------------------------

------------------------------------------------------------

------------------------------------------------------------

------------------------------------------------------------

------------------------------------------------------------

------------------------------------------------------------

------------------------------------------------------------

------------------------------------------------------------

------------------------------------------------------------

------------------------------------------------------------

------------------------------------------------------------

------------------------------------------------------------

------------------------------------------------------------

------------------------------------------------------------

------------------------------------------------------------

------------------------------------------------------------

------------------------------------------------------------

------------------------------------------------------------

-----------------------------------

>Muscodor_suturae_MSUB_2380

------------------------------------------------------------

------------------------------------------------------------

------------------------------------------------------------

------------------------------------------------------------

------------------------------------------------------------

------------------------------------------------------------

------------------------------------------------------------

------------------------------------------------------------

------------------------------------------------------------

------------------------------------------------------------

------------------------------------------------------------

------------------------------------------------------------

------------------------------------------------------------

------------------------------------------------------------

------------------------------------------------------------

------------------------------------------------------------

------------------------------------------------------------

------------------------------------------------------------

------------------------------------------------------------

------------------------------------------------------------

-----------------------------------

>Muscodor_coffeana_COAD_1842

------------------------------------------------------------

------------------------------------------------------------

------------------------------------------------------------

------------------------------------------------------------

--------------------------CACACTCTCGCATCTGCGGAGAACCAACACTCCG

GTCGGCCGAGACGGCAAGCTTGCGAAGCCCCGCCAGCTTCACAACACACACTGGGGCCTG

GTCTGTCCCGCTGAGACGCCCGAGGGCCAGGCCTGTGGTCTGGTCAAGAACTTGTCCCTC

ATGTGCTCCGTCAGTGTCGGTACTTCGACAGAGCCCATCATCGAGTACATGGCCTCGCGA

AACATGGAGATTCTGGAAGAGTACGAGCCCCAGCGCTATCCTAACGCCACCAAGATCTTC

CTCAACGGCTCATGGATCGGTGTACATCACGACCCCAAGTCTCTGGTGAGAGATGTCCAG

CAGCTGCGCCGAAGCAATCAGATCCCTGCAGAGGTATCTTTGGTTCGTGACATTCGTGAT

CGTGAGTTCAAGATCTTTTCGGATGCGGGCCGAGTCATGCGGCCCCTGTACGTTGTGGAA

CAAGAGGAT------ACGGGAACTGCCGTCAAGGGACAACTGGCCCTGACCAGAGAGATG

ATCAAA------------GAACTCAAAGATGACGGAGAGCCTGAAAAGGTATTAAAG---

---AGAGGATACTACTACGGCTGGCAGGGCTTGGTCCACGCCGGTGCGATCGAGTATTTA

GACGCCGAGGAAGAAGAGACGGCCATGATTTGCATGACGCCCGAAGACCTTGAAAACTAC

CGACTTAACAAACTTGGTGAGGGCGGCGGCGAC------------GAGAAC---------

---CCTGCTGACGCGCCTAACAAGCGTCTGAAGACCAAGATCAACCCTACTACCCACATG

TACACTCATTGTGAGATTCATCCGAGCATGCTGTTGGGCATCTGCGCAAGTATAATACCC

TTCCCAGATCACAATCAGGTAAGGGCAGCCACGGTC------------------------

-----------------------------------

>Muscodor_yucatanensis_MEXU_25511

------------------------------------------------------------

------------------------------------------------------------

------------------------------------------------------------

------------------------------------------------------------

------------------------------------------------------------

------------------------------------------------------------

------------------------------------------------------------

------------------------------------------------------------

------------------------------------------------------------

------------------------------------------------------------

------------------------------------------------------------

------------------------------------------------------------

------------------------------------------------------------

------------------------------------------------------------

------------------------------------------------------------

------------------------------------------------------------

------------------------------------------------------------

------------------------------------------------------------

------------------------------------------------------------

------------------------------------------------------------

-----------------------------------

>Muscodor_sp._SMH_1255

------------------------------------------------------------

------------------------------------------------------------

------------------------------------------------------------

------------------------------------------------------------

------------------------------------------------------------

------------------------------------------------------------

------------------------------------------------------------

------------------------------------------------------------

------------------------------------------------------------

------------------------------------------------------------

------------------------------------------------------------

------------------------------------------------------------

------------------------------------------------------------

------------------------------------------------------------

------------------------------------------------------------

------------------------------------------------------------

------------------------------------------------------------

------------------------------------------------------------

------------------------------------------------------------

------------------------------------------------------------

-----------------------------------

>Muscodor_brasiliensis_LGMF_1256

------------------------------------------------------------

---------------------------------AAGCGGAGCATCGAGCAGGGCAAGCAG

TTCAACATCGCCCTGGCGGTCAAGTCTAACATCATTACCGCCGGGCTCAAGTACTCCCTC

GCCACGGGCAACTGGGGCGACCAGAAGAAGGCCATGTCCTCGACCGCAGGTGTTTCCCAG

GTGCTGAACAGATACACCTTCGCGTCCACGCTCTCGCATCTGCGGAGGACCAACACTCCG

GTCGGCCGAGACGGCAAGCTTGCCAAGCCCCGCCAGCTTCACAACACGCACTGGGGCCTC

GTCTGTCCTGCGGAGACGCCCGAGGGCCAGGCCTGTGGTCTGGTCAAGAACTTGTCCCTC

ATGTGCTCCGTCAGTGTCGGCACTTCGACGGAGCCCATCATCGAGTACATGGCCTCGCGA

AACATGGAGATTCTGGAAGAGTACGAGCCCCAGCGCTATCCCAACGCCACCAAGATCTTT

CTCAACGGCTCGTGGATCGGTGTCCATCATGATCCCAAGTCTCTGGTGAGAGATGTCCAG

CAGCTGCGCCGAAGCTCTCAGATCCCTCCAGAAGTATCTTTGGTTCGTGACATTCGCGAT

CGTGAGTTCAAGATCTTTTCGGATGCGGGCCGAGTCATGCGGCCCTTGTACGTTGTGGAA

CAAGAGGAC------ACGCGAGCTGCCGTCAAGGGACAACTGGCCCTGACCAGAGCCATG

CTCGAT------------AAACTCATAGACGACGGAAAGCCCGACAAGGCACCTGAT---

---TACGAA---TACTACGGCTGGGAGGGCTTGGTCCACGACGGCGCGATCGAGTATCTA

GACGCCGAGGAAGAAGAGACGGCCATGATTTGCATGACGCCCGAAGACCTTGAAAACTAC

CGACTTAGCAAGCTTGGGGAGGGCGGCAGTGAA------------GAGAAC---------

---CCTGCCGACGCGCCTAACAAGCGTCTGAAGACCAAGATCAACCCTACTACCCACATG

TACACCCATTGTGAGATTCATCCGAGCATGCTGTTGGGCATCTGCGCAAGTATAATACCC

TTCCCAGATCATAATCA-------------------------------------------

-----------------------------------

>Muscodor_alba_9_6

------------------------------------------------------------

------------------------------------------------------------

------------------------------------------------------------

------------------------------------------------------------

------------------------------------------------------------

------------------------------------------------------------

------------------------------------------------------------

------------------------------------------------------------

------------------------------------------------------------

------------------------------------------------------------

------------------------------------------------------------

------------------------------------------------------------

------------------------------------------------------------

------------------------------------------------------------

------------------------------------------------------------

------------------------------------------------------------

------------------------------------------------------------

------------------------------------------------------------

------------------------------------------------------------

------------------------------------------------------------

-----------------------------------

>Muscodor_alba_MONT_620

------------------------------------------------------------

------------------------------------------------------------

------------------------------------------------------------

------------------------------------------------------------

------------------------------------------------------------

------------------------------------------------------------

------------------------------------------------------------

------------------------------------------------------------

------------------------------------------------------------

------------------------------------------------------------

------------------------------------------------------------

------------------------------------------------------------

------------------------------------------------------------

------------------------------------------------------------

------------------------------------------------------------

------------------------------------------------------------

------------------------------------------------------------

------------------------------------------------------------

------------------------------------------------------------

------------------------------------------------------------

-----------------------------------

>Muscodor_crispans_MONT_2347

------------------------------------------------------------

------------------------------------------------------------

------------------------------------------------------------

------------------------------------------------------------

------------------------------------------------------------

------------------------------------------------------------

------------------------------------------------------------

------------------------------------------------------------

------------------------------------------------------------

------------------------------------------------------------

------------------------------------------------------------

------------------------------------------------------------

------------------------------------------------------------

------------------------------------------------------------

------------------------------------------------------------

------------------------------------------------------------

------------------------------------------------------------

------------------------------------------------------------

------------------------------------------------------------

------------------------------------------------------------

-----------------------------------

>Muscodor_musae_JCM_18230

------------------------------------------------------------

------------------------------------------------------------

------------------------------------------------------------

------------------------------------------------------------

------------------------------------------------------------

------------------------------------------------------------

------------------------------------------------------------

------------------------------------------------------------

------------------------------------------------------------

------------------------------------------------------------

------------------------------------------------------------

------------------------------------------------------------

------------------------------------------------------------

------------------------------------------------------------

------------------------------------------------------------

------------------------------------------------------------

------------------------------------------------------------

------------------------------------------------------------

------------------------------------------------------------

------------------------------------------------------------

-----------------------------------

>Muscodor_oryzae_JCM_18231

------------------------------------------------------------

------------------------------------------------------------

------------------------------------------------------------

------------------------------------------------------------

------------------------------------------------------------

------------------------------------------------------------

------------------------------------------------------------

------------------------------------------------------------

------------------------------------------------------------

------------------------------------------------------------

------------------------------------------------------------

------------------------------------------------------------

------------------------------------------------------------

------------------------------------------------------------

------------------------------------------------------------

------------------------------------------------------------

------------------------------------------------------------

------------------------------------------------------------

------------------------------------------------------------

------------------------------------------------------------

-----------------------------------

>Muscodor_rosea_MONT_2098

------------------------------------------------------------

------------------------------------------------------------

------------------------------------------------------------

------------------------------------------------------------

------------------------------------------------------------

------------------------------------------------------------

------------------------------------------------------------

------------------------------------------------------------

------------------------------------------------------------

------------------------------------------------------------

------------------------------------------------------------

------------------------------------------------------------

------------------------------------------------------------

------------------------------------------------------------

------------------------------------------------------------

------------------------------------------------------------

------------------------------------------------------------

------------------------------------------------------------

------------------------------------------------------------

------------------------------------------------------------

-----------------------------------

>Muscodor_kashayum_NFCCI_2947

------------------------------------------------------------

------------------------------------------------------------

------------------------------------------------------------

------------------------------------------------------------

------------------------------------------------------------

------------------------------------------------------------

------------------------------------------------------------

------------------------------------------------------------

------------------------------------------------------------

------------------------------------------------------------

------------------------------------------------------------

------------------------------------------------------------

------------------------------------------------------------

------------------------------------------------------------

------------------------------------------------------------

------------------------------------------------------------

------------------------------------------------------------

------------------------------------------------------------

------------------------------------------------------------

------------------------------------------------------------

-----------------------------------

>Muscodor_tigerensis_NFCCI_3172

------------------------------------------------------------

------------------------------------------------------------

------------------------------------------------------------

------------------------------------------------------------

------------------------------------------------------------

------------------------------------------------------------

------------------------------------------------------------

------------------------------------------------------------

------------------------------------------------------------

------------------------------------------------------------

------------------------------------------------------------

------------------------------------------------------------

------------------------------------------------------------

------------------------------------------------------------

------------------------------------------------------------

------------------------------------------------------------

------------------------------------------------------------

------------------------------------------------------------

------------------------------------------------------------

------------------------------------------------------------

-----------------------------------

>Muscodor_cinnanomi_BCC_38842

------------------------------------------------------------

------------------------------------------------------------

------------------------------------------------------------

------------------------------------------------------------

------------------------------------------------------------

------------------------------------------------------------

------------------------------------------------------------

------------------------------------------------------------

------------------------------------------------------------

------------------------------------------------------------

------------------------------------------------------------

------------------------------------------------------------

------------------------------------------------------------

------------------------------------------------------------

------------------------------------------------------------

------------------------------------------------------------

------------------------------------------------------------

------------------------------------------------------------

------------------------------------------------------------

------------------------------------------------------------

-----------------------------------

>Muscodor_camphorae_NFCCI_3236

------------------------------------------------------------

------------------------------------------------------------

------------------------------------------------------------

------------------------------------------------------------

------------------------------------------------------------

------------------------------------------------------------

------------------------------------------------------------

------------------------------------------------------------

------------------------------------------------------------

------------------------------------------------------------

------------------------------------------------------------

------------------------------------------------------------

------------------------------------------------------------

------------------------------------------------------------

------------------------------------------------------------

------------------------------------------------------------

------------------------------------------------------------

------------------------------------------------------------

------------------------------------------------------------

------------------------------------------------------------

-----------------------------------

>Muscodor_ghoomensis_NFCCI_3234

------------------------------------------------------------

------------------------------------------------------------

------------------------------------------------------------

------------------------------------------------------------

------------------------------------------------------------

------------------------------------------------------------

------------------------------------------------------------

------------------------------------------------------------

------------------------------------------------------------

------------------------------------------------------------

------------------------------------------------------------

------------------------------------------------------------

------------------------------------------------------------

------------------------------------------------------------

------------------------------------------------------------

------------------------------------------------------------

------------------------------------------------------------

------------------------------------------------------------

------------------------------------------------------------

------------------------------------------------------------

-----------------------------------

>Muscodor_indica_NFCCI_3235

------------------------------------------------------------

------------------------------------------------------------

------------------------------------------------------------

------------------------------------------------------------

------------------------------------------------------------

------------------------------------------------------------

------------------------------------------------------------

------------------------------------------------------------

------------------------------------------------------------

------------------------------------------------------------

------------------------------------------------------------

------------------------------------------------------------

------------------------------------------------------------

------------------------------------------------------------

------------------------------------------------------------

------------------------------------------------------------

------------------------------------------------------------

------------------------------------------------------------

------------------------------------------------------------

------------------------------------------------------------

-----------------------------------

>Muscodor_suthepensis_JCM_18232

------------------------------------------------------------

------------------------------------------------------------

------------------------------------------------------------

------------------------------------------------------------

------------------------------------------------------------

------------------------------------------------------------

------------------------------------------------------------

------------------------------------------------------------

------------------------------------------------------------

------------------------------------------------------------

------------------------------------------------------------

------------------------------------------------------------

------------------------------------------------------------

------------------------------------------------------------

------------------------------------------------------------

------------------------------------------------------------

------------------------------------------------------------

------------------------------------------------------------

------------------------------------------------------------

------------------------------------------------------------

-----------------------------------

>Muscodor_darjeelingensis_NFCCI_3095

------------------------------------------------------------

------------------------------------------------------------

------------------------------------------------------------

------------------------------------------------------------

------------------------------------------------------------

------------------------------------------------------------

------------------------------------------------------------

------------------------------------------------------------

------------------------------------------------------------

------------------------------------------------------------

------------------------------------------------------------

------------------------------------------------------------

------------------------------------------------------------

------------------------------------------------------------

------------------------------------------------------------

------------------------------------------------------------

------------------------------------------------------------

------------------------------------------------------------

------------------------------------------------------------

------------------------------------------------------------

-----------------------------------

>Muscodor_strobelii_NFCCI_2907

------------------------------------------------------------

------------------------------------------------------------

------------------------------------------------------------

------------------------------------------------------------

------------------------------------------------------------

------------------------------------------------------------

------------------------------------------------------------

------------------------------------------------------------

------------------------------------------------------------

------------------------------------------------------------

------------------------------------------------------------

------------------------------------------------------------

------------------------------------------------------------

------------------------------------------------------------

------------------------------------------------------------

------------------------------------------------------------

------------------------------------------------------------

------------------------------------------------------------

------------------------------------------------------------

------------------------------------------------------------

-----------------------------------

>Muscodor_yunnanensis_CGMCC_3.18908

--------------------------------------------------GCACATTGTC

CGACGCATGACGCAAGAAGTTCTGTCTCACTTAAAGCGTAGCATCGAACAGGGCAAGCAG

TTCAACATTGCTCTGGCTGTGAAGTCCAACATTATCACCGCCGGGCTCAAGTACTCGCTT

GCCACTGGCAACTGGGGCGATCAGAAGAAAGCTATGTCCTCGACCGCAGGTGTCTCCCAG

GTTTTGAACAGGTACACCTTTGCGTCCACCCTCTCGCATCTCAGGAGAACAAACACTCCG

GTCGGGCGAGATGGTAAACTTGCCAAGCCCCGACAGCTTCACAACACACATTGGGGGTTG

GTCTGTCCCGCGGAGACGCCCGAGGGCCAAGCTTGTGGTCTGGTGAAGAACTTATCTCTC

ATGTGCTCCGTCAGTGTCGGTACTTCGACAGAGCCCATCATTGACTACATGGCCTCCCGT

AACATGGAGATTCTGGAGGAGTACGAGCCCGGCCGCCATCCCAACGCTACCAAGATCTTC

CTCAACGGCTCGTGGATTGGTGTACACCATGATCCCAAGTTCCTGGTCAGAGATGTTCAG

CAGCTGCGCCGAAGCGGCCAGATCCCTGCAGAGGTGTCTTTGATTCGCGACATTCGTGAT

CGTGAATTCAAGATCTTTTCGGACGCTGGACGAGTCATGCGACCCTTGTATGTTGTGGAA

CAGGAAGAT------TCCCATGATCGTGTCAAAGGACAATTGGCCGTTACTAGACCTATG

ATAAAG------------AAACTTGAGAAGGACCAACAGCCTGGATTACGTAAGCAA---

---GGTTGGCAATATTATGGCTTTGAGGGTTTGATCCTCGACGGTGCGATCGAGTATCTA

GACGCAGAGGAAGAAGAAACCTCCATGATCTGCATGACGCCTGAAGATCTCGAAAATTAC

CGTCTTGCGAAACTCGGGGAGGGTGACGGTGAG------------GATGACGACAGAGAT

GATGCTGAGCATGCACCCAACAAGCGCCTTAAAACCAAGATTAACCCTACCACCCACATG

TACACCCATTGCGAGATTCACCCGAGCATGCTGTTAGGTATCTGCGCAAGTATCATCCCC

TTTCCAGATCACAATCAGGTAAGGGCGGCACTGGTTCTTTT-------------------

-----------------------------------

>Muscodor_fengyangensis_CGMCC_2862

------------------------------------------CTTTTCCGTAACATCGTC

CGCCGCATGACGCAGGAGGTCTTGTCCCACTTGAAGCGAAGCATCGAGCAGGGCAAGCAG

TTCAACATCGCCCTCGCCGTCAAGGCCAATATTATTACTAGCGGTCTCAAGTACTCCCTC

GCCACCGGTAACTGGGGCGATCAGAAGAAAGCCATGAGCTCCACCGCTGGCGTGTCCCAG

GTGCTCAACAGATATACTTTCGCCTCCACGCTCTCGCATTTGCGACGAACCAACACGCCC

GTAGGTCGAGATGGCAAGCTCGCCAAGCCCCGTCAGCTTCACAACACCCACTGGGGCCTA

GTTTGTCCTGCCGAGACCCCCGAGGGTCAGGCCTGTGGTCTGGTGAAGAACTTGTCTCTT

ATGTGCTCCGTCAGTGTCGGCACCTCGACGGAGCCCATCATTGAGTACATGGCGTCCCGG

AACATGGAGATTCTCGAAGAATACGAACCCCAGCGCTATCCCAATGCCACCAAGATCTTT

CTCAACGGCTCATGGATCGGCGTCCACCACGATCCCAAGTCTCTCGTGAGAGATGTCCAG

CAGCTACGCCGAACCAATCAGATCCCTGCAGAAGTATCATTGGTTCGCGACATCCGTGAT

CGCGAGTTCAAGATCTTCTCGGACGCTGGCCGAGTCATGCGACCCTTGTTTGTCGTCGAA

CAAGAGGAC------ACGCCCGACCGCCTGAAGGGACAGCTAGCTCTCACCAAAGAAATG

GCCAAG------------AAAATCGAAGCCGATCAAGATCCGGCAACGATAGAAAGG---

---AACGAA---TATTATGGTTGGGAGGGGTTGGTCGATGACGGCGCCATTGAGTATCTG

GATGCCGAGGAGGAAGAGACGGCCATGATTTGCATGACGCCCGAAGA-------------

------------------------------------------------------------

------------------------------------------------------------

------------------------------------------------------------

------------------------------------------------------------

-----------------------------------

>Camillea_obularia_ATCC_28093

------------------------------------------------------------

------------------------------------------------------------

------------------------------------------------------------

------------------------------------------------------------

---GTTGACAGATACACATTTTCCTCTACTCTATCTCACTTGCGACGTACGAATACTCCG

GTTGGAAGAGATGGCAAGCTGGCCAAGCCACGACAACTTCACAATACTCACTGGGGTCTT

GTCTGTCCTGCAGAGACCCCTGAAGGTCAGGCTTGTGGTCTGGTGAAGAACCTGTCATTG

ATGTGCTCCATCAGTGTCGGTACATCGACAGAGCCTATCATTGATTACATGATTACTCGT

AATATGGAGGTGCTTGAAGAGTACGAGCCTATGCGATACCCTCACGCCACCAAGATCTTT

CTCAATGGTTCTTGGATTGGTGTTCACCAAGACCCCAAGGCGCTGGTCAGGGATGTCCAG

CAACTACGTCGGAGCAATCAGATACCAGCTGAGGTGTCCCTAGTTCGTGACATCCGAGAC

CGTGAGTTCAAGATCTTTTCAGATGCCGGTCGCGTCATGAGACCTTTGTTCGTTGTCGAG

CAGGAGGACGTGACTGAGACCGGTGTTGAGAAGGGAACGTTGGCTCTCAACAAGAGCATG

GTGAGA------------AGACTTGAGCTCGACCAGACCCTCCCCCCTGGGAGC------

---GATGAG---TATTATGGCTGGCCAGGTTTGGTGAATGACGGTGTCATCGAGTACCTT

GATGCAGAAGAGGAAGAGACGGCCATGATATGTATGACACCAGAAGATCTCGAGATCTTC

CGGATGACGAAGCTTGGCCACGAGGTGGTGAAC------------GACAAT---------

------GCCGACGATCTCAATAAGCGACTGAAGACCAAAATAAATCCCACCACCCATATG

TACACGCATTGCGAAATCCATCCTAGTATGCTGCTTGGCATTTGCGCTAGCATTATTCCC

TTCCCCGATCACAATCAGGTATGCCAACGTCATGTAA-----------------------

-----------------------------------

>Obolarina_dryophila_MUCL_49882

------------------------------------------------------------

--------GACGCAGGAGGTGCTGTCGCAGATGAAACGCAGTATCGAGCAGAACCGGGGC

TTCGCCATCGAATTGGCCGTGAAGCCGACTATCATCACCAACGGTCTGAAGTACTCGCTC

GCCACTGGCAACTGGGGTGATCAGAAGAAAGCCATGAGCTCGACGGCGGGTGTCTCACAG

GTGTTGAACCGATATACATTCTCCTCTACCCTCTCTCACTTGAGACGCACGAATACTCCG

GTCGGAAGAGATGGCAAGCTCGCCAAGCCACGTCAACTTCACAACACCCACTGGGGTCTC

GTCTGTCCTGCAGAGACCCCTGAAGGTCAAGCCTGTGGTCTAGTGAAGAACTTGTCATTG

ATGTGCTCTATCAGTGTCGGCACATCAACAGAGCCCATCATTGATTATATGATTACTCGT

AATATGGAGGTGCTTGAAGAGTACGAGCCTATGCGATACCCTCACGCCACTAAGATCTTC

CTCAACGGTTCTTGGATCGGTGTTCATCAAGACCCCAAGGCGCTGGTCAGGGATGTCCAG

CAACTGCGTCGAAGCAATCAGATCCCAGCTGAGGTGTCCCTAGTGCGTGACATTCGAGAC

CGTGAGTTCAAGATCTTTTCAGATGCCGGTCGAGTCATGAGACCCTTGTTCGTTGTCGAG

CAGGAGGACGTGGCTGAGACCGGTGTTGAGAAGGGGACGCTGGCTCTCAACAAGAGTATG

GTCAGG------------AGACTTGAGCTCGACCAGACCCTCCCCCCTGGAAGC------

---GAGGAG---TATTATGGCTGGCCGGGTTTGGTGAACGATGGTGTCATTGAATACCTT

GATGCGGAAGAGGAAGAGACGGCCATGATATGTATGACACCGGAAGATCTCGAGATCTAC

CGGATGACGAAGCTTGGCCATGAGGTTGTGAAC------------GACAAC---------

------GCGGACGATCTCAACAAGCGACTGAAGACAAAAATGAATCCAACCACCCATATG

TACACGCATTGCGAAATCCACCCTAGTATGCTGCTTGGCATTTGCGCTAGCATTATTCCT

TTCCCCGATCACAATCAGGTATGCTAATACTATTTAATGTC---TATACTAATC------

-----------------------------------

>Biscogniauxia_marginata_MFLUCC_12_0740

------------------------------------------------------------

------------------------------------------------------------

------------------------------------------------------------

-----------------------------------------CACGGCAGGTGTCTCGCAG

GTGCTAAACCGATACACGTTTTCCTCTACTCTCTCTCACTTGAGGCGTACGAACACTCCT

GTAGGAAGAGATGGCAAGCTCGCCAAGCCACGACAACTTCATAATACCCACTGGGGCCTC

GTCTGTCCGGCAGAGACGCCCGAGGGTCAAGCTTGTGGCCTAGTGAAGAACCTGTCATTG

ATGTGCTCTATCAGCGTGGGCACGTCCACGGAGCCCATTATTGATTACATGATTACTCGA

AACATGGAAGTGCTTGAAGAGTATGAGCCCATGCGATATCCTCATGCCACTAAGATTTTC

CTCAACGGCTCTTGGATTGGTGTCCACCAGGACCCGAAGGCGCTCGTTAGGGATGTCCAG

CAATTGCGTCGGAGCAACCAGATTCCAGCGGAGGTGTCCCTAGTTCGCGACATCCGAGAC

CGCGAGTTTAAGATCTTCTCAGATGCTGGTCGAGTCATGCGGCCCTTGTTTGTGGTCGAG

CAAGAGGACGTGGCTGAGACTGGTGTTGAGAAGGGATCACTGGCTCTCAACAAGGACATG

GTGAGA------------AGATTAGAAAACGACCAAACGCTCCCCCCTGGAAGC------

---GAAGAG---TACTATGGATGGCAAGGTTTGGTAAACGATGGTGTCATCGAATACCTC

GATGCCGAAGAGGAAGAGACTGCTATGATATGCATGACGCCGGAAGATCTCGAGATCTAT

CGGATGACTAAGCTTGGCCATGAGATGGTGAAT------------GACAAC---------

------GGAGACGACCTTAATAAGCGACTCAAGACAAAGATAAATCCAACCACACACATG

TACACGCATTGCGAGATCCACCCTAGTATGCTTCTGGGTATCTGCGC-------------

------------------------------------------------------------

-----------------------------------

>Graphostroma_platystoma_CBS_270.87

------------------------------------------------------------

---------------------------------AAGCGGAGTATCGAACAGAACCGGGGT

TTCGCTATCGAGCTAGCTGTGAAGCCGACTATCATTACCAACGGTCTGAAGTACTCGCTC

GCCACCGGCAACTGGGGTGACCAGAAGAAAGCCATGAGCTCCACAGCTGGTGTTTCGCAA

GTGCTAAACCGATACACATTTTCCTCGACGCTCTCTCACTTGAGACGTACAAACACTCCC

GTAGGGAGAGATGGCAAGCTCGCCAAGCCACGGCAACTTCATAATACTCACTGGGGTCTT

GTCTGTCCCGCCGAAACTCCCGAAGGCCAAGCTTGTGGCCTGGTGAAGAACCTGTCATTG

ATGTGCTCCATCAGCGTGGGCACATCGACAGAGCCCATTATCGATTACATGATTACCCGA

AACATGGAAGTGCTCGAGGAATACGAGCCCATGCGATATCCCCACGCCACCAAGATCTTC

CTCAACGGCTCTTGGATTGGTGTTCACCAGGACCCGAAGGCGCTCGTCAGGGATGTTCAG

CAACTGCGCCGGAGCAATCAGATTCCAGCAGAGGTGTCCCTAGTTCGCGACATCCGAGAC

CGCGAGTTTAAGATTTTCTCGGACGCTGGTCGCGTCATGCGACCCTTGTTTGTTGTCGAA

CAGGAGAGCGTGCCCGAGACGGGTGTCGAGAAGGGATCACTAGCTCTCAACAAGGACATG

GTGAGA------------CGACTTGAAATCGACCAAACGCTCCCTCCTGGAAGC------

---GAAGAG---TACTACGGCTGGCAAGGTTTGGTAAACGACGGTGTCATTGAATACCTT

GATGCCGAGGAGGAAGAGACGGCTATGATATGCATGACGCCAGAAGATCTCGAGATCTAT

CGGAGGACCAAGCTTGGTGAAGAGATTGTGAAC------------GACAAC---------

------GGAGACGATCTTAATAAGCGACTCAAGACAAAAATAAATCCAACCACGCACATG

TACACGCATTGCGAAATTCATCCTAGTATGCTTCTGGGTATCTGCGCCAGCATCATCCCC

TTCCCTGATCACA-----------------------------------------------

-----------------------------------

>Astrocystis_concavispora_MFLUCC_14.0174

------------------------------------------------------------

------------------------------------------------------------

------------------------------------------------------------

----------------------------------TGAGCTCTACCGCCGGTGTGGCTCAG

GTCTTGAACAGATATACTTTCGCCTCCACACTATCGCATTTACGAAGAACCAACACACCT

GTGGGTAGAGATGGCAAACTTGCCAAGCCGCGGCAGCTTCTCGCATGCCCCTGGGGTCTG

GTCTGCCCGGCTGAGACGCCGGAAGGTCAGGCTTGCGGCCTAGTCAAGAATCTGTCCCTC

ATGTGCTCCATCAGTGTCGGCACATCGACCGAGCCCATCATAGAATACATGATCTCGCGG

AATATGGAGGTTGCGGAAGAGTATGAACCTGCGAGATACCCGAATGCCACCAGCATAATC

CTCAATGGGTCGTGGATTGGTATTCATCAAGATCCAAAGTCATTGGTCCGGGACGTTCAA

CAGTTGCGGCGCACGAACCAGATCCCGGCCGAGGTTTCGCTGATTAGGGACATCCGTGAC

CGTGAGTTCAAGATCTTCTCGGATGCTGGCCGAGTCATGCGGCCCTTGTTCGTAGTCGAG

AACGACCCCGAGCCCGACAATGGTGTCCAGCAGGGACAGCTAGTCTTGACGAAAGACATG

GTTCGA------------CGACTTGAGATCGACCAGACCCTTCCACCAGGAAGC------

---GAAGAA---TATTTCGGATGGCAAGGCCTGGTCAATGCCGGTGTCATCGAATACATG

GATGCCGAGGAAGAGGAGACAGCCATGATCTGCATGACACCCGAAGATCTGGAGGCATTC

AGATTGACCAAATTAGGCCAGTACGACCCCGAAGCCGAG------AACAAT---------

------CTGCTGGAGCCGAACAAGCGACTCAAGACACGTATGAACCCCACGACACACACT

TACACCCACTGCGAAATCCACCCAAGCATGCTTCTCGGTATCTGCGCCAGCATTATCCCT

TTCCCCGATCATAACCAGGTACGTGTCATGCAATCCTTCCCATCGCAATTACTGATCCAA

TCTCTAGTCACCCCGTAATACATATCAATCCGCC-

>Rhopalostroma_angolense_CBS_126414

------------------------------------------------------------

------------------------------------------------------------

------------------------------------------------------------

------------------------------------------------------------

--TTTTGACCGATACACGTTCGCGTCAACTCTCTCCCATTTAAGGCGAACGAACACGCCT

ATTGGAAGAGACGGGAAGCTCGCGAAACCTCGACAGCTGCACAATACCCACTGGGGTCTG

GTCTGTCCGGCCGAAACGCCCGAAGGCCAGGCTTGCGGTCTGGTGAAGAATCTGTCGCTG

ATGTGCTCCATCAGCGTGGGTACCTCAACGGATCCCATCGTAGACTATATGATTACTAGG

AATATGGAAGTCTTGGAGGAATATGAGCCGATGCGATACCCTAACGCTACCAAGATCTTC

CTCAACGGATCGTGGATCGGCGTGCACCAGGATCCCAAGTCTCTCGTCCGAGACGTCCAG

CAGCTTCGTCGGGCTAACCAAATCCCCTCCGAAGTGTCCCTCGTTCGCGATATTCGTGAT

CGCGAGTTCAAGATCTTTTCGGACGCAGGCCGTGTCATGCGGCCCTTGTTCGTGGTGCAG

CAAGAGGACGATCCCGATGCCGGTATCACGAAAGGGTCGCTGGCCCTTACCAAGGAGATG

ATCCAG------------AGGCTGGAGGCGAGTGTCGATCTCGACCCGGAGAGC------

---GAGGAG---TACTTTGGTTGGCAAAGTCTTGTTAACGAGGGCGTTATCGAGTACCTC

GACGCGGAGGAGGAAGAAACGGCCATGATTTGTATGACACCCGAAGATTTGGAAACCTAT

CGGATGTCCAAGCTTGGGTACGACGTGTCTCAG------------GACAAC---------

------GGAGATGAGATCAACAAGCGGCTCAAGACCAAGTTGAATCCCACGACGCACATG

TACACGCATTGCGAGATCCATCCTAGCATGCTCCTGGGTATCTGCGCGAGCATCATCCCC

TTCCCCGACCACAACCAGGTACGTATTATTACTATCTATCGTTCCGTTGCTAACCTGTCG

CC--TAGT---------------------------

>Annulohypoxylon_truncatum_CBS_140778

--------------------------------------------------AAATATTGTT

CGCCGACTAGTGCAAGAGATAACTCAGCATCTCAAGCGCTGCATCGACGGGAACAAACGT

TTCCAGATTGAACTTGCCGCCAAGCCCGCCATTATCACCAACGGCTTGAAGTACTCGCTC

GCTACGGGTAACTGGGGCGATCAAAAGAAGGCGATGAGCTCGACTGCCGGTGTATCGCAG

GTCTTGAACCGCTACACTTTCTCGTCAACCCTTTCTCATTTACGACGAACGAACACGCCC

ATCGGAAGAGATGGAAAGCTAGCCAAGCCACGGCAGCTCCACAACACACACTGGGGTCTC

GTCTGTCCGGCAGAGACTCCCGAAGGCCAGGCTTGTGGCTTGGTGAAGAATCTATCCTTG

ATGTGTTCTATCAGTGTTGGAACATCGACAGATCCTATCGTGGACTACATGATCACTAGG

AATATGGAAGTCCTGGAGGAGTATGAACCGATGAGATATCCCAACGCCACTAAGATCTTC

CTCAACGGCTCCTGGATCGGTGTACACCAGGACCCAAAGACCCTCGTCAGGGACGTCCAG

GCACTTCGTCGAGCCAACCAGATACCTGCTGAGGTCTCGCTAGTTCGTGATATCCGAGAC

CGTGAATTCAAGATCTTTTCGGATGCCGGTCGTGTGATGCGCCCCCTGTTCCGCGTACAA

CAGGAAGACATCGCCGAGCAGGGCATCGAGAAGGGCACCTTGGCTCTTACCAAGCAGATG

ATCAAA------------CGTCTAGAAGCAGATGTCGATCTGGACCCGGAGAGC------

---GAGGCG---TACTATGGCTGGCAAGGTCTAGTCAACGAGGGAGTTATCGAGTTCCTC

GATGCGGAAGAAGAGGAGACTGCGATGATTTGCATGACGCCGGAAGATTTGGACACCTAC

CGTATGACCAAGCTTGGGTATGAGGTGTCCCAG------------GACAAC---------

------GGAGATGAGGTGAATAAGCGACTCAAGACTAAAATCAACCCGTCAACGCACATG

TATACCCATTGCGAGATCCATCCCAGTATGCTCCTGGGTATCTGCGCAAGCATCATTCCA

TTCCCAGATCATAACCAGGTATGTGATGTA---TGTTGCTTTTCGCTACTAACCA-----

-----------------------------------

>Hypoxylon_fragiforme_MUCL_51264

------------------------------------------CTATTCCGGAATATTGTT

CGTCGATTGGTCCAGGAGATTACGCAGCATCTGAAGCGTTGTATCGATTCGAATCGACGT

TTCCAAATCGAGCTTGCCGCCAAACCTGCCATCATCACCAACGGTCTGAAGTACTCCCTC

GCCACAGGTAACTGGGGCGACCAAAAGAAGGCGATGAGCTCGACTGCCGGTGTCTCCCAG

GTCCTGAACCGATATACTTTCGCCTCGACCCTCTCTCACTTGAGACGGACGAACACTCCC

ATCGGAAGAGACGGGAAGCTTGCGAAGCCTCGACAGCTTCACAATACTCATTGGGGTCTT

GTCTGTCCAGCTGAGACGCCCGAAGGCCAGGCCTGTGGACTGGTGAAGAACTTGTCGCTG

ATGTGCTCCATCAGCGTGGGTACATCGACGGATCCTATCGTGGAGTATATGATTACGAGA

AATATGGAAGTCCTGGAGGAATACGAACCGATGCGATACCCTAATGCCACCAAGATCTTC

CTTAACGGTTCTTGGATTGGTGTACACCAGGATCCCAAGACTCTTGTCAAGGATATCCAG

GCGCTTCGTCGGGCCAACCAGATTCCCTCCGAGGTTTCCTTGATCCGCGATATCCGTGAT

CGTGAGTTCAAGATATTCTCAGACGCAGGTCGTGTCATGCGCCCCTTGTTTGTCGTGCAA

CAAGAAGATAATCCCGATCAAGGCATTGCCAAGGGTACATTGGCCCTTACCAAAGAGATG

ATCCAG------------CGACTAGAAGCGGATGTTGATCTTGATCCTGAAAGC------

---GAGGAG---TACTTTGGCTGGCAAGGTCTCGTTAACGAGGGTGTAATCGAGTTTCTC

GACGCGGAGGAAGAGGAAACGGCTATGATTTGCATGACACCCGAAGACCTGGAAAACTAC

CGGTTGACCAAGCTCGGATTT---------------------------------------

------------------------------------------------------------

------------------------------------------------------------

------------------------------------------------------------

-----------------------------------

>Hypocreodendron_sanguineum_J.D.R._169

GGCAAGAAGCGACTGGACCTCGCTGGGCCTCTACTCGCTAAGCTTTTCCGCAACATCGTA

CGACGAATGACACAAGAAGTCTTGTCACATCTAAAGCGAAGTATCGAGCAAGGAAAGCAA

TTCAATATCGCCCTAGCTGTAAAGTCAAATATCATTACGAGCGGATTAAAATATTCTCTC

GCTACTGGAAATTGGGGCGATCAAAAGAAGGCCATGAGCTCTACCGCCGGTGTTTCACAG

GTGCTGAACCGATACACATTTGCATCTACTTTGTCACATTTGCGACGAACAAACACCCCA

GTCGGACGGGATGGAAAGCTGGCGAAGCCACGTCAACTTCACAATACCCACTGGGGTCTG

GTTTGTCCTGCCGAGACGCCCGAAGGCCAGGCTTGCGGCCTAGTCAAGAATCTGTCCTTG

ATGTGCTCCATCAGTGTTGGCACTTCTACCGAACCCATTATAGATTACATGACGTTCCGC

AATATGGAGGTTTTGGAAGAATATGAGCCCCTAAGATATCCACATGCTACCAAAATCTTC

CTCAACGGGTCCTGGGTCGGTGTCCATCAAGACCCGAAGGCTTTGGTCAAGGACGTCCAA

CAGCTACGCCGAACAAACCAGATTCCGGCTGAGGTGTCTTTAATCCGGGATATTCGCGAT

CGCGAGTTCAAAATCTTTTCGGACGCAGGCCGTGTCATGCGGCCTCTGTTTGTGGTTGAG

CAAGAAGATGACCCAGATAATGGTGTTCAAAAGGGCTCACTGGTATTGACAAAAGACATG

GTACGC------------AGGCTCGAGATTGATCAGACGCTACCGCCTGGAAGT------

---GATGAG---TATTTTGGGTGGCAGGGTCTTGTCAATGCCGGTGTGATTGAATACATG

GACGCCGAAGAAGAAGAGACTGCGATGATTTGCATGACACCTGAAGACCTGGAGGCCTAT

CGGCTGACCAAGTTGGGCTTTAAAAATACAGACGACGAC------CCCGAC---------

------TCCGAAACGCCTAACAAACGCTTGAAGACTAAGATCAATCCTACAACACATACA

TACACTCATTGTGAAATTCATCCCAGCATGCTGCTTGGTATCTGCGCAAGCATCATTCCT

TTCCCTGATCATAATCAAGTAAGTGGCAGGTGATTTTTGTCTTTATTGTCACTAATATAT

ATTCTAGTCCCCCAGAAATACCTACCAGTCTGCTA

>Thamnomyces_dendroidea_CBS_123578

------------------------------------------------------------

------------------------------------------------------------

------------------------------------------------------------

------------------------------------------------------------

---------------ACTTTCTCGTCGACTCTTTCCCATCTAAGGCGAACCAACACGCCT

ATCGGAAGAGACGGGAAGCTCGCGAAACCTCGACAGCTGCACAATACCCACTGGGGTCTT

GTCTGTCCGGCCGAAACGCCCGAAGGCCAGGCCTGCGGTCTGGTGAAGAACCTATCGCTT

ATGTGCTCCATCAGCGTGGGTACCTCGACGGATCCTATCGTAGACTACATGATTACTAGG

AATATGGAAGTCTTAGAGGAATACGAGCCGATGCGATACCCTAACGCCACCAAGATCTTC

CTCAACGGATCCTGGATCGGTGTGCATCAGGATCCCAAGTCTCTCGTCAGAGATGTCCAG

CAGCTTCGTCGGGCTAACCAAATCCCCTCCGAAGTATCTCTCGTTCGTGATATCCGTGAT

CGCGAGTTCAAGATCTTTTCGGACGCCGGTCGTGTCATGCGGCCCTTGTTCGTGGTGCAG

CAAGAGGATGATCCCGAGGCTGGTATCACGAAGGGCTCGCTGGCTCTTACCAAGGAAATG

ATCCAG------------AGGTTGGAGGCGAGTGTTGATGTCGACGCGGAGAGC------

---GAAGAG---TACTTTGGCTGGCAAAGTCTTGTCAACGCGGGTGTTATCGAGTACCTC

GACGCGGAGGAGGAAGAAACGGCCATGATTTGCATGACCCCCGAAGATTTAGAAACCTAC

CGGATGTCTAAACTCGGATATGATGTGTCTCAG------------GACAAC---------

------GGGGACGAGATTAACAAGCGGCTCAAGACCAAGTTGAATCTTACGACGCACATG

TACACGCATTGCGAGATCCATCCCAGCATGCTCCTGGGTATCTGCGCGAGCATCATCCCT

TTCCCCGATCACAATCAGGTATGTAATTCTAATATCAAT--ATCCGCTGCTAAC---TCT

ATAC-------------------------------

>Pyrenopolyporus_hunteri_MUCL_52673

------------------------------------------------------------

---------------------------------------------------AACAGACGC

TTCCAAATCGAGCTTGCTGCCAAGCCGGCCATAATCACCAATGGTTTGAAATATTCTCTA

GCCACAGGCAACTGGGGCGACCAGAAGAAAGCCATGAGCTCCACGGCCGGCGTGTCGCAG

GTCCTAAACAGATATACGTTCGCCTCGACCCTTTCCCATTTACGACGAACGAACACGCCC

ATCGGAAGGGACGGCAAGCTCGCGAAGCCGCGACAGCTACACAACACTCATTGGGGTCTG

GTATGTCCGGCCGAGACGCCCGAAGGCCAAGCCTGCGGGCTGGTCAAGAATCTGTCGCTT

ATGTGCTCCATCAGCGTGGGTACCTCAACGGATCCTATCGTAGATTATATGATCACCAGA

AACATGGAGGTCTTGGAGGAATATGAGCCCATGAGATATCCTAACGCAACCAAGATCTTC

CTCAACGGCTCCTGGATCGGTGTGCACCAAGATCCCAAGTCTCTAGTTAGAGACGTTCAG

CAGCTGCGCCGGGCCAACCAGATTCCCTCCGAGGTATCTTTAGTTCGCGACATCCGAGAC

CGCGAGTTCAAGATTTTCTCAGACGCCGGCCGCGTCATGCGTCCCTTGTTTGTTGTACAG

CAAGAGGATGACCCGGACACCGGTGTCCCCAAGGGCCACCTGGCTCTCACGAAGACCCAG

ATTGCG------------AAGCTGGAGGCAAGCATCGACGTAGAGGTCGACGCT------

---CCCGGC---TACTATGGCTGGCAAGGGTTAGTTAACGACGGTGTTATCGAGTATCTC

GATGCGGAGGAGGAGGAGACGGCTATGATATGCATGACGCCCGAAGACTTGGAAACATAT

CGCATGGCCAAGGCCGGCATTGATATGCCTCAG------------GACAAC---------

------GGGGACGAGATCAACAAGCGCCTCAAGACCAAGGTTAACCCCACGACGCACATG

TACACGCACTGCGAGATCCACCCGAGTATGCTTCTAGGTATTTGCGCTAGCATTATTCCC

TTCCCAGACCATAATCAGGTACGTAATTCTCCCCTTCGG---------------------

-----------------------------------

>Rostrohypoxylon_terebratum_CBS_119137

-------------------------------------------TGTTTAGGAATATAATT

CGTCGGCTGGTGGCTGAGACTACACAGCATCTCAAACGCTGCATTGACAGTAACAAGCGT

TTCCAAATCGAGCTTGCCGCTAAGCCAGGCATCATCACCAACGGTTTGAAGTACTCGCTC

GCCACGGGTAATTGGGGCGATCAGAAGAAGGCGATGAACTCGACCGCCGGCGTGTCACAG

GTCTTGAACAGGTATACTTTCGCGTCAACCCTTTCTCACTTGAGACGAACCAACACTCCC

ATCGGAAGAGACGGAAAGCTAGCTAAACCACGGCAGCTTCACAACACTCATTGGGGTCTG

GTCTGTCCAGCAGAGACGCCCGAAGGCCAGGCTTGCGGATTGGTGAAGAACTTGTCCTTG

ATGTGTTCCATCAGTGTTGGTACATCGACAGATCCCATTGTCGACTACATGATAACCAGG

AACATGGAAGTTCTTGAGGAGTATGAACCCATGAGATATCCCAACGCCACCAAGATCTTC

CTCAACGGCTCTTGGATCGGTGTTCATCAAGACCCTCAATCCCTCGTCAGAGATATTCAG

ACACTTCGCCGGGCCAACCAGATTCCCGCTGAGGTATCGTTAATTCGTAATATTCGAGAC

CGTGAATTCAAGATCTTCTCGGATGCTGGCCGTGTGATGCGTCCCCTCTTCCGTGTACAA

CAGGAGACCGACGAGGCGCAGGGCATTGAGAAGGGCACATTAGCCCTTACCAAACAGATG

ATTAAG------------CGCCTAGAAGCGGATGTCGAGTTGGATCCAGAGAGT------

---GAAGAA---TACTTCGGCTGGCAAGGTCTAGTCAACGAAGGTGTTATCGAGTACCTC

GACGCGGAGGAAGAGGAAACGGCCATGATTTGCATGACGCCTGAAGACTTGGACATTTAT

CGCATGA-----------------------------------------------------

------------------------------------------------------------

------------------------------------------------------------

------------------------------------------------------------

-----------------------------------

# *tub2*

>Lopadostoma_turgidum_CBS_133207

CTCTGTTTACTT----GCCTTCTTGGAGCTGCTGCAGGCTTCACGG-------CTAGAGC

GCGTCCCAGTCAAT-GCCCCTGACCCCCGCTGCCCCTC----------------CACCTT

CACT-----------------GCCCCCGGGATC--GACACCTTCGAGT--------TCAT

CTGAAGAAAACCCGAT---GAAACGCAGCTAACCGTGTCTCTCGTGCCTATAGGTTCACC

TCCAGACCGGCCAATGCGTAAGTAACATCCCGACAACAACCTTTCGAGATGGGCGAATTG

CGGCGCTCACATGAAATCTTAGGGTAACCAAATCGGTGCTGCTTTCTGGTGCGTATTACC

TCCACGAGACACGATCAGTGATGAGCAAGATGCTGCATCTGATCCAGGCAAACCATCTCT

GGCGAGCACGGTCTCGACAGCAACGGTGTGTATGTAGCACTATCACCCACCCCTGTGC--

TTGTATGACTCTAGGCTTACCACTTCCAAACAGCTACAATGGCACCTCCGAGCTCCAGCT

GGAGCGTATGAGCGTCTACTTCAACGAGGTAATGTGTT----GAAT--CACCAGACCTAA

TATCCAGTTTCTAACCTTAATTCGTGATGTAGGCCTCGGGCAACAAATATGTCCCCCGCG

CTGTCCTCGTCGATCTCGAGCCCGGTACCATGGACGCCGTCCGTGCCGGTCCCTTCGGTC

AGCTCTTCCGCCCCGACAACTTCGTCTTCGGCCAGTCCGGTGCTGGAAACAACTGGGCCA

AGGGTCACTACACCGAGGGTGCCGAGCTCGTCGACCAGGTTCTCGATGTCGTCCGTCGTG

AGGCTGAGGGCTGCGACTGCCTCCAGGGCTTCCAGATCACCCACTCCCTTGGTGGTGGTA

CCGGTGCTGGTATGGGCACCCTGTTGATCTCCAAGATCCGCGAGGAGTTCCCCGACCGCA

TGATGGCCACCTTCTCCGTCGTCCCCTCCCCCAAGGTTTCCGACACCGTCGTCGAGCCTT

ACAACGCCACCCTCTCCGTCCACCAGCTGGTCGAGAACTCCGACGAGACTTTCTGTATCG

ACAACGAGGCTCTGTACGATATTTGCATGCGTACCCTGAAGCTGTCCAACCCCTCCTACG

GCGACCTGAACCACCTCGTCTCCGCTGTCATGTCCGGCGTCACCACCTGCTTGCGTTTCC

CCGGCCAGCTGAACTCTGACCTGCGCAAGCTGGCTGTCAACATGGTTCCCTTCCCCCGTC

TTCACTTCTTCATGGTCGGCTTCGCTCCCCTCACTAGCCGCGGTGCTCACTCTTTCCGTG

CCGTCACCGTCCCTGAGTTGACCCAGCAGATGTTCGACCCCAAGAACATGATGGCTGCCT

CCGACTTCCGCAACGGTCGTTACCTGACTTGCTCCGCTATCTTGTAAGCTCACACAAAAA

CCCGCCTTTTTTCCCCCCATTGCTAACTTCA----TCGTTTAGCCGCGGTAAGGTCTCTA

TGAAGGAGGTTGAGGACCAGATGCGCAATGT

>Creosphaeria_sassafras_ST.MA._14087

------------------CTG--CAACGCTGCA---GCATTCAGGGCAAGACCCTGAATC

GCGTCTCGCTCAATGCCCCTTGACCTCCGCTTCTCCTCCTCTCCAC--------CATATT

CACT-----------------GCCCTCGAGATCACGACGTTTTCGAGT--------TCAT

GTGAAAGAAC------AAGGAATCACAACTAACCGCTTTTCTCTTATCTATAGGTTCATC

TTCAGACCGGCCAATGCGTAAGTAACATCCCGACAAGCGTCCTTTGCGCTGTGAA-ACCG

TGGGGCTCACACGATATGACAGGGTAACCAAATTGGTGCTGCTTTCTGGTGCGTACCGCG

TCCATGAGACGCGAT-AACGACGAGCAGGACATTGCGTCTGATTTAGGCAAACCATCTCT

GGCGAGCATGGCCTCGACAGCAACGGTGTGTATGTAGCTATAGCCCATACCCCTTTACTT

TTGTATGACCCCAGGCTTACCA-CCTCCAACAGCTATAATGGCACCTCCGAGCTCCAGCT

GGAGCGGATGAAAGTGTACTTTAACGAGGTAATGTGTT----GAAC--CAGCAGACCTAA

TATCCAGTTTCTAACCTTGACCCGTGATGTAGGCCTCTGGCAACAAATATGTTCCCCGCG

CTGTCCTTGTCGATCTCGAGCCCGGTACCATGGATGCTGTCCGTTCCGGTCCTTTTGGCC

AGCTTTTCCGTCCCGACAACTTCGTCTTCGGCCAGTCCGGTGCCGGAAACAACTGGGCCA

AGGGTCATTACACCGAGGGTGCTGAGCTTGTCGACCAAGTTCTCGATGTCGTCCGTCGTG

AGGCTGAGGGTTGCGACTGCCTCCAGGGTTTCCAGATCACCCACTCGCTCGGTGGTGGTA

CCGGTGCCGGTATGGGTACACTGCTGATCTCCAAGATCCGTGAGGAGTTTCCCGACCGCA

TGATGGCCACCTTCTCCGTCGTCCCGTCTCCCAAGGTTTCCGACACCGTCGTTGAGCCCT

ACAACGCCACCCTGTCCGTCCACCAGCTTGTTGAGAACTCCGACGAGACCTTCTGCATTG

ACAACGAAGCTCTGTACGACATCTGTATGCGTACCCTGAAGCTGTCCAACCCTTCCTATG

GCGACCTGAACCACCTCGTCTCCGCTGTCATGTCCGGCGTCACTACCTGTCTGCGTTTCC

CTGGCCAGCTGAACTCTGACTTGCGCAAGCTGGCTGTCAACATGGTTCCCTTCCCCCGTC

TTCATTTCTTCATGGTCGGCTTTGCTCCCCTCACCAGCCGCGGCGCTCACTCTTTCCGTG

CTGTCACCGTCCCGGAGTTGACACAGCAGATGTTCGACCCCAAGAACATGATGGCTGCTT

CCGACTTCCGTAACGGTCGCTACCTGACTTGCTCCGCTATCTTGTAAGCCTACGCGAAAA

GCAGCTCAACCATTTTCTCCTGCTAACTTC------------------------------

-------------------------------

>Diatrype_disciformis_CBS_197.49

------------------------------------------------------------

------------------------------------------------------------

------------------------------------------------------------

------------------------------------------------------------

------------------------------------------------------------

------------------------------------------------------------

------------------------------------------------------------

------------------------------------------------------------

------------------------------------------------------------

------------------------------------------------------------

------------------------------------------------------------

------------------------------------------------------------

------------------------------------------------------------

------------------------------------------------------------

------------------------------------------------------------

------------------------------------------------------------

------------------------------------------------------------

------------------------------------------------------------

------------------------------------------------------------

------------------------------------------------------------

------------------------------------------------------------

------------------------------------------------------------

------------------------------------------------------------

------------------------------------------------------------

------------------------------------------------------------

-------------------------------

>Eutypa_lata_UCR_EL1

CTCTTTCTGTGTTTACTACCAGACAA-GCCCTG-AA---------------CCCTACAAC

GCGTCCAACCTCATGTTGCCTGATCCAAA-TCCACCAAAATTCTGCATCTGCATTATCTC

CAGTCTCCTATCT----GCT-TCTTGCTATGTCGCGGCTCA----AATCTACTGGC---T

CTCCAA----AATATCATTAAAATAATACTAACCTCGTCTTTCGTCAATATAGGTTCACC

TTCAGACCGGCCAATGCGTAAGTGCCTC----ACTTCTC-GAGTATCGACCATTACATTG

CGGGGCTCATAGAAATCAATAGGGTAACCAAATTGGTGCTGCTTTCT-------------

----------------------------------------------GGCAAACCATCTCT

GGCGAGCATGGTCTCGACAGCAACGGTGTGTATGTAACCGATTTTTAATTACCTAGATAA

TCGT---GTGATTGTGTGATTTACACCCAATAGCTACAATGGCACCTCCGAGCTCCAGCT

TGAGCGCATTAACGTCTACTTCAACGAGGTAAGCAATTGCTAACTCGCCGCTGTTTGGCT

TTGCTAACAAATTTTTTCTTTTCCAACTGTAGGCGTCCGGCAACAAGTATGTTCCTCGTG

CCGTTCTTGTCGATCTCGAGCCCGGTACCATGGATGCCGTCCGTGCTGGTCCCTTCGGTC

AGCTTTTCCGTCCCGACAACTTCGTCTTTGGACAATCCGGTGCTGGCAACAACTGGGCCA

AGGGTCACTACACTGAGGGTGCTGAGCTTGTTGACCAGGTTCTTGACGTTGTCCGTCGTG

AGGCCGAGGGTTGCGACTGCCTCCAGGGCTTCCAGATCACCCACTCTCTCGGTGGTGGTA

CCGGTGCCGGTATGGGTACCTTGTTGATCTCCAAGATCCGCGAAGAGTTCCCTGACCGTA

TGATGGCCACTTTCTCCGTTGTGCCCTCTCCCAAGGTTTCCGACACTGTCGTTGAGCCTT

ACAACGCCACCCTTTCCGTCCACCAGCTGGTTGAGAACTCAGACGAGACCTTCTGCATTG

ACAACGAGGCTCTCTACGACATCTGCATGCGTACCCTGAAGCTGTCCAACCCTTCGTATG

GTGACCTGAACCACCTCGTCTCCGCCGTCATGTCTGGTGTCTCCACCTCCCTGCGTTTCC

CCGGTCAGCTGAACTCTGATCTGCGCAAGCTGGCCGTCAACATGGTCCCCTTCCCCCGTC

TCCACTTCTTCATGGTCGGCTTCGCTCCTCTGACCAGCCGTGGTGCCCACTCTTTCCGCG

CTGTCACCGTTCCCGAGTTGACCCAGCAGATGTTCGACCCCAAGAACATGATGGCTGCTT

CTGACTTCCGTAACGGTCGTTACCTGACCTGCTCTGCCATCTTGTAAGTCTAACACGTTA

CTACTCATTTTATTAATACTTGCTAACGTCA-----CATGTAGCCGTGGTAAGGTCTCCA

TGAAGGAGGTCGAGGACCAGATGCGCAATGT

>Entoleuca_mammata_J.D.R._100

CTCTGTTTACTTTGCAACCCAATCAA-GCTTCGTGATTTTTTGCTACCCTATCCCTGAAA

ACGCTCC-----ACGCCCCTCGATCGTGGTCTGTCCACATCGATACGCAAGCATCATGCG

CGATCACTGATGC----TATGATCGTCGATGATGCTACTTCGTTGGGTCCCCTGGGACG-

TTATCGAAACTGTTGCTTTAGAGTTAAGCTAACGATACCTTCCCCGTGTCTAGGTTCACC

TCCAAACCGGCCAATGCGTAAGCTTCCT--CGATGCTTCGCGATGACGGCGAGCATCTCG

TGGACTCACAATGAAATCATAGGGTAACCAAATCGGTGCTGCTTTCT-------------

----------------------------------------------GGCAACAAATTTCG

GGCGAGCACGGCCTTGACGGCAATGGCGTGTATGTTCTTGACGTCCATTGAGTCTGGGGA

TACCAGAATAATTGACTAATATGCGTGGGACAGCTACAACGGAACGTCCGAGCTTCAGCT

CGAGCGTATGAGCGTCTACTTCAACGAGGTACGCAAACACCAGTTCATCGG-GAGTTGTG

CCATCGATATTCTAACGTGTT-GAATTTTTAGGGTGCTGGCAACAAGTATGTCCCCCGCG

CCGTTCTCGTCGATTTGGAGCCCGGTACCATGGATGCTGTCCGCGCCGGTCCCTTCGGTC

AGCTCTTCCGACCCGACAACTTCGTCTTCGGTCAGTCAGGTGCTGGCAACAACTGGGCCA

AGGGTCACTACACGGAGGGTGCCGAGCTGGTCGACACCGTTCTCGATGTCGTCCGTCGCG

AGGCTGAGGGCTGCGACTGCCTTCAAGGTTTCCAGATCACTCACTCCCTCGGCGGTGGTA

CTGGTGCCGGTATGGGTACGCTGTTGATCTCCAAGATCCGCGAAGAGTTCCCCGACCGCA

TGATGGCTACCTTCTCTGTTATGCCCTCTCCCAAGGTCTCCGACACCGTTGTTGAGCCCT

ACAACGCCACCCTCTCCGTCCACCAGCTGGTCGAGAACTCCGACGAGACCTTCTGTATCG

ATAACGAAGCTCTGTACGATATCTGCATGCGTACACTGAAGCTATCCAACCCCTCATACG

GTGACCTGAACCACCTGGTCTCCGCCGTCATGTCTGGTGTCACCACCTGCCTGCGTTTCC

CTGGTCAGCTTAACTCTGACCTGCGCAAGTTGGCTGTGAACATGGTGCCGTTCCCCCGTC

TGCACTTCTTCATGGTCGGCTTTGCTCCTCTTACCAGCCGTGGTGCCCACTCTTTCCGTG

CCGTCACGGTTCCTGAGTTGACTCAACAAATGTTCGACCCCAAGAACATGATGGCCGCCG

CTGACTTCCGCAACGGTCGCTACCTAACATGCTCTGCCATCCTGTAAGAATCTAACCCTT

CC-CCCTCGACACCATCCGTTACTAATCTAACAT-TTCAATAGCCGTGGTAAGGTCTCTA

TGAAGGAAGTTGAGGACCAGATGCGAAATGT

>Rosellinia_corticium_MUCL_51693

CTCTGTTTACTTTGCAACCCAATCAA-GCCTCATGATTTTTTGCTACCCTATCCCTGAAA

ACGCTCC-----ACGCCCCGCGATCGTAATCTGTCCACATCGATACGCAAGCATCATGCG

CGATCACGGAT----TTCGTGATCGTCGATGATGCTACTTCGTTGGATCCCATCGGATGT

TTATCGAAACTGTTGCTTTAGAGTCAGGCTAACCATGCTTCTCCCGTGTCTAGGTTCACC

TCCAAACCGGCCAATGCGTAAGCTACCT--CGACGCCTCGCGATGACGGCGAGCATCTCG

TGGGCTCCATGTGGAATCATAGGGTAACCAAATCGGTGCTGCTTTCT-------------

----------------------------------------------GGCAACAAATTTCG

GGCGAGCACGGCCTCGACGGCAATGGAGTGTATGTTCTTGACATCCATCGAGTCCAGGAT

TACAGGAACAATCGACTAATACGTCTGGTATAGCTACAACGGAACGTCCGAGCTCCAGCT

CGAGCGTATGAGCGTTTACTTCAACGAGGTATGCAAATACCGGTTCATTGGGGGGTTGTG

ATATCAATATTCTAACGTGTT-GAATTTTTAGGGTGCTGGCAACAAATATGTCCCCCGCG

CCGTTCTTGTCGATCTGGAGCCCGGTACCATGGATGCTGTCCGCGCCGGACCTTTCGGTC

AGCTCTTCCGACCCGACAACTTCGTCTTCGGTCAGTCAGGTGCTGGCAACAACTGGGCCA

AGGGTCACTACACTGAGGGTGCCGAGCTGGTCGACCAAGTTCTCGATGTCGTCCGTCGCG

AGGCTGAGGGTTGCGACTGCCTCCAAAGTTTCCAGATCACCCACTCCCTCGGTGGTGGTA

CTGGTGCCGGTATGGGTACGCTGTTGATCTCCAAGATCCGCGAGGAGTTCCCCGACCGCA

TGATGGCTACCTTCTCCGTTATGCCCTCCCCCAAGGTCTCCGACACCGTTGTTGAGCCTT

ACAACGCCACCCTCTCCGTCCACCAGCTGGTCGAGAACTCCGACGAGACCTTCTGTATCG

ATAACGAAGCTCTGTACGATATCTGCATGCGTACACTGAAGCTATCCAACCCCTCATACG

GTGACCTGAACCACCTGGTCTCCGCCGTCATGTCCGGTGTCACCACCTGCTTGCGTTTCC

CTGGTCAGCTTAACTCTGACCTGCGCAAGTTGGCCGTGAACATGGTGCCGTTCCCTCGTC

TGCACTTCTTCATGGTCGGCTTTGCTCCCCTTACCAGCCGCGGAGCCCACTCTTTCCGTG

CCGTCACGGTTCCTGAGTTGACACAACAAATGTTCGACCCCAAGAACATGATGGCCGCCG

CCGATTTCCGCAACGGTCGTTACCTAACATGCTCTGCCATCTTGTAAGAACCCAGGCCTT

CCCCTTTTACCCTTTCTGTTTGCTAATCTAATAT-TCCAATAGCCGTGGTAAGGTCTCCA

TGAAGGAAGTTGAGGACCAGATGCGAAATGT

>Xylaria_hypoxylon_CBS_122620

---------------------GT----------CCATATTCGGCTGCCTTATACCGAAAC

GCGTCCCAATCTAT-GCCCTTGATCTTAGCTTCTCCACATATACACATCAGCATCGTTCG

CAGTCAACTATCT----CGTAGCTGCCAATAACACAGCTTCCTTGAACCTACTAGATTGT

CTAGTGAAATTATTATATGAGAACTACGCTAACCATGCTTTTCCCTTTTGCAGGTTCACC

TCCAAACCGGCCAATGCGTAAGTCGCCCCCCGATCCTCGATAACGATGTCTAGAACCTCC

CGAGGCTCACACTATACGACAGGGTAACCAAATTGGTGCTGCTTTCT-------------

----------------------------------------------GGCAACAAATTTCC

GGCGAGCACGGTCTCGATGGCAGTGGCGTGTATGTCTATTAGATCTATGAACTACAACCA

CGACTGAATGGTCGACTAACACTTGTGGGCCAGTTACAACGGAACCTCTGAGCTCCAGCT

GGAGCGCATGAGCGTTTACTTCAATGAGGTAGAAAGCCATCAAGTCGCGTTCGTCTCGTG

CAACAACCATTTTGATGC-TGATATTTTCTAGGGTGCTAATAACAAATATGTTCCTCGCG

CCGTCCTCGTCGACTTGGAGCCCGGTACCATGGATGCTGTCCGTTCTGGTCCCTTTGGTC

AGCTCTTCCGACCCGACAACTTCATCTTCGGCCAGTCTGGTGCTGGCAACAACTGGGCCA

AGGGTCACTACACAGAGGGTGCTGAGCTCGTTGACGCCGTTCTTGATGTCGTTCGTCGCG

AGGCTGAGGGCTGCGATTGCCTCCAGGGTTTCCAGATCACCCACTCGCTCGGTGGTGGTA

CCGGTGCTGGTATGGGTACTCTGCTGATCTCCAAGATTCGCGAGGAATTCCCTGACCGCA

TGATGGCTACCTTCTCCGTCATGCCCTCTCCCAAGGTCTCAGATACCGTCGTCGAGCCTT

ACAACGCTACCCTCTCCGTCCACCAGTTGGTTGAGAACTCCGATGAGACCTTCTGTATTG

ACAACGAGGCTCTGTACGATATCTGCATGCGCACCTTGAAGCTATCCAACCCCTCATATG

GTGATTTGAACCACCTTGTCTCTGCCGTCATGTCTGGCGTAACCACCTGCCTGCGTTTCC

CCGGTCAGCTTAACTCTGATCTGCGCAAACTAGCCGTCAACATGGTGCCCTTCCCTCGTC

TACACTTCTTTATGGTCGGGTTTGCCCCTCTCACTAGCCGTGGTGCCCACTCTTTCCGTG

CTGTCACGGTTCCCGAGCTGACCCAGCAAATGTTCGACCCCAAGAACATGATGGCTGCCG

CTGACTTCCGCAACGGTCGTTACCTCACATGCTCTGCTATCTTGTAAGAATTTGCCCTTT

TATCTTTTAACACGGCCAATTGCTAACTTATCCTTTTTACCAGCCGTGGCAAGGTTTCCA

-------------------------------

>Xylaria_arbuscula_CBS_126415

CTCTGTTTACTTTTCAACCCAA-----GCTCCGCCAAATTCTGCTACTTTCGCCCGAGAC

GCGTTACTGTCTATGCTTC--CATCTCCATCATCTCTCATCTGC--CTTAGCATGATAAA

TTACAGACAATAT----TTCTGTCATCAGAGGCATAGCTTGATTGAACTTCTCCCGTTAT

CTGGCAC---TATTGGGTGAAACCTAAACTAACCGCG---CTCCTCTCTACAGGTCCACC

TCCAGACCGGCCAATGCGTAAGTCGCTTTACGACCTTCGACGATAATACCTTGAAACAGC

CGAAACTTACATAGAATGACAGGGTAACCAAATTGGTGCTGCTTTCT-------------

----------------------------------------------GGCAGCAAATCTCC

GGCGAGCACGGTCTCGATGGCAGTGGCGTGTATGTGTACCTATGCCCTGGGTCATGGACT

ATAAGGACACAATGACTGATAGTTATGGAATAGTTACCAGGGAACCTCTGACCTCCAGCT

GGAGCGTATGAGGGTTTACTTCAACGAGGTAGGCTCAATCCGAGCTACATCGATATCATA

CAAGTAATGGTCTAACATGG--GTTTGTTCAGGGCTCGGGCAACAAGTACGTTCCTCGCG

CTGTCCTCGTCGATTTAGAGCCTGGTACCATGGACGCTGTCCGTGCTGGTCCCTTCGGTC

AGCTCTTCCGACCCGACAACATCGTCTTCGGTCAGTCTGGTGCTGGCAACAACTGGGCCA

AGGGTCACTACACTGAGGGTGCTGAGCTTGTTGACAACGTTCTTGACGTTGTCCGTCGTG

AGGCTGAGGGCTGTGACTGCCTCCAGGGTTTCCAGATCACCCACTCGCTCGGTGGTGGTA

CCGGTGCCGGTATGGGTACGCTGCTCATCTCCAAGATCCGTGAGGAGTTCCCCGACCGCA

TGATGGCTACCTTCTCCGTCATGCCCTCCCCCAAGGTATCGGACACCGTCGTCGAACCTT

ACAACGCCACTCTCTCCGTCCACCAGCTGGTCGAGAACTCCGACGAGACCTTCTGCATTG

ACAACGAGGCTCTCTACGACATCTGCATGCGCACCCTGAAGCTATCCAACCCTTCGTACG

GTGACCTGAACCACCTTGTCTCCGCTGTCATGTCTGGCGTCACCACTTGCCTTCGTTTCC

CTGGACAACTTAACTCTGACCTGCGCAAGTTGGCCGTCAACATGGTGCCCTTCCCTCGTC

TGCACTTCTTCATGGTCGGCTTCGCCCCTTTGACCAGCCGTGGTGCCCACTCTTTCCGTG

CCGTCACGGTTCCTGAGTTGACCCAGCAAATGTTCGACCCCAAGAACATGATGGCCGCCG

CTGACTTCCGCAACGGTCGCTACCTGACATGCTCTGCCATCTTGTGAGTACTCTTACTTC

GAGCTTCTAACATGTTTGATTACTAATCTGCTACCATCACTAGCCGTGGCAAGGTCTCTA

TGAAGGAGGTTGAGGACCAGATGCGAAATGT

>Oligostoma_insidiosum_CBS_147288

CTCTGTTTACTGTGCAACCCAATCGAAGCTCCGTCATTTTTTGCTACCTAATCCCTGGAC

GCGTCTACCCCAAGTTTGGCTGGTGCTGGCCTCTCCACATCTCTGCACCAACGTGGTTTG

CAGTAAACAATGCAGCTGGCATCTGCCAATGGCACAACTTCATCCGGCCTGCAGGGGT-T

TCCCAGAAGCGACGATCCGAGAATCGCGCTGACCATGCTTCCCCCGTCTCCAGGTTCACC

TCCAAACCGGCCAATGCGTAGGTTACCCGTCGGTCCTCA-ACACGGCGTCGAGAAACGCC

CGCGGCTCACATGGCTTGACAGGGTAACCAAATCGGTGCTGCTTTCT-------------

----------------------------------------------GGCAACAAATCTCA

GGCGAGCACGGTCTCGACGGCAATGGAGTGTACGTCTTGGACATCCGTGGGACGCGGTCA

CGACGCACTGGTGGACTGACGTGGATGGAACAGCTACAACGGAACCTCGGAGCTCCAGCT

GGAGCGCATGAGCGTTTACTTCAACGAGGTAGCTACC-GCCACGCCATTGCCGCTGCGTA

CGCC-ACTGTTCTAACACGTTGGGCGTTTTAGGGTGCCAACAACAAGTATGTCCCTCGCG

CCGTCCTCGTCGACTTGGAGCCCGGTACCATGGATGCTGTCCGTGCCGGTCCCTTCGGCC

AGCTCTTCCGCCCCGACAATTTCGTCTTCGGTCAATCCGGTGCCGGCAACAACTGGGCCA

AGGGCCATTACACCGAGGGTGCTGAGCTGGTCGACCAAGTGCTCGATGTTGTCCGTCGCG

AGGCCGAGGGCTGTGACTGCCTCCAGGGCTTCCAGATCACCCACTCGCTCGGTGGTGGTA

CCGGTGCCGGTATGGGTACGCTGCTGATCTCCAAGATCCGCGAGGAGTTCCCCGACCGCA

TGATGGCTACCTTCTCCGTCATGCCCTCTCCCAAGGTCTCTGACACTGTCGTCGAGCCCT

ACAACGCCACCCTCTCCGTCCACCAGCTGGTCGAGAACTCCGACCAGACCTTCTGCATTG

ACAACGAGGCTCTGTACGACATCTGCATGCGTACCCTGAAGCTATCCAATCCCTCCTACG

GTGACTTGAACCATCTTGTTTCTGCTGTTATGTCTGGTGTCACTACCTGCTTGCGTTTCC

CTGGCCAGCTTAACTCTGACCTGCGCAAGCTGGCTGTCAACATGGTGCCATTTCCTCGTC

TTCACTTCTTCATGGTCGGCTTTGCGCCTCTAACTAGTCGTGGTGCTTACTCTTTCCGTG

CTGTCACGGTTCCCGAGCTGACCCAGCAAATGTTCGACCCCAAGAACATGATGGCTGCCG

CTGACTTCCGCAACGGCCGTTATCTGACATGCTCTGCCATCTTGTAAGAGTCTACCCCTT

TCC-CTACTGCATAGCTAGTTGCTAACTCAATGCCCACACCAGCCGTGGTAAGGTTTCCA

TGAAGGAAGTGGAGGACCAGATGCGAAACGT

>Xylaria_laevis_HAST_419

CTCTGTTTACTTTGCAGCCCAATCAAAGCTCCGTCATTTTTTGCTACCTCATCCCTGAAC

GCGTCCACCCCGACGTTGGTTAATGCTAGCCTCTCCACATCTCTACACAAGCATCGTGTA

CAGTAAACCACGTAGTTGGCCGCTGCCAATAGCACAGCTTCATCCTCTTTGCAGGGCTGC

CTTCAGAAGCGATAATATGAGAATCACGCTAACCATGCTTCCCCCGTCTCTAGGTTCACC

TCCAAACCGGCCAATGCGTAGGTTACTCTCCGGTCTTCG-TGAAGGCGCTGAGAAGCTCC

TGCGGCTCACATGGCATGATAGGGTAACCAAATCGGTGCTGCTTTCT-------------

----------------------------------------------GGCAACAAATTTCC

GGCGAGCACGGTCTCGACGGCAATGGAGTGTATGTCTTTGGTATTTGTGGAACGTTGCGA

CGA--CACTAGTCGACTGACATGAATGAAACAGCTACAACGGAACCTCTGAGCTCCAGCT

GGAGCGCATGAGCGTTTACTTCAACGAGGTAGATACT-ACTAAACCACTCCTATTGCGTA

CATC-AGGATTCTAATACGATGGAAATTTTAGGGTGCTAACAACAAGTACGTCCCTCGCG

CCGTCCTCGTCGATTTGGAACCCGGTACCATGGATGCTGTCCGTGCCGGTCCCTTCGGCC

AGCTTTTCCGCCCCGACAACTTCGTCTTCGGCCAGTCCGGTGCTGGCAACAACTGGGCCA

AGGGCCACTACACCGAGGGTGCTGAGCTGGTTGACAACGTTCTCGATGTCGTCCGTCGCG

AGGCTGAGGGTTGTGACTGCCTTCAGGGTTTCCAGATCACCCACTCGCTTGGTGGTGGTA

CCGGTGCTGGTATGGGTACGCTGTTGATCTCCAAGATCCGCGAGGAGTTCCCTGACCGCA

TGATGGCTACCTTCTCCGTCATGCCCTCCCCCAAGGTCTCCGACACCGTTGTCGAGCCCT

ACAACGCCACCCTCTCCGTCCACCAGCTGGTCGAGAACTCGGACGAGACCTTCTGCATTG

ATAACGAGGCTCTGTACGACATCTGCATGCGTACCCTGAAGCTATCCAACCCCTCATATG

GTGACTTGAACCACCTTGTCTCCGCTGTCATGTCCGGTGTTACTACTTGCCTGCGTTTCC

CTGGTCAGCTTAACTCTGACCTGCGCAAGTTGGCTGTCAACATGGTGCCATTCCCTCGTC

TGCACTTCTTCATGGTCGGCTTCGCGCCTCTCACTAGTCGTGGTGCTCACTCTTTCCGTG

CTGTCACGGTTCCTGAGCTGACCCAGCAAATGTTTGACCCCAAGAACATGATGGCTGCTG

CTGACTTCCGCAACGGTCGTTACCTGACATGTTCTGCCATCTTGTAAGAAAATACCCCCC

TCCCCTACAACATAGCTTGTTGCTGACTA--TACTCTCACCAGCCGTGGCAAGGTTTCCA

TGAAGGAGGTTGAGGACCAGATGCGAAACGT

>Xylaria_longipes_CBS_148.73

------------------------------------------------------------

------------------------------------------------------------

------------------------------------------------------------

------------------------------------------------------------

------------------------------------------------------------

------------------------------------------------------------

------------------------------------------------------------

----------------------------NNNATGTCTTTGGCATCTATGGAACGTGGCGA

CGACGCACTGGTCAACTGACATGGATGGAATAGCTACAACGGAACCTCCGAGCTCCAGCT

GGAGCGCATGAGCGTTTACTTCAACGAGGTAGATACC-GCCAAACCACTCCCGTTGCGTA

TACC-AACATTCTGACACGTTGGGGATTTTAGGGTGCCAACAACAAGTATGTCCCTCGCG

CCGTCCTCGTCGACTTGGAGCCTGGTACCATGGACGCTGTCCGTGCCGGTCCCTTCGGCC

AGCTCTTCCGCCCCGACAACTTCGTCTTCGGTCAGTCCGGTGCCGGCAACAACTGGGCCA

AGGGTCACTACACCGAGGGTGCTGAGCTGGTCGACCAAGTTCTCGATGTCGTCCGCCGCG

AGGCTGAGGGCTGCGACTGCCTGCAGGGCTTCCAGATCACCCACTCGCTCGGTGGTGGTA

CTGGTGCCGGTATGGGTACGCTGCTGATCTCTAAGATTCGCGAGGAGTTCCCTGACCGCA

TGATGGCTACCTTCTCCGTCATGCCCTCTCCCAAGGTCTCCGACACCGTTGTCGAGCCCT

ACAACGCCACTCTCTCCGTCCACCAGTTGGTCGAGAACTCGGACCAGACCTTCTGCATTG

ACAACGAGGCTCTGTACGACATTTGCATGCGTACCCTGAAGCTATCCAACCCCTCATACG

GTGACTTGAACCACCTTGTCTCCGCGGTTATGTCTGGTGTTACGACCTGCCTGCGTTTCC

CTGGTCAGCTTAACTCTGACCTGCGCAAGTTGGCCGTCAACATGGTGCCGTTCCCCCGTC

TGCACTTCTTCATGGTCGGCTTTGCGCCTCTTACTAGCCGTGGTGCTCACTCTTTCCGTG

CTGTCACGGTTCCCGAGTTGACCCAGCAAATGTTTGACCCCAAGAACATGATGGCTGCCG

CTGACTTCCGCAACGGTCGTTACCTGACATGCTCTGCTATCTTGTAAGCAGCCACCCTTT

CCCTCCGCAGCAC----AGTTGCTAACTGAATACTTTCACCAGCCGTGGCAAGGTTTCCA

TGAAGGAGGTTGAGGACCAGATG--------

>Xylaria_digitata_HAST_919

CTCTGTTTACTTTGCAACCCAATCAAAGCTCCGTCA-TTTTTGCTACCTTATCCCTGAAC

GCGCCCACCCCAAGGTTGGTTGATGCTGGCCTCTCCACATCTCTGCACACACGTGGTTTG

CAGTAAACAATGCAGTTGGCAGCTGCCAATAGCACAACTTCGCCCTGTCTGCAGGGTTA-

TCTCAGAAGCGACAATATGAGAATCGCGCTAACCATGCTTCCCCCGTCTCCAGGTTCACC

TCCAAACCGGCCAATGCGTAGGTCACCCGTCGGTCCTTA-TAACGGCGTCGAGAA-----

----GCTCACATGGCATGATAGGGTAACCAAATCGGTGCTGCTTTCT-------------

----------------------------------------------GGCAACAAATCTCT

GGCGAGCACGGTCTCGACGGCAATGGAGTGTACGTCTTTGGCATCCGTGGGACGCGGCGA

CGATGCACTGATAGGCTGACATGGATGGAACAGCTACAACGGAACCTCCGAGCTCCAGCT

GGAGCGCATGAGCGTTTACTTCAACGAGGTAGATACC-GCCAAACCACTGCCGTTGCGTA

CACC-AATATTCTAACGCGTTGGGGATTTTAGGGTGCCAACAACAAGTATGTCCCCCGCG

CCGTCCTCGTCGACTTGGAACCCGGTACCATGGATGCTGTCCGTGCCGGTCCCTTTGGCC

AGCTTTTCCGCCCCGACAACTTCGTCTTCGGTCAGTCCGGTGCCGGCAACAACTGGGCCA

AGGGCCACTACACCGAGGGTGCTGAGCTGGTTGACAACGTTCTCGATGTTGTCCGTCGCG

AGGCCGAGGGCTGCGACTGCCTCCAGGGCTTCCAGATCACCCACTCGCTCGGTGGTGGTA

CCGGTGCTGGTATGGGTACGCTGCTGATCTCCAAGATTCGCGAGGAGTTCCCCGACCGTA

TGATGGCTACCTTCTCTGTCATGCCTTCTCCTAAGGTCTCGGACACCGTTGTCGAGCCCT

ACAACGCCACCCTCTCCGTCCACCAGCTGGTCGAGAACTCGGACGAGACCTTCTGCATTG

ACAACGAGGCTCTGTACGACATCTGCATGCGTACCCTAAAGCTATCCAACCCCTCCTACG

GTGACCTGAACCACCTTGTCTCCGCTGTTATGTCTGGTGTTACTACCTGCCTGCGTTTCC

CTGGTCAGCTTAACTCTGACCTGCGCAAGTTGGCTGTCAACATGGTGCCATTCCCTCGTC

TGCACTTCTTCATGGTCGGCTTTGCGCCTCTGACTAGTCGTGGTGCTCACTCTTTCCGTG

CTGTCACGGTTCCCGAGCTGACCCAGCAAATGTTTGACCCCAAGAACATGATGGCTGCCG

CAGACTTCCGCAACGGCCGTTACCTGACGTGCTCTGCTATCTTGTAAGAAACCACCCTTT

CCC-CTACAGCATAGCTAGTTGCTAACCGAATACTCTCACCAGCCGTGGCAAGGTCTCCA

TGAAGGAAGTTGAGGACCAGATGCGAAACGT

>Leptomassaria_simplex_CBS_147282

CTCTGTTTACTTTGCAACCCAATCAAAGCTCCATCATTTTTTGCTACCTGATCCCTGGAC

GCGTCCACCTCAAGGTTGGCGGGTGCTGGCCTCTCCACACCTCTTAACCAACGTGACTTG

CCGTACACATTGGAGTTGGCAGCTGTCAATTGCACAA---GATCCTGTCGGCAGGGT--T

TTACAGAAGCGACCATGTGAGAATCGCGCTAACCATGCTTTCCCCGTGTCCAGGTTCACC

TCCAAACCGGCCAATGCGTAGGTCACCCGTCACTCCTCG-TACCGGCGTCGGGACACTCC

CGCGGCTCACATGGCATCATAGGGTAACCAAATCGGTGCTGCTTTCT-------------

----------------------------------------------GGCAACAAATCTCT

GGCGAGCACGGTCTCGACGGCAATGGAGTGTACGTGTTTGGCATCCGTGGGACGAGGTCA

CGGCGCACTGGTAGACTGACGTGGATGGCACAGCTACAACGGAACCTCCGAGCTCCAGCT

GGAGCGCATGAGCGTTTACTTCAACGAGGTAGGTGCC-GCCAAGCCGCTGCCGTTGCGTA

CATC-CACAGGCTGACACG-TGGGGATTCCAGGGTGCCAACAACAAGTATGTCCCTCGCG

CCGTCCTCGTCGACTTGGAGCCCGGTACCATGGATGCTGTCCGTGCCGGCCCCTTCGGCC

AGCTTTTCCGCCCCGACAACTTCGTCTTCGGTCAGTCCGGTGCCGGCAACAACTGGGCCA

AGGGCCACTACACCGAGGGTGCTGAGCTGGTTGACCAGGTTCTCGATGTGGTCCGTCGCG

AGGCTGAAGGCTGCGACTGCCTCCAGGGCTTCCAGATCACCCACTCGCTTGGTGGTGGTA

CCGGTGCCGGTATGGGTACGCTGCTGATCTCCAAGATTCGCGAGGAGTTCCCCGACCGCA

TGATGGCTACCTTCTCCGTCATGCCCTCTCCTAAGGTCTCTGACACCGTCGTCGAGCCCT

ACAACGCCACCCTCTCCGTCCACCAGCTGGTCGAGAACTCGGACGAGACCTTCTGCATTG

ACAACGAGGCTCTGTACGACATCTGCATGCGTACCCTCAAGCTATCCAACCCCTCCTACG

GTGATTTGAACCACCTGGTCTCCGCCGTTATGTCTGGTGTTACTACCTGCCTGCGTTTCC

CTGGTCAGCTTAACTCTGACCTGCGCAAGTTGGCTGTCAACATGGTGCCATTCCCTCGTC

TGCACTTCTTCATGGTCGGCTTTGCGCCTCTGACAAGTCGTGGTGCTCACTCTTTCCGTG

CCGTCACGGTTCCCGAGCTGACCCAGCAAATGTTCGACCCGAAGAACATGATGGCTGCCG

CTGACTTCCGCAACGGCCGTTATCTGACATGCTCTGCTATCTTGTAAGAAAACACCGCTT

TCCCTT-CCGCATAGACAGTGGCTAACTAAATACCCCCACCAGCCGTGGCAAGGTTTCCA

TGAAGGAAGTGGAGGACCAGATGCGAAACGT

>Stilbohypoxylon_elaeicola_Y.M.J._173

CTCTGTTTACTTTGCAACCCAATCACAGCTCCGCCATTTTTTGCTGCCCTATCCCTGAAC

GCGTCCACTTCCATGCTCTTGGATCCTAGCCTCTCCACATCTATATGCAAGCTTCGATGT

CAGACGTCAATGCTGCCTATGGCTGCCAATTACACGGCTTCATGCAAATTGTCGGGTTGC

CCAGAGAAGCTTAGAAATAGGAAGCTTGCTAACCGGGCGT-CTCCCTCAACAGGTTCACC

TCCAAACCGGCCAATGCGTAGGTCGCCATCCGACCCTCCGTAACGACTTCGAGAATCCCC

CGTGGCTCACATCGTATGATAGGGTAACCAAGTTGGTGCTGCTTTCT-------------

----------------------------------------------GGCAGCAAATCTCG

GGCGAGCATGGTCTCGATGGCAGCGGCGTGTATGTCCTTGGCATCAATGGAACACGATGA

CGAGCCAATGGTCGACTGACATGAGCCGAACAGGTACAATGGAACCTCCGAGCTCCAGCT

TGAGCGCATGAGCGTCTACTTCAACGAGGTATCACATCACTGACCCACGTTTTTCATGCA

CGACGAGCATGCTAATATGTCGAATCATCCAGGGTGCCGGCAACAAATATGTCCCTCGCG

CCGTTCTCGTCGATCTGGAGCCCGGTACCATGGACGCCGTCCGTGCGGGTCCTTTCGGTC

AGCTCTTCCGACCCGATAACTTCGTCTTTGGCCAGTCCGGTGCCGGCAACAACTGGGCCA

AGGGTCACTACACTGAGGGTGCTGAGCTCGTCGACCAAGTCCTCGACGTTGTCCGTCGTG

AGGCCGAGGGCTGCGACTGCCTCCAGGGTTTCCAGATCACCCACTCGCTTGGTGGCGGTA

CCGGTGCCGGTATGGGTACGCTGTTGATCTCCAAGATCCGTGAGGAGTTCCCTGACCGCA

TGATGGCCACCTTCTCCGTCATGCCCTCACCCAAGGTCTCCGACACCGTTGTCGAGCCCT

ACAACGCCACCCTCTCCGTCCACCAGCTGGTCGAGAACTCCGATGAGACCTTCTGTATTG

ACAACGAGGCTCTTTACGATATCTGCATGCGTACCTTGAAGCTATCCAACCCCTCGTACG

GCGACCTGAACCACCTGGTCTCCGCTGTCATGTCTGGCGTTACCACCTGCCTCCGTTTCC

CAGGTCAGCTGAACTCTGATCTGCGTAAATTGGCCGTCAACATGGTGCCCTTCCCTCGTC

TGCACTTCTTCATGGTCGGCTTTGCTCCCCTCACCAGCCGTGGTGCTCACTCTTTCCGGG

CTGTCACTGTTCCGGAATTGACTCAGCAAATGTTCGACCCTAAGAACATGATGGCTGCTG

CTGACTTCCGTAACGGGCGCTACCTGACATGCTCTGCCATCTTGTAAGAATTCACCCTTT

CTCTGCAAGCATGAGCCCATCACTAACTTGACGC-TCTACTAGCCGTGGCAAGGTTTCCA

TGAAGGAGGTCGAGGATCAGATGAGAAATGT

>Xylaria_ianthinovelutina_HAST_553

CTCTGTTTACTTTGCAACCCAATCACAGCTCCGCGATTTTTCGCTGCCCTATCCCTGAAC

GCGTCCGCTTTCATGCTCTTGGATGCTAGCCTCTCCACATCTATATGCAAGCCTCGATGT

CAAACGTCAATGCTGCCTATAGCTGCCAATAAAACGGCTTCATGCAAATGGCCGGGTTGC

CCAGAGAAACTGAGAAAGAAGAACCTCGCTAACCGGGTGT-CCCCCTCGACAGGTTCACC

TCCAAACCGGCCAATGCGTAGGTCGCCATCCGATCCTCCGCAACGACTTCGAGAACGCCC

CGTGGCTCACATCGCATAACAGGGTAACCAAATCGGTGCTGCTTTCT-------------

----------------------------------------------GGCAGCAAATCTCG

GGCGAGCATGGTCTCGATGGCAGTGGCGTGTATGTCCTTGGCATCAATGGAACATGGTGA

CGAGCCAAGGGTCAACTGACATGGGTGGGATAGCTACAACGGAACCTCCGAGCTCCAGCT

CGAGCGCATGAGCGTTTACTTCAACGAGGTAGCCCATCACTGACCCACTTTTTTTATGCA

CGACGAGCATTCTAACGTGTCGAATCATCCAGGGTGCCGGTAACAAATATGTCCCTCGCG

CCGTTCTCGTCGATCTGGAGCCCGGTACCATGGATGCCGTCCGTGCGGGTCCTTTCGGTC

AGCTCTTCCGACCCGATAACTTCGTCTTTGGCCAGTCCGGTGCTGGCAACAACTGGGCCA

AGGGTCATTACACTGAAGGTGCTGAGCTCGTGGACCAAGTCCTTGATGTTGTCCGTCGCG

AGGCCGAGGGCTGCGACTGCCTTCAGGGCTTCCAGATCACCCACTCGCTCGGTGGCGGCA

CTGGTGCTGGTATGGGTACGCTGTTGATCTCTAAGATCCGTGAGGAGTTCCCTGACCGCA

TGATGGCCACCTTCTCCGTCATGCCCTCGCCCAAGGTCTCCGATACCGTTGTCGAGCCCT

ACAACGCCACCCTCTCCGTCCACCAGCTGGTCGAGAACTCCGATGAGACCTTCTGCATTG

ACAACGAGGCTCTCTACGACATCTGCATGCGCACACTGAAGCTATCCAACCCCTCATACG

GCGACCTGAACCACCTGGTCTCCGCCGTCATGTCTGGTGTGACCACCTGCCTGCGCTTCC

CTGGTCAGCTGAACTCTGATCTGCGCAAATTGGCCGTCAACATGGTGCCCTTCCCTCGTC

TGCACTTCTTCATGGTCGGCTTTGCCCCTCTTACGAGCCGTGGTGCTCATTCTTTCCGTG

CGGTCACCGTTCCCGAGTTGACCCAGCAGATGTTCGACCCTAAGAACATGATGGCTGCTG

CTGACTTCCGTAACGGTCGCTACCTGACATGCTCTGCCATCTTGTAAGAACCCC----TC

TCCTGTAGGCATGAGTCCATCGCTAACTTGACGC-TCTACTAGCCGTGGCAAGGTTTCCA

TGAAGGAAGTTGAGGATCAGATGCGAAACGT

>Xylaria_polymorpha_MUCL_49884

-----------TTGCAACCCAATCAAAGCTCCGTCATTTTTTGCTACCTGATCCCTGGAC

GCGTCTGCCCCGATGTTGGTTGATGCTGGTCTCTCCACATCTCTACACACGCATCGTCTG

CAGTAAACAATGCAGCTGGCAATTGCCAATCGCACAACTTCATTCTGTCTGCAAGGTTGC

CTGCAGAAGCGATAACTTGAGAATCGCGCTAATCATACTTCGCCTGTCTCTAGGTTCACC

TTCAAACCGGCCAATGCGTAGGT-----------CCTCT-TAACGGCGTCGAGAAGCTCC

CGCGGCTCACATGGCATGATAGGGTAACCAAATTGGTGCTGCTTTCT-------------

----------------------------------------------GGCAACAAATCTCG

GGCGAGCACGGCCTCGATGGCAATGGAGTGTATGTGTTTGGCATCTGTGGAACGTGGCGA

AGACGCACTGGTCGACTGACATGGATGTGACAGCTACAACGGAACCTCCGAGCTCCAGCT

GGAGCGCATGAGCGTTTACTTCAACGAGGTAGATACC-GCCAAGCCACTGCCGTTGCGTA

CGCC-AATATGCTAACATG---GCGGTTTTAGGGTGCCAACAACAAGTATGTCCCTCGCG

CCGTCCTCGTCGACTTGGAGCCCGGTACCATGGATGCTGTCCGTGCCGGTCCTTTTGGCC

AGCTCTTCCGCCCCGACAACTTCGTCTTCGGTCAATCCGGTGCCGGCAACAATTGGGCCA

AGGGCCACTACACTGAGGGTGCTGAATTGGTTGACACCGTTCTCGATGTTGTCCGTCGCG

AGGCTGAGGGCTGTGACTGCCTCCAAGGCTTCCAGATCACCCACTCGCTCGGTGGTGGTA

CCGGTGCCGGTATGGGTACGCTGCTGATCTCCAAGATTCGCGAGGAGTTCCCCGACCGCA

TGATGGCTACCTTCTCCGTCATGCCCTCTCCTAAGGTCTCCGACACCGTCGTCGAGCCCT

ACAACGCCACCCTCTCCGTCCACCAGCTGGTCGAGAACTCGGACGAGACCTTCTGCATTG

ACAACGAGGCTCTGTACGACATCTGCATGCGTACCCTGAAGCTATCCAACCCCTCATACG

GTGACTTGAACCACCTTGTCTCCGCTGTCATGTCTGGTGTTACCACCTGCCTGCGTTTCC

CTGGTCAACTTAACTCTGACCTGCGCAAGTTGGCTGTCAACATGGTGCCATTCCCCCGTC

TGCACTTCTTCATGGTCGGCTTTGCGCCTCTCACTAGTCGTGGTGCTCACTCTTTCCGCG

CTGTCACGGTTCCCGAGCTGACTCAGCAAATGTTCGACCCCAAGAACATGATGGCTGCCG

CTGACTTCCGCAACGGTCGTTACCTGACATGCTCTGCTATCTTGTAAGGAACTACCCTTT

TCTCCT---TTACACCTAGTTGCTAACTGATTGCTCTCGCCAGCCGTGGCAAGGTTTCCA

-------------------------------

>Xylaria_atrosphaerica_HAST_91111214

CTCTGTTTACTCTGCAACCCAATCAAAGCTCCGTCATTTTTTGCTACCTGATCCCTGGAC

GCGTCTGCCCCGATGTTGGTTGATGGCGGCCTCTCCACATCTCTACACACGCATCGTCCG

TAGTAAACAATGCAGCTGGCAGTTGCCAATAGCACAACTTCATTCTTCCTGTCGTGTTGC

CTGCAGAAGCGATACCTTGAGAATCGCGCTAATCATGCTTCCCCTGTCTCTAGGTTCACC

TTCAAACTGGCCAATGCGTAGGT-----------CCTCT-TAACAGCGTCGAGAAGCTCC

CGCAGCTCACATGGCATGATAGGGTAACCAAATTGGTGCTGCTTTCT-------------

----------------------------------------------GGCAACAAATCTCT

GGCGAGCACGGTCTCGATGGCAATGGAGTGTATGTTTTGGGTATCTGTGGAACGTGGCGA

AGACGCACTGGTCAACTGACATGGATGGAACAGCTACAATGGAACCTCCGAGCTCCAGCT

GGAGCGCATGAGCGTTTACTTCAACGAGGTAGGTACC-GCCAAGCTACTCCCGTTGCGTA

CACC-AACATTCTAACATG---GGGGTTCTAGGGTGCCAACAACAAGTACGTCCCTCGCG

CCGTCCTCGTCGACTTGGAGCCCGGTACCATGGATGCTGTCCGTGCCGGTCCTTTTGGCC

AGCTCTTCCGCCCCGACAACTTCGTCTTCGGTCAGTCCGGTGCCGGCAACAACTGGGCAA

AGGGCCATTACACTGAGGGTGCTGAGCTGGTCGACAACGTTCTCGATGTCGTCCGTCGCG

AGGCTGAGGGCTGCGACTGCCTCCAGGGCTTCCAGATCACCCACTCTCTCGGTGGTGGTA

CCGGTGCCGGTATGGGTACGCTGCTGATCTCCAAGATCCGCGAGGAGTTCCCCGACCGCA

TGATGGCTACCTTCTCCGTCATGCCCTCTCCCAAGGTCTCCGACACCGTCGTCGAGCCCT

ACAACGCCACCCTCTCCGTCCACCAGCTGGTCGAGAACTCGGACGAGACCTTCTGCATTG

ACAACGAGGCTCTGTACGACATCTGCATGCGTACCCTGAAGCTATCCAACCCCTCATACG

GTGACTTGAACCACCTTGTCTCCGCCGTCATGTCTGGTGTTACCACCTGCCTGCGTTTCC

CTGGTCAGCTTAACTCTGACCTGCGCAAGTTGGCTGTCAACATGGTGCCATTCCCTCGTC

TGCACTTCTTCATGGTCGGCTTTGCGCCTCTCACTAGTCGTGGTGCTCACTCTTTCCGTG

CTGTCACGGTTCCCGAGCTGACTCAGCAAATGTTCGACCCCAAGAACATGATGGCTGCCG

CTGACTTCCGCAACGGTCGTTACCTGACATGCTCTGCTATCTTGTAAGGAACTACCCCTG

TCCCCT---TTACACCTAGTTGCTAACTAAATGCTCTCACCAGCCGTGGCAAGGTTTCCA

TGAAGGAGGTTGAGGACCAGATGCGAAACGT

>Amphirosellinia_nigrospora_HAST_91092308

CTCTGTTTACTTTGCAACCCAATCAAAGCTCTATCATTTTTGGCTACCTTGTCTCTGCAC

ACGTTTACCCTGATGTTGGTTGATGCTGGCCTCTCCACATCTCGGCAACACCATCATTTG

TAGTGGATCATACAGCTGATAACTGCCGATCGCACAACTTTATCCTACCCGCATGGTTAT

CCAAAGAAGCGAGAACATGAGAATCGCGCTAACCATGCTT-CCCCGTCTCTAGGTTCACC

TTCAAACCGGCCAATGCGTAGGTCATCCTTCGGTCTTTA-TAACGACGTCGAGAAGCTCC

CGCGGCTCACATGGTTTAACAGGGTAACCAAATTGGTGCTGCTTTCT-------------

----------------------------------------------GGCAACAAATCTCG

GGCGAGCACGGTCTCGACGGCAATGGAGTGTATGTTTTTGGCATTCGTGGAACGTGGGGA

TGACGCACTGGTCAACTGACATGGATGGAACAGCTACAACGGAACCTCGGAGCTCCAGTT

GGAGCGCATGAGCGTTTACTTCAACGAGGTAGGTAGC-ACCAAACCACTCCCGTTGCATG

CACC-ACATTTCTAACGCGTGGGGGGTTTTAGGGTGCCAACAACAAGTATGTCCCTCGCG

CAGTCCTCGTCGACTTGGAGCCGGGTACCATGGATGCTGTCCGTGCCGGTCCCTTCGGCC

AGCTCTTCCGCCCCGACAACTTCGTCTTCGGCCAGTCCGGTGCCGGCAACAACTGGGCCA

AGGGCCATTACACTGAGGGCGCTGAGCTGGTTGACCAAGTTCTTGATGTTGTCCGTCGCG

AGGCTGAGGGCTGCGACTGCCTCCAGGGCTTCCAGATCACTCACTCGCTCGGTGGTGGTA

CCGGTGCCGGTATGGGTACGCTGCTGATCTCCAAGATTCGCGAGGAGTTCCCCGACCGCA

TGATGGCTACTTTCTCCGTCATGCCCTCTCCCAAGGTCTCCGACACTGTCGTCGAGCCCT

ACAACGCCACCCTCTCGGTCCACCAGCTGGTCGAGAACTCGGACGAGACCTTCTGCATTG

ACAACGAGGCTCTGTACGATATCTGCATGCGTACACTGAAGCTATCCAACCCCTCGTACG

GTGACTTGAACCACCTTGTCTCCGCTGTCATGTCTGGCGTTACTACGTGCCTGCGTTTCC

CTGGTCAGCTGAACTCTGACCTACGCAAGTTGGCTGTCAACATGGTGCCATTCCCTCGTC

TGCACTTCTTCATGGTTGGCTTTGCGCCTCTTACCAGCCGTGGTGCTCACTCTTTCCGTG

CCGTGACGGTTCCTGAGTTAACTCAGCAAATGTTCGACCCCAAGAACATGATGGCTGCTG

CTGATTTCCGCAACGGTCGTTACCTGACGTGCTCTGCTATCTTGTAAGCAACCACCCTTT

CGCCTCACAGGACTATAGATTGCTAACT-AATACTCCCACCAGCCGTGGTAAGGTTTCCA

TGAAGGAAGTTGAGGACCAGATGCGAAACGT

>Dematophora_necatrix_CBS_349.36

CTCTGTTTACTTGGCAACCCAATCAA-GCCTCGCCAATTTTTGCTGCCCTA-TCCTGGAC

GCGCCCGCCCCCCCCTTGCCCAGATTTTTAGTCTCCACATCGATGCGCGAGCGTCGTTCG

TGATGAACCATGT----GGCAGTTGTGAAGTGCACGGCTTTGTTGAATCCGCCCGGATGG

CGAGAGAGGGTGTGGCATGAGCATCCCGCTAACCAGACTTTCTCCATCTATAGGTTCACC

TCCAGACCGGCCAGTGCGTAAGTCGCCCTCGTATCCACCGCGATGACGACGAAGATTCAT

CGAGTCTCACACAACACAATAGGGTAACCAAATTGGTGCTGCTTTCT-------------

----------------------------------------------GGCAACAAATCTCC

GGCGAGCATGGCCTCGACGGCAATGGCGTGTATGTTCCCAATCTCAAGATGCAGGGCAAT

CGATGGAAACGTAAACTGACGCACATGGAACAGCTACAACGGAACCTCCGAGCTCCAGCT

CGAGCGCATGAGCGTCTACTTCAACGAGGTACGCTGGTGCCAATCCATCTTAGCTCTTCG

CAATGAATGTTCTAATTCGTC-GGCTATCTAGGGTGCTGGCAACAAATATGTTCCCCGTG

CCGTTCTCGTCGATCTGGAGCCCGGTACCATGGATGCTGTCCGCGCCGGTCCCTTTGGTC

AGCTCTTCCGACCCGACAACTTCGTCTTCGGTCAGTCTGGTGCTGGCAACAACTGGGCCA

AGGGTCACTACACTGAGGGCGCCGAGCTGGTCGACAACGTCCTCGATGTTGTGCGTCGTG

AGGCTGAGGGCTGCGACTGCCTTCAGGGTTTCCAGATCACCCACTCCCTCGGTGGTGGTA

CCGGTGCCGGTATGGGTACTCTGTTGATCTCCAAGATTCGCGAAGAGTTCCCCGACCGCA

TGATGGCCACCTTCTCCGTTATGCCCTCTCCTAAGGTCTCAGACACCGTCGTCGAGCCCT

ATAACGCTACCCTCTCCGTCCACCAGCTGGTCGAGAACTCAGACGAGACCTTCTGCATTG

ACAACGAAGCTCTGTACGACATCTGCATGCGTACCTTGAAGCTATCCAACCCTTCATACG

GTGACCTGAACCACCTAGTCTCCGCTGTCATGTCTGGTGTTACCACCTGCTTGCGTTTCC

CCGGTCAGCTGAACTCTGACCTGCGCAAGTTGGCCGTGAACATGGTGCCATTCCCCCGTC

TGCACTTCTTCATGGTTGGCTTTGCCCCTCTCACTAGCCGCGGCGCCCACTCTTTCCGCG

CCGTTACCGTTCCCGAGTTAACCCAGCAAATGTTCGACCCTAAGAATATGATGGCTGCTG

CTGACTTCCGTAACGGTCGCTACCTTACCTGCTCTGCTATCTTGTAAGCGACAGCCCCTT

TC-CCTCTGGCACGCC-TATCGCTAACGTGATACTCTTACTAGCCGTGGCAAGGTTTCGA

TGAAGGAAGTGGAGGATCAGATGCGAAAC--

>Xylaria_oxyacanthae_859_JDR

CTCTATTTACTGGGCAACCCAATCACAGCCCGCTCGTATTTTGCTGCCTTATCCCTGAAC

GCGTCCG--CG-ATAGCCCGGGATGCTAGCCTCTCCACACATACACATGAGGGTCATCTG

CAGTCGAGAATGC----CATGGATGCCAGCAGCAGAGCTTATTTT-----------TCA-

-------------------GGAAAAAGACTAACCACGCGTGCCTCGTCTGCAGGTTCACC

TCCAAACCGGCCAATGCGTACGTCGCTCATCGACCCTCTATGGCGACGTCGTCGATCGCC

CGAAACTCACATGACATGATAGGGTAACCAAATTGGTGCTGCTTTCT-------------

----------------------------------------------GGCAACAAATTTCC

GGCGAGCACGGTCTCGACGGCAATGGCGTGTATGTGCTGGTCTTCAGG--AGCAAGAGGA

CTACGCGATGGCCGACTGACACTCATGGAATAGTTACAACGGAACCTCTGAGCTCCAGCT

CGAGCGCATGAGCGTCTACTTCAACGAGGTAGCCACCCACCCACTCGCCTCCATCTCATA

TTACCGATAGTCTAACATGTTTGGGTTTTCAGGGTGCTGGCAACAAGTATGTCCCTCGCG

CCGTTCTCGTCGATTTGGAGCCAGGTACCATGGATGCTGTCCGTGCCGGTCCTTTCGGCC

AGCTCTTCCGCCCCGACAACTTCGTCTTCGGCCAGTCTGGTGCTGGCAACAACTGGGCCA

AGGGCCACTACACTGAGGGTGCTGAGCTCGTTGACAGCGTTCTTGATGTCGTTCGTCGTG

AGGCCGAGGGTTGCGACTGCCTCCAGGGTTTCCAGATCACCCACTCGCTCGGTGGTGGTA

CCGGTGCCGGTATGGGTACCCTGCTTATCTCCAAGATTCGTGAGGAGTTCCCCGACCGCA

TGATGGCCACCTTCTCCGTCATGCCCTCTCCCAAGGTCTCCGACACCGTCGTCGAGCCCT

ACAACGCCACCCTCTCCATCCACCAGCTGGTCGAGAACTCCGACGAGACCTTCTGTATTG

ATAACGAGGCTCTCTACGACATCTGCATGCGCACCCTGAAGCTGTCCAACCCCTCATATG

GTGACTTGAACCACCTCGTCTCCGCTGTCATGTCGGGTGTCACTACTTGCCTGCGTTTCC

CTGGTCAGCTTAACTCTGATCTGCGCAAGTTGGCTGTGAACATGGTGCCCTTCCCTCGTC

TGCACTTCTTCATGGTCGGCTTTGCCCCTCTCACTAGCCGTGGTGCCCACTCTTTCCGTG

CTGTCACGGTTCCTGAGTTGACCCAGCAGATGTTCGACCCCAAGAACATGATGGCTGCGG

CTGACTTCCGTAACGGTCGTTACCTGACATGCTCTGCTATCTTGTAAGTTGATTTCCCTC

CTTTGTTCGAGATTGTTGGTAACTAACATTGTGTTTTCTCTAGCCGTGGCAAGGTTTCCA

TGAAGGAGGTTGAGGACCAGATGCGCAACGT

>Albicollum_vincensii_CBS_147286

CTCTGTTTACTTTGGACGCTGGTCGA-GCTCCGTCAATTTTTGCTGCCCTGTCCCTGAAC

GCGTCCCTTCCCTTGCCCCTTGACCTTGGCCTCTCCACATTCACACATCAGCACTCTCGA

TAGTAGACAGTCT----GGTGGTTGTCAATAACACAGCTTCAT--AATGCACCAAACTGC

CTTGAGGAACCATTACTTGAGAATTGAGCTAACCGTCCTTCCCGCGTCTATAGGTCCACC

TCCAAACCGGCCAATGCGTAAGTCACCATTCGACC-TCGACGATGACACCGAGAATGTTC

AAGTACTTACAT-GAATGATAGGGTAACCAAATTGGTGCTGCTTTCT-------------

----------------------------------------------GGCAGCAAATCTCT

GGCGAGCACGGCCTCGACAGCAATGGCGTGTATGTGGAGAGCCGATGGAAATATTG----

CGATGGAATGGTCGACTGACAGTCATGAAACAGCTACAACGGAACTTCTGAGCTCCAGCT

CGAGCGCATGAGCGTCTACTTCAACGAGGTACGGACGCATTGAAGGCAATATTCTTAGCA

CGACCGACA-GCTAACTCGTTGAATTTCACAGGGTTCCGGCAACAAGTATGTTCCCCGCG

CCGTTCTCGTCGATCTCGAGCCCGGTACTATGGACGCTGTCCGTGCTGGTCCCTTCGGTC

AGCTCTTCCGACCCGACAACTTCGTTTTCGGTCAGTCCGGTGCCGGCAACAACTGGGCCA

AGGGTCATTACACTGAGGGTGCCGAGCTGGTCGACCAGGTTCTCGACGTCGTCCGTCGCG

AGGCAGAGGGCTGCGACTGCCTGCAGGGTTTCCAGATCACACACTCGCTCGGTGGTGGCA

CCGGTGCCGGTATGGGTACGCTGTTGATCTCCAAAATTCGCGAGGAATTCCCCGACCGCA

TGATGGCCACATTCTCCGTTGTACCCTCTCCCAAGGTCTCCGACACTGTTGTCGAGCCCT

ACAACGCCACCCTCTCCGTCCACCAGCTGGTCGAGAACTCCGACGAGACCTTCTGCATTG

ACAATGAGGCTCTATACGACATCTGCATGCGCACGCTGAAGCTGTCCAACCCCTCGTACG

GTGACCTGAACCACCTTGTCTCCGCCGTTATGTCTGGTGTCACCACCTGTCTGCGTTTCC

CCGGTCAGCTCAACTCCGATCTGCGCAAGTTGGCTGTCAACATGGTGCCCTTCCCTCGTC

TTCATTTCTTCATGGTCGGATTTGCTCCTCTCACCAGCCGTGGTGCTCAATCTTTCCGCT

CTGTCACCGTTCCCGAGTTGACCCAGCAAATGTTCGACCCCAAGAATATGATGGCTGCCT

CTGACTTCCGTAACGGTCGTTACCTAACATGCTCTGCCATCTTGTAAGAAAGACCCCGTT

CCCCTTGGTCCGAACCAATTTACTAACTTTTTGCCCTCACTAGCCGTGGCAAGGTCTCCA

TGAAGGAAGTCGAGGATCAGATGCGAAATGT

>Albicollum_longisporum_CBS_147283

CTCTGTTTACTTTGCAACCCGATTGA-GCCCGGTCAATTTTCGCTGCCCTA-TCCTAAAC

GTGT-CCTCCCAATGCCACTTGGTCTTGGTCTCTCTACATGTAAACATCAGCACAATCGA

CGGCAAACCGTACAGT-GGTGGTTGCCAATAACAAAGCTTCAT--AAACTGCCAAACCGT

CTGGAGGAACTGCCACATGAGAACTGGGCTAACCGTGCTTTCCACGTCTATAGGTTCACC

TTCAAACCGGCCAATGCGTAAGTCGCCCATCGACCCTCCACGATGACACCGGGAATTTTT

AAGGGCTCACATATAACGATAGGGTAACCAAATCGGTGCTGCTTTCT-------------

----------------------------------------------GGCAGCAAATCTCG

GGCGAACACGGCCTCGACAGCAATGGCGTGTATGTACAGAACCCGTGAAAATAGTA----

CGGTGGGATGGTTAACTGACAGCCTCCAAACAGTTACAACGGAACATCCGAGCTCCAGCT

CGAGCGCATGAGCGTCTACTTCAATGAGGTACGCAAGCATTAAAATCGATACCTGTAGCG

CAACCGACA-TCTAACCTATTCAATCTCACAGGGTTCCGGCAACAAGTATGTTCCCCGTG

CCGTTCTCGTCGATCTCGAGCCCGGTACCATGGATGCTGTCCGTGCTGGTCCCTTCGGTC

AGCTCTTCCGACCCGACAACTTCGTCTTCGGCCAGTCTGGTGCCGGCAACAACTGGGCCA

AGGGTCATTATACTGAGGGTGCCGAACTGGTCGACCAGGTTCTCGACGTCGTTCGTCGCG

AGGCAGAGGGCTGCGACTGCCTGCAGGGTTTCCAGATCACCCACTCGCTCGGTGGTGGTA

CTGGTGCCGGTATGGGTACGTTGCTGATCTCTAAGATCCGCGAGGAATTTCCCGACCGCA

TGATGGCCACCTTCTCTGTTGTGCCCTCTCCCAAGGTCTCTGACACCGTCGTCGAGCCCT

ACAACGCCACCCTCTCCGTCCACCAGCTGGTCGAGAACTCCGACGAGACCTTCTGCATTG

ACAATGAGGCTCTGTACGATATCTGCATGCGCACCCTGAAGCTGTCCAACCCCTCATATG

GTGACCTAAACCACCTTGTCTCCGCCGTCATGTCTGGTGTCACCACCTGCCTGCGCTTCC

CCGGTCAACTCAACTCCGATCTGCGCAAGTTGGCTGTCAATATGGTGCCCTTCCCTCGTC

TACACTTCTTCATGGTCGGATTTGCTCCTCTTACCAGCCGCGGTGCTCAATCTTTCCGTT

CTGTTACTGTTCCCGAGTTGACCCAGCAAATGTTCGACCCCAAGAACATGATGGCTGCCT

CTGACTTCCGTAACGGTCGTTATCTGACATGCTCTGCCATCTTGTAAGAATACTCAATT-

---CCCTTAGTATGATAACTGACTAATTTCTCACCCTCCCTAGCCGTGGTAAGGTATCCA

TGAAGGAAGTCGAGGATCAGATGCGAAATGT

>Stromatoneurospora_phoenix_BCC_82040

CTCTGTTTACTCTGCAACCCGACTGA-GCTCCACCAAATTTTGCTGCCCTATTCTTGAAC

GCGTTCCAACGTATGCACCCCAACTTCACCATCTCCACTTGTGCACACAAGCATTACCTG

TAGCG---------------A----CAGACAGCAAAGCTTCATAC---TTGTCAATCTAT

CAAAAG----------AAAGGAATGAGGCTAACGATAGATTCGACGACTACAGGTCCACC

TCCAAACCGGCCAATGCGTAAGTCGTCTTCCCCTTC---ACGATGGTAACGAAAG-TTGG

AGAAACTTACGCGAAACAATAGGGTAACCAAGTTGGTGCTGCTTTCT-------------

----------------------------------------------GGCAACAAATCTCC

GGAGAGCACGGTCTCGATGGCAGTGGCGTGTATGTTTTTTAC--CTAC-----ACTGCAG

TTGTACAGCGGTTGCCTAACAGCGATGGAACAGGTACAACGGAACCTCCGAGCTCCAGCT

CGAGCGCATGAGCGTCTACTTCAATGAGGTACAGAAAATTTATCCTATTATTGGCTAAAG

TGAC-AATT-TTTAACGCGATGGATTACACAGGGTGCCGGCAACAAGTTCGTCCCTCGCG

CCGTCCTCGTCGATCTCGAGCCCGGTACCATGGATGCCGTCCGTGCTGGTCCCTTCGGTC

AGCTTTTCCGACCCGACAACTTCGTCTTCGGCCAGTCTGGTGCTGGCAACAACTGGGCCA

AGGGTCATTACACCGAGGGTGCCGAGTTGGTCGACCAGGTTCTCGATGTAGTCCGTCGCG

AGGCCGAGGGTTGCGACTGCCTCCAGGGTTTCCAGATCACCCACTCTCTCGGTGGTGGTA

CTGGTGCCGGTATGGGTACTCTGTTGATTTCCAAGATCCGCGAAGAGTTCCCCGACCGCA

TGATGGCCACTTTCTCCGTCATGCCCTCCCCTAAGGTCTCCGACACCGTTGTCGAGCCCT

ACAACGCCACTCTTTCCGTTCACCAGCTTGTCGAGAACTCGGACGAGACCTTCTGCATTG

ACAACGAGGCTCTCTACGATATCTGCATGCGCACCCTCAAGCTGTCCAACCCCTCCTACG

GCGACTTGAACCACCTTGTTTCCGCCGTCATGTCTGGCGTCACCACATGCCTGCGTTTCC

CCGGTCAGCTGAA-----------------------------------------------

------------------------------------------------------------

------------------------------------------------------------

------------------------------------------------------------

------------------------------------------------------------

-------------------------------

>Sarcoxylon_compunctum_CBS_359.61

CTCTGTTTACTTTGCAACCCAATCGATACTCCACCAAATTTTACTACCCGATTCGTGAAC

GCGTCGC-GTC-ATACTTC--GGTTTCAG-CTCTCCACTCGTACACAAAGGAATTG-AAG

TAGC-------------------TGGAAACAATACAGCTTCATGT---ATCTGAAATCGT

CTGGAA----------GAGAAAACTCGGCTAACCACTCTTGCCGCGTCTACAGGTCCACC

TCCAAACCGGCCAGTGCGTAAGTCGTCTCTCGCTCC---GCGATATTAGCATGTTCTCGG

AAAAACTCACATGGAATACTAGGGTAACCAAGTTGGTGCTGCCTTCT-------------

----------------------------------------------GGCAACAAATCTCT

GCGGAGCACGGTCTCGACGGAAATGGCGTGTACGTATATGACATCTGCGTTAAACAACAA

TAACGAATTTATTGATTGACTGTGATGGAACAGGTACAATGGAACTTCCGAGCTCCAGCT

CGAGCGTATGAGCGTCTACTTCAATGAGGTACGCGATCCCTAGTCC--AAATTTTTTCTC

TAGCAAAAATCCTAACATGATTGATTTTACAGGGTGCTGGCAACAAGTTCGTCCCTCGCG

CCGTTCTCGTCGATCTCGAGCCCGGTACCATGGATGCCGTCCGTGCTGGTCCCTTCGGTC

AGCTTTTCCGACCCGACAACTTCGTCTTCGGCCAGTCTGGTGCTGGCAACAATTGGGCCA

AGGGTCACTACACCGAGGGTGCTGAGTTGGTGGACCAGGTTCTCGATGTTGTCCGTCGCG

AGGCTGAAGGCTGCGACTGCCTCCAGGGTTTCCAGATCACCCACTCTCTCGGTGGTGGTA

CCGGTGCCGGTATGGGTACTCTGTTGATCTCCAAGATCCGTGAGGAGTTCCCCGACCGCA

TGATGGCTACCTTCTCCGTCATGCCCTCCCCTAAGGTCTCCGATACCGTTGTCGAGCCCT

ACAACGCCACACTCTCCGTCCACCAGCTGGTCGAGAACTCCGACGAGACCTTCTGCATTG

ACAACGAGGCTCTGTACGATATCTGCATGCGCACCCTTAAGTTGTCCAACCCCTCGTACG

GCGACTTGAACCACCTCGTCTCCGCTGTCATGTCTGGCGTCACCACCTGCCTGCGCTTCC

CTGGTCAGCTGAACTCTGATCTTCGCAAGCTGGCCGTCAACATGGTGCCTTTCCCTCGTC

TCCACTTCTTCATGATTGGCTTTGCTCCTCTCACAAGCCGTGGCGCTCACTCTTTCCGTG

CCATCACCGTTCCCGACTTGACCCAGCAGATGTTCGACCCCAAGAATATGATGGCTGCTG

CTGACTTCCGCAACGGTCGTTACTTGACATGCTCTGCCATCTTGTAAGCACTCGCCCTCT

TCTCCCTGCACGAGCTGCGTCACTAAC-TAGTCACTTCTCTAGCCGTGGTAAGGTTTCCA

TGAAGGAGGTTGAGGACCAGATGCGAAACGT

>Podosordaria_mexicana_WSP176

CTCTGTTTACTTTGCAACCCAACGAATACTCCACCAAATTTTGCTGCCTTAAACTTGAAC

GCGTCGCAATC-ATGCCTC--ACTATCAG-CTCTCCACTCGTGAACGAAGGAGT------

----G-----------GAGTAACTGGAAGCAACACCTCTTTCTGG---CACTTTGACCGT

CGGGAAC---------ACAGATTTTTGGCTAACCAAACTTTTC----TCTTAGGTCCACC

TTCAAACCGGCCAATGCGTGAGTGATAGCTCGCTTC---GTGATCGTGGCATAGT-----

GTAGACTAACACTAAATAATAGGGTAACCAAGTTGGTGCTGCGTTCT-------------

----------------------------------------------GGCAGCAGATCTCT

GCGGAACACGGTCTCGACGGCAGTGGCGTGTATGTGCATTCGGTCAACAGAAGACGCCGA

GAAGGGATCAACTGACTAATTTGATGGAAACAGGTACAATGGAACTTCAGACCTCCAGCT

CGAGCGCATGAGCGTCTATTTCAATGAGGTATGCAGTT----GTCA--GTCTGTTTCTTG

CAGGAAATTTACTAATTTGATAATCGATACAGGGTGCTGGCAACAAATTCGTCCCTCGCG

CCGTTCTCGTCGATCTCGAGCCCGGTACCATGGACGCTGTCCGTGCTGGTCCCTTCGGTC

AGCTCTTCCGACCCGACAACTTCGTCTTCGGCCAGTCTGGTGCCGGCAACAACTGGGCCA

AGGGCCATTACACTGAGGGTGCTGAGTTGGTGGACCAGGTTCTCGACGTCGTCCGCCGTG

AGGCTGAAGGCTGCGACTGCCTGCAGGGTTTCCAGATCACCCACTCTCTCGGTGGTGGAA

CTGGTGCCGGTATGGGTACTCTGTTGATCTCCAAAATCCGCGAGGAGTTCCCCGACCGCA

TGATGGCAACTTTCTCCGTCATGCCTTCCCCCAAGGTCTCCGACACCGTCGTCGAGCCTT

ATAACGCCACACTCTCGATCCACCAGCTGGTCGAGAACTCGGATGAGACCTTCTGCATTG

ACAACGAGGCCTTGTACGACATCTGCATGCGCACCCTGAAGTTGTCCAACCCCTCGTACG

GCGATCTGAATCACCTCGTCTCCGCTGTCATGTCTGGCGTCACCACCTGCCTGCGCTTCC

CCGGTCAGCTGAACTCTGACCTGCGCAAGCTGGCCGTCAACATGGTGCCTTTCCCTCGTC

TCCACTTCTTCATGATTGGCTTCGCCCCCCTCACAAGTCGTGGCGCGTACTCTTTCCGTG

CTGTCACTGTCCCCGACTTGACACAGCAGATGTTCGACCCCAAGAACATGATGGCAGCTG

CTGACTTCCGTAACGGTCGTTATCTCACATGCTCCGCGATCTTGTAAGTTTTCACTTTTT

TGCGCTTCAGCGAGCGGCGTTGCTAACTTTTTACCCTTACCAGCCGTGGCAAGGTTTCTA

TGAAGGAAGTTGAGGACCAGATGCGCAACGT

>Poronia_punctata_CBS_656.78

CTCTGTTTACTTTGCAACCCAATCAC-GACCTGCCAAATTTTGCTGCCCTATTGTCCGAC

GCGTCCAAATAC--GCTTTACGTTCTCTGCCTGTTTACAG----GTATATGCATCGGCTT

CATAG---------------A----GAACCAGCGGAGTTTCATCAAGCATATGAATCTA-

----------------GAAGAGAGCATGTTAAGCTAATTTTTTTCAATTACAGGTGCACC

TCCAAACCGGCCAATGCGTAAGTCGTCTGCCGCCACCGA----TAAT-ATGCGAAGTAGC

CGAAACTCATTAGATAT--TAGGGTAACCAAGTTGGTGCTGCTTTCT-------------

----------------------------------------------GGCAACAAATCTCC

GGGGAGCACGGTCTCGATGGCAATGGCGTGTATGTGTTTGGAAACTTCCCAATCACAAGG

TTCCGGATCGGTTGATTGACTGATGCAAAATAGTTACAACGGAAACTCGGAGCTGCAGCT

CGAGCGCATGAGCGTCTACTTCAATGAGGTACGTGGTT--------AT----ATCCGGTG

GAAACATTTTTCTGACTTGAT-GGTTGCGCAGGGTGCCGGCAACAAGTACGTCCCCCGCG

CCGTCCTGGTCGATCTCGAGCCCGGTACGATGGATGCTGTCCGTGCTGGTCCTTTCGGTC

AGCTTTTCCGACCCGACAACTTTGTTTTCGGCCAGTCCGGTGCTGGCAACAACTGGGCCA

AGGGTCATTACACCGAAGGTGCCGAGTTGGTGGACCAGGTTCTCGATGTTGTCCGTCGCG

AAGCTGAGGGCTGCGATTGCCTTCAGGGTTTCCAGATCACCCACTCCCTCGGTGGTGGTA

CCGGTGCCGGCATGGGTACGCTGTTGATCTCCAAGATCCGTGAGGAGTTCCCCGACCGAA

TGATGGCCACCTTCTCCGTCATGCCCTCCCCTAAGGTCTCTGACACCGTTGTCGAGCCCT

ACAACGCGACCCTCTCCGTACACCAGCTTGTCGAGAACTCCGACGAGACCTTCTGTATCG

ACAACGAGGCTCTATACGACATCTGCATGCGCACGCTCAAGCTGTCGAACCCTTCGTACG

GTGACCTGAACCACCTCGTATCCGCCGTCATGTCCGGTGTCACCACCTGCCTGCGTTTCC

CTGGTCAGCTGAACTCTGATCTGCGCAAGCTGGCTGTCAACATGGTGCCCTTCCCTCGTC

TCCATTTCTTCATGGTTGGCTTTGCTCCTCTTACCAGCCGTGGCGCTCACTCTTTCCGTG

CCGTCACCGTTCCCGAGTTGACACAGCAAATGTTCGACCCCAAGAACATGATGGCTGCTG

CTGATTTCCGTAACGGCCGTTACCTCACATGCTCTGCCATCTTGTAAGCATATAAAGATC

CAATCCAGTTGAAAACAGGTTACTAATC--------------------------------

-------------------------------

>Entalbostroma_erumpens_ICMP_21152

CTCTGTCTACTTTGCAACCCAATCGA-TCTCCAACCATTTCTCCTACCTTGGACTACGGC

GCGTTCCGACCTAATCCTCTTGATGTCAACCCCTCCAGATCTACACACAAG---AACTTG

TCGTGGACAGTTTGG-TGGTGACTGTTAATATCATGGTTCTTTCATATCTACCATGTGGC

CCAAAA----TTGTACATGAAAACAGAGCTAACCAAATCCTCCCTCTTGACAGGTTCACC

TCCAAACCGGCCAATGCGTACGTCGCTTTACGATGCTCAATGACGAGTCCCGAGATCTCC

CCGTACTCACATAATATCACAGGGTAACCAAATTGGTGCTGCTTTCT-------------

----------------------------------------------GGCAACAAATTTCT

GGCGAGCACGGTCTCGATGGCAGTGGCGTGTATGTTGATCATCCCCAAGAAACAACATGA

CAACGGAATGGTCGACTGATGACTTTGAAACAGTTACAACGGTACCTCTGAGCTCCAGCT

CGAGCGTATGAGCGTCTACTTCAACGAGGTACGCGACTATCGAGTCATGTTGACTCTGTG

CAATCAACGTTCTAATGTGGG-GCTTTTCTAGGGTGCCGGTAACAAGTATGTTCCTCGCG

CTGTCCTCGTCGATTTGGAGCCCGGTACCATGGATGCTGTCCGTGCCGGTCCCTTCGGTC

AGCTCTTCCGCCCCGACAACTTCGTCTTCGGTCAGTCCGGTGCCGGCAACAACTGGGCCA

AGGGTCATTACACGGAAGGTGCTGAGCTCGTCGACCAAGTTCTCGATGTCGTCCGTCGTG

AGGCCGAGGGCTGTGACTGCCTCCAGGGTTTCCAAATCACCCACTCGCTCGGCGGTGGTA

CCGGTGCTGGTATGGGTACGCTGCTGATCTCCAAGATCCGTGAGGAGTTCCCCGACCGCA

TGATGGCCACCTTCTCCGTCATGCCTTCTCCCAAGGTTTCCGACACCGTTGTCGAGCCTT

ACAACGCCACCCTCTCGGTCCACCAGCTGGTCGAGAACTCCGATGAGACCTTCTGTATCG

ATAACGAGGCTCTGTACGATATCTGCATGCGTACCCTGAAGCTCTCCAACCCTTCGTACG

GTGACCTGAACCACCTCGTCTCCGCTGTCATGTCTGGCGTGACCACCTGTCTGCGCTTCC

CCGGTCAGCTTAACTCTGATCTGCGCAAGTTGGCCGTCAACATGGTGCCCTTCCCTCGTC

TGCATTTCTTCATGGTGGGCTTTGCTCCTCTCACCAGCCGTGGTGCTCACTCTTTCCGTG

CCGTCACGGTTCCCGAGTTGACCCAGCAAATGTTTGACCCTAAGAACATGATGGCCGCCG

CTGACTTCCGTAACGGTCGTTACCTGACATGCTCTGCTATTTTGTAAGAAGCTGCTTTTT

TATTCTTTTGTATGATGAAATACTAACCTCTCTT-TTTACCAGCCGTGGCAAGGTTTCCA

TGAAGGAAGTCGAGGACCAGATGCGCAACGT

>Induratia_apiospora_ATCC_60639

------------------CCAAT----------------TTTATTGGGCAGTCCCCGAAC

GCGTCCGAG---A-TGCCCCTGAAT-CTGCCCCTCCAC--------ACACACAA----AG

CAGCACCGAGACAGCT------CCGCCAGCACCAGAGCAT-GTCC---CCGTCGAAA--T

ACACCCGAAAGA-TG-AG--AATCGCTACTAACTGTGTGTTTCGTATTCATAGGTTCACC

TTCAAACCGGCCAATGCGTAAGTATCTCTTCGACCGCCGACTATCGCGCTGGGAATATTG

CGGGGCTCACATAATTTCGCAGGGTAACCAAATTGGTGCCGCCTTCTGGTGTGTACTTAC

GGCATACAACAGGGCTTGCTGCATGAATATTGACATCTTGGTTTTAGGCAAACCATCTCT

GGCGAGCACGGCCTCGACAGCAATGGCACGTATGTACCCTATCACATCCC----CGC---

TGAT-CGGCTT-GGACTGACCGCCTTTGAACAGCTACAACGGAACCTCCGAGCTCCAACT

CGAACGCATGAGCGTCTACTTCAACGAGGTATGCAATTGTTTAATCA-TGAACTTAGAGC

ATCAAGAATACCTAACATAGTTGCATTCATAGGGCTCCGGCAACAAGTATGTTCCTCGCG

CTGTCCTCGTTGATCTCGAGCCCGGTACCATGGACGCAGTCCGCGCTGGTCCCTTCGGTC

AACTCTTCCGCCCCGACAACTTCGTCTTCGGCCAGTCTGGTGCCGGCAACAACTGGGCCA

AGGGTCACTACACTGAGGGTGCTGAGCTTGTCGATACCGTTCTCGATGTTGTCCGTCGCG

AGGCTGAGGGCTGCGACTGCCTTCAGGGATTCCAGATCACCCACTCGCTCGGTGGTGGTA

CCGGTGCGGGTATGGGTACCTTGCTGATCTCCAAGATTCGTGAGGAGTTCCCCGACCGAA

TGATGGCCACCTTCTCCGTCGTTCCCTCCCCTAAGGTCTCCGACACTGTTGTCGAGCCCT

ACAACGCTACCCTCTCCGTCCACCAGCTGGTCGAGAACTCCGACGAGACCTTCTGCATTG

ACAATGAGGCTCTGTACGACATTTGCATGCGAACTTTGAAGCTATCCAACCCCTCATACG

GCGATCTGAACCACCTGGTCTCCGCCGTTATGTCTGGTGTCACCACTTGCCTACGCTTAC

CCGGCCAGCTCAACTCCGACCTCCGCAAGCTGGCTGTCAAT-------------------

------------------------------------------------------------

------------------------------------------------------------

------------------------------------------------------------

------------------------------------------------------------

-------------------------------

>Barrmaelia_rhamnicola_BR

CTCTGTTTACCACGGAGCCCAATGAC----------------ACCGGGCGATCCCTGGAC

GCGCTCG-GC--AGTACCCCTGATTTCTGCCCCTCCAC--------GC----AA----CG

CAGCAAAGGGAGAGCT-----TCTGCCAGCAGCAGAGCATCGTCC---TCGTCGAGA--T

TTACCAGCAACATTGGGAGCGAGCACTGCTAACCGTGTCTCTCTCAATTATAGGTTCACC

TCCAGACCGGCCAATGCGTAAGTCTCTCCTCGACCGCCGACTATCGCGCTGGGAATATTG

CGGGGCTCACACAATCACACAGGGTAACCAAATCGGTGCCGCCTTCTGGTGTGTACCTAC

GGCTTGCAACGCCATTTCCGGTATGAATGTTGACTCTTGCAATTTAGGCAAACCATCTCT

GGCGAGCACGGCCTCGACAGCAATGGAGTGTACGTAGCACAAGTCTGCCC---TCGACCA

TAACTCGAATCGAGACTGACCGCCTGTCAACAGCTATAATGGAACCTCCGAGCTCCAGCT

TGAGCGCATGAGCGTCTACTTCAACGAGGTATGCAATCGTCAATTGATTGTCCTAGTCTG

GCACGGCACAGCTAACGCAATTCCAATGACAGGCTTCCGGCAACAAGTATGTTCCTCGCG

CCGTCCTCGTCGATCTCGAGCCCGGTACCATGGACGCCGTCCGTGCTGGCCCCTTCGGTC

AACTCTTCCGACCCGACAACTTCGTCTTCGGGCAATCCGGTGCTGGAAACAACTGGGCCA

AGGGTCACTACACTGAGGGTGCTGAGCTTGTTGACCAGGTTCTGGATGTTGTCCGACGCG

AGGCCGAGGGCTGCGACTGCCTTCAGGGCTTCCAGATCACCCACTCGCTGGGTGGTGGTA

CCGGTGCTGGTATGGGTACTTTGCTGATCTCCAAGATCCGCGAGGAGTTCCCTGACCGCA

TGATGGCCACCTTCTCCGTCGTCCCCTCCCCCAAGGTCTCTGACACCGTCGTCGAGCCTT

ACAACGCCACCCTCTCGGTCCACCAGCTGGTCGAGAACTCGGACGAGACCTTCTGCATTG

ACAACGAGGCTCTGTACGACATCTGCATGCGCACTCTCAAGCTGTCCAACCCCTCGTACG

GTGACCTGAACCACCTCGTCTCTGCTGTCATGTCTGGCGTCACCACCTGTCTGCGATTCC

CTGGCCAGCTCAACTCCGACCTGCGCAAGCTGGCCGTGAACATGGTTCCCTTCCCTCGTC

TCCACTTCTTCATGGTTGGCTTCGCTCCCCTAACCAGCCGTGGTGCTTACTCTTTCCGTG

CTGTCACCGTTCCCGAGTTGACCCAGCAGATGTTCGACCCCAAGAACATGATGGCTGCCT

CCGACTTCCGCAACGGTCGCTATCTCACATGCTCTGCCATCTTGTAAGAAGAACTGTCCC

CTTGTTGGGTATAGCCTCGGAGCTAACT-------TTCTCTAGCCGCGGCAAGGTCTCCA

TGAAGGAGGTCGAGGACCAGATGCGAAACGT

>Barrmaelia_macrospora_CBS_142768

CTCTGTTTACCACGGAGCTCAATGCCATAATG-CC-------ACCGGGCGATCCCTGGAC

GCGCTCG-GC--AGCACCCCTGATTTCTGCCCCTCCAC--------ACTTGGAA----TG

CAGCAAAGGGAGAGCT-----TCTGCCAGCAGCAGAGCATCGTCC---TCGTCGAGA--T

TTACCAGCAACATTG-GG---ATCACTGCTAACCGTGTCTCTCTCAATTATAGGTTCACC

TCCAGACCGGCCAATGCGTAAGTCTTTCCTCGACCACCGACTATCGCGCTGGGAATGTTG

CGGGGCTCACACGATTTCACAGGGTAACCAAATTGGTGCCGCCTTCTGGTGTGTACCTAC

GGCTTGCAACACCATTTCCGGCATGAATGTTGACTCTTGCGTTTCAGGCAAACCATCTCG

GGCGAGCACGGCCTCGACAGCAATGGAGTGTACGTAGCACGAGTCTGCCC---TCGACCA

--ACTCAAACCGAGACTGACCGCCTGTCAACAGCTACAACGGAACCTCCGAGCTCCAGCT

TGAGCGCATGAGCGTCTACTTCAACGAGGTATGCAACCGTCAATTGACTGCCCTAGTCTG

GCACGGCACAGCTAACGCAATTCCAATGACAGGCTTCCGGCAACAAGTATGTTCCTCGCG

CCGTCCTCGTCGATCTCGAGCCCGGTACCATGGACGCTGTCCGTGCTGGTCCCTTCGGTC

AACTCTTCCGACCCGACAACTTCGTCTTCGGGCAATCCGGTGCTGGAAACAACTGGGCCA

AGGGTCACTACACTGAGGGTGCTGAGCTTGTTGACCAGGTTCTGGATGTTGTCCGACGCG

AGGCCGAGGGCTGCGACTGCCTTCAGGGCTTCCAGATCACCCACTCGCTGGGTGGTGGTA

CCGGTGCCGGTATGGGTACTTTGCTGATCTCCAAGATCCGCGAGGAGTTCCCTGACCGCA

TGATGGCCACCTTCTCTGTCGTCCCCTCCCCCAAGGTCTCTGACACCGTCGTCGAGCCTT

ACAACGCCACCCTCTCGGTCCACCAGCTGGTCGAGAACTCGGACGAGACCTTCTGCATTG

ACAACGAGGCTCTGTACGACATCTGCATGCGCACTCTCAAGCTGTCCAACCCCTCGTACG

GTGACCTGAACCACCTCGTCTCTGCTGTCATGTCTGGCGTCACCACCTGTCTGCGATTCC

CTGGCCAGCTCAACTCCGACCTGCGCAAGCTGGCCGTGAACATGGTTCCCTTCCCCCGTC

TCCACTTCTTCATGGTTGGATTCGCTCCCCTAACAAGCCGTGGTGCTTACTCTTTCCGTG

CTGTCACCGTTCCCGAGTTGACCCAGCAGATGTTCGACCCCAAGAACATGATGGCTGCCT

CCGACTTCCGCAACGGTCGCTACCTCACATGCTCTGCCATCTTGTAAGAAGAACTGTCCC

CTTGTTGGGTATAGCCTCGGAGCTAACT-------TTTTCTAGCCGTGGCAAGGTCTCCA

TGAAGGAGGTCGAGGACCAGATGCGAAACGT

>Barrmaelia_rappazii_CBS_142771

CTCTGTTTACCACGGAGCCCAATGCC----------------ACCGGGCGTTCCCTGGAC

GCGCTCG-GC--AGTACCCCTGATTTCTGCCCCTCCAC--------ACTTGGAA----TG

CAGCAACGGGAGAGCT-----TCTGCCAGCAGCAGAGCATCGTCC---TCGTCAAGA--T

TCACCAGGAACCTTGGGG--AATCGCAGCTAATCGTGTCTCTCTCAATTATAGGTTCACC

TCCAGACCGGCCAATGCGTAAGTCTCTCCTCGACCACCGACTATCGCGCTGGGAATGTTG

CGGGGCTCACACAATTACACAGGGTAACCAAATCGGTGCCGCCTTCTGGTGTGTACCTAC

GGCTTGCAACACCATTTCCGGCATGAATGTTGACTCTTGCATTTCAGGCAAACCATCTCT

GGCGAGCACGGCCTCGACAGCAATGGAGTGTACGTAGCACGAATCTGCCC---TCGACCA

TAACTCGAATCGAGACTGACCGCCTGTCAACAGCTACAACGGAACCTCCGAGCTCCAGCT

TGAGCGCATGAGCGTCTACTTCAACGAGGTATGCAACCGTCAATTGACTGCCCTAGTCTG

GCACGGCACAGCTAACGCAATTCCAATGACAGGCTTCCGGCAACAAGTATGTTCCTCGCG

CCGTCCTCGTCGATCTCGAGCCCGGTACCATGGACGCCGTCCGTGCTGGTCCCTTCGGTC

AACTCTTCCGACCCGACAACTTCGTCTTCGGACAATCCGGTGCTGGAAACAACTGGGCCA

AGGGTCACTACACTGAGGGTGCTGAGCTTGTTGACCAGGTTCTGGATGTCGTCCGACGCG

AGGCCGAGGGCTGCGACTGCCTTCAGGGCTTCCAGATCACCCACTCGCTGGGTGGTGGTA

CCGGTGCCGGTATGGGTACTTTGCTGATCTCCAAGATCCGCGAGGAGTTCCCCGACCGCA

TGATGGCCACCTTCTCCGTCGTCCCCTCCCCCAAGGTCTCTGACACCGTCGTCGAGCCTT

ACAACGCCACCCTCTCGGTCCACCAGCTGGTCGAGAACTCGGACGAGACCTTCTGCATTG

ACAACGAGGCTCTGTACGACATCTGCATGCGCACTCTCAAGCTGTCCAACCCCTCGTACG

GTGACCTGAACCACCTCGTCTCTGCTGTCATGTCTGGCGTCACCACCTGTCTGCGATTCC

CTGGCCAGCTCAACTCCGACCTGCGCAAGCTGGCCGTGAACATGGTTCCCTTCCCTCGTC

TCCACTTCTTCATGGTTGGCTTCGCTCCCCTAACCAGCCGTGGTGCTTACTCTTTCCGTG

CTGTCACCGTTCCCGAGTTGACCCAGCAGATGTTCGACCCCAAGAACATGATGGCTGCCT

CCGACTTCCGCAACGGTCGCTACCTCACGTGCTCTGCCATCTTGTAAGAAGAACTGTCCC

CTTGCTGGGTATAGCCTCCGAGCTAACT-------TTCTCTAGCCGCGGCAAGGTCTCCA

TGAAGGAGGTCGAGGACCAGATGCGAAACGT

>Barrmaelia_oxyacanthae_CBS_142770

CTCTGTTTACCACCGAGCTCAATGCC----------------GCCGGGCGATCCCTGGAC

GCGCTCA-GC--AGTACCCCTGATTTCTGCCCCTCCAC--------ACTTGGAA----TG

CAGCAAAGGGAGAGCT-----T---CCAGCAGCAGAGCATCGTCC---TCGTCAAGA--T

TCACCAGGAACC--GGGG--AATCACAGCTAATCGAGTCTCTCTCAATTACAGGTTCACC

TCCAGACCGGCCAATGCGTAAGTCTCTCCTCGACCACCGACTATCGCGCTGGGAATGTTG

CGGAGCTCACACAATTACACAGGGTAACCAAATCGGTGCCGCCTTCTGGTGTGTACCTAC

GGCTTGCAACACCATTTCCGGCATGAATGTTGACCATTGCGTTTCAGGCAAACCATCTCT

GGCGAGCACGGCCTCGACAGCAATGGAGTGTACGTAACACGAGTCTGCCC---TCGACCA

TAACTCGAATCGAGACTGACAGCCTGTCAACAGCTACAACGGAACCTCCGAGCTCCAGCT

TGAGCGCATGAGCGTCTACTTCAACGAGGTATGCAATCGTCAATTGACTGTCCTAGTCTG

GCACGGCACAGCTAACGCAATTCCAATGGCAGGCTTCCGGCAACAAGTATGTTCCCCGCG

CCGTCCTCGTCGATCTCGAGCCCGGTACCATGGACGCCGTCCGTGCTGGTCCCTTCGGTC

AACTCTTCCGACCCGACAACTTCGTCTTCGGACAATCCGGTGCTGGAAACAACTGGGCCA

AGGGTCACTACACTGAGGGTGCTGAGCTTGTTGACCAGGTTCTGGATGTCGTCCGACGCG

AGGCCGAGGGCTGCGACTGCCTTCAGGGCTTCCAGATCACCCACTCGCTGGGTGGTGGTA

CCGGTGCCGGTATGGGTACTTTGCTGATCTCCAAGATCCGCGAGGAGTTCCCTGACCGCA

TGATGGCCACCTTCTCCGTCGTCCCCTCCCCCAAGGTCTCTGACACCGTCGTCGAGCCTT

ACAACGCCACCCTCTCGGTCCACCAGCTGGTCGAGAACTCGGACGAGACCTTCTGCATTG

ACAACGAGGCTCTGTACGACATCTGCATGCGCACTCTCAAGCTGTCCAACCCCTCGTACG

GTGACCTGAACCACCTCGTCTCTGCTGTCATGTCTGGCGTCACCACCTGTCTGCGGTTCC

CTGGCCAGCTCAACTCCGACCTGCGCAAGCTGGCCGTGAACATGGTTCCCTTCCCTCGTC

TCCACTTCTTCATGGTTGGCTTCGCTCCCCTAACCAGCCGTGGTGCTTACTCTTTCCGTG

CTGTCACCGTTCCCGAGTTGACCCAGCAGATGTTCGACCCCAAGAACATGATGGCTGCCT

CCGACTTCCGCAACGGTCGCTACCTCACATGCTCTGCCATCTTGTAAGAAGAACTGTCCC

CTTGTTGGGTATAG--TCGGAGCTAACT-------TTTTCCAGCCGTGGCAAGGTCTCCA

TGAAGGAGGTCGAGGACCAGATGCGAAACGT

>Barrmaelia_moravica_CBS_142769

CTCTGTTTACCACGGAGCCCAATGCC-----------------TCGGGCGATCCCTGGAC

GCGCTCG-GC--AGTACCCCTGATTTCTGCCCCTCCAC--------ACTTGGAA----TG

CAGGAACGGGAGAGCT-----TCTGCCAGCAGCAGAGCATCGTCC---TCGTCAAGA--T

TCACCAGGAACCTTGGGG--AATCACAGCTAATGGTGTCTCTCTTAATTATAGGTTCACC

TCCAGACCGGCCAATGCGTAAGTCTCCCCTCGACCGCCGACTATCGCGCTGGGAATGTCG

TGGGGCTCACATAATTACACAGGGTAACCAAATCGGTGCCGCCTTCTGGTGTGTACCTAC

GACTTGCAACACCATTTCCGGCATGAATGTTAATTCTGGCATTTCAGGCAAACCATCTCT

GGCGAGCACGGCCTCGACAGCAATGGAGTGTACGTAGCACAAGTCTGTCC---TCGACCA

TGCCTCGAATCGAGACTGACCGCCTGTCAACAGCTATAATGGAACCTCCGAGCTCCAGCT

TGAGCGCATGAGCGTCTACTTCAACGAGGTATGCAATCGTCAATTGACTGTCCTAGTCTG

CCACGGCACAGCTAACGCAATTCCAATGGCAGGCTTCCGGTAACAAGTATGTTCCTCGCG

CCGTCCTCGTCGATCTCGAGCCCGGTACCATGGACGCCGTCCGTGCTGGTCCCTTCGGTC

AACTGTTCCGACCCGACAACTTCGTCTTCGGACAATCCGGTGCTGGAAACAACTGGGCCA

AGGGTCACTACACTGAGGGTGCTGAGCTTGTTGACCAGGTTCTGGATGTCGTCCGACGCG

AGGCCGAGGGCTGCGACTGCCTTCAGGGCTTCCAGATCACCCACTCGCTGGGTGGTGGTA

CCGGTGCCGGTATGGGTACTTTGCTGATCTCCAAGATCCGCGAGGAGTTTCCCGACCGCA

TGATGGCCACCTTCTCCGTCGTCCCCTCCCCCAAGGTCTCTGACACCGTCGTCGAGCCTT

ACAACGCCACCCTCTCTGTCCACCAGCTGGTCGAGAACTCGGACGAGACCTTCTGCATTG

ACAACGAGGCTCTGTACGACATCTGCATGCGCACTCTCAAGCTGTCCAACCCCTCGTACG

GTGACCTGAACCACCTCGTCTCTGCTGTCATGTCTGGCGTCACCACCTGTCTGCGATTCC

CTGGCCAGCTCAACTCCGACCTGCGCAAGCTGGCCGTGAACATGGTTCCCTTCCCTCGTC

TCCACTTCTTCATGGTTGGCTTCGCTCCCCTAACCAGCCGTGGTGCTTACTCTTTCCGTG

CTGTCACCGTTCCCGAGTTGACCCAGCAGATGTTCGACCCCAAGAACATGATGGCTGCCT

CCGACTTCCGCAACGGTCGCTACCTCACATGCTCTGCCATCTTGTAAGAAGAACTGTCCC

CTTGCTGGGTATAGCCTCGGAGCTAACT-------TTCTCTAGCCGTGGCAAGGTCTCCA

TGAAGGAGGTCGAGGACCAGATGCGAAACGT

>Entosordaria_perfidiosa_CBS_142773

CTCTGTTTACCCCGGAGCCCAACCGAGCGAGCACCTCCAA-----------TCCCTGGAC

GCGTCCGAGCCAAGTACCCCTGATTTCTACCCCTCCAC--------ACACACAG----CG

CAGCAACGAGAGAGCT--------GCCAGCACCAGAGCATCGTCC---GAGTTAAAA--T

ACACCAGGAACATTG-GG---ACCACTGCTGACCGTGTCGTTCTCAATTATAGGTTCACC

TTCAGACCGGCCAATGCGTAAGTCTCTCCTCGACCGCCGACTATCGCGCTGGGAATATTG

CGGGGCTCACACAATTTCACAGGGTAACCAAATCGGTGCCGCCTTCTGGTGTGTACCTAC

GGCATGCAACACGACCGCCGGCATGAATATTGACT-TTGCGTTTCAGGCAAACCATCTCT

GGCGAGCACGGTCTCGACAGCAATGGAGTGTACGTATCCTGGCTCTGCAC---TCGCCTG

GCATTCGAATTGGGACTGACCGCCTGTAAACAGCTACAACGGAACTTCCGAGCTCCAGCT

CGAGCGCATGAGCGTCTACTTCAACGAGGTATGCAATTGTCAATTGGTTGTCCTAGTCTG

GCCCGGCACAGCTAACACAACTTCAATGGCAGGCTTCCGGCAACAAGTATGTTCCTCGCG

CTGTCCTCGTCGATCTCGAGCCCGGTACCATGGACGCCGTCCGTGCTGGTCCCTTCGGTC

AACTCTTCCGACCCGACAACTTCGTTTTCGGTCAATCCGGTGCTGGAAACAACTGGGCCA

AGGGTCACTACACCGAGGGTGCTGAGCTTGTTGACCAAGTTCTGGACGTCGTCCGCCGTG

AGGCTGAGGGCTGCGACTGCCTCCAGGGTTTCCAGATCACCCACTCGCTCGGTGGTGGTA

CCGGTGCCGGTATGGGTACTTTACTGATCTCCAAGATCCGTGAGGAGTTCCCCGACCGCA

TGATGGCCACCTTCTCCGTCGTCCCCTCCCCCAAGGTCTCCGACACCGTTGTCGAGCCCT

ACAACGCTACCCTCTCGGTCCACCAGCTGGTCGAGAACTCGGACGAGACCTTCTGCATTG

ACAACGAGGCTCTATACGACATCTGCATGCGCACTCTCAAGCTGTCCAACCCCTCGTACG

GTGACCTGAACCACCTGGTCTCTGCTGTCATGTCTGGCGTCACCACTTGCCTGCGATTCC

CTGGCCAACTCAACTCCGACCTGCGCAAGCTGGCTGTGAACATGGTTCCCTTCCCTCGTC

TTCACTTCTTCATGGTTGGCTTTGCTCCTCTGACCAGCCGTGGTGCTCACTCTTTCCGTG

CTGTCACCGTTCCCGAGTTGACCCAGCAGATGTTCGACCCCAAGAACATGATGGCTGCGT

CCGACTTCCGCAACGGTCGCTACCTCACATGCTCTGCCATCTTGTAAGACGCTCTGCCCC

TTT-TCGGTCATGACCTGAAAGCTAACT---CG--TTCCCTAGCCGTGGCAAGGTCTCCA

TGAAGGAGGTCGAGGACCAGATGCGCAACGT

>Entosordaria_quercina_CBS_142774

CTCTGTTTACCCCGGAGCCCAACCGAGCGAGCACCTCCAATTACCGG-GTTTCCCTGCAC

GCGTCCGAGCCAAGTACCCCTGATTTATGCCCCTCCAC--------ACACACAA----CG

CAGCAACAAGAGAGCT-----GCAGCCAGCACCGGAGCACCGTCC---GAGTCGAAA--T

ACACCAGAAACATTG-GG---ACCAGTGCTGACCGTGTCTTTCTCAATTATAGGTTCACC

TTCAGACCGGCCAATGCGTAAGTCTCTCCTCGACCGCCGACTATCGCGCTGGGAATATTG

CGGGGCTCACACAATTTCACAGGGTAACCAAATCGGTGCCGCTTTCTGGTGTGTACCTAC

GGCATGCAACACGACCGCCGGCATGAATATTGACTGTTGCGTTTCAGGCAAACCATCTCT

GGCGAGCACGGCCTCGACAGCAATGGAGTGTACGTATCCTGGGTCTGCAC---TCGCCTC

GGATTCGAATT-GGACTGACCGCCTGTGAACAGCTACAACGGAACTTCCGAGCTCCAGCT

CGAGCGCATGAGCGTTTACTTCAACGAGGTATGCGATTGTCAATTGGCTGTCCTAGTATG

GTCTGGCATAGCTAACGCAATTTCAACGGCAGGCTTCCGGCAACAAGTATGTTCCTCGCG

CTGTCCTCGTCGATCTCGAGCCCGGTACCATGGACGCCGTTCGTGCTGGTCCCTTCGGTC

AACTCTTCCGACCCGACAACTTCGTTTTCGGTCAATCCGGTGCTGGAAACAACTGGGCCA

AGGGTCACTACACCGAGGGTGCTGAGCTTGTTGACCAAGTTCTGGACGTCGTCCGTCGTG

AGGCTGAGGGCTGCGACTGCCTGCAGGGTTTCCAGATCACCCACTCGCTCGGTGGTGGTA

CCGGTGCCGGTATGGGTACTTTGCTGATCTCCAAGATCCGTGAGGAGTTCCCCGACCGCA

TGATGGCCACCTTCTCCGTCGTCCCCTCCCCCAAGGTTTCCGACACCGTTGTCGAGCCCT

ACAACGCCACCCTCTCGGTCCACCAGCTGGTCGAGAACTCGGACGAGACTTTCTGCATTG

ACAACGAGGCCCTGTACGACATCTGCATGCGCACTCTCAAGCTATCCAACCCCTCGTACG

GTGACCTGAACCACCTGGTCTCTGCTGTCATGTCTGGCGTCACCACTTGCCTGCGTTTCC

CTGGCCAGCTCAACTCCGACCTGCGCAAGCTGGCTGTGAACATGGTTCCCTTCCCTCGTC

TCCACTTCTTCATGGTTGGATTTGCTCCTCTGACCAGCCGTGGTGCTCATTCTTTCCGTG

CTGTCACCGTTCCCGAGTTGACCCAGCAGATGTTCGACCCCAAGAACATGATGGCTGCCT

CCGACTTCCGCAATGGTCGCTACCTCACATGCTCTGCCATCTTGTAAGACGCTCTGCCCC

CTT-TTGGTCATGACCTGAAAGCTAACT---CG--TTCCCTAGCCGTGGCAAGGTCTCCA

TGAAGGAGGTCGAGGACCAGATGCGCAACGT

>Xylaria_apoda_HAST_90080804

CTCTGTTTACTTTGTAACCCAATCAAAGCCCCGACTTTTTTTGCTGCCTTATCCCTGAAC

GCGTCCCCTCCCATGCCCCTTAATTCTAGCCTCTCCACATCTATATGCAAGCCTCTTTGG

CAACTCGCAATGCGGTCTATGCCTGCCAATAACGCGGCTTCATGAAATCTGCCAGGTTAC

CCAGAGAAATGTCAACATGAGAATCGCGCTAACTGGGTTC-TCCCCTCTATAGGTTCACC

TCCAAACCGGCCAATGCGTAGGTCGCCC--CGATCCTCTATAGCGACGTCGAGAAACCCC

CGAGGCTCACATGGCATGACAGGGTAACCAAATCGGTGCTGCTTTCT-------------

----------------------------------------------GGCAACAAATCTCT

GGCGAGCACGGTCTCGATGGCAGTGGCGTGTACGTCTGGTCT--TAATGG---ATGGCGA

CGACACAATGGTCAACTAACATAGATGCAATAGCTACAATGGAACCTCTGAGCTCCAGCT

CGAGCGCATGAGCGTTTACTTCAACGAGGTAGCTTACTACTAGACCGCTTTTCCCCCCTA

CGACCAGCATTCTAATGTGTCGAGTTATCCAGGGCGCCGGTAACAAATATGTCCCCCGTG

CCGTTCTCGTCGATTTGGAGCCCGGTACCATGGATGCCGTCCGTGCGGGTCCCTTCGGTC

AGCTCTTCCGACCCGACAACTTTGTCTTCGGCCAGTCTGGTGCTGGCAACAACTGGGCCA

AGGGTCATTACACTGAGGGTGCTGAGCTGGTTGACAACGTTCTTGATGTTGTTCGTCGCG

AGGCTGAGGGCTGCGACTGCCTCCAGGGCTTCCAGATCACCCACTCGCTCGGTGGTGGCA

CCGGTGCCGGTATGGGTACTCTGTTGATCTCCAAGATTCGTGAGGAGTTCCCTGACCGTA

TGATGGCCACCTTCTCCGTCATGCCCTCGCCCAAGGTCTCCGACACTGTCGTCGAGCCCT

ACAACGCCACCCTTTCCGTCCACCAGCTGGTCGAGAACTCCGATGAGACTTTCTGCATTG

ACAACGAGGCTCTCTACGACATCTGCATGCGCACCCTGAAGCTATCCAACCCTTCGTACG

GTGACCTGAACCACCTTGTCTCCGCTGTCATGTCTGGTGTTACCACCTGCTTGCGTTTCC

CCGGTCAGCTTAACTCTGATCTGCGCAAGTTGGCTGTCAACATGGTGCCCTTCCCTCGTC

TGCACTTCTTCATGGTCGGCTTTGCGCCTCTCACTAGTCGTGGTGCTCACTCTTTCCGTG

CTGTCACGGTTCCCGAGTTGACCCAGCAAATGTTCGACCCCAAGAACATGATGGCCGCCG

CTGACTTCCGCAACGGTCGTTACTTGACATGCTCCGCTATCTTGTAAGAACCATCCTCTC

CCCCTTCAGCATATGTCCATCGCTAATTTGGTACCCTCACTAGCCGTGGCAAGGTTTCCA

TGAAGGAAGTTGAGGACCAGATGCGAAACGT

>Nemania_primolutea_HAST_91102001

CTCTGTTTACTTTGCAACCCAATCAA-GCTCCCTCATTTTTTGCTACCCTATCCCTGAAC

GCGTCCC--CG-ACGCCCCTCGCTATTAA----TCCTCATCTCTACATGGGCATCGTCCA

CTGTAAAGAATGT----GGTAACTGCTGATAGCAAAGTTTCATCCAACTCTCCAGATTAT

ATGCAGA---CCAAATCCGAAAATCGGGCTAACGTGATTC-CACCTTATACAGGTCCACC

TCCAAACCGGCCAATGCGTAAGTCGCGCTTCGATCCGCCTCGACGATGTCGATAATATCC

CCGGGCTCACATGGCATGATAGGGTAACCAAATTGGTGCTGCTTTCT-------------

----------------------------------------------GGCAACAAATTTCC

GGCGAGCACGGTCTCGACGGCAATGGCGTGTATGTCTAGCAT--CCATGAAACCTGGGGA

AGACGAAGTAATGTTCTAACAT-CGTGAAACAGCTACAATGGAACCTCGGAGCTCCAGCT

CGAGCGCATGAGCGTTTACTTCAACGAGGTACGCCCGCACCAATCCACTAACGATCCTTA

TAACCGCGATGCTAACGCATTCGATATTTTAGGGTGCCGGCAACAAGTATGTCCCTCGCG

CCGTTCTCGTCGATTTGGAGCCTGGTACAATGGATGCCGTCCGTGCTGGTCCGTTCGGTC

AACTCTTCCGACCTGATAACTTCGTTTTCGGCCAGTCTGGTGCTGGCAACAACTGGGCCA

AGGGTCACTACACTGAGGGTGCTGAGCTGGTTGACAACGTTCTCGATGTCGTTCGTCGTG

AGGCTGAGGGCTGCGACTGCCTCCAGGGTTTCCAGATCACCCACTCGCTCGGTGGTGGTA

CCGGTGCTGGTATGGGTACTCTGCTCATCTCCAAAATCCGTGAGGAGTTCCCCGACCGCA

TGATGGCCACCTTCTCCGTTATGCCTTCTCCCAAGGTCTCGGACACTGTCGTCGAGCCCT

ACAACGCTACCCTCTCCGTCCACCAGCTGGTCGAGAACTCCGACGAGACCTTCTGCATTG

ACAACGAGGCCTTGTACGATATCTGCATGCGCACCCTAAAGCTGTCTAACCCCTCATACG

GTGATCTGAATCACCTCGTCTCCGCCGTCATGTCTGGTGTTACTACCTGCCTGCGTTTCC

CTGGTCAGCTTAACTCTGATCTGCGCAAGCTGGCTGTCAACATGGTGCCTTTCCCTCGTC

TGCACTTCTTCATGGTCGGCTTTGCCCCTCTCACTAGCCGCGGTGCGCACTCTTTCCGTG

CCGTCACCGTTCCCGAGTTGACTCAGCAAATGTTCGACCCCAAGAACATGATGGCCGCCG

CTGATTTCCGCAACGGTCGTTACCTCACATGCTCTGCCATCTTGTAAGAGTCGCCCCTTC

CCCCCTTCGTCGCGATGTGTTACTGACTTGATACTTCAAATAGCCGTGGTAAGGTTTCCA

TGAAGGAGGTCGAAGACCAGATGCGAAATGT

>Nemania_uda_CBS_148422

CTCTGTTTACGTTGCAACCCAATCAA-GCTCCGTCATTTCCCCCCTACTGATCCCTAAAC

GCGTCCC-----ACGCCCTTTGGCCCTCGCCCCTCCACACCTAGACACCAGCACCATCCC

CAGGAAACAACGT----GGTGGCTACCCAGCGCACAGATTTGTCCTATTCGCCAGATGGT

CCAGTGAAGAAATG-CATGAAAACAAGGCTAACCAACCCTTTCCCCTTCACAGGTCCACC

TCCAGACCGGCCAATGCGTAAGTCGCTCTTCGAGCTGCCACGATGACGTCGAGAACTTCC

CGCGGCTCACATGACATGATAGGGCAACCAAATTGGTGCTGCTTTCT-------------

----------------------------------------------GGCAACAAATTTCC

GGCGAGCACGGCCTCGACGGCAATGGCGTGTATGTCGACGGCATCTCTACAACATAGGCA

CAATAGAATGGCGAACTGACGTGGGTTGAACAGCTACAACGGATCCTCTGAGCTCCAGCT

CGAGCGCATGAGCGTCTACTTCAACGAGGTATGCCAGCGCGAATTCACCTCATCTTCGTG

GAGTCGAGATTCTAACGCGTTCGACTTTGTAGGGTGCCGGCAACAAGTACGTTCCTCGCG

CTGTTCTCGTTGATCTAGAGCCCGGTACTATGGACGCTGTCCGCGCTGGCCCTTTCGGTC

AGCTCTTCCGACCCGACAACTTCGTCTTCGGTCAGTCCGGTGCCGGCAACAACTGGGCCA

AGGGTCACTACACCGAGGGTGCTGAGCTGGTCGACCAGGTTCTGGATGTTGTCCGTCGCG

AAGCCGAGGGTTGTGACTGCCTTCAGGGCTTCCAGATCACCCACTCGCTTGGTGGTGGTA

CCGGTGCTGGTATGGGTACGCTGCTGATCTCCAAGATTCGTGAGGAGTTCCCCGACCGCA

TGATGGCCACCTTCTCCGTCATGCCCTCTCCCAAGGTCTCGGATACCGTCGTCGAGCCCT

ACAATGCCACCCTCTCTGTCCACCAGCTGGTTGAGAACTCAGATGAGACCTTCTGCATTG

ACAACGAGGCCCTTTACGATATCTGCATGCGTACCCTAAAGCTATCTAACCCCTCGTACG

GTGACCTGAACCACCTGGTCTCCGCTGTCATGTCCGGTGTCACGACCTGCTTGCGTTTCC

CCGGTCAGCTGAACTCCGACCTGCGCAAGCTGGCTGTGAACATGGTGCCTTTCCCTCGTC

TGCACTTCTTCATGGTCGGCTTTGCTCCTCTCACCAGCCGTGGGGCTCACTCCTTCCGCG

CTGTTACGGTTCCTGAGCTTACCCAGCAAATGTTCGACCCCAAGAACATGATGGCTGCCG

CAGACTTCCGCAACGGTCGTTACCTCACATGCTCTGCCATTTTGTAAGAACCTCTCTCCT

TTTGCTTCGGCATGGCCGGTTGCTAACTTCATACTCCCACCAGCCGCGGCAAGGTCTCCA

TGAAGGAAGTTGAGGACCAGATGCGCAACGT

>Nemania_ethancrensonii_CBS_148337

CTCTGTTTACGTTGTCACCCAATCGA-GCTCCATCTTTTTTT-CTGCC-TATCCCTAAAC

GCGTCCG-----ATGCCCTTCGGCCTCAACCTCCCCACATCTAGACACAAGCATCATCCC

CAGAAAACAACAT----GGTGGCTGTTCATCGCACAGATGTGTCCCATACGCCAGATCGT

CTAGAAGAAAAAAAGAAAGAACACCAGGCTAACCAAGCTTTTCTCGTTCACAGGTCCACC

TCCAGACCGGCCAATGCGTAAGTCGCTCTTCGAGAGTCCGCGACG---TCGAAACTCTTC

CACGACTCACAAGGCATAACAGGGTAACCAAATTGGTGCTGCTTTCT-------------

----------------------------------------------GGCAACAAATTTCC

GGCGAGCATGGCCTCGATGGCAATGGCGTGTATGTCCACGACATCTCTACAATAAAGGTT

CATTAAAATGGATGGCTGACATGGATGGGATAGGTACAACGGAACCTCTGAGCTCCAGCT

TGAGCGCATGAGCGTCTACTTCAACGAGGTATGCAAACCAAAATCTTCTTCCACCTGGTG

GAATCGAGATGCTGACAGGTTATGTTTGTTAGGGTGCCGGTAACAAGTATGTTCCTCGCG

CCGTTCTCGTCGATCTGGAGCCTGGTACTATGGACGCTGTCCGTGCTGGTCCTTTCGGTC

AGCTCTTCCGACCCGACAACTTCGTCTTCGGTCAGTCCGGTGCCGGCAACAACTGGGCCA

AGGGTCACTACACAGAGGGTGCTGAGCTGGTCGACCAAGTTCTGGATGTCGTCCGTCGCG

AGGCCGAGGGCTGTGACTGCCTCCAGGGCTTCCAGATCACCCACTCGCTTGGTGGTGGTA

CCGGTGCCGGTATGGGTACGCTACTGATCTCCAAGATCCGCGAAGAGTTCCCCGACCGCA

TGATGGCCACCTTCTCTGTCATGCCCTCTCCCAAGGTCTCGGATACCGTCGTCGAGCCCT

ACAATGCCACTCTCTCCGTTCATCAACTGGTTGAGAACTCCGATGAGACCTTCTGCATTG

ATAACGAGGCTCTGTACGATATCTGCATGCGTACCCTCAAGCTATCCAACCCCTCGTACG

GTGACCTGAACCACCTCGTCTCCGCTGTCATGTCTGGTGTCACAACCTGCCTGCGTTTCC

CTGGTCAGCTTAACTCTGATCTGCGCAAGCTGGCGGTGAACATGGTGCCATTCCCTCGTC

TGCACTTCTTCATGGTCGGCTTCGCTCCCCTCACGAGCCGTGGCGCCCACTCTTTCCGCG

CCGTTACCGTCCCCGAGCTCACCCAGCAGATGTTCGACCCCAAGAACATGATGGCTGCCG

CCGATTTCCGTAACGGTCGCTACCTGACATGCTCTGCGATCTTGTAAGAAACACTCTTTT

TCCTCTTCAC-ATGGCTATCTGCTAACTTCAC---TTTTCTAGCCGTGGCAAGGTTTCGA

TGAAGGAAGTCGAGGACCAGATGCGCAACGT

>Clypeosphaeria_mamillana_CBS_140735

------------------------------------------------CTATCTTTGAAC

ATGTCCA-----GC-------GACGT----CTTTACGT--CGATACATCACCATATCCTC

CAGC----------------GGCCCTCGCTGTCGCATCTTCCTCCACCTCGTAACGTC-C

TTACGA----GGCAACCACAGA-----GCTAACCAGATATTTCGCCTCTCTAGGTTCACC

TCCAGACCGGTCAATGCGTAAGTTTCTCTTCGATCCCCATCGGCG-CCTCACGAC-----

TCTCGCTCACAATATACAACAGGGTAACCAAATTGGTGCTGCTTTCTGGKGTGTGCCGAC

TGCAAATTTCGCGACTCGAGGCATCGATGTTGACTACGTTATTTTAGGCAGCAGATTTCC

GGCGAGCACGGCCTCGACGGTAGCGGAGTGTATGTTTACCTCTCCTGTAAAAAAAAGAAA

AAAAGCAGTGACTGGCTGACACGCTTTGAACAGTTACAATGGTACCTCCGAGCTCCAGCT

TGAGCGCATGAGCGTCTACTTCAACGAGGTATGCATTCGTCAGACT-GAACAACACCGTC

AAACCACCTTTCTTACC-ATGCCATTCTCTAGGGTTCCGGCAACAAGTACGTCCCTCGCG

CCGTCCTTGTGGATCTCGAGCCCGG-----------------------------------

------------------------------------------------------------

------------------------------------------------------------

------------------------------------------------------------

------------------------------------------------------------

------------------------------------------------------------

------------------------------------------------------------

------------------------------------------------------------

------------------------------------------------------------

------------------------------------------------------------

------------------------------------------------------------

------------------------------------------------------------

------------------------------------------------------------

------------------------------------------------------------

-------------------------------

>Digitodochium_amoenum_CBS_147285

CTCTGTTTACTTTAGAGCCCGACCAAACGAGCA---ACTCTCCCATCCCTATCCCTGAAC

GCGTCCGGGTCTGCCTTGCACCGTCTCAA-CTCTCCACCCCTCCAT-----CTC------

-----------------------------------GCCCTCAACATGTCCCCCAGATTGC

C---------------ATGAGA----AGCTAACCTTGTTTTCCGCCTCTCTAGGTTCATC

TTCAAACCGGCCAATGCGTAAGTCACCTTCGACTCCTCC----------CCCGACGTCCT

CATCGCTCACATAGAATAACAGGGTAACCAAATTGGTGCTGCTTTCTGGTGTGTACCGAC

CGCACGATACTCGAATTGTGCCATCAATATTGACAGGGGCGTTCTAGGCAACAGATTTCC

GGCGAACATGGTCTCGACGGCAACGGAGTGTATGTCTACCCAACAGAC--ACCTACCCGC

TGGTGATATGATTGGCTGACCA-CGTTCAACAGTTACAATGGAACCTCTGAGCTCCAGCT

CGAGCGCATGAGCGTCTACTTCAACGAGGTACGCAAAGGCCAGGCCAG-ACGAAATTTCG

AAGCCGACTTTCTAACC-GTGAAACACTCTAGGGTGCTGGCAACAAGTACGTCCCCCGTG

CCGTCCTCGTCGATCTCGAGCCCGGTACCATGGATGCCGTCCGTGCTGGTCCTTTCGGTC

AGCTGTTCCGTCCCGACAACTTCGTTTTTGGCCAGTCCGGTGCTGGCAACAACTGGGCCA

AGGGTCACTACACTGAGGGCGCTGAGCTCGTCGATCAGGTCCTCGATGTTGTCCGTCGCG

AGGCTGAGGGCTGCGACTGCCTCCAGGGCTTCCAGATCACCCACTCTCTCGGTGGTGGTA

CTGGTGCTGGTATGGGTACGCTGCTTATCTCAAAGATTCGCGAGGAGTTCCCCGACCGCA

TGATGGCCACTTTCTCCGTCGTGCCCTCTCCTAAGGTTTCCGACACCGTCGTCGAGCCCT

ACAACGCCACCCTTTCCGTTCACCAGCTTGTCGAGAACTCGGACGAGACCTTCTGCATTG

ATAACGAGGCTCTCTACGATATCTGCATGCGTACCCTTAAGCTGTCCAACCCCTCTTACG

GTGACCTGAACCACCTTGTCTCTGCCGTCATGTCCGGCGTTACCACTTGCTTGCGTTTCC

CTGGCCAGCTCAACTCTGATCTGCGCAAATTGGCTGTCAACATGGTGCCTTTCCCTCGTC

TCCATTTCTTCATGGTTGGCTTCGCCCCTCTGACCAGCCGTGGCGCTCACTCTTTCCGTG

CCGTCACTGTCCCCGAGTTGACCCAGCAGATGTTCGACCCCAAGAACATGATGGCTGCCT

CGGACTTCCGCAACGGTCGCTACTTGACCTGCTCTGCCATCTTGTGGGTTATCTTTCAGA

GCCCTTCCCGTGAACTTAGATGCTAACA------TCCCCCCAGCCGTGGCAAGGTTTCTA

TGAAGGAGGTTGAGGACCAGATGCGCAACGT

>Occultitheca_rosae_HKAS_102393

TTTTACTTGCTGCTTGACCCGACCATACACACAAGAACTCCTCCCATCTTATCCATGAAC

GCGTCCG-GCC-AT-------GATGCCAA-CTCTCCAC-------------CTTCTTCT-

CAGC------------TTGCAACA-TCTATGACACATCTTCGCCCTATTCGCCGTGTTGT

TTGGAC----------AGAAGGATTGTGCTAACCATATTTTTCGCGTCTCTAGGTTCACC

TCCAGACCGGCCAATGCGTAAGTCGCCA--CCATCCTTC-CAG----TCCTCAACGTCGC

CATTGCTCACATTCAATAATAGGGTAACCAAATTGGTGCTGCTTTCTGGTGTGTACCGCC

TACACCAACTACGCCCTTTGGTAGCCGTATTGACTGGGCAACTACAGGCAGCAGATCTCC

GGCGAGCACGGTCTCGACGGCAGCGGAGTGTATGT---CGAC--CCTTG-AACTCCCTCC

TAACGAATTCATTGGCTGACCTGGGCTCAACAGTTACAATGGAACCTCGGAGCTCCAGCT

TGAGCGCATGAGCGTCTACTTCAACGAGGTACCTACCCGCCAGATACGAACGATATCCCG

AACCCCATTTTCTAACCGTGAAACAATAACAGGGTGCCGGTAACAAGTATGTGCCCCGCG

CCGTCCTCGTCGATCTCGAGCCCGGTACCATGGATGCCGTCCGCGCTGGTCCCTTCGGTC

AGCTCTTCCGTCCCGACAACTTCGTCTTCGGTCAGTCCGGTGCTGGCAACAACTGGGCCA

AGGGTCACTACACTGAGGGTGCCGAGCTCGTCGACCAGGTTCTCGATGTCGTCCGCCGTG

AGGCCGAGGGCTGTGACTGCCTCCAGGGTTTCCAGATTACCCACTCTCTCGGCGGTGGTA

CCGGTGCCGGTATGGGTACGCTCCTGATCTCCAAGATCCGTGAGGAATTCCCCGACCGCA

TGATGGCCACCTTCTCCGTCGTGCCCTCCCCCAAGGTTTCCGACACCGTTGTTGAGCCCT

ACACGTCAACCTCTTCCGTCCACCAGCTTGTCGAAAACTCGGACGAGACCTTCTGCAT--

------------------------------------------------------------

------------------------------------------------------------

------------------------------------------------------------

------------------------------------------------------------

------------------------------------------------------------

------------------------------------------------------------

------------------------------------------------------------

-------------------------------

>Magnostiolata_mucida_MFLU_19_2133

-----------GCCCAGCCCAAT---TCG-------CCTCGCTCCATCCTAGCCCTACAC

GCGTCCGTGTCTCTGCTCCGTGACAT----CTATCCGT------GC-----CTCCATCTG

AAGC------------TCACAATCGACGGTA--ACAGCC--ATCT-----------TTA-

---------------------------GCTAACCATATCTTTCGCGTCTCTAGGTCCACC

TTCAGACCGGTCAATGCGTGAGTCGCCT--CGCTCGACAGCAACCACTTCTAAACGTCGT

CA-CGCTCACATATAACCACAGGGTAACCAAATTGGCGCTGCCTTCTGGTGTGTACC---

TA----AAGGAAGGCCTCAGGCATCAATATTGACTCGGATATTCTAGGCAGCAGATTTCC

GGCGAGCACGGTCTCGACGGCAATGGCGTGTAGGTTGCTGAG------AAAACTGCCCCC

-AG-GAAAGCGGTGACC-ACTTT--CTCAACAGGTACAATGGTACTTCGGAGCTCCAGCT

TGAGCGCATGAGCGTTTACTTCAACGAGGGGCGCAGGGACCAAACCCGTTCCTTCCCGCA

AAATTCACTTTC-AAACATAAAGTGATA-TAGG---------------------------

------------------------------------------------------------

------------------------------------------------------------

------------------------------------------------------------

------------------------------------------------------------

------------------------------------------------------------

------------------------------------------------------------

------------------------------------------------------------

------------------------------------------------------------

------------------------------------------------------------

------------------------------------------------------------

------------------------------------------------------------

------------------------------------------------------------

------------------------------------------------------------

------------------------------------------------------------

-------------------------------

>Anthostomelloides_krabiensis_MFLUCC_15_0678

------------------------------------------------------------

------------------------------------------------------------

------------------------------------------------------------

------------------------------------------------------------

------------------------------------------------------------

------------------------------------------------------------

------------------------------------------------------------

------------------------------------------------------------

------------------------------------------------------------

------------------------------------------------------------

------------------------------------------------------------

------------------------------------------------------------

------------------------------------------------------------

------------------------------------------------------------

------------------------------------------------------------

------------------------------------------------------------

------------------------------------------------------------

------------------------------------------------------------

------------------------------------------------------------

------------------------------------------------------------

------------------------------------------------------------

------------------------------------------------------------

------------------------------------------------------------

------------------------------------------------------------

------------------------------------------------------------

-------------------------------

>Linosporopsis_ischnotheca_CBS_145761

CTCTGTTTACTCCAGAGCCCAACCGC-TGGCCATCGGCATTGATGTACCACCCCACGAAC

GCGCTCG------CCATCACTGATCTTTGCCCTTCCTCAC--GTACCTGGACAACATTAT

TGTCGAGGCATGC----TGT-GTTGGTGGGAGCACGGCT--GTCTGGCCCACTAGACAAC

TTGCTG----------GCAAGAATTGAGCTAACCGTGCATTTCGCGTCTACAGGTCCATC

TTCAGACTGGCCAATGCGTAAGTCGCCTCCCTCGCTT--CCGATGACTCCCCGAGTCTTA

AACAGACTAACCAAAATAACAGGGCAACCAAATTGGTGCTGCTTTCTGGTGCGTACCGAA

CAGACAACTCGCAGCCGTTGGCGGGGTTACTAACCATTGTGCTCCAGGCAACAGATTTCC

GGCGAGCATGGCCTCGACGGCAATGGCGTGTACGTACACACC--CAGCTCAATTTGCCTT

TTCTGAA-------ACTGACCTT---TAGACAGTTACAATGGAACTTCCGAGCTACAGCT

CGAGCGCATGAGCGTTTACTTCAACGAGGTATGCGACTACGAGTCTGCTCATACCACCTA

AAGC-ACCATTTTAATCCCATCTTCTTTCTAGGGAGCTGGCAACAAATATGTCCCCCGCG

CCGTCCTCGTCGATCTTGAGCCCGGTACCATGGATGCTGTCCGTGCCGGTCCTTTCGGCC

AACTCTTCCGTCCCGACAACTTCGTCTTCGGCCAATCCGGTGCCGGCAACAACTGGGCGA

AGGGTCACTACACCGAGGGTGCTGAGCTCGTTGATCAGGTTCTCGATGTCGTTCGCCGCG

AGGCTGAGGGATGCGACTGCCTTCAGGGTTTCCAAATCACACACTCGCTCGGTGGTGGTA

CCGGTGCCGGTATGGGAACGCTATTGATCTCCAAAATCCGTGAAGAATTCCCCGACCGCA

TGATGGCTACCTTCTCCGTCGTTCCCTCGCCCAAGGTGTCGGACACCGTCGTCGAACCCT

ACAACGCCACCTTGTCCGTTCATCAACTGGTGGAGAACTCGGACGAGACGTTCTGTATCG

ATAACGAGGCGCTGTACGACATCTGCATGCGTACCCTTAAGCTGTCAAACCCTTCATACG

GTGACTTGAACCATCTTGTCTCCGCTGTCATGTCTGGTGTCACCACTTGCTTGCGTTTCC

CTGGACAGCTCAACTCCGATCTGCGCAAGTTGGCCGTGAACATGGTGCCGTTCCCTCGTC

TGCACTTCTTCATGGTCGGCTTCGCTCCTCTGACCAGCCGTGGTGCCCACTCGTTCCGCG

CCGTCACCGTCCCCGAACTTACCCAGCAAATGTTCGACCCCAAGAACATGATGGCTGCCT

CGGATTTCCGCAATGGTCGCTACCTGACCTGCTCTGCTATCTTGTAAGTCACTTATGACC

GTGCCTTGTGCTCGATTTGCTTGTGAAAGCTAACATTCCCTAGCCGTGGCAAGGTGTCGA

TGAAGGAGGTCGAGGATCAGATGCGTAATGT

>Linosporopsis_ochracea_CBS_145999

CTCTGTTTACTTTGAGGCCCAACCGACGGGCCACCAGCTTCAATGGGGCCATCCCTGAAC

GCGTCCG------CGATCCGTGGTTTTGGTCTCTCTTCAC--GGACCTAGAGAACCACGT

TGTCAAAACAAGT----TAT-GTTGGCAGAGATACGGTCCAAAACGGC--------CTAT

CTGGAA----TCAAGGTCGAGAGTTGAGCTAACCGTGTGTTTCGCACCTACAGGTCCATC

TTCAGACCGGTCAATGCGTAAGTTGTCTATCTACCTT--TCGACCACCCCGCGAATGTTC

GTGAACTGACGGGACATAACAGGGTAACCAAATCGGTGCTGCTTTCTGGTGCGTGCTAGG

CAGACAATTTGCTAGCGTGGGCGTGAATGTTGACTTGCGTGCTCCAGGCAACAGATTTCG

GGCGAGCATGGCCTTGACGGCAATGGCGTGTACGTACCTGGCAACGACTC---AGACTCG

CATCGAACAAGCAAACTGACACT---CATAAAGCTACAACGGAACCTCTGAGCTCCAGCT

CGAGCGCATGAGCGTCTACTTCAACGAGGTTAGTTACTACAAGTCTGTTCTCACCGCGCA

AAGC-AGCTTTTTAATCCCATCTTTCCTCTAGGGTGCCGGCAACAAATATGTCCCCCGCG

CCGTCCTCGTCGATCTTGAGCCTGGTACCATGGATGCTGTCCGCGCCGGTCCTTTCGGTC

AGCTGTTCCGTCCCGACAACTTCGTCTTCGGCCAATCCGGTGCCGGCAACAACTGGGCCA

AGGGTCACTACACCGAGGGTGCTGAGCTTGTCGACCAAGTTCTCGATGTCGTTCGCCGCG

AGGCTGAGGGCTGCGACTGCCTGCAGGGTTTCCAGATCACCCACTCGCTCGGTGGTGGTA

CCGGTGCTGGTATGGGTACGCTGTTGATCTCCAAGATTCGCGAGGAATTCCCCGACCGCA

TGATGGCTACTTTCTCGGTTGTCCCCTCGCCCAAGGTGTCCGACACCGTCGTCGAGCCCT

ACAACGCCACCCTCTCAGTCCACCAGCTGGTTGAGAACTCGGACGAGACCTTCTGTATCG

ACAACGAGGCGCTGTACGACATCTGCATGCGTACCCTCAAGCTGTCAAACCCCTCATACG

GTGACCTGAACCACCTCGTCTCCGCCGTCATGTCTGGTGTCACCACTTGCCTGCGTTTCC

CTGGACAGCTCAACTCCGACCTGCGCAAGCTGGCTGTGAACATGGTGCCGTTCCCCCGTC

TGCACTTCTTCATGGTCGGCTTCGCTCCTCTGACCAGCCGTGGTGCCCACTCGTTCCGCG

CTGTCACCGTCCCCGAGTTGACCCAGCAAATGTTCGACCCCAAGAACATGATGGCTGCCT

CGGATTTCCGCAACGGTCGCTACCTGACCTGCTCTGCTATCTTGTAAGCCATCCCGAAAG

CTGCCTCGTGGATGACTCGTTACTTGAAACTAACATTTCCCAGCCGTGGTAAGGTGTCAA

TGAAGGAGGTTGAGGACCAGATGCGTAATGT

>Emarcea_castanopsidicola_CBS_117105

CTCTGTTTACCTTGGAGCCCAACCCGAGCCGGA---ATCGAACAAGCCTTGTCCCTGGAC

GCGTTGAGTTCGGTACTAGATGGTTCTTGCCGGCTTGCTC-GA-ACCTCATATTTATTTC

TTGC--CT-------------GTCACAAATAGCATTGTTTCATGA---ATGCTCGTCCAT

CCACCAGAACCACCTACGAAGAATTCTGCTGACCGTGTTTTTTCTGCGAATAGGTTCACC

TCCAGACCGGTCAGTGCGTAAGTAACATCCCGACCC---CCAAAGTCATCGAAGCAAACA

TAGAACTGACACATGATGATAGGGTAACCAAATTGGTGCTGCTTTCTGGTGTGTACCACG

CGCGTCCAACGGTGTTACCGTCTCGTATATTGAC-ATCAACCTCCAGGCAACAGATCTCC

GGCGAGCACGGTCTCGACGGCAATGGCGTGTATGTTTACAGCATCGATTG---ACCTTTG

----AAAGGCAATGACTGACC--TTTGACATAGCTACAATGGCACCTCTGAGCTCCAGCT

CGAGCGCATGAGCGTCTACTTCAACGAGGTACGTCAACCATAGGTCGTGATCTCTAGAAA

CAAG-CATGGCTGACCGAATCCTCTTCTTCAGGGTGCCGGCAACAAGTACGTCCCCCGCG

CCGTCCTCGTCGATCTCGAGCCCGGTACCATGGACGCCGTCCGCGCCGGTCCCTTCGGCC

AGCTCTTCCGCCCCGACAACTTCGTCTTCGGCCAGTCCGGTGCCGGCAACAACTGGGCCA

AGGGTCACTACACCGAGGGTGCTGAGCTCGTCGACCAGGTCCTCGACGTCGTCCGCCGCG

AGGCCGAGGGCTGTGACTGCCTCCAGGGTTTCCAGATCACCCACTCCCTCGGTGGTGGTA

CCGGTGCCGGTATGGGTACGCTCTTGATCTCCAAGATCCGCGAGGAGTTCCCCGACCGCA

TGATGGCCACCTTCTCCGTCGTCCCCTCCCCCAAGGTCTCCGACACCGTCGTCGAGCCCT

ACAACGCCACCCTCTCCGTCCACCAGCTCGTCGAGAACTCGGACGAGACCTTCTGCATCG

ACAACGAGGCCCTCTACGACATCTGCATGCGTACCCTCAAGTTGTCCAACCCCTCCTACG

GCGACCTCAACCACCTCGTCTCCGCCGTCATGTCCGGCGTCACCACCTGTCTGCGTTTCC

CCGGTCAGCTGAACTCTGACCTGCGCAAGCTCGCCGTCAACATGGTGCCCTTCCCCCGTC

TGCACTTCTTCATGGTCGGCTTCGCCCCCCTCACCAGCCGCGGCGCCGGTGCTTTCCGCG

CCGTCACCGTCCCCGAGCTCACCCAGCAGATGTTCGACCCCAAGAACATGATGGCTGCCT

CGGACTTCCGCAACGGTCGCTACCTCACCTGCTCTGCCATCTT-----------------

-------------------------------------------CCGCGGCAAGGTCTCCA

TGAAGGAGGTCGAGGACCAGATGCGCAAC--

>Emarcea_eucalyptigena_CBS_139908

CTCTGTTTACCTTGGAGCCCAACCCGAGCCGGA---ATCGAACAAGCCTTGTCCTTGGAC

GCGTTGAGTTCGATATTAGATGGTTCTTGCAGACTGGCTC-GG-ACCTCGGATTCATCAT

TTGC--CT-------------GTCACAAATAGCATTGTTTCATTA---ATGTCACTCCAT

ATGCCAGAGCCGCCCACGAAGAACTCTGCTAACCGTGTTTTCTGCG-ATCTAGGTTCACC

TCCAGACCGGTCAGTGCGTAAGTAGCATCTTGACTCCCAACCAAGTCGTCGAGCTTGACA

TGTAACTAACGCACGATATCAGGGTAACCAAATTGGTGCTGCTTTCTGGTATGTACCACG

CGCGTCAAACGGTGTTACCGTCTCGTATATTGAC-ATCAACCTCCAGGCAACAGATCTCC

GGCGAGCACGGTCTCGACGGCAATGGAGTGTATGTTTACAGCATCAACCT---ATATGTA

----GAAAGCATTGACTGACC--TTTGACATAGCTACAATGGCACCTCTGAGCTCCAGCT

CGAGCGCATGAGCGTCTACTTCAACGAGGTACGTCAACAACATCATATCATCCCTGGAAA

CAACACATGGCTAACTGGACTCTTTGCTTTAGGGTGCCGGCAACAAGTACGTCCCCCGCG

CCGTCCTCGTCGATCTCGAGCCCGGTACCATGGACGCCGTCCGCGCTGGTCCCTTCGGTC

AGCTCTTCCGCCCCGACAACTTCGTCTTCGGCCAGTCCGGTGCCGGCAACAACTGGGCCA

AGGGTCACTACACCGAGGGTGCTGAGCTTGTCGACCAGGTCCTCGACGTCGTCCGCCGCG

AGGCCGAGGGCTGTGACTGCCTCCAGGGTTTCCAGATCACCCACTCCCTCGGTGGTGGTA

CCGGTGCCGGTATGGGTACGCTCTTGATCTCCAAGATCCGCGAGGAGTTCCCCGACCGCA

TGATGGCCACCTTCTCCGTCGTCCCTTCCCCCAAGGTCTCCGACACCGTCGTCGAGCCCT

ACAACGCCACCCTCTCCGTCCACCAGCTCGTCGAGAACTCGGACGAGACCTTCTGTATCG

ACAACGAGGCCCTCTACGACATCTGCATGCGTACCCTCAAGCTCTCCAACCCCTCTTACG

GCGACCTCAACCACCTCGTCTCCGCCGTCATGTCCGGTGTCACCACCTGTCTCCGCTTCC

CCGGTCAGCTGAACTCTGACCTGCGCAAGCTCGCCGTCAACATGGTGCCCTTCCCCCGTC

TGCACTTCTTCATGGTCGGCTTCGCCCCCCTCACCAGCCGCGGCGCCGGTGCCTTCCGCG

CCGTCACCGTCCCCGAGCTCACCCAGCAGATGTTCGACCCCAAGAACATGATGGCTGCCT

CGGACTTCCGCAACGGTCGCTACCTCACCTGCTCTGCCATCTT-----------------

-------------------------------------------CCGCGGCAAGGTCTCCA

TGAAGGAGGTCGAGGACCAGATGCGCAAC--

>Kretzschmaria_deusta_CBS_163.93

---------------------T-CAA-GCTCGGTCGATATTCGCTGCCCCAAGCCAGGAC

GCGTCCCGCCCTATGCTTCTTGACCTCCGCCTCTCCACATCCGC--ACAACCATCATCTG

CAGTGGACGATGT----GGTGGCGACCAATAGCACAGCTTGATTCAACCGGCCAACTTGT

CTAGCA----TGTTTCATGAAAACCAGGCTAACCGCGTTCTTCCCATCTCCAGGTTCACC

TCCAAACCGGCCAATGCGTAAGTGCTCT--CAATCCTCGATGACGACGTAAAGAATCTCC

CAAGGCTCACATTGCATGACAGGGTAACCAAATCGGTGCTGCTTTCT-------------

----------------------------------------------GGCAACAAATCTCT

GGCGAGCACGGTCTCGATGGCAGTGGCGTGTATGTCTATGTCACCTATGGAGCAGAACGA

CGACGCAATAGCCGACTAACCATCACGGAACAGGTACAATGGAACCTCTGAGCTCCAGCT

CGAGCGCATGAGTGTTTACTTCAACGAGGTACGCAACCACCGCGTCATGTTGGTCCCGTG

CAACCAACGTTTTAACATG---AAATTTCTAGGGTGCCGGAAACAAGTATGTCCCTCGCG

CCGTCCTCGTCGATTTGGAGCCCGGCACCATGGACGCCGTCCGCGCTGGTCCCTTCGGTC

AGCTCTTCCGACCCGACAACTTCGTCTTCGGCCAGTCTGGTGCTGGCAACAACTGGGCCA

AGGGTCACTACACCGAGGGTGCTGAGCTTGTCGACACCGTTCTCGATGTCGTTCGTCGCG

AGGCCGAGGGCTGCGACTGCCTCCAGGGTTTCCAAATCACCCACTCGCTCGGTGGCGGCA

CCGGTGCTGGTATGGGTACACTGCTGATCTCCAAGATCCGCGAGGAGTTCCCTGACCGCA

TGATGGCCACCTTCTCCGTCATGCCCTCGCCCAAGGTTTCGGACACCGTCGTCGAGCCTT

ACAATGCCACGCTCTCCGTCCACCAGTTGGTCGAGAACTCCGATGAGACCTTTTGCATTG

ATAACGAGGCTCTGTACGATATCTGCATGCGCACCTTGAAGCTATCCAACCCATCATACG

GTGACTTGAACCACCTTGTCTCCGCTGTCATGTCTGGCGTAACCACCTGCCTGCGTTTCC

CCGGTCAGCTTAACTCTGATCTGCGCAAGTTGGCCGTGAATATGGTGCCCTTCCCGCGTC

TGCACTTCTTCATGGTCGGCTTTGCTCCTCTCACCAGCCGTGGCGCTTACTCTTTCCGCG

CCGTCACCGTGGCCGAGCTGACCCAGCAAATGTTTGACCCCAAGAACATGATGGCCGCCG

CCGACTTCCGTAACGGTCGCTACCTCACATGCTCTGCTATTTTGTAAGAACTCGCTCTCG

TACCTTTCCACACAGCCAATTGCTAATTTGATGCGTTTTTCAGCCGTGGCAAGGTCTCCA

TGAAGGAGGTTGAGGACCAGATGCGAAA---

>Collodiscula_japonica_CBS_124266

------------------------------------------------------------

------------------------------------------------------------

------------------------------------------------------------

------------------------------------------------------------

-----ACTGGCCAATGTGTGAGTATCTCGGCGTTCTTGCCTTACTATGCCTCAGC---TT

CAAGGCCACCATGATTGTACAGGGCAACCAAATCGGCGCTGCCTTCT-------------

----------------------------------------------GGCAGAATATCTCG

GGCGAGCACGGTCTGAACACCGATGGCATGTGAGCAATCTCCGGTCATCCATCTAACCGT

AAACAGAGTGCTCAGCTATCGCA---TTTAAAGATACGAAGGTACCTCGGATTTACAATT

GGAACGCATGAATGTCTATTTCAATGAGGTATGGGCCT--------ACCGTCTGAGTGTG

TTGGCCTCAAAGCAGAGTGCTCACAAACGTAGGCGTCCCACAACAAATACGTGCCTCGTG

CCGTGTTAGTTGATTTGGAGCCGGGCACGATGGACGCAGTGCGTGCCGGCCCCCTTGGCC

AAATGTTCCGTCCAGACAACATCGTTTTTGGACAGTCGGGTGCTGGCAACAATTGGGCAA

AAGGCCATTACACCGAAGGAGCCGAACTGGTTGACCAGGTTCTTGATGTTGTTCGCCGGG

AGACCGAGGGCTGCGATTGCCTTCAGGGCTTTCAAATCACGCATTCGTTGGGTGGTGGTA

CTGGTTCTGGTATGGGGACACTGCTTGTCTCCAAGATTCGTGAAGAGTTCCCGGACCGTA

TAATGGCTACTTTCAGTGTCATTCCGTCACCAAAGGTGTCTGATACTGTCGTCGAACCGT

ATAATGCCACGCTCTCGATCCATCAGTTGGTTGAAAACTCAGAGGAAACTTTCTGCATCG

ACAACGAGGCCCTCTATGATATCTGCCAACGTACTCTAAAGCTTACCAACCCTTCATATG

GCGACCTAAATCACCTTGTGTCAGCTGTTATGTCTGGCGTTTCGACCTCTCTACGCTTCC

CTGGGCAATTGAATTCAGATCTGCGTAAATTGGCTGTAAACATGGTGCCGTTTCCCCGTC

TCCATTTCTTTATGGTCGGTTTTGCACCTTTGACTAGTCGTGGCGCCTATTCTTTCCGCG

CAGTGACTGTGCCAGAACTCACCCAACAGATCTTCGACCCTAAGAACATGATGACGGGCT

CTGACTTTCGTAACGGACGGTACCTGACATGCTCTGCAATCTT-----------------

-------------------------------------------CCGAGGAAAAATCGCAA

TGAAAGAGGTCGAGGAACAGATGCGCAA---

>Daldinia_concentrica_CBS_113277

----------------GCCCAGT--A-------TCA------CTAGAGAAACCCCTGAAC

GCGTCCGA---AAA------------------------AACTCC----------------

----AAAACATCCTTATTCTACCCCTCATACACAAAACGCTACCATATCTACATTTTATA

TTGCAACTACACCAACGTGAAATCAAAGCTAACCGCGTTT----CTTCAATAGGTTCATC

TTCAGACTGGCCAATGTGTAAGTAACAG--CGATCATCGAGAACCATATATAAGACACAG

CGGGGCTCACATGAGATGATAGGGTAACCAAATCGGTGCCGCTTTCT-------------

----------------------------------------------GGCAAACCATCTCT

AGCGAGCACGGTCTCGACAGCAATGGAGTGTATGTATTCGAA--TTGTTGATTCCCAT--

CGACGAGAATATCAACTAATCATCCATCAACAGTTACAACGGTACTTCCGAGCTCCAGCT

CGAGCGCATGAGCGTCTACTTCAACGAGGTATGAATT-TGTAGGAA--TAGGGATAAATA

AACGGAATTGCTAATTGCCTCAACGCGTGCAGGCTTCTGGCAACAAGTATGTTCCTCGTG
[truncated: 193,627 more chars]
